# Advantages of Asymmetry: A Synthetic and Structural Exploration of *s*‐Triazine Chemistry Starting From Pyrrolated Ammeline and Melam

**DOI:** 10.1002/open.70252

**Published:** 2026-07-13

**Authors:** Thaddäus J. Koller, Alexander Pichler, Johannes N. Singer, Zehua Xu, Vasiliki Valsamidou, Reinhard M. Pritzl, Wolfgang Schnick

**Affiliations:** ^1^ Department of Chemistry University of Munich (LMU) Munich Germany

**Keywords:** azo compounds, N ligands, reaction mechanisms, solid‐state structures, synthetic methods

## Abstract

Melam and ammeline are simple, *s*‐triazine‐based compounds first described by Liebig nearly 200 years ago. Outgoing from these two compounds, synthetic strategies for asymmetrically substituted *s*‐triazines were developed. As the initial key step, Clauson–Kaas pyrrolation of the compounds’ primary amino groups was carried out, significantly improving their solubility in organic solvents and thereby facilitating further conversions. These include functionalization of the *s*‐triazine bridging amino group of pyrrolated melam by reaction with electrophiles, while pyrrolated ammeline was deoxychlorinated and subsequently reacted with nucleophiles to displace the resulting Cl substituent. Moreover, a reaction protocol for reversion of pyrrolyl into amino groups was developed, which involved ozonolysis, followed by treatment with aqueous NaOCl of the resulting formamide derivatives. Thereby, melam derivatives with essentially the same coordination site but enhanced solubilities were obtained. This enabled the preparation of a Cu(II) coordination complex from aqueous solution, which is unheard of for melam itself. Finally, the thus accessible *s*‐triazines were structurally characterized to gain deeper insights on how the different conducted transformations influence the physicochemical properties relevant for future applications.

## Graphical abstract

The chemistry of melam and ammeline has remained virtually unexplored, despite being known for nearly two centuries. Therefore, synthetic strategies for asymmetrically substituted *s*‐triazines have been developed that exploit their latent potential as synthetic building blocks. The thus accessible *s*‐triazines were additionally structurally characterized to better comprehend their observed physicochemical properties.

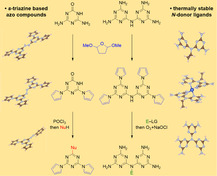

## 1 Introduction

**SCHEME 1:**
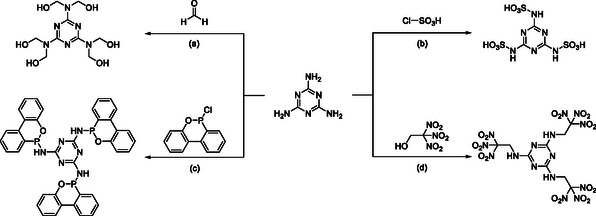
Exemplary syntheses of functional *s*‐triazines starting from melamine: preparation of a thermosetting plastic precursor by reaction with formaldehyde (a) [8, 9], a solid‐state acid catalyst by reaction with chlorosulfonic acid (b) [10, 12], a flame retardant by reaction with 6‐chloro‐6*H*‐dibenzo[*c*,*e*][1–5]oxaphosphinine (c) [13], and a high‐energy‐density material by reaction with 2,2,2‐trinitroethan‐1‐ol (d) [14, 15].

1,3,5‐Triazine, also known as *s*‐triazine (*s* = symmetrical), is an aromatic heterocycle consisting of three alternating carbon and nitrogen atoms. Its derivatives are being investigated for a broad array of applications such as pharmaceuticals [1, 3], herbicides [4, 5], or flame retardants [6, 7]. A readily available and commonly employed starting material for *s*‐triazine syntheses is melamine (i.e., 2,4,6‐triamino‐*s*‐triazine). Its main industrial use is in combination with formaldehyde, whose addition products are excellent precursors for thermosetting plastics [8, 9]. However, melamine’s amino groups can also be reacted with other electrophiles for the preparation of further functional *s*‐triazines (Scheme 1) [10, 15].

**SCHEME 2:**
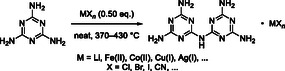
Reported synthetic conditions for the preparation of melam coordination complexes [17, 19].

Melamine’s trivial name is derived from melam (i.e., bis(4,6‐diamino‐*s*‐triazin‐2‐yl)amine), of which it is the formal aminolysis product. Both were named and first studied by the famous chemist Justus von Liebig [16]. Melam is nowadays investigated as a bidentate ligand with high thermal stability and excellent resistance against oxidation, which is attributable to the fact that it is composed exclusively of C─N and N─H bonds [17, 19]. Moreover, the resulting coordination complexes are considered promising precursors for polymeric carbon nitrides (PCN), which are being studied as heterogeneous photocatalysts, particularly for H_2_O splitting [20, 22]. They can conveniently be prepared by solid‐state reaction of melamine with the corresponding metal salt (Scheme 2).

**SCHEME 3:**
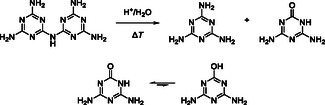
Hydrolysis of melam by treatment with hot acid (top) and depiction of the equilibrium between ammeline’s two most prevalent tautomers (bottom).

The conversion of melamine under similar conditions with an NH_4_^+^ salt like NH_4_Br or NH_4_I leads to the formation of the corresponding melamium salt [23, 25]. Alternatively, melamium salts can be obtained by direct reaction of melam with acids such as aqueous HClO_4_ and HCl [17, 19, 26]. However, when subjected to hot acid for prolonged periods of time, it gets hydrolyzed to melamine and ammeline (i.e., 4,6‐diamino‐*s*‐triazin‐2(1*H*)‐one) [16, 26]. Ammeline is formally obtained by replacing one of melamine’s amino groups with a hydroxyl group, when considering the enol tautomer of the former (Scheme 3).

Ammeline is discussed as part of insensitive high‐energy‐density materials (HEDM) due to its reasonably high nitrogen content as well as its moderate basicity (p*K*_b_ = 9.5) [27], which allows it form salts with a wide variety of oxidizing acids such as HNO_3_ and HClO_4_ [28, 32]. These salts can be expected to have superior energetic performances compared to their melamine analogs due to ammeline’s improved oxygen balance. A second distinctive feature of ammeline compared to melamine is its moderate acidity (p*K*_a_ = 9.4) [27], which enables it to also form stable salts containing deprotonated ammeline from aqueous solution [31].

While the acid–base and coordination chemistry of ammeline and melam is well understood, the reactivity of their functional groups against electrophiles has remained virtually unexplored so far. This is despite the fact that both compounds should be better suited as starting materials for the preparation of asymmetrically substituted *s*‐triazines compared to the otherwise commonly employed melamine, whose three identical functional groups make the synthesis of such compounds challenging. The main two barriers that have so far hampered their use as synthetic building blocks are their higher commercial price [33] and their generally far poorer solubility [34, 35] when compared with melamine.

Therefore, the aim of this work was to provide simple access to both compounds and then overcome the hurdle posed by their low solubility to enable an extensive exploration of their chemistry through their functional groups for the synthesis of asymmetrically substituted *s*‐triazines. In the case of ammeline, a cost‐effective and easily scalable synthetic protocol recently reported by our group was utilized [31]. For the preparation of melam, modern synthetic approaches are employing either a sealed glass ampoule [17, 36] or an ammonothermal autoclave [37]. This prevents easy scalability into the gram scale in the case of the first or requires dedicated equipment in the case of the latter. Therefore, a straightforward muffle furnace synthesis for high‐purity melam was developed, which alleviates both aforementioned issues. As the next key step, ammeline and melam were reacted with the electrophile 2,5‐dimethoxyTHF. This not only made them readily soluble in most organic solvents by severe polarity reduction through transformation of their primary amino groups into pyrrolyl groups but also left their remaining functional groups untouched, enabling them to be used as the basis for further transformations. This type of reaction, known as a Clauson–Kaas pyrrole synthesis [38], has also been shown to be feasible with melamine’s amino groups [39] and of its tri‐*s*‐triazine/*s*‐heptazine analog melem [40].

For pyrrolated ammeline, a deoxychlorination was carried out to obtain an *s*‐triazine with a reactive Cl substituent, which could be successfully displaced by a series of nucleophiles. Pyrrolated melam, on the other hand, was reacted with several electrophiles to functionalize its *s*‐triazine bridging secondary amino group. For the resulting products, a reaction protocol was developed to revert their pyrrolyl into amino groups to obtain compounds with essentially the same coordination site as melam but enhanced solubility. This allowed the preparation of a Cu(II) coordination complex from aqueous solution, which has never been reported for melam itself, as the harsh reaction conditions of the aforementioned solid‐state synthesis method lead to the reduction of Cu(II) to Cu(I) (Figures S11, S12, and S63). A further emphasis was lain on the structural characterization of the thus accessible *s*‐triazines via single‐crystal X‐ray diffraction (SCXRD) for a more complete comprehension of their observed physicochemical properties such as acidity, solubility, density, and absorption behavior (Tables S1–S39) [41].

## 2 Results and Discussion

### 2.1 Starting Material Synthesis

**SCHEME 4:**
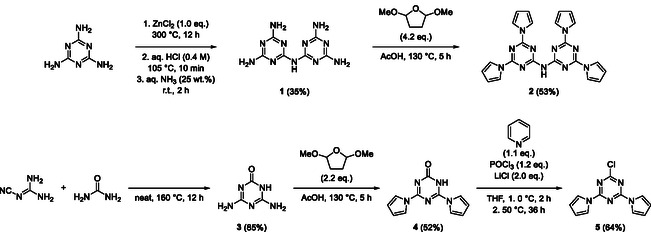
Synthetic pathways toward pyrrolated melam (**2**) (top) and deoxychlorinated pyrrolated ammeline (**5**) (bottom).

Melam (**1**) was prepared by the synthesis of its ZnCl_2_ coordination complex in a muffle furnace, followed by hydrolysis of the same in boiling aqueous HCl (0.4 M) and subsequent neutralization of resulting hydrochloride in aqueous NH_3_ (25 wt.%). Drying at 200 °C finally gave anhydrous **1** (Figure S1). The thus obtained **1** was reacted with 2,5‐dimethoxyTHF in boiling AcOH, yielding pyrrolated melam (**2**). Ammeline (**3**) was synthesized by solid‐state reaction of dicyandiamide and urea in a 1:1 molar ratio. Essentially the same reaction conditions as for **1** could be applied for its pyrrolation. Finally, deoxychlorination of the resulting pyrrolated ammeline (**4**) by conversion with POCl_3_ and pyridine as base led to the synthesis of **5** (Scheme 4).

**FIGURE 1:**
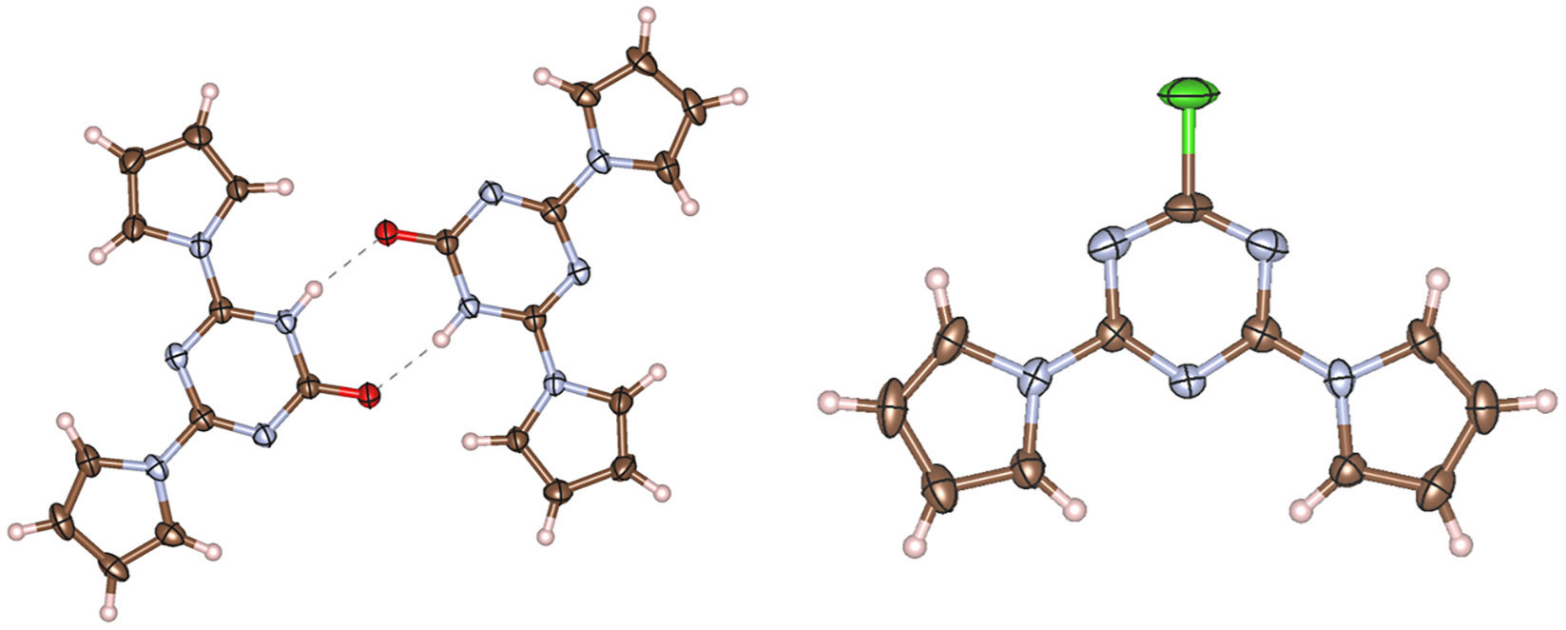
Molecular dimer of **4** (left) and singular molecule of **5** (right) in the crystal. All atoms besides the hydrogen atoms are drawn as thermal ellipsoids at the 50% probability level (atomic coloring: H white, C brown, N light‐blue, O red, Cl green).

**FIGURE 2:**
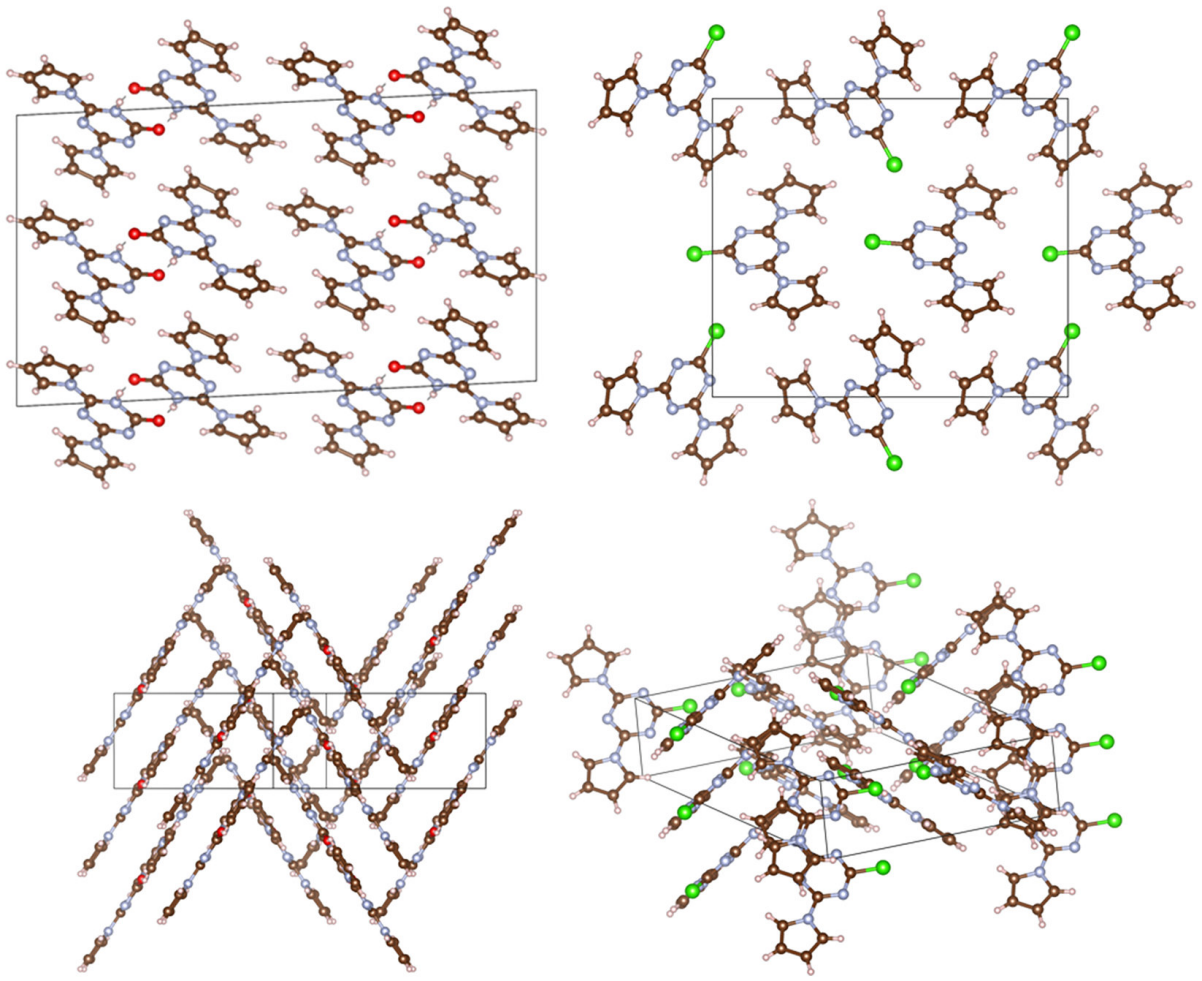
Crystal structure of **4** viewed along [010] (top left) and [403] (bottom left) as well as crystal structure of **5** viewed along [100] (top right) and [64−3] (bottom right) (atomic coloring: H white, C brown, N light‐blue, O red, Cl green).

Analogous to **3**, **4** occurs exclusively as its 2(1*H*)‐keto tautomer in the solid, indicative by the C─O bond length of 1.238(3) Å, which is similar to that of **3** (1.2512(17) Å) [31]. It forms dimers by a pair of hydrogen bonds between NH and CO groups. As in pyrrolated melamine [40], its pyrrolyl substituents are in plane to the *s*‐triazine ring, which is also the case for **5** (Figure 1). As a result, the molecules of **4** and **5** are planar, allowing effective interconnection by parallel staggered π−π stacking and thereby building of columns of molecules with identical orientation along [010] and [100], respectively. Molecules of adjacent columns are tilted against each other in the stacking direction to achieve T‐shaped π−π stacking between the hydrogen atoms and π‐electrons of their pyrrolyl substituents (Figure 2).

**FIGURE 3:**
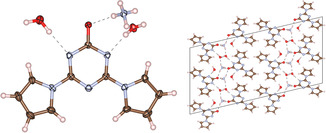
Asymmetric unit of **6·2H**_**2**_ **O** in the crystal with all atoms besides the hydrogen atoms drawn as thermal ellipsoids at the 50% probability level (left) and crystal structure of **6·2H**_**2**_ **O** viewed along [010] (right) (atomic coloring: H white, C brown, N light‐blue, O red).

**SCHEME 5:**
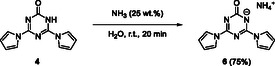
Reaction conditions used for the preparation of **6**.

It was figured that the NH group of **4** should be significantly more acidic than that of **3** due to the lower electron‐donating effect of pyrrolyl groups compared to amino groups. This was demonstrated by dissolution of **4** in aqueous NH_3_ (25 wt.%), followed by solvent evaporation at room temperature, which led to the preparation of its NH_4_^+^ salt (**6**) as a dihydrate (Scheme 5). This behavior stands in contrast with that of **3**, which only reprecipitates in its neutral state from aqueous NH_3_ (25 wt.%) [31]. Next to π−π stacking interactions similar to those found in **4** and **5**, a hydrogen bonding network can be observed in **6·2H**_**2**_ **O**, formed by the solvated H_2_O molecules, NH_4_^+^ cations as well as oxygen and nitrogen atoms of the deprotonated molecules of **4**. The C─O bond length of deprotonated **4** was determined to be slightly longer than in its neutral form (1.268(4) Å) (Figure 3). This observation was also made in the case of **3** [31].

### 2.2 Reactions of Pyrrolated Melam (**2**) With Electrophiles

For the functionalization of its *s*‐triazine bridging amino group, pyrrolated melam (**2**) was reacted with a series of organic halides (R─X) (Table 1).

**TABLE 1:** Reaction conditions used for the functionalization of the *s*‐triazine bridging amino group of **2** by reaction with electrophiles (R─X).

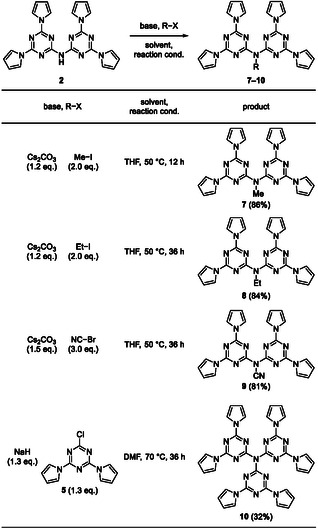

Methylation of **2** by reaction with MeI in THF at 50 °C for 12 h using Cs_2_CO_3_ as base gave **7**. For ethylation and cyanidation of **2** by reaction with EtI and BrCN, the reaction time had to be increased to 36 h for complete conversion, yielding **8** and **9**, respectively. In the case of the reaction of **2** with deoxychlorinated pyrrolated ammeline (**5**), no conversion was observed when conducting the reaction in THF, even when using stronger bases such as KO*t*Bu or NaH. However, when changing the solvent to the more polar DMF, the desired transformation into **10** was achieved.

**FIGURE 4:**
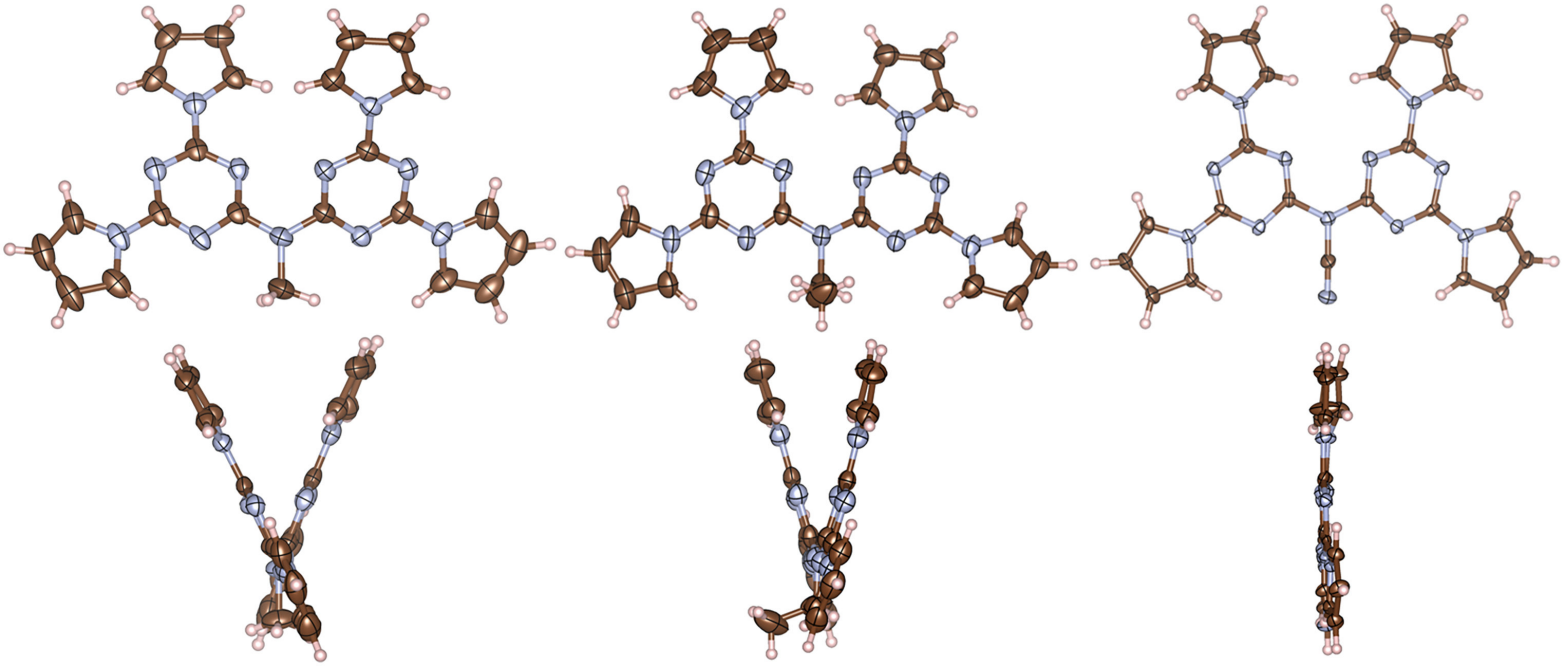
Singular molecule of **7** (left), **8** (middle), and **9** (right) in the crystal viewed roughly perpendicular (top) and parallel (bottom) to the *s*‐triazine planes. All atoms besides the hydrogen atoms are drawn as thermal ellipsoids at the 50% probability level (atomic coloring: H white, C brown, N light‐blue).

**FIGURE 5:**
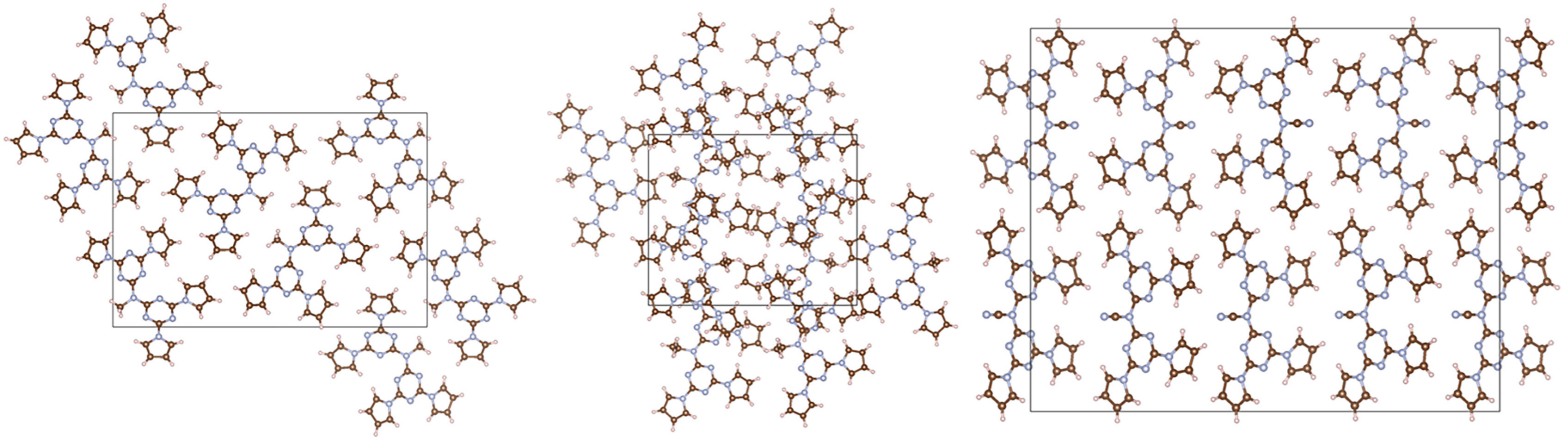
Crystal structure of **7** viewed along [100] (left), crystal structure of **8** viewed along [100] (middle), and crystal structure of **9** viewed along [001] (right) (atomic coloring: H white, C brown, N light‐blue).

In the product series **7**–**9**, a clear trend regarding their solubility in organic solvents such as DCM, THF, and DMF could be observed, with **8** being the most soluble and **9** being the least soluble. An examination of their crystal structures (Figures 4 and 5) provides a possible explanation for this observed trend.

In its crystal structure, the molecules of **9** exhibit almost planar geometry, resulting in very efficient parallel staggered π−π stacking, which, in turn, causes the low solubility of **9**. In contrast, the molecules of **7** und **8** are not close to planar in the solid, as they have pronounced dihedral angles of 48.4(3)° and 34.01(17)°, respectively, between their two *s*‐triazine rings. Nevertheless, the molecules of **7** form π−π stacking columns as **9** despite their nonplanarity, but in contrast to **8**, whose more bulky ethyl substituents circumvent this. In addition to reducing solubility, more effective π−π stacking leads to a higher density. Consequently, **9** (1.487 g/cm^3^) was found to have a higher density than **7** (1.380 g/cm^3^), while the density of **8** (1.347 g/cm^3^) was determined to be comparatively lower.

### 2.3 Reactions of Deoxychlorinated Pyrrolated Ammeline (**5**) With Nucleophiles

Due to the electron‐poor nature of the *s*‐triazine ring, it was figured that the Cl substituent of deoxychlorinated pyrrolated ammeline (**5**) should be readily displaceable by reaction with nucleophiles (Table 2).

**TABLE 2:** Reaction conditions used for the displacement of the Cl substituent of **5** with nucleophiles (R─H or Cat.^+^R^−^).

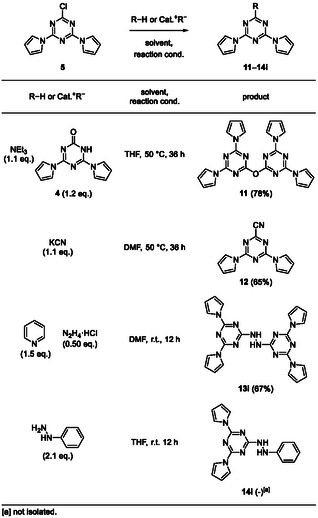

**FIGURE 6:**
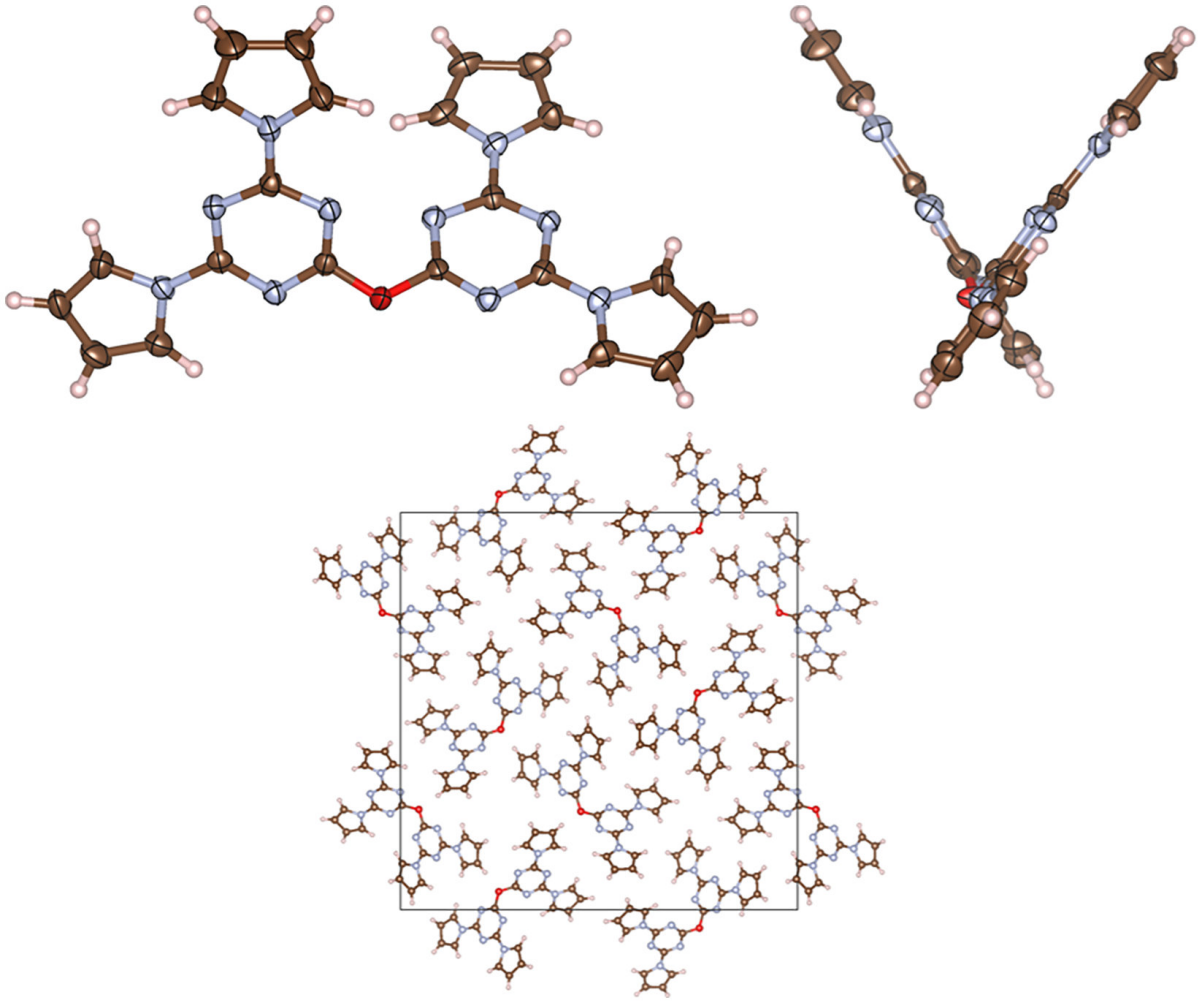
Singular molecule of **11** in the crystal viewed roughly perpendicular (top left) and parallel (top right) to the *s*‐triazine planes with all atoms besides the hydrogen atoms drawn as thermal ellipsoids at the 50% probability level as well as crystal structure of **11** viewed along [001] (bottom) (atomic coloring: H white, C brown, N light‐blue, O red).

First, the reaction of **5** with pyrrolated ammeline (**4**) under use of NEt_3_ as base was investigated, affording **11**. This compound is chemically closely related to pyrrolated melam (**2**), with the only difference being that the *s*‐triazine bridging NH functionality is replaced by O. Analogous to the methylated derivative of pyrrolated melam (**7**), the molecules of **11** arrange in the solid into columns connected by parallel staggered π−π stacking. Furthermore, the density of **11** (1.384 g/cm^3^) is very similar to that of **7**. However, the determined dihedral angle between its two *s*‐triazine rings is 69.6(4)° and thus substantially larger than that of **7**, suggesting that the oxygen’s second lone pair is sterically more demanding than the methyl group of **7**. The C─O─C angle was found to be 121.2(3)°, which is similar to those reported for diphenylether (118.27(10)° and 117.88(12)°) [42] and indicates *sp* ^2^‐hybrdization of the oxygen atom (Figure 6).

**FIGURE 7:**
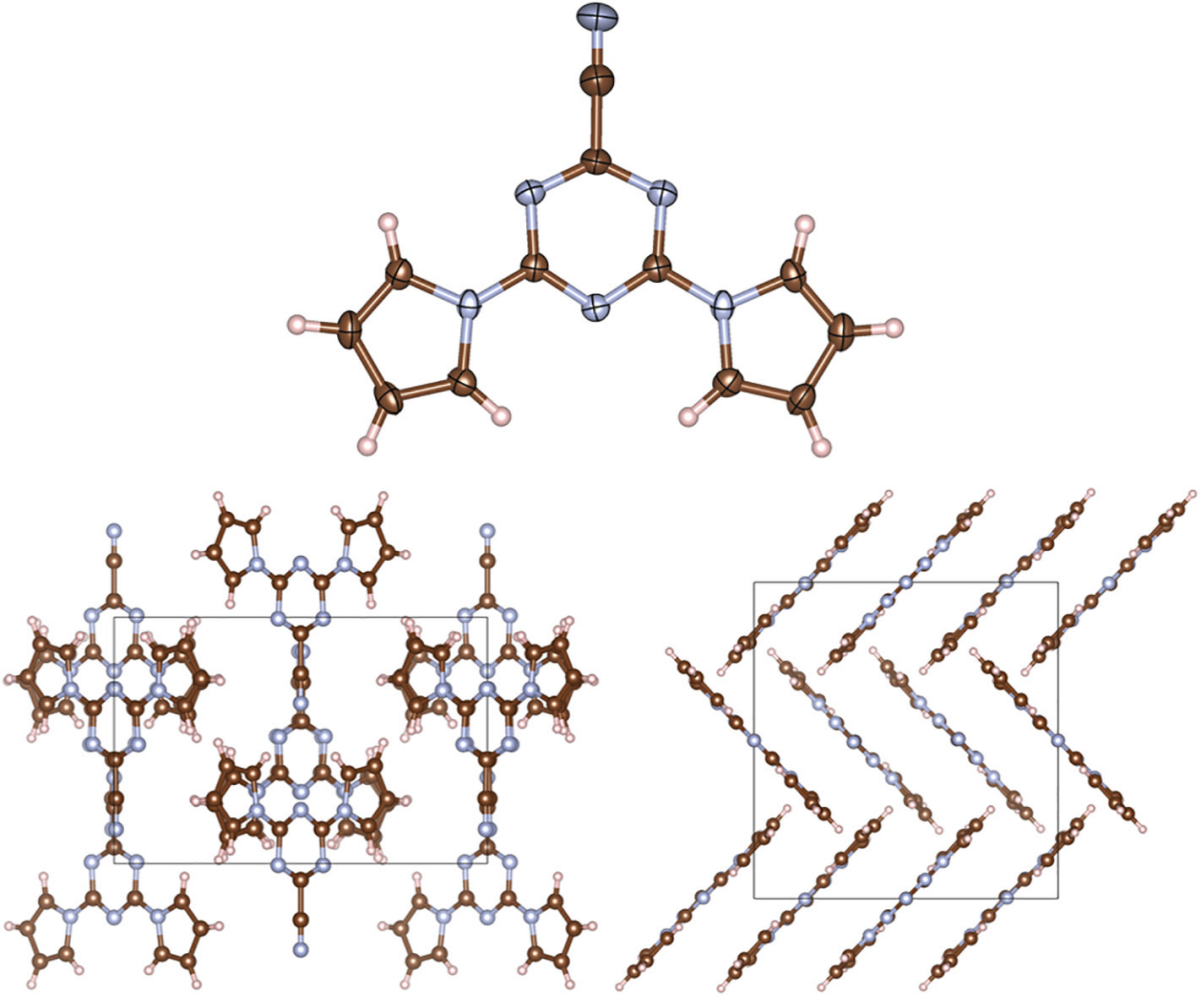
Singular molecule of **12** in the crystal with all atoms besides the hydrogen atoms drawn as thermal ellipsoids at the 50% probability level (top) as well as crystal structure of **12** viewed along [100] (bottom left) and [001] (bottom right) (atomic coloring: H white, C brown, N light‐blue).

**SCHEME 6:**
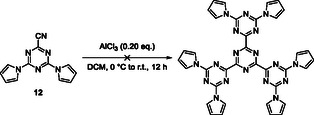
Reaction conditions used for the attempted trimerization of **12**.

Substitution of Cl with CN was achieved by reaction of **5** with KCN, which gave **12**. Analogous to **5**, the molecules of **12** show planar geometry in the solid and are interconnected by parallel staggered and T‐shaped π−π stacking. In contrast to **5**, however, **12** does not form columns with identical molecular orientations, as every third row along [100] has an opposite orientation (Figure 7). An attempt was made to trimerize **12** through conversion with the Lewis acid AlCl_3_. However, this yielded only poorly defined polymeric products (Scheme 6).

**SCHEME 7:**
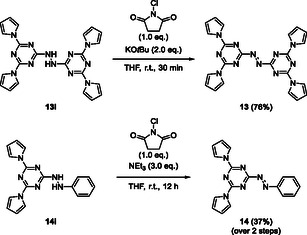
Reaction conditions used for the oxidation of the hydrazino functionality of **13i** (top) and **14i** (bottom).

The reaction of N_2_H_4_·HCl with two equivalents of **5** and pyridine as base led to the functionalization of both of its nitrogen atoms, thereby giving the symmetrically 1,2‐disubstitued hydrazine derivative **13i**. Reacting **13i** with *N*‐chlorosuccinimide resulted in the oxidation to the respective azo/diazene derivative **13**. Conversion of **5** with phenylhydrazine yielded **14i**, which, in contrast to **13i**, was not isolated and instead directly oxidized to **14**, using similar reaction conditions as for **13i** (Scheme 7).

**FIGURE 8:**
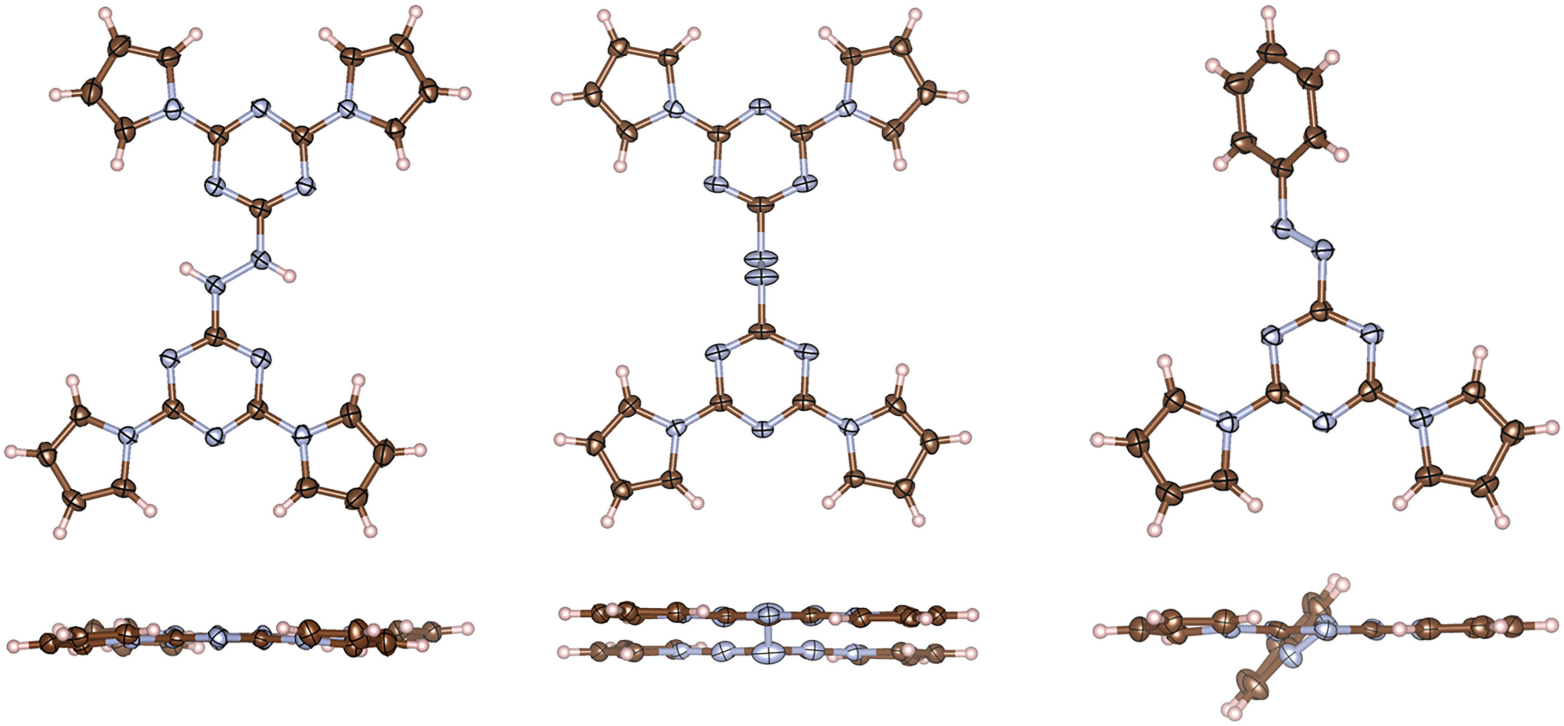
Singular molecule of **13i** (left), **13** (middle), and **14** (right) in the crystal viewed perpendicular (top) and parallel (bottom) to the *s*‐triazine planes. All atoms besides the hydrogen atoms are drawn as thermal ellipsoids at the 50% probability level (atomic coloring: H white, C brown, N light‐blue).

**FIGURE 9:**
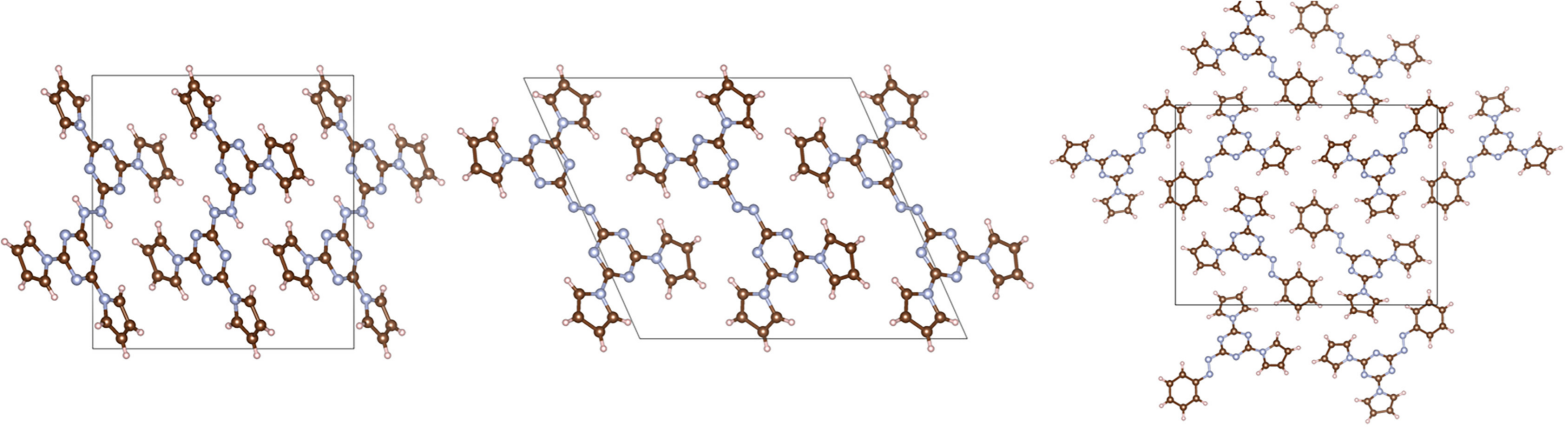
Crystal structure of **13i** (left) viewed along [010] (left), crystal structure of **13** viewed along [010] (middle), and crystal structure of **14** viewed along [100] (right) (atomic coloring: H white, C brown, N light‐blue).

In the crystal, the molecules of **13i** exhibit an essentially planar geometry, which is most likely due to weak intramolecular hydrogen bonding between the hydrazine hydrogen atoms and the lone pairs of *s*‐triazine nitrogen atoms (Figure 8). This contrasts with 1,2‐diphenylhydrazine, which exhibits a dihedral angle of around 55° between its two phenyl rings to reduce the electrostatic repulsion between its hydrazine and phenyl hydrogen atoms [43]. Nevertheless, the N─N bond length of **13i** (1.385(4) Å) was found to be similar to that of 1,2‐diphenylhydrazine (1.394(8) Å) [43]. In the case of **13**, the *s*‐triazinyl substituents are rotated about 90° out of the CNNC plane to avoid steric repulsion between the lone pairs of the hydrazine and *s*‐triazine nitrogen atoms. Nevertheless, the two *s*‐triazine rings are parallel to each other. As this steric repulsion is absent in azobenzene, its phenyl rings are instead roughly parallel to the CNNC plane [44]. The N─N bond length of **13** (1.162(4) Å) was found to be significantly shorter than that of azobenzene (1.2405(3) and 1.24716(16) Å) [44]. This can be attributed to the out‐of‐plane rotation of the *s*‐triazinyl substituents, which prevents the π‐orbitals of the diazene nitrogen atoms from interacting with the π‐systems of the *s*‐triazinyl substituents. Based on the aforementioned observations, a dihedral angle of around 90° between the phenyl and *s*‐triazinyl substituents may be expected in the case of **14**. However, this dihedral angle was determined to be only around 41°. While its phenyl substituent is roughly parallel to the CNNC plane as in azobenzene, its *s*‐triazinyl substituent is considerably less rotated out‐of‐plane compared to those of **13**. The N─N bond length of **14** (1.249(3) Å) was found to be very similar to that of azobenzene. Despite their distinct molecular geometries, all three compounds form parallel staggered π−π stacking columns in the crystal (Figure 9). These findings demonstrate that replacing phenyl with dipyrrolyl‐*s*‐triazinyl substituents alters significantly an azo compounds’ molecular geometry. Therefore, UV/Vis spectroscopy was conducted with **13**, **14** and commercially purchased azobenzene to study how this also affects an azo compound’s absorption behavior (Figures S59–S61). A strong blueshift of the absorption compared to azobenzene can be observed for **13**, which can be explained similarly to the shortened N─N bond length by the absence of any interactions between the π‐electrons of the diazene nitrogen atoms and those of the *s*‐triazine rings. This observation is also consistent with the yellow color of **13** in comparison with the orange color of azobenzene. In contrast, **14** shows a slight redshift of its absorption compared to azobenzene, which fits to its more pronounced orange coloration compared to azobenzene (Figure S64).

### 2.4 Reversion of the Pyrrolyl Groups Into Amino Groups

In several previously published works, pyrrolyl groups have been utilized as protecting groups for amino groups. However, compared to more common amine protecting groups such as *tert*‐butyloxycarbonyl (Boc) or fluorenylmethoxycarbonyl (Fmoc), more sophisticated reaction conditions are required to revert pyrrolyl groups into amino groups. A literature‐known deprotection protocol for pyrrolyl groups involves ozonolysis with reductive work‐up and subsequent acidic hydrolysis of the resulting formamide derivatives [45, 50]. However, the use of O_3_ is incompatible with many compounds, as it also reacts with other electron‐rich aromatic rings and oxidizable functionalities such as alkenyl or aldehyde groups [51, 54]. Due to the absence of such sensitive groups in the pyrrolated melam derivatives **7**–**10** and the closely related **11**, ozonolysis was considered feasible in their cases. For this reason, a reaction protocol involving ozonolysis was developed for the reversion of their pyrrolyl groups into amino groups, affording the products **15**–**19** (Table 3).

**TABLE 3:** Reaction conditions used for the reversion of the pyrrolyl groups into amino groups of compounds **7**–**11**.

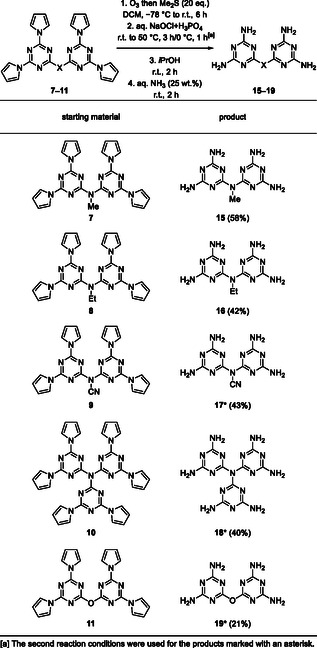

**SCHEME 8:**
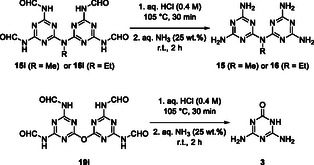
Results of the acidic hydrolysis of the formamide intermediates **15i**, **16i**, and **19i**.

Ozonolysis of **7**–**11** with reductive work‐up using Me_2_S successfully led to the respective formamide derivatives **15i**–**19i** as intermediates, which was verified by FTIR and NMR spectroscopy. In the case of the ozonolysis product of **9**, **17i**, a strong signal at around 1710 cm^−1^ became visible in the FTIR spectrum, resulting from C─O double bond stretching of the formyl groups (Figure S55). In its ^1^H‐NMR spectrum, two doublets at 9.42 and 11.60 ppm were found, corresponding to the hydrogen atoms bound to the formamide nitrogen and carbon atoms, respectively (Figure S46). Their coupling constant was determined to be 9.2 Hz, showing that the formamide groups predominantly exist in (*Z*)‐conformation [55]. Additionally, a small singlet at 8.13 Hz was detected, corresponding to HCOOH [56], which was generated from the reaction of trace H_2_O dissolved in the DCM with the compound’s diformimide groups (i.e., R─N(CHO)_2_), initially formed by the ozonolysis. The HCOOH signal was better visible in the case of the ozonolysis product of **7**, **15i** (Figure S40). Subsequently, the transformation of the formamide intermediates into the respective amines by acidic hydrolysis was investigated. For the formamide intermediates **15i** and **16i**, this could be achieved by stirring in aqueous HCl (0.4 M) for 30 min at 105 °C. In the case of the formamide intermediates **17i**–**19i**, however, these harsh reaction conditions also resulted in the nucleophilic attack of H_2_O at the carbon atoms bound to the *s*‐triazine bridging NR^1^R^2^/OR substituent. This means that in the case of the formamide intermediate **19i**, for example, only ammeline (**3**) was obtained as the reaction product instead of the desired **19** (Scheme 8).

Using lower reaction temperatures gave the same result, while the use of weaker acids such as aqueous AcOH also led to this cleavage of the molecule without even converting the formamide groups into amino groups. Another commonly employed deprotection option for formamides is the hydrolysis under alkaline conditions [57, 60]. However, regardless of the base used (e.g., NaOH, NH_3_, or Na_2_HPO_4_), only poorly defined polymeric products were obtained.

**SCHEME 9:**
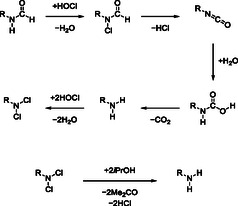
Proposed reaction mechanism for the transformation of a formamide group into an amino group by conversion with acidified aqueous NaOCl (top), followed by work‐up with *i*PrOH (bottom).

Since neither basic nor acidic hydrolysis showed to be applicable, a second oxidative deprotection step was considered. However, the reaction with aqueous H_2_O_2_ (30 wt.%) led only to some form of polymerization, while the conversion with di‐*tert*‐butyl peroxide in chlorobenzene [61] resulted in no reaction at all. In contrast, the reaction with aqueous NaOCl (13% active chlorine) acidified with a small amount of H_3_PO_4_ proved to be promising. Gas formation was observed during these reactions, which was confirmed as CO_2_ by clouding of an aqueous Ba(OH)_2_ solution placed above the reaction vessel (Figure S65). This points to the oxidation of the formamide groups to carbamic acid groups, which subsequently decomposed into amino groups under release of CO_2_. This transformation presumably proceeded under formation of isocyanate groups as intermediates, which is supported by the fact that *N*‐halo‐formamides are known to convert into isocyanates upon heating in the presence of a base [62, 64]. Therefore, the reaction’s mechanism is most likely closely related to that of the Hofmann degradation/rearrangement [65, 66], in which primary amines are generated from primary amides (i.e., R─C(O)─NH_2_), which are constitutional isomers of the respective secondary formamides (i.e., R─N(H)─CHO). However, since the employed reaction conditions are similar to those reported for the hexachlorination of melamine [67], the desired amino groups initially reacted further into dichloramino groups. This was confirmed FTIR spectroscopy, where the absence of any NH stretching signals in the wavenumber range of 3600–3000 cm^−1^ was observed after treatment with acidified aqueous NaOCl. Simultaneously, all signals originating from C─O double bond stretching disappeared as well (Figure S56). The dichloramino groups were reverted to amino groups through work‐up with *i*PrOH. This most likely led to the oxidation of *i*PrOH into Me_2_CO under simultaneous formation of HCl, which was supported by the fact that the solution became strongly acidic (Scheme 9).

As a result, the products were initially obtained as hydrochlorides, which, however, could easily be converted to their neutral form by stirring in aqueous NH_3_ (25 wt.%). In contrast to acidic hydrolysis, this deprotection protocol proved to be applicable to all target compounds and further resulted in higher purity in the case of **15** and **16**, as the former yielded heavily discolored products. It was further investigated whether oxidation of pyrrolyl groups is possible directly by acidified aqueous NaOCl, thereby allowing the first oxidation step by ozonolysis to be skipped. However, no reaction was observed between **7**–**11** and acidified aqueous NaOCl.

**15**–**19** should be able to form coordination complexes with transition metal salts analogous to melam (**1**) and were found to have improved solubility in solvents like DMF and DSMO compared to **1**, with **16** in particular exhibiting high solubility in hot H_2_O as well. Therefore, the synthesis of coordination complexes from solution, contrary to **1** itself, may be feasible for those compounds. Additionally, **18** possesses three coordination sites structurally similar to that of **1**, which means it could potentially be utilized as a bridging ligand for coordination polymers similar to 1,4,5,8,9,12‐hexaazatriphenylene (HAT) [68, 72]. Another intriguing trait of **18** alongside **17** is that both contain only C─N and N─H bonds, meaning that they can be described as molecular mixtures of carbon(IV) nitride C_3_N_4_ and NH_3_. Their resulting formal NH_3_ contents per formula C_3_N_4_ lie between those of melam (**1**) and melem (Table 4). On the other hand, **19** can be considered the formal anhydride of ammeline (**3**).

**TABLE 4:** Sum formula and NH_3_ content per formula C_3_N_4_ of **17** and **18** in comparison to dicyandiamide, melamine, melam (**1**), and melem.

Compound	Sum formula	NH_3_ content per formula C_3_N_4_
Dicyandiamide	C_2_H_4_N_4_	C_3_N_4_·2NH_3_
Melamine	C_3_H_6_N_6_	C_3_N_4_·2NH_3_
Melam (**1**)	C_6_H_9_N_11_	C_3_N_4_·^3^/_2_NH_3_
**18**	C_9_H_12_N_16_	C_3_N_4_·^4^/_3_NH_3_
**17**	C_7_H_8_N_12_	C_3_N_4_·^8^/_7_NH_3_
Melem	C_6_H_6_N_10_	C_3_N_4_·NH_3_

**FIGURE 10:**
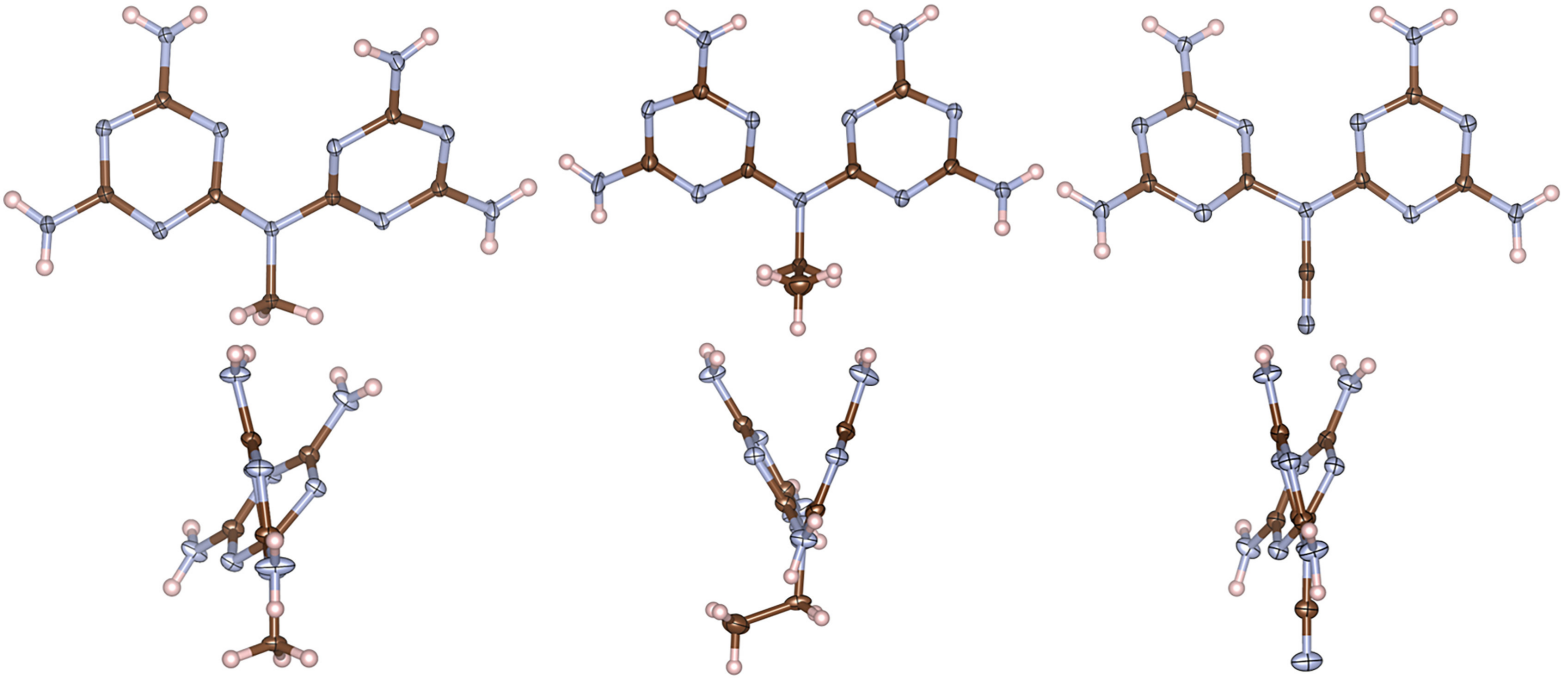
Singular molecule of **15** (left), **16** (middle), and **17** (right) in the crystal viewed roughly perpendicular (top) and parallel (bottom) to the *s*‐triazine planes. All atoms besides the hydrogen atoms are drawn as thermal ellipsoids at the 50% probability level (atomic coloring: H white, C brown, N light‐blue).

**15**, **16**, and **17** were obtained as monohydrate, sesquihydrate, and anhydrate, respectively. In these structures, the molecules of **15**–**17** have nonplanar geometries due to pronounced dihedral angles between their *s*‐triazine rings (49.95(12)° in **15·H**_**2**_ **O**; 50.7(2)° and 57.9(3)° in **16·1.5H**_**2**_ **O**; 44.28(16)° in **17**). These angles are significantly wider than those found for **1** in its anhydrous form (19.49(14)° and 24.65(10)°) [17] and its dihydrate (5.71(14)°) [33]. In the pyrrolyl derivatives **7** and **8**, the dihedral angles arise through essentially equal out‐of‐plane rotations of the *s*‐triazine rings in opposite directions. While this is also the case in **16·1.5H**_**2**_ **O**, this does not apply to **15·H**_**2**_ **O** and **17**, where one of the *s*‐triazine rings is close to parallel to the central C─N(R)─C plane, whereas the other *s*‐triazine ring is rotated much further out of plane for compensation (Figure 10).

**FIGURE 11:**
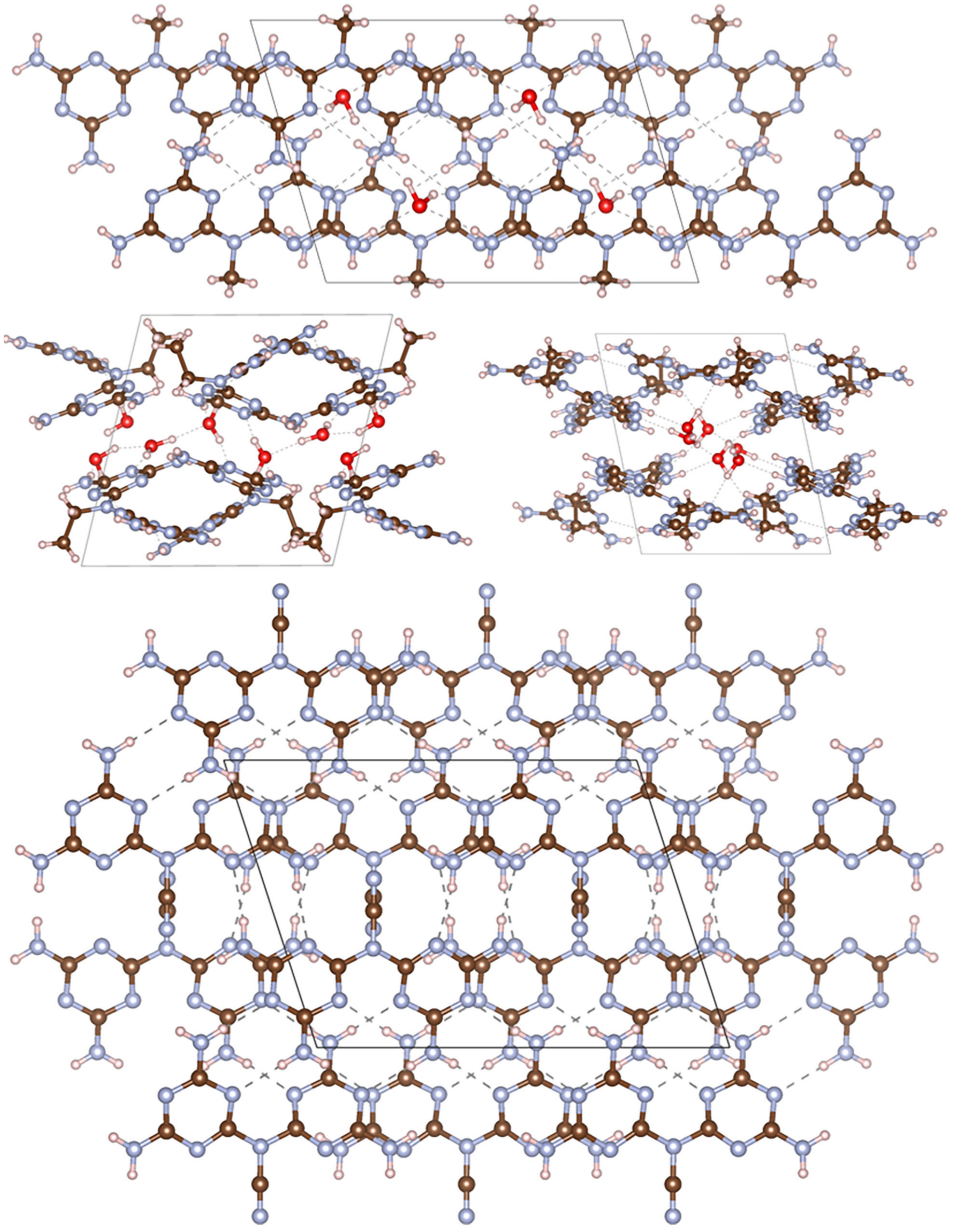
Crystal structure of **15·H**_**2**_ **O** viewed along [010] (top), crystal structure of **16·1.5H**_**2**_ **O** viewed along [100] (middle left) and [001] (middle right) as well as crystal structure of **17** viewed along [010] (bottom) (atomic coloring: H white, C brown, N light‐blue, O red).

As in **1**, **15·H**_**2**_ **O**, **16·1.5H**_**2**_ **O** and **17** are predominantly held together through hydrogen bonding and only weak π−π stacking is observed, which contrasts with **1·2H**_**2**_ **O** that consists of layers interconnected through parallel staggered π−π stacking. In **15·H**_**2**_ **O**, the H_2_O molecules occur isolated from another in pseudo‐tetrahedral coordination spheres. They are held in place through hydrogen bonding to nearby molecules of **15** by both their lone pairs and hydrogen atoms, which interact with the hydrogen atoms of amino groups and π‐orbitals of nitrogen atoms, respectively. In contrast, the H_2_O molecules in **16·1.5H**_**2**_ **O** form hydrogen bonds predominantly among themselves, thereby building up H_2_O channels along [001] within the structure (Figure 11).

**FIGURE 12:**
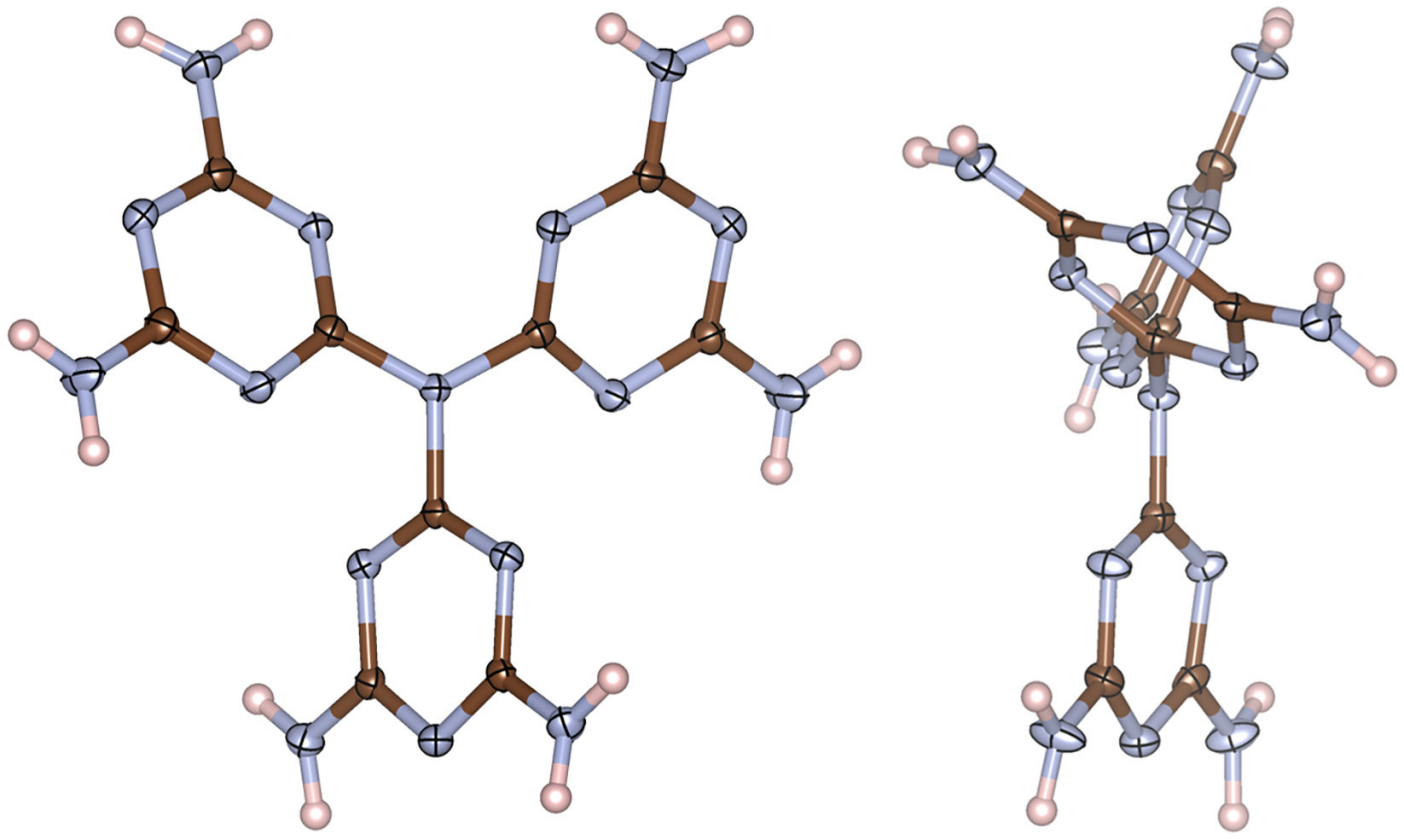
Singular molecule of **18** in the crystal viewed roughly perpendicular (left) and parallel (right) to the *s*‐triazine planes. All atoms besides the hydrogen atoms are drawn as thermal ellipsoids at the 50% probability level (atomic coloring: H white, C brown, N light‐blue).

**18** was found to form a dihydrate from aqueous solution. In **18·2H**_**2**_ **O**, the molecules of **18** exhibit a paddlewheel‐like geometry, as all three *s*‐triazine rings are rotated outward in the same direction from the central C_3_N plane. The same geometry was found for the closely related tris(4,6‐dichloro‐*s*‐triazin‐2‐yl)amine [73], which has Cl substituents in place of the NH_2_ substituents. In **18**, however, the *s*‐triazine rings do not exhibit the same degree of out‐of‐plane rotation, as it is roughly twice as large for one *s*‐triazine ring (59.79(10)°) than for the other two *s*‐triazine rings (27.24(11)°) (Figure 12).

**FIGURE 13:**
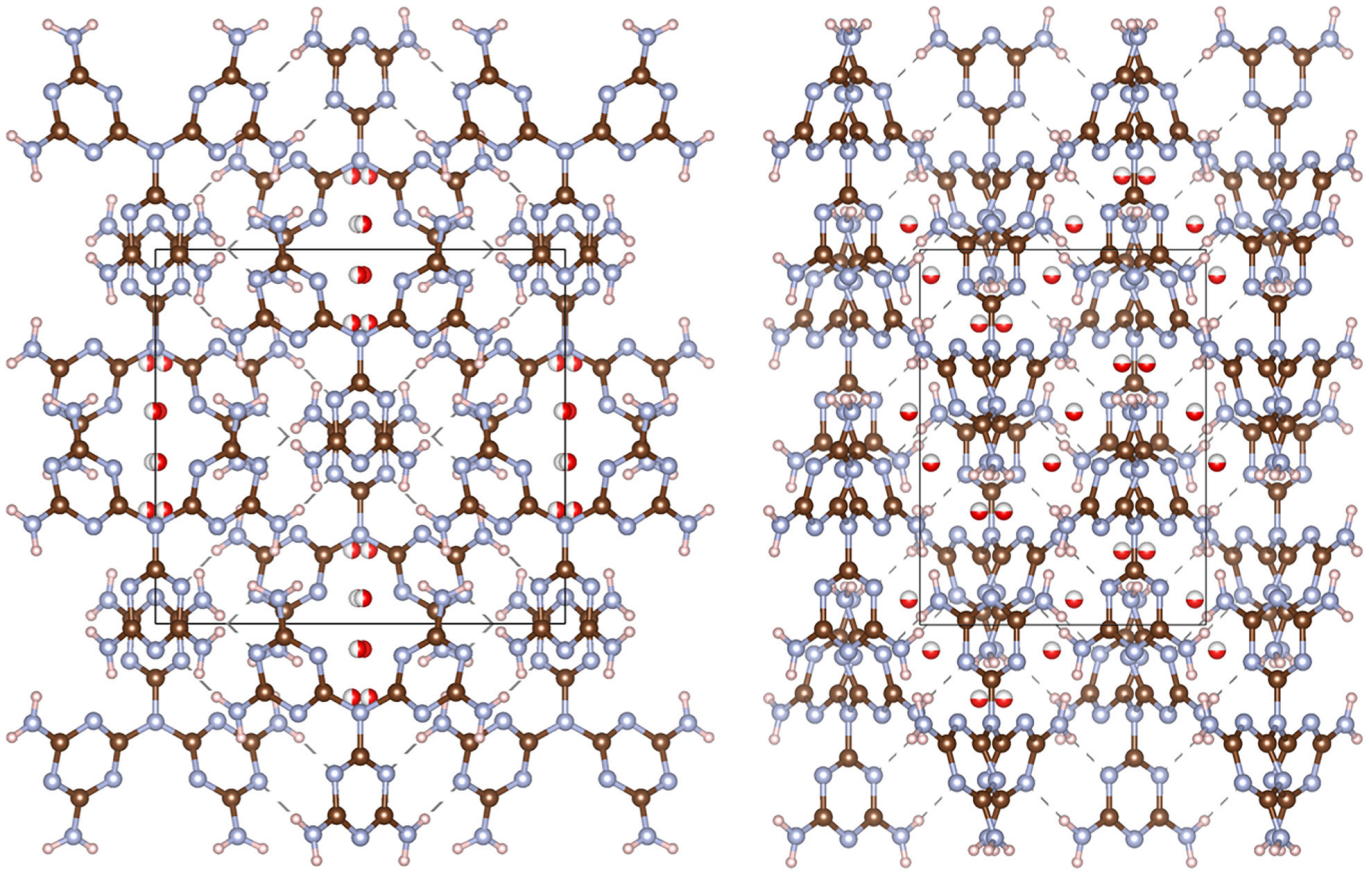
Crystal structure of **18·2H**_**2**_ **O** viewed along [001] (left) and [100] (right) (atomic coloring: H white, C brown, N light‐blue, O red).

The molecules of **18** interconnect solely via hydrogen bond pairs between their amino group hydrogen atoms and their *s*‐triazine nitrogen atom lone pairs. The H_2_O molecules are located in S‐shaped channels between the molecules of **18**. In contrast to **16·1.5H**_**2**_ **O**, the H_2_O molecules exhibit a disorder and every oxygen position is only half occupied (Figure 13).

**FIGURE 14:**
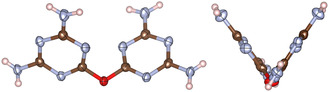
Singular molecule of **19** in the crystal viewed roughly perpendicular (left) and parallel (right) to the *s*‐triazine planes. All atoms besides the hydrogen atoms are drawn as thermal ellipsoids at the 50% probability level (atomic coloring: H white, C brown, N light‐blue, O red).

Analogous to **17**, **19** crystallized as an anhydrate from aqueous solution. In its crystal structure, the molecules exhibit a dihedral angle of 78.7(3)° between both *s*‐triazine rings and thus a significantly wider angle than those of **15**–**17**, analogous to their pyrrolyl counterparts. As in **11**, the oxygen atoms seem to be *sp* ^2^‐hybridized, indicative by the C─O─C angle of 116.8(2)° (Figure 14).

**FIGURE 15:**
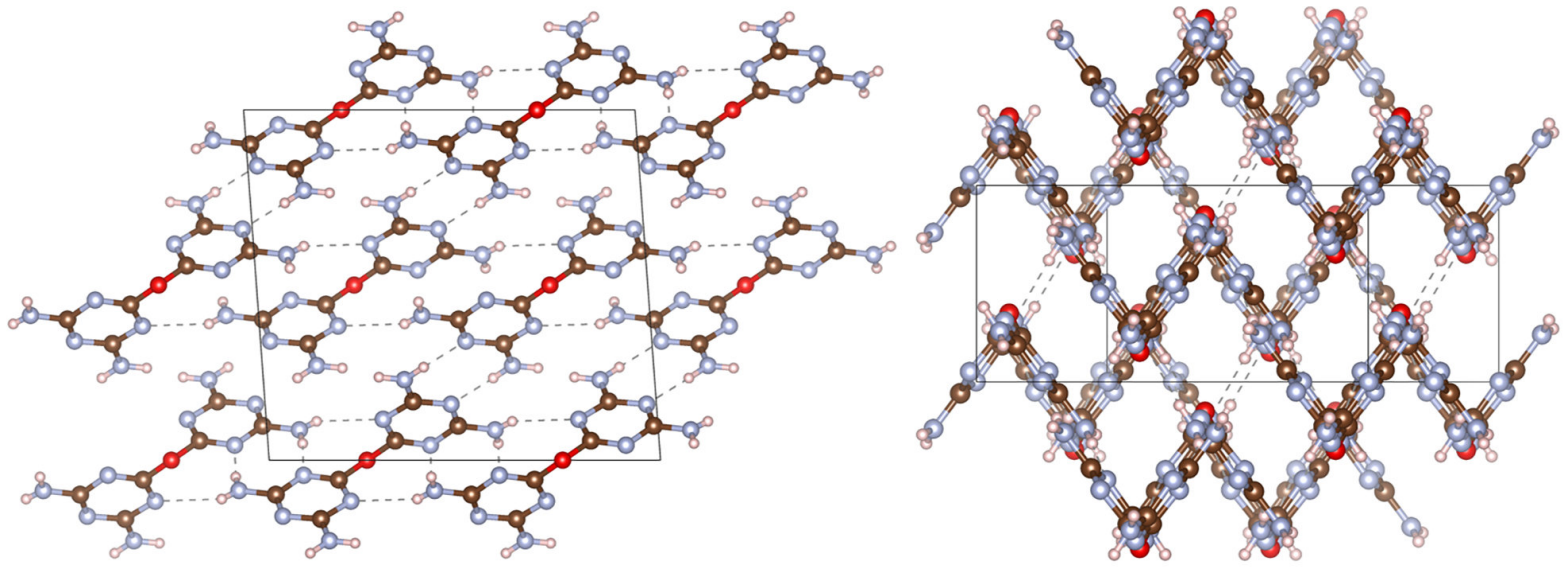
Crystal structure of **19** viewed along [010] (left) and [103] (right) (atomic coloring: H white, C brown, N light‐blue, O red).

As in the structure of **1** and **17**, the molecules of **19** form a hydrogen bonding network. In contrast to **1** and **17**, however, the presence of parallel staggered π−π stacking between the molecules can additionally be found in **19** (Figure 15). Nevertheless, the density at 20 °C of **19** (1.651 g/cm^3^) is lower than of that of **1** (1.666 g/cm^3^), just like that of **17** (1.648 g/cm^3^) (Table S40). A potential reason for this may be their greater deviation from planar molecular geometry compared to **1**.

Due to significantly improved aqueous solubility of **16** compared to **1**, it was figured that coordination complexes of **16** should be accessible from aqueous solution. Cu(II) was chosen as the metal center due to its high for affinity for *N*‐donor ligands and the fact that Cu(II) coordination complexes of **1** have not yet been synthesized. Moreover, Cu(II) coordination complexes with other bidentate *N*‐donor ligands such as 2,2′‐‍bipyridine (bipy) or 1,10‐phenanthroline (phen) have been demonstrated to function as effective homogenous catalysts in various types of reactions [74, 79]. ClO_4_^−^ was selected as the counter anion because it is weakly coordinating and insensitive to hydrolysis.

**SCHEME 10:**
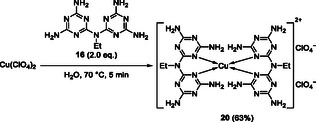
Reaction conditions used for the preparation of **20**.

In order to obtain the desired Cu(II) coordination complex of **16**, an aqueous solution of Cu(ClO_4_)_2_ was added to an aqueous solution of **16** at 70 °C. A reaction between both compounds became directly apparent as the resulting solution became dark‐blue. This contrasts sharply with the analogous attempted reaction between **1** and Cu(ClO_4_)_2_ in H_2_O, where no substantial color change was observed even when the temperature was raised until the reaction mixture boiled. Upon cooling to room temperature, the target compound **20** precipitated as a trihydrate that turned out to be brown in color rather than the expected dark‐blue (Figure S66). This color is consistent with the observation that **20** exhibits absorption across nearly the entire wavelength range used in UV/Vis spectroscopy measurements (Figure S62). Additionally, in contrast to previously reported coordination complexes of **1**, not one but two molecules of **16** are coordinating each metal center (Scheme 10).

**FIGURE 16:**
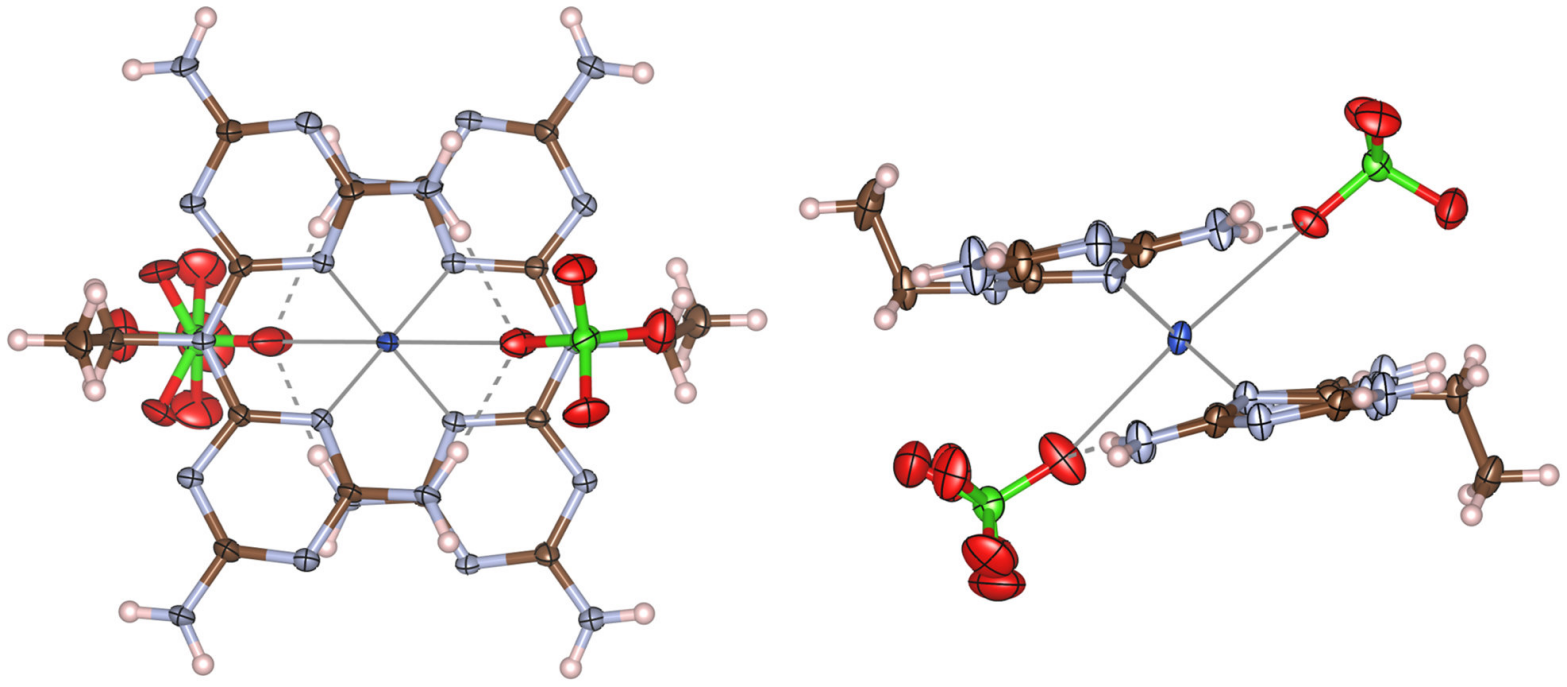
Asymmetric unit of **20·3H**_**2**_ **O** excluding the H_2_O molecules in the crystal viewed perpendicular (left) and parallel (right) to the *s*‐triazine planes. All atoms besides the hydrogen atoms are drawn as thermal ellipsoids at the 50% probability level (atomic coloring: H white, C brown, N light‐blue, O red, Cl green, Cu dark‐blue).

In the crystal structure of **20·3H**_**2**_ **O**, two molecules of **16** bidentately coordinate each Cu(II) cation in an essentially square planar fashion via lone pairs of *s*‐triazine nitrogen atoms (Cu─N: 1.97–2.00 Å). Additionally, one oxygen atom of every ClO_4_^−^ anion forms a weak coordination bond to a single Cu(II) cation (Cu─O: 2.786(6) and 2.881(5) Å), overall resulting in a significantly Jahn–Teller distorted, pseudo‐octahedral coordination for the Cu(II) cations (Figure 16). Similar to **1** in its coordination complexes, the coordination of Cu(II) results in a more planar geometry for the molecules of **16**, indicative by the small dihedral angles between their *s*‐triazine rings (0.5(5)° and 3.3(5)°). To reduce steric repulsion between their amino groups, the two molecules of **16** of one complex are not coplanar to each other. The coordinated Cu(II) cation is also not located in either of the two molecular planes, as is the case for metal cations in previously reported coordination complexes of **1**, but is instead positioned roughly in between both molecular planes. Due to this coordination, the *s*‐triazine rings of **16** distort into an unusual, nonperfectly planar geometry to still enable their nitrogen atoms’ lone pairs to coordinate the Cu(II) cation. As a further consequence, the C─N(Et)─C angles of 125.2(4)° and 126.0(4)° are significantly narrower than the C─N(H)─C angles found for coordination complexes of **1** (132°–134°) [17, 19]. In contrast, the found Cu─N coordination bond lengths are similar to those reported for Cu(I) coordination complexes of **1** (1.96–2.03 Å) [19].

**FIGURE 17:**
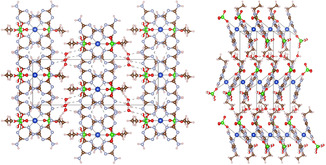
Crystal structure of **20·3H**_**2**_ **O** viewed along [100] (left) and [102] (right) (atomic coloring: H white, C brown, N light‐blue, O red, Cl green, Cu dark‐blue).

The molecules of **16** form rows along [102] via hydrogen bonding between their hydrogen atoms of the amino groups and lone pair of the *s*‐triazine nitrogen atoms. The rows of **16** are interconnected by the Cu─N coordination bonds as well as π−π stacking interactions between amino nitrogen atoms and *s*‐triazine carbon atoms, thereby building up stacks along [001]. In similar manner as in **16·1.5H**_**2**_ **O**, the H_2_O molecules fill up the resulting channels between these stacks, forming hydrogen bonds between themselves as well as with nearby molecules of **16** and ClO_4_^−^ anions. Further hydrogen bonds are formed between the oxygen atoms of the ClO_4_^−^ anions and other amino group hydrogen atoms. One of the two ClO_4_^−^ anions exhibits a disorder (Figure 17).

## 3 Conclusion

Within the scope of this work, first approaches for tapping the latent potential of melam (**1**) and ammeline (**3**) as starting materials for the preparation of asymmetrically substituted *s*‐triazines were developed, almost two centuries after their first description through Liebig. Their near insolubility in all solvents, previously restricting their use to simple acid–base and solid‐state reactions, was resolved through pyrrolation of their primary amino groups by reaction with 2,5‐dimethoxyTHF in boiling AcOH, which conveniently preserved their remaining functional groups for further transformations in organic solvents.

Pyrrolated ammeline (**4**) was deoxychlorinated using POCl_3_, which allowed the displacement of the resulting Cl substituent with nucleophiles, which among others gave access to *s*‐triazine‐based azo compounds. Solid‐state structure analysis revealed that the introduction of *s*‐triazinyl substituents results in the departure of the essentially planar geometry usually found for azo compounds, resulting from electrostatic repulsion between the lone pairs of *s*‐triazine and diazene nitrogen atoms. UV/Vis spectroscopy demonstrated that these structural changes can be utilized to fine‐tune an azo compound’s absorption behavior. In particular, replacing one or two phenyl substituents with dipyrrolyl‐*s*‐triazinyl substituents results in a red‐ or blueshift, respectively. As azo compounds are examined for applications such as dyes and photoswitches [80, 83], these insights may prove valuable in the future development of functional *s*‐triazine‐based azo compounds.

In the case of pyrrolated melam (**2**), the *s*‐triazine bridging amino group was functionalized through reactions with electrophiles. The pyrrolyl groups of the resulting products were reverted into amino groups through ozonolysis, followed by reaction of the resulting formamide intermediates with acidified aqueous NaOCl and subsequent work‐up in *i*PrOH. All thus obtained derivatives of **1** turned out to have better solubility than their parent compound, for which their solved crystal structures provided evidence that this may be due to their less planar geometry, resulting from the greater rotation out of the central C─N(R)─C plane of their *s*‐triazinyl substituents compared to those of unsubstituted **1**. This enables solution‐based coordination chemistry inaccessible to **1** itself, as exemplary demonstrated by the reaction between the ethylated derivative **16** and Cu(ClO_4_)_2_ in aqueous solution, yielding a coordination complex with a fundamentally different coordination geometry compared to all previously reported coordination complexes of **1**, namely, two molecules of **16** bidentately coordinate one Cu(II) cation at the same time. In addition, the improved solubility of the resulting coordination complexes themselves could even open up their use for homogenous catalysis, especially since catalytically potent but reduction‐prone metal centers similar to Cu(II) now also represent a potentially viable option. Examples thereof would be Pd(II) and Pt(II) [84, 87], which just get converted into their elemental form under typical reaction conditions used for the synthesis of coordination complexes of **1** (Figures S12 and S13).

Overall, this study provides a promising and versatile synthetic platform for the important *s*‐triazine compound class through the development of various synthetic strategies for the transformation of the functional groups of **1** and **3**.

## Conflicts of Interest

The authors declare no conflicts of interest.

## Supporting information

The authors have cited additional references within the Supporting Information [88, 100].

## Data Availability

The data that support the findings of this study are available in the supplementary material of this article.
