# Supplementary material for: Advantages of Asymmetry: A Synthetic and Structural Exploration of s‐Triazine Chemistry Starting From Pyrrolated Ammeline and Melam
Source: ChemistryOpen. 2026 Jul 13;15(7):e70252. doi: 10.1002/open.70252 (PMC13359248; doi:10.1002/open.70252)
Supplement: Supplementary file 1 — The authors have cited additional references within the Supporting Information [88, 100]. [file OPEN-15-e70252-s001.pdf]

Supporting Information
©Wiley-VCH 2026
69451 Weinheim, Germany

Advantages of Asymmetry: A Synthetic and Structural Exploration of *s*-Triazine Chemistry Starting from Pyrrolated Ammeline and Melam

Thaddäus J. Koller, Alexander Pichler, Johannes N. Singer, Zehua Xu, Vasiliki Valsamidou, Reinhard M. Pritzl and Wolfgang Schnick

Abstract: Melam and ammeline are simple, *s*-triazine based compounds first described by Liebig nearly 200 years ago. Outgoing from these two compounds, synthetic strategies for asymmetrically substituted *s*-triazines were developed. As the initial key step, Clauson-Kaas pyrrolation of the compounds' primary amino groups was carried out, significantly improving their solubility in organic solvents and thereby facilitating further conversions. These include functionalization of the *s*-triazine bridging amino group of pyrrolated melam by reaction with electrophiles, while pyrrolated ammeline was deoxychlorinated and subsequently reacted with nucleophiles to displace the resulting Cl substituent. Moreover, a reaction protocol for reversion of pyrrolyl into amino groups was developed, which involved ozonolysis, followed by treatment with aqueous NaOCl of the resulting formamide derivatives. Thereby, melam derivatives with essentially the same coordination site but enhanced solubilities were obtained. This enabled the preparation of a Cu(II) coordination complex from aqueous solution, which is unheard of for melam itself. Finally, the thus accessible *s*-triazines were structurally characterized to gain deeper insights on how the different conducted transformations influence the physicochemical properties relevant for future applications.

DOI: 10.1002/open.2026XXXXX

Table of Contents

Deployed Analysis Methods **3**
Single Crystal X-Ray Diffraction (SCXRD) 3
Powder X-Ray Diffraction (PXRD) 3
Nuclear Magnetic Resonance Spectroscopy (NMR) 3
Fourier Transform Infrared Spectroscopy (FTIR) 3
Ultraviolet/Visible Spectroscopy (UV/Vis) 3
Mass Spectrometry (MS) 3
Elemental Analysis (EA) 3
Experimental Part **4**
General Considerations 4
Experimental Procedures 4
Analytical Results **14**
SCXRD Data 14
PXRD Data 52
NMR Spectra 59
FTIR Spectra 79
UV/Vis Spectra 82
Pictures of Prepared Samples 84
References **86**
Author Contributions **87**

Deployed Analysis Methods

Single Crystal X-Ray Diffraction (SCXRD)

SCXRD measurements for the determination of crystal structures were conducted on a *Bruker D8 Venture* (Mo-K_α radiation) or *Rigaku XtaLAB Synergy* (Cu-K_α radiation) diffractometer. Raw data integration and absorption correction was done on the program *APEX3*[1] or *CrysAlis^{Pro} 1.171.42.102a*.[2] Structure solutions were carried out with *SHELXT-2018/2* and the resulting solutions were refined with *SHELXL-2018/3*.[3] Structure visualizations were done with the program *VESTA 3.4.6*.[4]

Powder X-Ray Diffraction (PXRD)

PXRD measurements on samples filled in glass capillaries (Ø 0.50 mm) were performed on a *Stoe Stadi-P* diffractometer (Cu-$K_{\alpha 1}$ radiation) equipped with a Ge(111)-monochromator and *Dectris Mythen 1 K* silicon strip detector. All PXRD patterns were analyzed either by comparison with theoretical patterns created with the program *WinXPow 3.0.2.1*[5] or by the Rietveld method[6] (i.e. Rietveld refinement) using the program *TOPAS 6*.[7] All results therefrom were visualized with the program *Origin 2019b*.[8]

Nuclear Magnetic Resonance Spectroscopy (NMR)

NMR spectra of samples dissolved in deuterated solvents ($CDCl_3$ or DMSO-d_6) were recorded either on a *Bruker 400* or *Bruker 400 TL* instrument. The resulting data was analyzed and visualized with the program *MestReNova 12.0.2*.[9] The determined chemical shifts δ are reported in ppm relative to the solvent peak ($CDCl_3$: δ_H = 7.26 ppm, δ_C = 77.16 ppm; DMSO-d_6: δ_H = 2.50 ppm, δ_C = 39.52 ppm).[10] For the characterization of the observed signal multiplicities the following abbreviations were used: s: singlet, d: doublet, t: triplet, q: quartet, quin: quintet, m: multiplet (can be used in combination; e.g. dd: doublet of doublet). Coupling constants J are reported in Hz.

Fourier Transform Infrared Spectroscopy (FTIR)

FTIR spectra were recorded in a wavenumber range of 4400–650 cm^{-1} on a *Perkin Elmer Spectrum BX-II* instrument equipped with a *Smiths Detection DuraSampl IR II Diamond ATR* sensor. The wavenumbers $\tilde{\nu}$ of the most significant absorption bands are reported in cm^{-1} and the resulting data was visualized with the program *Origin 2019b*.[8]

Ultraviolet/Visible Spectroscopy (UV/Vis)

UV/Vis spectra were recorded in wavelength range of 200–800 nm on a *Jasco V-650* spectrophotometer. To achieve an optimal visualization of all absorption bands, each sample was mixed with an arbitrary amount of $BaSO_4$ prior to the measurement. The wavelengths λ of the most significant absorption bands are reported in nm and the resulting data was visualized with the program *Origin 2019b*.[8]

Mass Spectrometry (MS)

MS measurements were performed on a *Finnigan MAT 95* instrument. Ionization of the samples was done via electron impact ionization (EI) with an electron energy of 70 eV. The thus obtained *m/z* (mass to charge ratio) signals are reported with their respective percentage intensity relative to the base peak in brackets. The high-resolution mass spectrometry (HRMS) peak of the molecule is reported alongside the theoretically expected value determined with the program *ChemDraw 20*.[11]

Elemental Analysis (EA)

For the determination of C, H and N contents, combustion analyses were performed using an *Elementar vario el* or *Elementar vario micro* instrument. Cl contents were determined by potentiometric titration with $AgNO_3$ on a *Metrohm Titrando 888*. All values are reported in wt.% alongside the theoretically expected values determined with the program *ChemDraw 20*.[11]

Experimental Part

General Considerations

Chemicals/Solvents

All chemicals and solvents were obtained from commercial sources and were used without further purification except for $POCl_3$ and solvents used for flash column chromatography, which were purified via distillation prior to use. The distilled $POCl_3$ was stored in a Schlenk flask under argon atmosphere. The employed Cs_2CO_3 was stored in a furnace at 105 °C for at least 12 h prior to use.

Technical Details

All solvent-state reactions were carried out under magnetic stirring. Moisture sensitive reactions (e.g. deoxychlorination with $POCl_3$) were performed in flame-dried glassware under argon atmosphere using the Schlenk technique and under usage of dry solvents stored in septum-sealed bottles over molecular sieves. Solvent removal was performed using a *Heidolph Hei-VAP Core* rotary evaporator with the water bath temperature set to 40 °C except for AcOH, where the water bath temperature was set to 55 °C. For flash column chromatography and suction filtrations through silica (i.e. silica plug), silica gel with particle sizes of 0.040–0.063 mm and pore sizes of 60 Å was employed.

Experimental Procedures

Preparation of Melam (i.e. Bis(4,6-diamino-1,3,5-triazin-2-yl)amine) (**1**)

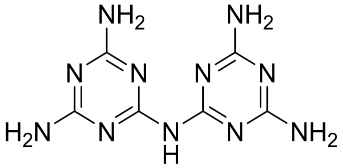

Melamine (5.04 g, 40.0 mmol, 1.0 eq.) and $ZnCl_2$ (5.45 g, 40.0 mmol, 1.0 eq.) were finely ground together with an agate mortar and pestle. The resulting fine powder was transferred into a corundum crucible. After covering the corundum crucible with a lid, it was placed into a muffle furnace, in which it was heated at 300 °C for 12 h. The resulting intermediate was removed from the crucible, ground again and added to boiling aqueous HCl (0.4 M, 500 ml). After stirring for 10 min, the hot suspension was suction filtered and the resulting filtrate was stored at 4 °C overnight. The resulting precipitate was suction filtered off and washed with additional H_2O (3 × 50 ml). Subsequently, the filtered precipitate was added to aqueous NH_3 (25 wt.%, 80 ml). After stirring at room temperature for 2 h, the resulting suspension was suction filtered and the residue was washed with H_2O (3 × 20 ml). The residue was superficially dried at 105 °C, followed by full dehydration at 200 °C for 12 h, which yielded the title compound (1.63 g, 6.94 mmol, 35%) as a white solid.
^{1}H-NMR (400 MHz, DMSO-*d*$_6$, ppm): δ = 9.28 (s, 1H), 6.54 (s, 8H).
^{13}C-NMR (100 MHz, DMSO-*d*$_6$, ppm): δ = 167.3, 164.4.
FTIR (neat, ATR, cm^{-1}): $\tilde{\nu}$ = 3485, 3458, 3302, 3171, 1687, 1641, 1611, 1586, 1547, 1515, 1452, 1416, 1340, 1251, 1175, 1038, 807, 782, 748, 682.
EA (wt.%): calc. for $C_6H_9N_{11}$: C 30.64, H 3.86, N 65.50; found: C 30.48, H 3.91, N 65.36.
HRMS (EI, 70 eV): *m/z* calc. ($C_6H_9N_{11}$): 235.1042; found: 235.1038.
MS (EI, 70 eV, %): *m/z* = 235 (100), 194 (10), 152 (28), 126 (14), 110 (12), 85 (16), 68 (17), 43 (23).

Preparation of Bis(4,6-di(1*H*-pyrrol-1-yl)-1,3,5-triazin-2-yl)amine (**2**)

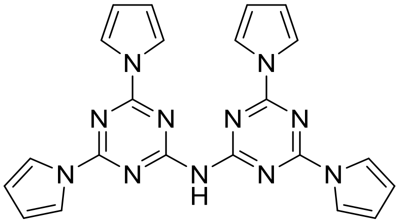

Melam (**1**) (1.18 g, 5.00 mmol, 1.0 eq.) was added to a solution of 2,5-dimethoxyTHF (2.78 g, 21.0 mmol, 4.2 eq.) in AcOH (100 ml). The resulting reaction mixture was stirred at 130 °C under reflux for 5 h. After cooling to room temperature, H_2O (100 ml) was added and the resulting suspension was suction filtered, followed by washing of the residue with additional H_2O (3 × 50 ml). Subsequently, the residue was dissolved in DCM (400 ml) and suction filtered through silica gel, which was then rinsed with additional DCM (3 × 100 ml). After solvent removal, the residue was recrystallized from boiling DCE. After storage at 4 °C overnight, the resulting suspension was suction filtered and the residue was washed with additional ice-cold DCE (20 ml), yielding the title compound (921 mg, 2.12 mmol, 42%) as a white solid. By a second analogous recrystallization of the filtrate, further product (230 mg,

0.528 mmol, 53% combined) was obtained. If necessary, the product may be further purified by sublimation under dynamic vacuum at 220 °C.
^{1}H-NMR (400 MHz, $CDCl_3$, ppm): δ = 8.04 (s, 1H), 7.85–7.79 (m, 8H), 6.42–6.36 (m, 8H).
^{13}C-NMR (100 MHz, $CDCl_3$, ppm): δ = 164.9, 163.0, 119.5, 113.6.
FTIR (neat, ATR, cm^{-1}): $\tilde{\nu}$ = 3260, 3151, 1603, 1565, 1530, 1469, 1428, 1374, 1340, 1309, 1230, 1199, 1093, 1071, 1048, 1036, 1006, 972, 954, 906, 866, 807, 799, 729, 684, 667.
HRMS (EI, 70 eV): *m/z* calc. ($C_{22}H_{17}N_{11}$): 435.1668; found: 435.1683.
MS (EI, 70 eV, %): *m/z* = 435 (100), 210 (6), 118 (25), 93 (8), 67 (5).

Preparation of Ammeline (i.e. 4,6-Diamino-1,3,5-triazin-2(1*H*)-one) (**3**)

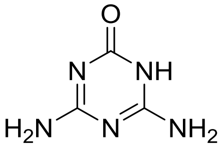

Dicyandiamide (3.36 g, 40.0 mmol, 1.0 eq.) and urea (2.40 g, 40.0 mmol, 1.0 eq.) were finely ground together with an agate mortar and pestle. The resulting fine powder was transferred into a corundum crucible. After covering the corundum crucible with a lid, it was placed into a muffle furnace, in which it was heated at 160 °C for 12 h. The resulting crude product was removed from the crucible, ground again and finally recrystallized from aqueous Na_2CO_3 (0.2 M). The resulting suspension was suction filtered and the residue was washed with H_2O (3 × 50 ml), which yielded the title compound (4.31 g, 33.9 mmol, 85%) as a white solid.
FTIR (neat, ATR, cm^{-1}): $\tilde{\nu}$ = 3465, 3084, 2863, 2654, 1715, 1685, 1610, 1508, 1446, 1410, 1165, 1038, 991, 868, 788, 685.
EA (wt.%): calc. for $C_3H_5N_5O$: C 28.35, H 3.97, N 55.10; found: C 28.12, H 4.04, N 54.51.
HRMS (EI, 70 eV): *m/z* calc. ($C_3H_5N_5O$): 127.0494; found: 127.0489.
MS (EI, 70 eV, %): *m/z* = 127 (100), 111 (10), 85 (6), 68 (6), 43 (40).

Preparation of 4,6-Di(1*H*-pyrrol-1-yl)-1,3,5-triazin-2(1*H*)-one (**4**)

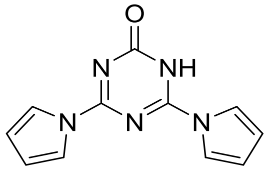

Ammeline (**3**) (1.27 g, 10.0 mmol, 1.0 eq.) was added to a solution of 2,5-dimethoxyTHF (2.91 g, 22.0 mmol, 2.2 eq.) in AcOH (100 ml). The resulting reaction mixture was stirred at 130 °C under reflux for 5 h. Subsequently, the solvents were removed and aqueous NH_3 (0.2 M, 200 ml) was added to the residue. After stirring for 5 h at room temperature, the resulting suspension was suction filtered. The filtrate was adjusted to a pH of 4–5 by dropwise addition of AcOH. The resulting precipitate was suction filtered and washed with additional H_2O (3 × 10 ml). The filtered precipitate was dissolved in aqueous NH_3 (0.2 M, 200 ml) and precipitated again by dropwise addition of AcOH until a pH of 4–5 was reached, which yielded the title compound (1.18 g, 5.20 mmol, 52%) as a beige solid. If necessary, the product may be further purified by sublimation under dynamic vacuum at 250 °C.
^{1}H-NMR (400 MHz, DMSO-*d*$_6$, ppm): δ = 13.40 (s, 1H), 7.85–7.80 (m, 4H), 6.42–6.37 (m, 4H).
^{13}C-NMR (100 MHz, DMSO-*d*$_6$, ppm): δ = 165.8, 161.2, 119.4, 113.3.
FTIR (neat, ATR, cm^{-1}): $\tilde{\nu}$ = 3147, 1632, 1435, 1377, 1317, 1098, 1055, 1016, 944, 885, 808, 753, 730.
HRMS (EI, 70 eV): *m/z* calc. ($C_{11}H_9N_5O$): 227.0807; found: 227.0799.
MS (EI, 70 eV, %): *m/z* = 227 (100), 161 (14), 93 (28), 67 (35).

Preparation of 2-Chloro-4,6-di(1*H*-pyrrol-1-yl)-1,3,5-triazine (**5**)

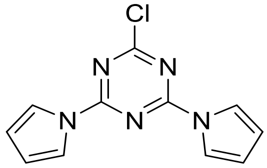

LiCl (423 mg, 10.0 mmol, 2.0 eq.) was added into a Schlenk flask, in which it was dried under dynamic vacuum at 450 °C using a heat gun. **4** (1.14 g, 5.00 mmol, 1.0 eq.) was added to the dried LiCl, which were then both dissolved in dry THF (50 ml). After addition of dry pyridine (396 mg, 0.40 ml, 5.00 mmol, 1.0 eq.), the reaction mixture was cooled down to 0 °C and $POCl_3$ (920 mg, 0.55 ml, 6.00 mmol, 1.2 eq.) was added dropwise. The resulting suspension was stirred at 0 °C for 2 h and subsequently at 50 °C for 36 h. After cooling to room temperature, the reaction was quenched by addition of H_2O (1 ml) and subsequent stirring for 1 min. The quenched mixture was added to a suspension of $MgSO_4$ (5 g) in *i*Hex (200 ml). After stirring for 10 min at room temperature, the resulting suspension was suction filtered through silica gel, which was then rinsed with additional *i*Hex/THF 4:1 (3 × 100 ml). After solvent removal, the residue was sublimed under dynamic vacuum at 75 °C, which yielded the title compound (854 mg, 3.09 mmol, 64%) as a white solid.
^{1}H-NMR (400 MHz, $CDCl_3$, ppm): δ = 7.76–7.71 (m, 4H), 6.42–6.34 (m, 4H).
^{13}C-NMR (100 MHz, $CDCl_3$, ppm): δ = 172.4, 162.7, 119.8, 114.4.

FTIR (neat, ATR, cm^{-1}): $\tilde{\nu}$ = 2920, 2852, 1609, 1554, 1539, 1521, 1490, 1461, 1437, 1373, 1348, 1331, 1273, 1231, 1556, 1069, 1059, 1030, 1024, 982, 947, 870, 861, 817, 802, 780, 738, 707, 669.
HRMS (EI, 70 eV): *m/z* calc. ($C_{11}H_8ClN_5$): 245.0468; found: 245.0472.
MS (EI, 70 eV, %): *m/z* = 245 (100), 118 (24), 92 (41), 87 (10), 66 (6).

Preparation of Ammonium 2-Oxo-4,6-di(1*H*-pyrrol-1-yl)-2*H*-1,3,5-triazin-1-ide (**6**)

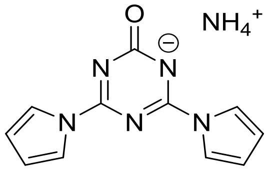

4 (227 mg, 1.00 mmol, 1.0 eq.) was stirred in aqueous NH_3 (25 wt.%, 20 ml) at room temperature for 20 min. The resulting solution was transferred into a crystallizing dish. After solvent evaporation, the resulting crystalline mass was transferred into glass filter crucible, to which ice-cold aqueous NH_3 (25 wt.%, 4 ml) was added. After 2 min, the aqueous NH_3 was removed through suction filtration, yielding the dihydrate of the title compound (210 mg, 0.749 mmol, 75%) as a white solid.
FTIR (neat, ATR, cm^{-1}): $\tilde{\nu}$ = 3605, 3327, 3159, 3031, 1685, 1633, 1571, 1543, 1519, 1459, 1416, 1375, 1314, 1259, 1239, 1187, 1083, 1068, 1047, 1037, 958, 895, 865, 848, 823, 793, 766, 748, 728, 699.
EA (wt.%): calc. for $C_{11}H_{16}N_6O_3$: C 47.14, H 5.75, N 29.98; found: C 47.33, H 5.60, N 29.96.

Preparation of *N*-Methyl-bis(4,6-di(1*H*-pyrrol-1-yl)-1,3,5-triazin-2-yl)amine (**7**)

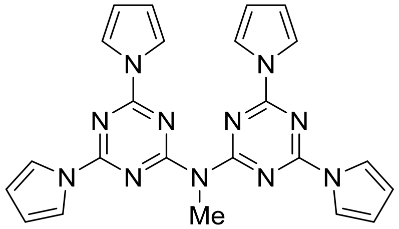

Cs_2CO_3 (782 mg, 2.40 mmol, 1.2 eq.) was added into a Schlenk flask, in which it was dried under dynamic vacuum at 200 °C using a heat gun. **2** (871 mg, 2.00 mmol, 1.0 eq.) and dry THF (30 ml) were successively added to the dried Cs_2CO_3. After dissolution of **2**, MeI (568 mg, 0.25 ml, 4.00 mmol, 2.0 eq.) was added and the resulting reaction mixture was stirred at 50 °C for 12 h. After cooling to room temperature, H_2O (60 ml) was added and the resulting suspension was suction filtered, followed by washing of the residue with H_2O/THF 2:1 (2 × 20 ml) and then H_2O (2 × 20 ml). Subsequently, the residue was dissolved in DCM (100 ml) and suction filtered through silica gel, which was then rinsed with additional DCM (3 × 50 ml). After solvent removal, *c*Hex (20 ml) was added to the residue. After stirring at room temperature for 2 h, the resulting suspension was filtered and the residue was washed with additional *c*Hex (10 ml), yielding the title compound (771 mg, 1.72 mmol, 86%) as a white solid.
^{1}H-NMR (400 MHz, $CDCl_3$, ppm): δ = 7.83–7.77 (m, 8H), 6.40–6.34 (m, 8H), 3.90 (s, 3H).
^{13}C-NMR (100 MHz, $CDCl_3$, ppm): δ = 168.3, 162.7, 119.6, 113.4, 35.4.
FTIR (neat, ATR, cm^{-1}): $\tilde{\nu}$ = 3152, 3105, 1596, 1557, 1528, 1464, 1439, 1396, 1364, 1262, 1237, 1225, 1202, 1155, 1068, 1059, 1035, 957, 902, 866, 856, 808, 786, 723, 695.
HRMS (EI, 70 eV): *m/z* calc. ($C_{23}H_{19}N_{11}$): 449.1825; found: 449.1811.
MS (EI, 70 eV, %): *m/z* = 449 (100), 239 (24), 118 (17), 93 (17), 67 (5).

Preparation of *N*-Ethyl-bis(4,6-di(1*H*-pyrrol-1-yl)-1,3,5-triazin-2-yl)amine (**8**)

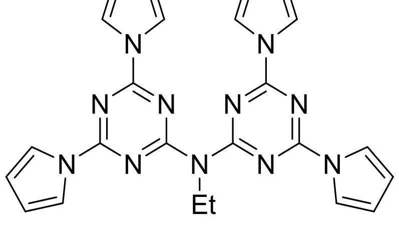

Cs_2CO_3 (782 mg, 2.40 mmol, 1.2 eq.) was added into a Schlenk flask, in which it was dried under dynamic vacuum at 200 °C using a heat gun. **2** (871 mg, 2.00 mmol, 1.0 eq.) and dry THF (30 ml) were successively added to the dried Cs_2CO_3. After dissolution of **2**, EtI (624 mg, 0.32 ml, 4.00 mmol, 2.0 eq.) was added and the resulting reaction mixture was stirred at 50 °C for 36 h. After cooling to

room temperature, H_2O (60 ml) was added and the resulting suspension was suction filtered, followed by washing of the residue with H_2O/THF 2:1 (2 × 20 ml) and then H_2O (2 × 20 ml). Subsequently, the residue was dissolved in DCM (100 ml) and suction filtered through silica gel, which was then rinsed with additional DCM (3 × 50 ml). After solvent removal, *c*Hex (20 ml) was added to the residue. After stirring at room temperature for 2 h, the resulting suspension was filtered and the residue was washed with additional *c*Hex (10 ml), yielding the title compound (779 mg, 1.68 mmol, 84%) as a white solid.
^{1}H-NMR (400 MHz, $CDCl_3$, ppm): δ = 7.82–7.76 (m, 8H), 6.40–6.34 (m, 8H), 4.54 (q, *J* = 7.0 Hz, 2H), 1.49 (t, *J* = 7.0 Hz, 3H).
^{13}C-NMR (100 MHz, $CDCl_3$, ppm): δ = 167.9, 162.8, 119.6, 113.3, 43.7, 13.8.
FTIR (neat, ATR, cm^{-1}): $\tilde{\nu}$ = 3152, 2969, 1596, 1569, 1528, 1462, 1439, 1369, 1328, 1284, 1262, 1194, 1150, 1127, 1067, 1049, 1033, 987, 968, 955, 929, 909, 867, 811, 787, 754, 722, 688, 658.
HRMS (EI, 70 eV): *m/z* calc. ($C_{24}H_{21}N_{11}$): 463.1981; found: 463.1970.
MS (EI, 70 eV, %): *m/z* = 463 (100), 253 (69), 210 (5), 118 (27), 93 (19), 67 (6).

Preparation of *N*-Cyano-bis(4,6-di(1*H*-pyrrol-1-yl)-1,3,5-triazin-2-yl)amine (**9**)

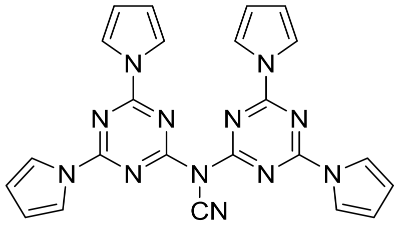

Cs_2CO_3 (977 mg, 3.00 mmol, 1.5 eq.) was added into a Schlenk flask, in which it was dried under dynamic vacuum at 200 °C using a heat gun. **2** (871 mg, 2.00 mmol, 1.0 eq.) and dry THF (30 ml) were successively added to the dried Cs_2CO_3. After dissolution of **2**, BrCN (635 mg, 6.00 mmol, 3.0 eq.) was added and the resulting reaction mixture was stirred at 50 °C for 36 h. After cooling to room temperature, H_2O (60 ml) was added and the resulting suspension was suction filtered, followed by washing of the residue with H_2O/THF 2:1 (2 × 20 ml) and then H_2O (2 × 20 ml). Subsequently, the residue was dissolved in hot DCM (600 ml) and suction filtered through silica gel, which was then rinsed with additional hot DCM (3 × 100 ml). After solvent removal, *c*Hex (20 ml) was added to the residue. After stirring at room temperature for 2 h, the resulting suspension was filtered and the residue was washed with additional *c*Hex (10 ml), yielding the title compound (744 mg, 1.62 mmol, 81%) as a white solid.
^{1}H-NMR (400 MHz, $CDCl_3$, ppm): δ = 7.79 (t, *J* = 2.4 Hz, 8H), 6.41 (t, *J* = 2.4 Hz, 8H).
^{13}C-NMR (100 MHz, $CDCl_3$, ppm): δ = 165.0, 163.1, 119.9, 114.6, 105.4.
FTIR (neat, ATR, cm^{-1}): $\tilde{\nu}$ = 3143, 3122, 2250, 1598, 1561, 1528, 1467, 1442, 1355, 1303, 1258, 1180, 1106, 1070, 1051, 1034, 1013, 953, 915, 866, 804, 784, 731.
HRMS (EI, 70 eV): *m/z* calc. ($C_{23}H_{16}N_{12}$): 460.1621; found: 460.1606.
MS (EI, 70 eV, %): *m/z* = 460 (100), 394 (7), 210 (8), 118 (48), 92 (7), 66 (6).

Preparation of Tris(4,6-di(1*H*-pyrrol-1-yl)-1,3,5-triazin-2-yl)amine (**10**)

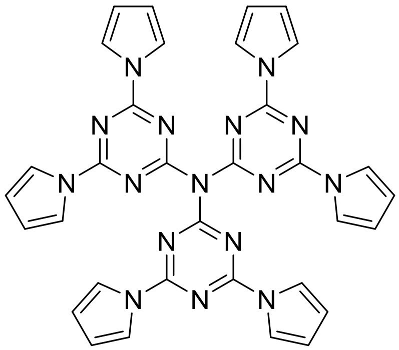

NaH (62.4 mg, 2.60 mmol, 1.3 eq.) was added to a solution of **2** (871 mg, 2.00 mmol, 1.0 eq.) in dry DMF (30 ml). After stirring for 20 min at room temperature, **5** (639 mg, 2.60 mmol, 1.3 eq.) was added and the resulting reaction mixture was stirred at 70 °C for 36 h. After cooling to room temperature, H_2O (60 ml) was added and the resulting suspension was suction filtered, followed by washing of the residue with H_2O/THF 2:1 (2 × 20 ml) and then H_2O (2 × 20 ml). Subsequently, the residue was dissolved in DCM (200 ml) and suction filtered through silica gel, which was then rinsed with additional DCM (3 × 50 ml). After solvent removal, Et_2O

(200 ml) was added to the residue, followed by stirring at room temperature for 2 h. The resulting suspension was suction filtered and the residue was washed with additional Et_2O (2 × 15 ml). Subsequently, the residue was recrystallized from boiling DCE. After storage at 4 °C overnight, the resulting suspension was suction filtered and the residue was washed with additional ice-cold DCE (10 ml), yielding the title compound (418 mg, 0.649 mmol, 32%) as a white solid.
^{1}H-NMR (400 MHz, $CDCl_3$, ppm): δ = 7.71–7.67 (m, 12H), 6.37–6.32 (m, 12H).
^{13}C-NMR (100 MHz, $CDCl_3$, ppm): δ = 168.2, 163.3, 119.7, 113.9.
FTIR (neat, ATR, cm^{-1}): $\tilde{\nu}$ = 3149, 3105, 1577, 1557, 1534, 1514, 1461, 1434, 1372, 1351, 1305, 1261, 1228, 1107, 1069, 1051, 1032, 1011, 952, 917, 863, 806, 785, 763, 731.
HRMS (EI, 70 eV): *m/z* calc. ($C_{33}H_{24}N_{16}$): 644.2370; found: 644.2361.
MS (EI, 70 eV, %): *m/z* = 644 (100), 435 (5), 327 (5), 210 (22), 118 (50), 93 (8).

Preparation of Bis(4,6-di(1*H*-pyrrol-1-yl)-1,3,5-triazin-2-yl)oxide (**11**)

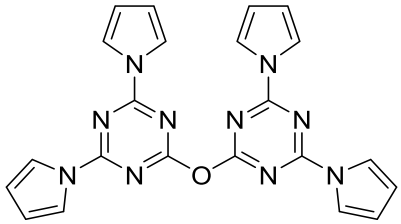

5 (737 mg, 3.00 mmol, 1.0 eq.) was added to **4** (818 mg, 3.60 mmol, 1.2 eq.) and dry NEt_3 (334 mg, 0.46 ml, 3.30 mmol, 1.1 eq.) in dry THF (30 ml). The resulting reaction mixture was stirred at 50 °C for 36 h. After cooling to room temperature, H_2O (60 ml) was added and the resulting suspension was suction filtered, followed by washing of the residue with H_2O/THF 2:1 (2 × 20 ml) and then H_2O (2 × 20 ml). Subsequently, the residue was dissolved in DCM (200 ml) and suction filtered through silica gel, which was then rinsed with additional DCM (3 × 50 ml). After solvent removal, the residue was recrystallized from boiling DCE. After storage at 4 °C overnight, the resulting suspension was suction filtered and the residue was washed with additional ice-cold DCE (10 ml), yielding the title compound (864 mg, 1.98 mmol, 66%) as a white solid. By a second analogous recrystallization of the filtrate, further product (159 mg, 0.365 mmol, 78% combined) was obtained.
^{1}H-NMR (400 MHz, $CDCl_3$, ppm): δ = 7.78–7.71 (m, 8H), 6.41–6.35 (m, 8H).
^{13}C-NMR (100 MHz, $CDCl_3$, ppm): δ = 170.4, 164.3, 119.8, 114.2.
FTIR (neat, ATR, cm^{-1}): $\tilde{\nu}$ = 3148, 1600, 1564, 1527, 1466, 1445, 1378, 1341, 1295, 1254, 1186, 1091, 1070, 1050, 1031, 952, 903, 882, 805, 786, 736, 714, 673.
HRMS (EI, 70 eV): *m/z* calc. ($C_{22}H_{16}N_{10}O$): 436.1509; found: 436.1504.
MS (EI, 70 eV, %): *m/z* = 436 (88), 370 (100), 210 (11), 186 (11), 118 (78), 92 (11), 66 (12).

Preparation of 2-Cyano-4,6-di(1*H*-pyrrol-1-yl)-1,3,5-triazine (**12**)

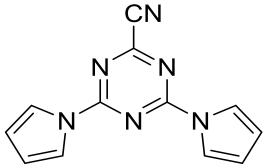

KCN (215 mg, 3.30 mmol, 1.1 eq.) was added to a solution of **5** (737 mg, 3.00 mmol, 1.0 eq.) in dry DMF (10 ml). The resulting reaction mixture was stirred at 50 °C for 36 h. After cooling to room temperature, H_2O (20 ml) was added and the resulting suspension was suction filtered, followed by washing of the residue with H_2O/DMF 2:1 (2 × 20 ml) and then H_2O (2 × 20 ml). Subsequently, the residue was dissolved in DCM (100 ml) and suction filtered through silica gel, which was then rinsed with additional DCM (3 × 50 ml). After solvent removal, the residue was sublimed under dynamic vacuum at 85 °C, which yielded the title compound (463 mg, 1.96 mmol, 65%) as a white solid.
^{1}H-NMR (400 MHz, $CDCl_3$, ppm): δ = 7.78–7.69 (m, 4H), 6.46–6.40 (m, 4H).
^{13}C-NMR (100 MHz, $CDCl_3$, ppm): δ = 162.3, 154.2, 119.8, 115.2, 114.2.
FTIR (neat, ATR, cm^{-1}): $\tilde{\nu}$ = 3147, 2925, 2256, 1558, 1535, 1519, 1464, 1440, 1375, 1288, 1248, 1182, 1172, 1087, 1071, 1030, 986, 946, 866, 852, 810, 785, 744, 726, 704.
HRMS (EI, 70 eV): *m/z* calc. ($C_{12}H_8N_6$): 236.0810; found: 236.0806.
MS (EI, 70 eV, %): *m/z* = 236 (100), 118 (12), 92 (40), 78 (7), 66 (7).

Preparation of 1,2-Bis(4,6-di(1*H*-pyrrol-1-yl)-1,3,5-triazin-2-yl)diazane (**13i**)

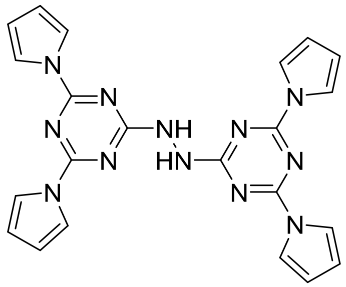

N_2H_4·HCl (68.5 mg, 1.00 mmol, 1.0 eq.) and dry pyridine (237 mg, 0.24ml, 3.00 mmol, 3.0 eq.) were successively added to a solution of **5** (491 mg, 2.00 mmol, 2.0 eq.) in dry DMF (20 ml). The resulting reaction mixture was stirred at 50 °C for 12 h. After cooling to room temperature, H_2O (40 ml) was added and the resulting suspension was suction filtered, followed by washing of the residue with H_2O/DMF 2:1 (2 × 20 ml) and then H_2O (2 × 20 ml). The residue was recrystallized from boiling DCE. After storage at 4 °C overnight, the resulting suspension was suction filtered and the residue was washed with additional ice-cold DCE (10 ml), yielding the title compound (303 mg, 0.672 mmol, 67%) as a white solid.
^{1}H-NMR (400 MHz, DMSO-*d*$_6$, ppm): δ = 10.55 (s, 2H), 7.90–7.80 (m, 4H), 7.68–7.62 (m, 4H), 6.44–6.38 (m, 4H), 6.31–6.25 (m, 4H).
^{13}C-NMR (100 MHz, DMSO-*d*$_6$, ppm): δ = 168.3, 162.0, 161.7, 119.1, 119.0, 112.9.
FTIR (neat, ATR, cm^{-1}): $\tilde{\nu}$ = 3377, 3146, 3117, 1563, 1546, 1527, 1462, 1447, 1417, 1368, 1294, 1263, 1244, 1179, 1070, 1031, 992, 977, 953, 893, 864, 802, 788, 749, 738, 726, 715.
HRMS (EI, 70 eV): *m/z* calc. ($C_{22}H_{18}N_{12}$): 450.1777; found: 450.1758.
MS (EI, 70 eV, %): *m/z* = 450 (100), 292 (5), 226 (14), 118 (6), 93 (10), 67 (5).

Preparation of (*E*)-1,2-Bis(4,6-di(1*H*-pyrrol-1-yl)-1,3,5-triazin-2-yl)diazene (**13**)

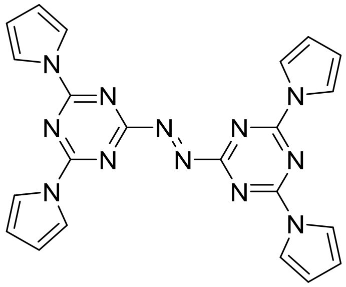

KO*t*Bu (112 mg, 1.00 mmol, 2.0 eq.) and *N*-chlorosuccinimide (66.8 mg, 0.500 mmol, 1.0 eq.) were successively added to a solution of **13i** (225 mg, 0.500 mmol, 1.0 eq.) in dry THF (10 ml). The resulting reaction mixture was stirred at room temperature for 30 min. Subsequently, H_2O (20 ml) was added and the resulting suspension was suction filtered, followed by washing of the residue with H_2O/THF 2:1 (2 × 20 ml) and then H_2O (2 × 20 ml). The residue was recrystallized from boiling DCE. After storage at 4 °C overnight, the resulting suspension was suction filtered and the residue was washed with additional ice-cold DCE (10 ml), yielding the title compound (170 mg, 0.379 mmol, 76%) as a yellow solid.
^{1}H-NMR (400 MHz, CDCl$_3$, ppm): δ = 7.90–7.85 (m, 8H), 6.46–6.41 (m, 8H).
^{13}C-NMR (100 MHz, CDCl$_3$, ppm): δ = 175.6, 163.9, 120.0, 114.5.
FTIR (neat, ATR, cm^{-1}): $\tilde{\nu}$ = 3148, 1563, 1541, 1518, 1464, 1443, 1367, 1294, 1247, 1170, 1080, 1066, 1031, 988, 951, 881, 866, 821, 783, 749, 733, 702.
UV/Vis (BaSO$_4$, nm): λ = 261, 354.
HRMS (EI, 70 eV): *m/z* calc. ($C_{22}H_{16}N_{12}$): 448.1621; found: 448.1632.
MS (EI, 70 eV, %): *m/z* = 450 (98), 420 (14), 354 (6), 292 (7), 226 (100), 210 (26), 160 (13), 118 (79), 93 (52), 67 (43).

Preparation of (*E*)-2-(Phenyldiazenyl)-4,6-di(1*H*-pyrrol-1-yl)-1,3,5-triazine (**14**)

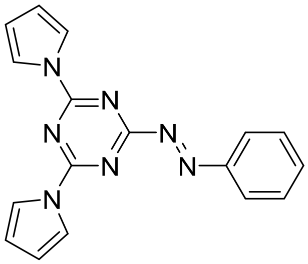

Phenylhydrazine (454 mg, 0.41 ml, 4.20 mmol, 2.1 eq.) was added to a solution of **5** (491 mg, 2.00 mmol, 1.0 eq.) in dry THF (10 ml). The resulting reaction mixture was stirred at room temperature for 12 h. Subsequently, H_2O (50 ml) and DCM (100 ml) were added. After stirring at room temperature for 10 min, the organic phase was separated off and the aqueous phase was extracted with additional DCM (2 × 50 ml). The combined organic phases were successively washed with aqueous HCl (0.4 M, 50 ml) and saturated aqueous $NaHCO_3$ (50 ml). After drying with $MgSO_4$ and subsequent solvent removal, the resulting residue was dissolved in THF (10 ml), followed by successive addition of NEt_3 (607 mg, 0.84 ml, 6.00 mmol, 3.0 eq.) and *N*-chlorosuccinimide (267 mg, 2.00 mmol,

1.0 eq.). After stirring at room temperature for 12 h, aqueous HCl (0.4 M, 50 ml) and DCM (100 ml) were added. After stirring at room temperature for 10 min, the organic phase was separated off and the aqueous phase was extracted with additional DCM (2 × 50 ml). The combined organic phases were successively washed with aqueous HCl (0.4 M, 50 ml) and saturated aqueous $NaHCO_3$ (50 ml). After drying with $MgSO_4$ and subsequent solvent removal, the resulting residue was purified via flash column chromatography (silica gel; *i*Hex:DCM 7:3), which yielded the title compound (234 mg, 0.743 mmol, 37%) as an orange solid.
^{1}H-NMR (400 MHz, $CDCl_3$, ppm): δ = 8.14–8.07 (m, 2H), 7.90–7.84 (m, 4H), 7.67–7.55 (m, 3H), 6.45–6.37 (m, 4H).
^{13}C-NMR (100 MHz, $CDCl_3$, ppm): δ = 176.2, 163.9, 152.5, 134.1, 129.5, 124.5, 119.9, 114.0.
FTIR (neat, ATR, cm^{-1}): $\tilde{\nu}$ = 3145, 3103, 2960, 1562, 1540, 1522, 1495, 1465, 1439, 1366, 1314, 1294, 1260, 1245, 1198, 1171, 1149, 1096, 1069, 1034, 1000, 953, 917, 860, 811, 783, 765, 747, 733, 707, 697, 679.
UV/Vis ($BaSO_4$, nm): λ = 263, 350, 475.
HRMS (EI, 70 eV): *m/z* calc. ($C_{17}H_{13}N_7$): 315.1232; found: 315.1236.
MS (EI, 70 eV, %): *m/z* = 315 (19), 287 (12), 226 (5), 118 (27), 105 (51), 77 (100).

Preparation of *N*-Methyl-bis(4,6-diamino-1,3,5-triazin-2-yl)amine (**15**)

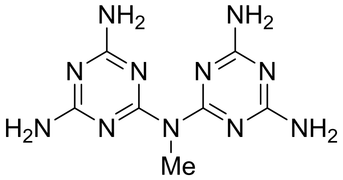

O_3 was introduced via a gas inlet into a solution of **7** (449 mg, 1.00 mmol, 1.0 eq.) in DCM (100 ml) at −78 °C for 10 min until the solution started to turn blue. After removal of excess O_3 by introduction of Ar for 10 min, Me_2S (1.24 g, 1.47 ml, 20.0 mmol, 20 eq.) was added dropwise at −78 °C. Subsequently, the reaction mixture was stirred at −78 °C for 1 h and then at room temperature for 5 h (Note: While stirring at −78 °C and during warming up to room temperature, the reaction flask was not stoppered). After addition of *i*Hex (150 ml), the resulting suspension was suction filtered, followed by washing of the residue with *i*Hex/DCM 3:2 (2 × 20 ml). The residue was added to ice-cold H_2O (10 ml). After stirring for 5 min, ice-cold aqueous NaOCl (13% active chlorine, 30 ml) was added, to which aqueous H_3PO_4 (85 wt.%, 0.6 ml) had been added beforehand. After stirring at room temperature for 1 h, the temperature of the reaction mixture was increased to 50 °C, at which it was stirred for additional 2 h. After cooling to room temperature, the resulting suspension was suction filtered, followed by washing of the residue with H_2O (3 × 10 ml). The residue was added to *i*PrOH (20 ml), in which it was stirred at room temperature for 2 h (Note: If significant C=O stretching vibrations are still detectable in the IR spectrum after treatment with aqueous NaOCl, it is advised to stir the intermediate in *i*PrOH for additional 2 h at 50 °C). The resulting suspension was suction filtered, followed by washing of the residue with *i*PrOH (2 × 5 ml). The residue was added to aqueous NH_3 (25 wt.%, 20 ml), in which it was stirred at room temperature for 2 h. Suction filtration of the resulting suspension, followed by washing of the residue with H_2O (2 × 10 ml) and then Me_2CO (2 × 10 ml), yielded the monohydrate of the title compound (156 mg, 0.583 mmol, 58%) as a white solid.
^{1}H-NMR (400 MHz, DMSO-*d*$_6$, ppm): δ = 6.48 (s, 8H), 3.24 (s, 3H).
^{13}C-NMR (100 MHz, DMSO-*d*$_6$, ppm): δ = 168.1, 167.6, 34.6.
FTIR (neat, ATR, cm^{-1}): $\tilde{\nu}$ = 3486, 3315, 3130, 1684, 1633, 1530, 1476, 1450, 1359, 1281, 1237, 1136, 1088, 1050, 1023, 945, 812, 766, 735.
HRMS (EI, 70 eV): *m/z* calc. ($C_7H_{11}N_{11}$): 249.1199; found: 249.1191.
MS (EI, 70 eV, %): *m/z* = 249 (100), 164 (5), 139 (73), 97 (7), 69 (14), 43 (20).

Preparation of *N*-Ethyl-bis(4,6-diamino-1,3,5-triazin-2-yl)amine (**16**)

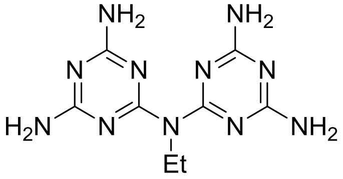

O_3 was introduced via a gas inlet into a solution of **8** (464 mg, 1.00 mmol, 1.0 eq.) in DCM (40 ml) at −78 °C for 10 min until the solution started to turn blue. After removal of excess O_3 by introduction of Ar for 10 min, Me_2S (1.24 g, 1.47 ml, 20.0 mmol, 20 eq.) was added dropwise at −78 °C. Subsequently, the reaction mixture was stirred at −78 °C for 1 h and then at room temperature for 5 h (Note: While stirring at −78 °C and during warming up to room temperature, the reaction flask was not stoppered). After addition of *i*Hex (60 ml), the resulting suspension was suction filtered, followed by washing of the residue with *i*Hex/DCM 3:2 (2 × 20 ml). The dry residue was added to ice-cold H_2O (10 ml). After stirring for 5 min, ice-cold aqueous NaOCl (13% active chlorine, 30 ml) was added, to which aqueous H_3PO_4 (85 wt.%, 0.6 ml) had been added beforehand. After stirring at room temperature for 1 h, the temperature of the reaction mixture was increased to 50 °C, at which it was stirred for additional 2 h. After cooling to room temperature, the resulting suspension was suction filtered, followed by washing of the residue with H_2O (3 × 10 ml). The residue was added to *i*PrOH (10 ml), in which it was stirred at room temperature for 2 h (Note: If significant C=O stretching vibrations are still detectable in the IR spectrum after treatment with aqueous NaOCl, it is advised to stir the intermediate in *i*PrOH for additional 2 h at 50 °C). After storage at 4 °C for 3 h, the resulting suspension was suction filtered, followed by washing of the residue with ice-cold *i*PrOH (2 × 5 ml). The residue was added to aqueous NH_3 (25 wt.%, 10 ml), in which it was stirred at room temperature for 2 h. Suction filtration of the resulting

suspension, followed by washing of the residue with H_2O (2 × 5 ml) and then Me_2CO (2 × 10 ml), yielded the sesquihydrate of the title compound (122 mg, 0.420 mmol, 42%) as a white solid.
^{1}H-NMR (400 MHz, DMSO-*d*$_6$, ppm): δ = 6.45 (s, 8H), 3.86 (q, J = 6.9 Hz, 2H), 1.09 (t, J = 6.9 Hz, 3H).
^{13}C-NMR (100 MHz, DMSO-*d*$_6$, ppm): δ = 167.6, 167.6, 41.3, 14.1.
FTIR (neat, ATR, cm^{-1}): $\tilde{\nu}$ = 3463, 3311, 3124, 1620, 1525, 1441, 1371, 1347, 1293, 1087, 1024, 905, 824, 804, 738.
HRMS (EI, 70 eV): *m/z* calc. ($C_8H_{13}N_{11}$): 263.1355; found: 263.1357.
MS (EI, 70 eV, %): *m/z* = 263 (80), 248 (14), 220 (7), 154 (100), 139 (7), 111 (16), 85 (11), 68 (24), 42 (31).

Preparation of *N*-Cyano-bis(4,6-diamino-1,3,5-triazin-2-yl)amine (**17**)

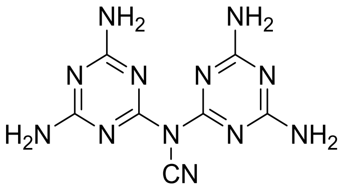

O_3 was introduced via a gas inlet into a solution of **9** (230 mg, 0.500 mmol, 1.0 eq.) in DCM (200 ml) at −78 °C for 10 min until the solution started to turn blue. After removal of excess O_3 by introduction of Ar for 10 min, Me_2S (621 mg, 0.73 ml, 10.0 mmol, 20 eq.) was added dropwise at −78 °C. Subsequently, the reaction mixture was stirred at −78 °C for 1 h and then at room temperature for 5 h (Note: While stirring at −78 °C and during warming up to room temperature, the reaction flask was not stoppered). After addition of *i*Hex (400 ml), the resulting suspension was suction filtered, followed by washing of the residue with *i*Hex/DCM 3:2 (2 × 20 ml). The dry residue was added to ice-cold H_2O (5 ml). After stirring for 5 min, ice-cold aqueous NaOCl (13% active chlorine, 15 ml) was added portion wise, to which aqueous H_3PO_4 (85 wt.%, 0.3 ml) had been added beforehand. After stirring at 0 °C for 1 h, the resulting suspension was suction filtered, followed by washing of the residue with H_2O (3 × 10 ml). The dry residue was added to ice-cold *i*PrOH (10 ml), in which it was stirred for at room temperature for 2 h. The resulting suspension was suction filtered, followed by washing of the residue with *i*PrOH (2 × 5 ml). The dry residue was added to aqueous NH_3 (25 wt.%, 10 ml), in which it was stirred at room temperature for 2 h. Suction filtration of the resulting suspension, followed by washing of the residue with H_2O (2 × 5 ml) and then Me_2CO (2 × 5 ml), yielded the title compound (56.3 mg, 0.216 mmol, 43%) as a white solid.
^{1}H-NMR (400 MHz, DMSO-*d*$_6$, ppm): δ = 7.16 (s, 4H), 7.02 (s, 4H).
^{13}C-NMR (100 MHz, DMSO-*d*$_6$, ppm): δ = 167.6, 163.9, 109.3.
FTIR (neat, ATR, cm^{-1}): $\tilde{\nu}$ = 3491, 3344, 3154, 2254, 1687, 1634, 1601, 1530, 1335, 1242, 798.
HRMS (EI, 70 eV): *m/z* calc. ($C_7H_8N_{12}$): 260.0995; found: 260.0985.
MS (EI, 70 eV, %): *m/z* = 260 (91), 235 (63), 217 (46), 192 (6), 152 (54), 126 (34), 84 (19), 68 (49), 42 (100).

Preparation of Tris(4,6-diamino-1,3,5-triazin-2-yl)amine (**18**)

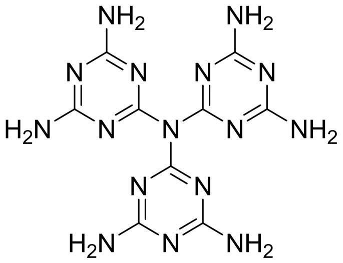

O_3 was introduced via a gas inlet into a solution of **10** (322 mg, 0.500 mmol, 1.0 eq.) in DCM (50 ml) at −78 °C for 10 min until the solution started to turn blue. After removal of excess O_3 by introduction of Ar for 10 min, Me_2S (621 mg, 0.73 ml, 10.0 mmol, 20 eq.) was added dropwise at −78 °C. Subsequently, the reaction mixture was stirred at −78 °C for 1 h and then at room temperature for 5 h (Note: While stirring at −78 °C and during warming up to room temperature, the reaction flask was not stoppered). After addition of *i*Hex (75 ml), the resulting suspension was suction filtered, followed by washing of the residue with *i*Hex/DCM 3:2 (2 × 20 ml). The dry residue was added to ice-cold H_2O (5 ml). After stirring for 5 min, ice-cold aqueous NaOCl (13% active chlorine, 15 ml) was added portion wise, to which aqueous H_3PO_4 (85 wt.%, 0.3 ml) had been added beforehand. After stirring at 0 °C for 1 h, the resulting suspension was suction filtered, followed by washing of the residue with H_2O (3 × 10 ml). The residue was added to ice-cold *i*PrOH (10 ml), in which it was stirred for at room temperature for 2 h. The resulting suspension was suction filtered, followed by washing of the residue with *i*PrOH (2 × 5 ml). The dry residue was added to aqueous NH_3 (25 wt.%, 10 ml), in which it was stirred at room temperature for 2 h. Suction filtration of the resulting suspension, followed by washing of the residue with H_2O (2 × 5 ml) and then Me_2CO (2 × 5 ml), yielded the dihydrate of the title compound (76.3 mg, 0.201 mmol, 40%) as a white solid.
^{1}H-NMR (400 MHz, DMSO-*d*$_6$, ppm): δ = 6.79 (s, 6H), 6.51 (s, 6H).
^{13}C-NMR (100 MHz, DMSO-*d*$_6$, ppm): δ = 168.0, 168.0.
FTIR (neat, ATR, cm^{-1}): $\tilde{\nu}$ = 3474, 3418, 3314, 3138, 1626, 1585, 1556, 1514, 1462, 1344, 1274, 1213, 1167, 1070, 1026, 984, 843, 827, 810, 796, 731, 684.
HRMS (EI, 70 eV): *m/z* calc. ($C_9H_{12}N_{16}$): 344.1431; found: 344.1427.
MS (EI, 70 eV, %): *m/z* = 344 (68), 303 (11), 261 (10). 235 (23), 192 (22), 152 (71), 126 (16), 110 (32), 85 (24), 68 (68), 42 (100).

Preparation of Bis(4,6-diamino-1,3,5-triazin-2-yl)oxide (**19**)

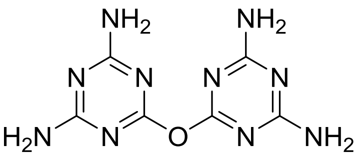

O_3 was introduced via a gas inlet into a solution of **11** (436 mg, 1.00 mmol, 1.0 eq.) in DCM (100 ml) at −78 °C for 10 min until the solution started to turn blue. After removal of excess O_3 by introduction of Ar for 10 min, Me_2S (1.24 g, 1.47 ml, 20.0 mmol, 20 eq.) was added dropwise at −78 °C. Subsequently, the reaction mixture was stirred at −78 °C for 1 h and then at room temperature for 5 h (Note: While stirring at −78 °C and during warming up to room temperature, the reaction flask was not stoppered). After addition of *i*Hex (150 ml), the resulting suspension was suction filtered, followed by washing of the residue with *i*Hex/DCM 3:2 (2 × 20 ml). The dry residue was added to ice-cold H_2O (10 ml). After stirring for 5 min, ice-cold aqueous NaOCl (13% active chlorine, 30 ml) was added portion wise, to which aqueous H_3PO_4 (85 wt.%, 0.6 ml) had been added beforehand. After stirring at 0 °C for 1 h, the resulting suspension was suction filtered, followed by washing of the residue with H_2O (3 × 10 ml). The residue was added to ice-cold *i*PrOH (10 ml), in which it was stirred for at room temperature for 2 h. The resulting suspension was suction filtered, followed by washing of the residue with *i*PrOH (2 × 5 ml). The residue was added to aqueous NH_3 (25 wt.%, 10 ml), in which it was stirred at room temperature for 2 h. Suction filtration of the resulting suspension, followed by washing of the residue with H_2O (2 × 5 ml) and then Me_2CO (2 × 5 ml), yielded the title compound (49.3 mg, 0.209 mmol, 21%) as a white solid.
^{1}H-NMR (400 MHz, DMSO-*d*$_6$, ppm): δ = 6.90 (s, 4H), 6.81 (s, 4H).
^{13}C-NMR (100 MHz, DMSO-*d*$_6$, ppm): δ = 169.4, 168.7.
FTIR (neat, ATR, cm^{-1}): $\tilde{\nu}$ = 3448, 3093, 1635, 1583, 1528, 1479, 1410, 1309, 1148, 1057, 1029, 834, 806, 704.
HRMS (EI, 70 eV): *m/z* calc. ($C_6H_8N_{10}O$): 236.0883; found: 236.0875.
MS (EI, 70 eV, %): *m/z* = 236 (74), 208 (100), 194 (68), 177 (11), 152 (43), 126 (66), 110 (15), 85 (13).

Preparation of Bis(*N*-ethyl-bis(4,6-diamino-1,3,5-triazin-2-yl)amine)copper(II) Diperchlorate (**20**)

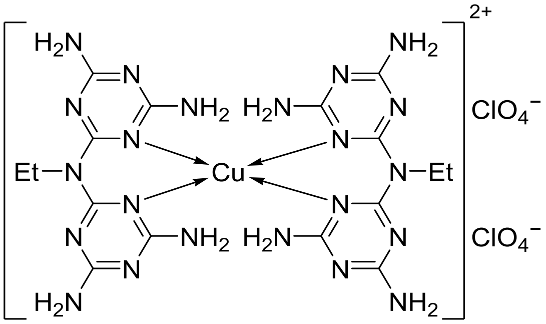

$Cu(ClO_4)_2$·$6H_2O$ (74.1 mg, 0.200 mmol, 1.0 eq.) dissolved in H_2O (2 ml) was added to a solution of **16·1.5H$_2$O** (116 mg, 0.400 mmol, 2.0 eq.) in H_2O (18 ml) at 70 °C. After stirring at 70 °C for 5 min, the resulting solution was allowed to slowly cool down to room temperature. Suction filtration of the resulting suspension, followed by washing of the residue with H_2O (5 ml), yielded the trihydrate of the title compound (106 mg, 0.126 mmol, 63%) as a brown solid.
FTIR (neat, ATR, cm^{-1}): $\tilde{\nu}$ = 3546, 3443, 3385, 3354, 3241, 3094, 1687, 1650, 1627, 1556, 1530, 1469, 1423, 1371, 1292, 1159, 1118, 1072, 1039, 926, 898, 801, 751, 725, 653.
EA (wt.%): calc. for $C_{16}H_{32}Cl_2CuN_{22}O_{11}$: C 22.80, H 3.83, N 36.55, Cl 8.41; found: C 22.95, H 3.82, N 36.45, Cl 8.50.
UV/Vis (BaSO$_4$, nm): λ = 228, 261, 398, 515, 658.

Analytical Results

SCXRD Data

Table S1. Selected crystallographic data of compounds **4**, **5**, **6**, **7**, **8** and **9**.

Formula	$C_{11}H_9N_5O$ (**4**)	$C_{11}H_8ClN_5$ (**5**)	$C_{11}H_{16}N_6O_3$ (**6·2H₂O**)	$C_{23}H_{19}N_{11}$ (**7**)	$C_{24}H_{21}N_{11}$ (**8**)	$C_{23}H_{16}N_{12}$ (**9**)
Molar mass [g/mol]	227.23	245.67	280.30	449.49	463.52	460.48
Crystal system	monoclinic	monoclinic	monoclinic	monoclinic	monoclinic	orthorhombic
Space group	$C2/c$ (no. 15)	$P2_1$ (no. 4)	$C2/c$ (no. 15)	$P2_1/c$ (no. 14)	$P2_1/n$ (no. 14)	$Pna2_1$ (no. 33)
Lattice parameters [Å; °]	a = 25.241(5)<br>b = 5.9342(13)<br>c = 14.104(3)<br>α = 90<br>β = 92.808(9)<br>γ = 90	a = 4.4385(7)<br>b = 17.383(3)<br>c = 14.669(2)<br>α = 90<br>β = 96.382(6)<br>γ = 90	a = 34.738(2)<br>b = 4.6484(3)<br>c = 16.8676(9)<br>α = 90<br>β = 103.161(2)<br>γ = 90	a = 3.9943(4)<br>b = 19.2532(19)<br>c = 28.150(3)<br>α = 90<br>β = 91.389(3)<br>γ = 90	a = 7.1320(8)<br>b = 19.814(2)<br>c = 16.2021(18)<br>α = 90<br>β = 93.086(4)<br>γ = 90	a = 34.096(2)<br>b = 27.800(2)<br>c = 4.3397(3)<br>α = 90<br>β = 90<br>γ = 90
Unit cell volume [Å^3]	2110.0(8)	1124.7(3)	2652.2(3)	2164.2(4)	2286.2(4)	4113.5(5)
Formula per unit cell	8	4	8	4	4	8
Calculated density [g/cm^3]	1.431	1.451	1.404	1.380	1.347	1.487
F(000)	944	504	1184	936	968	1904
Absorption coefficient [mm^{-1}]	0.100	0.323	0.106	0.091	0.088	0.099
Temperature [K]	173(2)	173(2)	173(2)	173(2)	173(2)	173(2)
Radiation wavelength [Å]	0.71073 (Mo-K_α)	0.71073 (Mo-K_α)	0.71073 (Mo-K_α)	0.71073 (Mo-K_α)	0.71073 (Mo-K_α)	0.71073 (Mo-K_α)
Diffractometer	Bruker D8 Venture	Bruker D8 Venture	Bruker D8 Venture	Bruker D8 Venture	Bruker D8 Venture	Bruker D8 Venture
θ range [°]	$3.232 \le \theta \le 24.995$	$3.648 \le \theta \le 27.498$	$3.614 \le \theta \le 24.994$	$3.256 \le \theta \le 24.994$	$3.187 \le \theta \le 24.997$	$3.165 \le \theta \le 27.499$
Index ranges	$-30 \le h \le 29$<br>$-7 \le k \le 7$<br>$-16 \le l \le 16$	$-5 \le h \le 5$<br>$-22 \le k \le 22$<br>$-19 \le l \le 18$	$-40 \le h \le 40$<br>$-5 \le k \le 5$<br>$-20 \le l \le 20$	$-4 \le h \le 4$<br>$-22 \le k \le 22$<br>$-33 \le l \le 32$	$-8 \le h \le 8$<br>$-23 \le k \le 23$<br>$-19 \le l \le 19$	$-44 \le h \le 44$<br>$-32 \le k \le 36$<br>$-5 \le l \le 5$
Total number of reflections	12532	19157	14196	22815	37980	48761
Independent reflections [$I \ge 2\sigma(I)$ / all data]	1512 / 1799	4507 / 5139	2006 / 2335	2457 / 3804	3116 / 4020	5832 / 9230
R_σ / R_{int}	0.0398 / 0.0611	0.0427 / 0.0420	0.0348 / 0.0601	0.0694 / 0.1056	0.0310 / 0.0614	0.0591 / 0.0688
Parameters	154	307	213	156	316	297
Restraints	0	1	0	0	0	1
R-values [$I \ge 2\sigma(I)$]	R_1 = 0.0616<br>wR_2 = 0.1531	R_1 = 0.0431<br>wR_2 = 0.0979	R_1 = 0.0792<br>wR_2 = 0.1728	R_1 = 0.0748<br>wR_2 = 0.1669	R_1 = 0.0449<br>wR_2 = 0.1190	R_1 = 0.0588<br>wR_2 = 0.1466
R-values (all data)	R_1 = 0.0737<br>wR_2 = 0.1586	R_1 = 0.0526<br>wR_2 = 0.1013	R_1 = 0.0908<br>wR_2 = 0.1776	R_1 = 0.1230<br>wR_2 = 0.1805	R_1 = 0.0617<br>wR_2 = 0.1260	R_1 = 0.1079<br>wR_2 = 0.1640
Goodness of fit	1.166	1.048	1.173	1.135	1.102	1.041
Max. / min. residual electron density [e/Å^3]	0.367 / -0.218	0.245 / -0.216	0.315 / -0.275	0.232 / -0.180	0.154 / -0.180	0.262 / -0.283
CCDC	2536406	2536407	2536408	2536412	2536414	2536416

Table S2. Selected crystallographic data of compounds **11**, **12**, **13i**, **13** and **14**.

Formula	$C_{22}H_{16}N_{10}O$ (**11**)	$C_{12}H_8N_6$ (**12**)	$C_{22}H_{18}N_{12}$ (**13i**)	$C_{22}H_{16}N_{12}$ (**13**)	$C_{17}H_{13}N_7$ (**14**)
Molar mass [g/mol]	436.45	236.24	450.48	448.47	315.34
Crystal system	tetragonal	orthorhombic	monoclinic	monoclinic	orthorhombic
Space group	$P4_2/n$ (no. 14)	$P2_12_12$ (no. 18)	$P2_1/c$ (no. 14)	$P2_1/c$ (no. 14)	$P2_12_12_1$ (no. 19)
Lattice parameters [Å; °]	a = 30.836(6)<br>b = 30.836(6)<br>c = 4.4060(9)<br>α = 90<br>β = 90<br>γ = 90	a = 13.4219(5)<br>b = 13.9526(5)<br>c = 9.2208(4)<br>α = 90<br>β = 90<br>γ = 90	a = 14.0791(9)<br>b = 5.5703(4)<br>c = 13.4690(9)<br>α = 90<br>β = 90.097(2)<br>γ = 90	a = 14.9622(4)<br>b = 4.35450(10)<br>c = 17.1967(5)<br>α = 90<br>β = 114.0730(10)<br>γ = 90	a = 4.0543(3)<br>b = 16.9370(10)<br>c = 22.2218(13)<br>α = 90<br>β = 90<br>γ = 90
Unit cell volume [Å^3]	4189.4(17)	1726.78(12)	1056.30(12)	1022.97(5)	1525.92(17)
Formula per unit cell	8	6	2	2	4
Calculated density [g/cm^3]	1.384	1.363	1.416	1.456	1.373
F(000)	1808	732	468	464	656
Absorption coefficient [mm^{-1}]	0.094	0.091	0.095	0.097	0.089
Temperature [K]	173(2)	173(2)	173(2)	173(2)	173(2)
Radiation wavelength [Å]	0.71073 (Mo-K_α)	0.71073 (Mo-K_α)	0.71073 (Mo-K_α)	0.71073 (Mo-K_α)	0.71073 (Mo-K_α)
Diffractometer	Bruker D8 Venture	Bruker D8 Venture	Bruker D8 Venture	Bruker D8 Venture	Bruker D8 Venture
θ range [°]	$3.369 \le \theta \le 24.970$	$3.291 \le \theta \le 27.484$	$3.351 \le \theta \le 24.995$	$3.481 \le \theta \le 27.494$	$3.654 \le \theta \le 25.000$
Index ranges	$-36 \le h \le 36$<br>$-36 \le k \le 36$<br>$-5 \le l \le 5$	$-16 \le h \le 17$<br>$-18 \le k \le 18$<br>$-11 \le l \le 11$	$-16 \le h \le 16$<br>$-6 \le k \le 6$<br>$-15 \le l \le 16$	$-19 \le h \le 19$<br>$-5 \le k \le 5$<br>$-22 \le l \le 22$	$-4 \le h \le 4$<br>$-20 \le k \le 20$<br>$-26 \le l \le 26$
Total number of reflections	41136	30932	14745	17483	23785
Independent reflections [$I \ge 2\sigma(I)$ / all data]	2783 / 3667	3098 / 3871	2900 / 3745	2045 / 2350	2454 / 2664
R_σ / R_{int}	0.0612 / 0.1181	0.0319 / 0.0530	0.0261 / 0.0449	0.0229 / 0.0352	0.0275 / 0.0526
Parameters	298	246	154	154	217
Restraints	0	0	0	0	0
R-values [$I \ge 2\sigma(I)$]	R_1 = 0.0843<br>wR_2 = 0.1585	R_1 = 0.0542<br>wR_2 = 0.1351	R_1 = 0.0395<br>wR_2 = 0.1068	R_1 = 0.0420<br>wR_2 = 0.1142	R_1 = 0.0392<br>wR_2 = 0.0922
R-values (all data)	R_1 = 0.1107<br>wR_2 = 0.1665	R_1 = 0.0726<br>wR_2 = 0.1426	R_1 = 0.0477<br>wR_2 = 0.1101	R_1 = 0.0478<br>wR_2 = 0.1169	R_1 = 0.0446<br>wR_2 = 0.0942
Goodness of fit	1.189	1.049	1.151	1.078	1.170
Max. / min. residual electron density [e/Å^3]	0.247 / -0.271	0.491 / -0.335	0.148 / -0.193	0.294 / -0.226	0.156 / -0.196
CCDC	2536417	2536418	2536419	2536420	2536422

Table S3. Selected crystallographic data of compounds **15·H$_2$O**, **16·1.5H$_2$O**, **17**, **18·2H$_2$O**, **19** and **20·3H$_2$O**.

Formula	$C_7H_{13}N_{11}O$ (**15·H$_2$O**)	$C_{16}H_{32}N_{22}O_3$ (**16·1.5H$_2$O**)	$C_7H_8N_{12}$ (**17**)	$C_9H_{16}N_{16}O_2$ (**18·2H$_2$O**)	$C_6H_8N_{10}O$ (**19**)	$C_{16}H_{32}Cl_2CuN_{22}O_{11}$ (**20·3H$_2$O**)

Molar mass [g/mol]	267.28	580.63	260.25	380.38	236.22	843.07
Crystal system	monoclinic	triclinic	monoclinic	orthorhombic	monoclinic	monoclinic
Space group	$P2_1/c$ (no. 14)	$P-1$ (no. 2)	$P2_1/c$ (no. 14)	*Pbcn* (no. 60)	$C2/c$ (no. 15)	$P2_1$ (no. 4)
Lattice parameters [Å; °]	a = 10.5881(3)<br>b = 7.4645(2)<br>c = 14.4251(5)<br>α = 90<br>β = 106.0880(10)<br>γ = 90	a = 10.4634(8)<br>b = 11.6151(10)<br>c = 12.1836(8)<br>α = 99.564(3)<br>β = 112.955(2)<br>γ = 96.966(3)	a = 10.7898(3)<br>b = 6.83110(10)<br>c = 14.7353(3)<br>α = 90<br>β = 107.953(3)<br>γ = 90	a = 13.4409(4)<br>b = 12.2609(3)<br>c = 9.3660(3)<br>α = 90<br>β = 90<br>γ = 90	a = 12.0038(8)<br>b = 5.8593(3)<br>c = 13.3912(8)<br>α = 90<br>β = 94.183(2)<br>γ = 90	a = 7.72300(10)<br>b = 24.2844(3)<br>c = 9.5072(2)<br>α = 90<br>β = 112.619(2)<br>γ = 90
Unit cell volume [$Å^3$]	1095.44(6)	1315.97(18)	1033.20(4)	1543.49(8)	939.35(10)	1645.91(5)
Formula per unit cell	4	2	4	4	4	2
Calculated density [g/cm^3]	1.621	1.465	1.673	1.637	1.670	1.701
F(000)	560	612	536	792	488	866
Absorption coefficient [mm^{-1}]	0.123	0.111	1.049	0.128	0.129	3.227
Temperature [K]	173(2)	173(2)	173(2)	173(2)	173(2)	173(2)
Radiation wavelength [Å]	0.71073 (Mo-K_α)	0.71073 (Mo-K_α)	1.54184 (Cu-K_α)	0.71073 (Mo-K_α)	0.71073 (Mo-K_α)	1.54184 (Cu-K_α)
Diffractometer	Bruker D8 Venture	Bruker D8 Venture	Rigaku XtaLAB Synergy	Bruker D8 Venture	Bruker D8 Venture	Rigaku XtaLAB Synergy
θ range [°]	$3.271 \leq \theta \leq 27.498$	$3.397 \leq \theta \leq 27.499$	$4.307 \leq \theta \leq 74.450$	$3.323 \leq \theta \leq 27.500$	$3.403 \leq \theta \leq 27.497$	$3.640 \leq \theta \leq 74.102$
Index ranges	$-13 \leq h \leq 13$<br>$-9 \leq k \leq 9$<br>$-18 \leq l \leq 18$	$-12 \leq h \leq 13$<br>$-15 \leq k \leq 15$<br>$-15 \leq l \leq 15$	$-13 \leq h \leq 13$<br>$-8 \leq k \leq 8$<br>$-9 \leq l \leq 17$	$-16 \leq h \leq 17$<br>$-15 \leq k \leq 15$<br>$-12 \leq l \leq 12$	$-15 \leq h \leq 15$<br>$-7 \leq k \leq 7$<br>$-17 \leq l \leq 16$	$-9 \leq h \leq 9$<br>$-30 \leq k \leq 28$<br>$-11 \leq l \leq 11$
Total number of reflections	20137	23596	9397	25785	5152	33650
Independent reflections [$I \geq 2\sigma(I)$ / all data]	2302 / 2513	4358 / 6020	1914 / 2061	1563 / 1772	889 / 1081	6269 / 6534
R_σ / R_{int}	0.0176 / 0.0281	0.0541 / 0.0494	0.0200 / 0.0165	0.0173 / 0.0405	0.0285 / 0.0366	0.0381 / 0.0389
Parameters	224	498	204	1772	94	521
Restraints	1	15	0	0	0	7
R-values [$I \geq 2\sigma(I)$]	R_1 = 0.0430<br>wR_2 = 0.1225	R_1 = 0.0688<br>wR_2 = 0.1265	R_1 = 0.0429<br>wR_2 = 0.1091	R_1 = 0.0446<br>wR_2 = 0.1252	R_1 = 0.0480<br>wR_2 = 0.1069	R_1 = 0.0414<br>wR_2 = 0.1078
R-values (all data)	R_1 = 0.0459<br>wR_2 = 0.1243	R_1 = 0.976<br>wR_2 = 0.1337	R_1 = 0.0476<br>wR_2 = 0.1112	R_1 = 0.0510<br>wR_2 = 0.1295	R_1 = 0.0617<br>wR_2 = 0.1115	R_1 = 0.0433<br>wR_2 = 0.1090
Goodness of fit	1.094	1.147	1.259	1.052	1.130	1.042
Max. / min. residual electron density [$e/Å^3$]	0.347 / -0.462	0.276 / -0.379	0.206 / -0.205	0.770 / -0.234	0.223 / -0.215	1.123 / -0.490
CCDC	2536424	2536432	2536433	2536434	2536436	2536437

Table S4. Fractional atomic coordinates and isotropic atomic displacement parameters U_{iso} of all independent atoms of **4**.

Atom label	x	y	z	U_{iso} [$Å^2$]
O1	0.77338(7)	0.4519(3)	0.57307(14)	0.0306(5)
N1	0.69054(8)	0.4405(4)	0.50341(15)	0.0227(5)

N2	0.71580(9)	0.7305(4)	0.61094(15)	0.0251(6)
N3	0.62801(9)	0.7180(4)	0.53585(15)	0.0236(5)
N5	0.65347(9)	1.0004(4)	0.64126(15)	0.0261(6)
N6	0.60424(8)	0.4228(4)	0.43420(15)	0.0232(5)
C1	0.72894(10)	0.5401(5)	0.56451(18)	0.0238(6)
C2	0.66733(11)	0.8051(5)	0.59380(17)	0.0233(6)
C3	0.64183(10)	0.5312(4)	0.49263(17)	0.0212(6)
C4	0.68601(13)	1.1137(5)	0.70806(19)	0.0312(7)
C5	0.65858(13)	1.2907(5)	0.7401(2)	0.0359(8)
C6	0.60791(13)	1.2910(5)	0.6925(2)	0.0369(8)
C7	0.60500(12)	1.1123(5)	0.6321(2)	0.0305(7)
C8	0.55130(11)	0.4910(5)	0.4247(2)	0.0324(7)
C9	0.52454(12)	0.3343(6)	0.3719(2)	0.0372(8)
C10	0.56090(12)	0.1644(5)	0.3468(2)	0.0339(7)
C11	0.60918(11)	0.2202(5)	0.38586(19)	0.0267(7)
H1	0.698268	0.317904	0.471837	0.027
H2	0.721295	1.073441	0.727625	0.037
H3	0.671073	1.396600	0.786452	0.043
H4	0.580569	1.397975	0.701206	0.044
H5	0.575472	1.071483	0.591291	0.037
H6	0.536720	0.623521	0.450778	0.039
H7	0.487713	0.337441	0.354423	0.045
H8	0.552788	0.034698	0.309364	0.041
H9	0.640908	0.135685	0.381081	0.032

Table S5. Anisotropic atomic displacement parameters U_{ij} of all independent atoms of **4**.

Atom label	U_{11} [Å^2]	U_{22} [Å^2]	U_{33} [Å^2]	U_{23} [Å^2]	U_{13} [Å^2]	U_{12} [Å^2]
O1	0.0254(11)	0.0344(11)	0.0317(11)	-0.0081(9)	-0.0021(8)	0.0080(9)
N1	0.0244(12)	0.0231(12)	0.0206(11)	-0.0014(9)	0.0019(9)	0.0042(10)
N2	0.0273(13)	0.0239(12)	0.0242(12)	-0.0008(10)	0.0002(9)	0.0040(10)
N3	0.0265(12)	0.0223(12)	0.0222(11)	0.0041(9)	0.0031(9)	0.0035(10)

N5	0.0328(13)	0.0221(12)	0.0235(12)	0.0018(9)	0.0041(10)	0.0056(10)
N6	0.0225(12)	0.0282(12)	0.0188(11)	0.0015(9)	0.0016(9)	0.0052(10)
C1	0.0253(15)	0.0267(15)	0.0194(13)	0.0012(11)	0.0017(11)	0.0044(12)
C2	0.0292(15)	0.0242(14)	0.0169(13)	0.0044(11)	0.0043(11)	0.0011(12)
C3	0.0244(14)	0.0241(14)	0.0153(12)	0.0051(11)	0.0037(10)	0.0031(11)
C4	0.0420(18)	0.0301(16)	0.0217(14)	-0.0003(12)	0.0020(12)	0.0012(13)
C5	0.056(2)	0.0293(16)	0.0234(14)	-0.0045(12)	0.0083(14)	0.0054(15)
C6	0.0454(19)	0.0323(17)	0.0347(16)	0.0048(13)	0.0184(14)	0.0142(14)
C7	0.0347(17)	0.0282(16)	0.0294(16)	0.0049(12)	0.0091(12)	0.0089(13)
C8	0.0260(15)	0.0436(18)	0.0276(15)	-0.0034(13)	0.0003(12)	0.0095(13)
C9	0.0260(16)	0.052(2)	0.0327(16)	-0.0071(15)	-0.0063(13)	0.0047(14)
C10	0.0371(17)	0.0377(17)	0.0264(15)	-0.0064(13)	-0.0032(13)	0.0033(14)
C11	0.0255(15)	0.0310(16)	0.0236(14)	-0.0027(12)	0.0002(11)	0.0070(12)

Table S6. Fractional atomic coordinates and isotropic atomic displacement parameters U_{iso} of all independent atoms of **5**.

Atom label	*x*	*y*	*z*	U_{iso} [Å^2]	Atom label	*x*	*y*	*z*	U_{iso} [Å^2]
Cl1A	0.2197(3)	0.51183(6)	0.77908(6)	0.0462(3)	C3B	0.3266(7)	0.6568(2)	0.4460(2)	0.0268(7)
Cl1B	0.2275(4)	0.44995(6)	0.52034(10)	0.0704(4)	C4B	-0.3047(8)	0.6689(3)	0.6824(2)	0.0370(9)
N1A	0.1039(6)	0.40183(16)	0.88844(19)	0.0270(6)	C5B	-0.4449(9)	0.7278(3)	0.7200(3)	0.0442(10)
N2A	0.3554(6)	0.38792(15)	1.03977(19)	0.0229(6)	C6B	-0.3642(9)	0.7970(3)	0.6773(3)	0.0434(10)
N3A	0.4814(6)	0.49240(16)	0.94463(19)	0.0275(6)	C7B	-0.1766(8)	0.7785(2)	0.6135(3)	0.0341(8)
N4A	-0.0193(6)	0.30322(16)	0.98225(18)	0.0238(6)	C8B	0.4975(8)	0.7744(2)	0.3745(2)	0.0308(8)
N5A	0.7263(6)	0.47594(15)	1.09071(19)	0.0244(6)	C9B	0.6781(9)	0.7893(3)	0.3079(3)	0.0400(9)
N1B	0.0414(7)	0.58262(18)	0.5704(2)	0.0348(7)	C10B	0.7758(8)	0.7176(3)	0.2747(3)	0.0405(9)
N2B	0.1706(6)	0.69971(16)	0.49926(18)	0.0245(6)	C11B	0.6570(8)	0.6605(2)	0.3215(2)	0.0339(8)
N3B	0.3501(7)	0.58020(18)	0.4475(2)	0.0354(7)	H1A	-0.291020	0.291442	0.856488	0.034
N4B	-0.1391(6)	0.69961(17)	0.61573(18)	0.0273(6)	H2A	-0.508828	0.177271	0.925075	0.041
N5B	0.4816(6)	0.69522(17)	0.38373(19)	0.0275(6)	H3A	-0.246731	0.161347	1.087197	0.040
C1A	0.2766(8)	0.46347(19)	0.8834(2)	0.0292(7)	H4A	0.126598	0.266661	1.116211	0.034
C2A	0.1563(7)	0.36715(18)	0.9698(2)	0.0218(6)	H5A	0.705754	0.396299	1.196890	0.036
C3A	0.5107(7)	0.45134(19)	1.0226(2)	0.0226(6)	H6A	1.099133	0.475058	1.284994	0.043
C4A	-0.2363(8)	0.2722(2)	0.9167(2)	0.0287(7)	H7A	1.224050	0.586837	1.185042	0.043
C5A	-0.3547(8)	0.2097(2)	0.9542(3)	0.0345(8)	H8A	0.903551	0.575792	1.036463	0.035
C6A	-0.2079(8)	0.2008(2)	1.0452(3)	0.0335(8)	H1B	-0.316581	0.616071	0.698386	0.044
C7A	-0.0036(8)	0.2584(2)	1.0612(2)	0.0284(7)	H2B	-0.575162	0.723790	0.767000	0.053
C8A	0.7946(8)	0.4418(2)	1.1761(2)	0.0300(7)	H3B	-0.429763	0.847353	0.690899	0.052
C9A	1.0100(8)	0.4850(2)	1.2242(3)	0.0356(8)	H4B	-0.087537	0.813425	0.574497	0.041
C10A	1.0797(8)	0.5475(2)	1.1683(3)	0.0357(8)	H5B	0.399627	0.811468	0.408573	0.037
C11A	0.9046(7)	0.54151(19)	1.0869(3)	0.0292(7)	H6B	0.730303	0.838803	0.287189	0.048
C1B	0.2028(9)	0.5487(2)	0.5112(3)	0.0374(9)	H7B	0.903393	0.711165	0.227388	0.049
C2B	0.0333(7)	0.6592(2)	0.5593(2)	0.0251(7)	H8B	0.686594	0.606850	0.313698	0.041

Table S7. Anisotropic atomic displacement parameters U_{ij} of all independent atoms of **5**.

Atom label	U_{11} [Å^2]	U_{22} [Å^2]	U_{33} [Å^2]	U_{23} [Å^2]	U_{13} [Å^2]	U_{12} [Å^2]
Cl1A	0.0649(6)	0.0409(5)	0.0315(4)	0.0134(4)	-0.0002(4)	-0.0030(5)
Cl1B	0.1165(11)	0.0223(4)	0.0727(8)	0.0031(5)	0.0115(8)	0.0030(6)
N1A	0.0280(14)	0.0256(14)	0.0274(15)	0.0016(12)	0.0029(11)	0.0038(12)
N2A	0.0201(13)	0.0229(13)	0.0262(14)	-0.0006(11)	0.0047(11)	0.0024(11)
N3A	0.0298(14)	0.0256(15)	0.0282(14)	0.0017(11)	0.0081(11)	0.0001(11)
N4A	0.0210(13)	0.0234(13)	0.0273(14)	-0.0016(11)	0.0034(11)	0.0020(11)
N5A	0.0227(13)	0.0232(13)	0.0282(14)	-0.0012(11)	0.0071(11)	0.0008(11)
N1B	0.0428(18)	0.0280(15)	0.0321(16)	0.0036(13)	-0.0026(13)	-0.0058(14)
N2B	0.0228(13)	0.0255(14)	0.0239(13)	-0.0009(11)	-0.0026(11)	-0.0020(11)
N3B	0.0458(18)	0.0271(15)	0.0319(16)	-0.0027(13)	-0.0016(13)	0.0057(14)
N4B	0.0229(13)	0.0357(15)	0.0225(14)	0.0022(12)	-0.0003(11)	-0.0061(12)
N5B	0.0241(14)	0.0348(15)	0.0234(14)	-0.0033(12)	0.0028(11)	-0.0008(12)
C1A	0.0324(18)	0.0290(19)	0.0270(16)	0.0064(13)	0.0078(14)	0.0072(14)
C2A	0.0178(14)	0.0209(15)	0.0272(15)	-0.0016(12)	0.0051(12)	0.0046(12)
C3A	0.0186(14)	0.0239(14)	0.0266(16)	-0.0044(13)	0.0082(12)	0.0044(13)
C4A	0.0247(16)	0.0324(18)	0.0285(17)	-0.0087(14)	0.0019(14)	0.0040(14)
C5A	0.0283(17)	0.0338(19)	0.043(2)	-0.0143(16)	0.0099(16)	-0.0060(15)
C6A	0.0357(19)	0.0279(18)	0.0389(19)	0.0001(15)	0.0131(16)	-0.0017(15)
C7A	0.0310(18)	0.0283(17)	0.0258(16)	0.0020(14)	0.0033(14)	0.0032(14)
C8A	0.0297(17)	0.0305(18)	0.0306(17)	0.0011(15)	0.0060(14)	0.0009(15)
C9A	0.0339(19)	0.040(2)	0.0321(18)	-0.0063(15)	-0.0004(15)	0.0055(16)
C10A	0.0305(18)	0.0309(18)	0.046(2)	-0.0101(16)	0.0053(16)	0.0001(16)
C11A	0.0239(16)	0.0253(16)	0.0394(19)	-0.0036(14)	0.0084(14)	0.0005(14)
C1B	0.050(2)	0.0203(16)	0.039(2)	0.0008(15)	-0.0052(18)	-0.0003(16)
C2B	0.0226(16)	0.0311(17)	0.0197(15)	0.0010(13)	-0.0057(12)	-0.0030(13)
C3B	0.0239(16)	0.0309(17)	0.0234(16)	-0.0032(13)	-0.0073(13)	-0.0007(14)
C4B	0.0315(19)	0.055(2)	0.0232(17)	0.0097(16)	-0.0014(14)	-0.0116(18)
C5B	0.0317(18)	0.079(3)	0.0221(18)	-0.0051(19)	0.0051(15)	-0.010(2)
C6B	0.038(2)	0.052(2)	0.041(2)	-0.0168(19)	0.0093(17)	-0.0039(19)
C7B	0.0323(19)	0.037(2)	0.0339(18)	-0.0064(16)	0.0068(15)	-0.0034(16)
C8B	0.0319(18)	0.0312(18)	0.0291(17)	0.0007(15)	0.0032(14)	-0.0015(15)
C9B	0.039(2)	0.051(3)	0.0296(18)	0.0055(17)	0.0016(16)	-0.0112(18)
C10B	0.0294(18)	0.065(3)	0.0268(18)	-0.0046(18)	0.0038(15)	-0.0015(19)
C11B	0.0291(18)	0.043(2)	0.0290(18)	-0.0105(16)	0.0016(14)	0.0033(16)

Table S8. Fractional atomic coordinates and isotropic atomic displacement parameters U_{iso} of all independent atoms of **6·2H$_2$O**.

Atom label	*x*	*y*	*z*	U_{iso} [Å^2]
O1	0.70848(7)	0.4921(6)	0.55242(16)	0.0185(6)
O2	0.68567(10)	0.0348(8)	0.4163(2)	0.0345(8)
O3	0.72877(9)	0.6770(7)	0.74929(18)	0.0241(7)
N1	0.66765(9)	0.7678(7)	0.60767(18)	0.0175(7)
N2	0.64291(9)	0.4443(7)	0.49756(19)	0.0193(7)
N3	0.59838(9)	0.7378(7)	0.5513(2)	0.0210(8)
N4	0.57491(9)	0.4203(8)	0.4462(2)	0.0213(8)
N5	0.62293(9)	1.0415(7)	0.65874(19)	0.0193(7)
N6	0.75522(11)	0.9908(8)	0.5846(2)	0.0181(7)
C1	0.67329(11)	0.5667(8)	0.5517(2)	0.0157(8)
C2	0.60747(11)	0.5381(9)	0.5010(2)	0.0193(9)
C3	0.63008(11)	0.8382(8)	0.6031(2)	0.0172(8)
C4	0.57592(12)	0.2101(9)	0.3888(2)	0.0252(9)
C5	0.53816(13)	0.1560(11)	0.3479(3)	0.0316(11)
C6	0.51315(13)	0.3370(11)	0.3806(3)	0.0362(12)
C7	0.53577(12)	0.4989(11)	0.4406(3)	0.0328(11)
C8	0.58585(13)	1.1249(10)	0.6686(3)	0.0284(10)
C9	0.59111(13)	1.3172(10)	0.7299(3)	0.0312(10)
C10	0.63226(13)	1.3598(9)	0.7600(2)	0.0271(10)
C11	0.65114(12)	1.1887(9)	0.7155(2)	0.0234(9)
H1	0.598996	0.119157	0.379568	0.030
H2	0.530115	0.020934	0.305016	0.038
H3	0.485154	0.344521	0.363506	0.043
H4	0.526558	0.639411	0.472800	0.039
H5	0.561154	1.058068	0.637522	0.034
H6	0.570686	1.408823	0.749590	0.037
H7	0.644377	1.484371	0.803096	0.033
H8	0.678966	1.172.816	0.722213	0.028
H9	0.7430(14)	0.814(12)	0.585(3)	0.036(14)
H10	0.7361(13)	1.139(11)	0.569(3)	0.025(12)
H11	0.7663(14)	1.029(11)	0.632(3)	0.034(14)
H12	0.7726(15)	1.006(12)	0.548(3)	0.046(15)
H13	0.6711(16)	0.147(14)	0.447(4)	0.056(17)
H14	0.6732(18)	-0.043(14)	0.368(4)	0.063(19)
H15	0.7132(17)	0.706(13)	0.706(4)	0.050(17)
H16	0.7425(18)	0.833(15)	0.755(4)	0.057(19)

Table S9. Anisotropic atomic displacement parameters U_{ij} of all independent atoms of **6·2H$_2$O**.

Atom label	U_{11} [Å^2]	U_{22} [Å^2]	U_{33} [Å^2]	U_{23} [Å^2]	U_{13} [Å^2]	U_{12} [Å^2]
O1	0.0171(14)	0.0185(14)	0.0203(13)	-0.0018(12)	0.0047(10)	0.0011(11)
O2	0.0311(17)	0.0357(19)	0.0360(19)	-0.0129(16)	0.0059(15)	0.0005(15)
O3	0.0270(17)	0.0236(16)	0.0185(15)	0.0027(13)	-0.0017(13)	-0.0002(14)
N1	0.0191(17)	0.0192(17)	0.0144(15)	0.0003(14)	0.0044(13)	0.0007(14)
N2	0.0201(17)	0.0171(17)	0.0210(17)	-0.0008(14)	0.0054(13)	-0.0025(14)
N3	0.0181(17)	0.0221(18)	0.0236(17)	0.0000(15)	0.0062(14)	0.0004(14)
N4	0.0162(17)	0.0243(19)	0.0218(17)	-0.0003(15)	0.0010(13)	-0.0040(14)
N5	0.0207(17)	0.0170(17)	0.0206(17)	0.0012(14)	0.0057(13)	0.0008(14)
N6	0.0220(18)	0.0159(18)	0.0175(18)	-0.0008(15)	0.0068(15)	-0.0014(16)
C1	0.019(2)	0.0138(19)	0.0137(17)	0.0051(15)	0.0031(15)	0.0013(16)
C2	0.020(2)	0.020(2)	0.0167(19)	0.0041(17)	0.0021(15)	-0.0026(17)
C3	0.021(2)	0.0146(19)	0.0161(18)	0.0038(16)	0.0058(15)	-0.0002(16)
C4	0.026(2)	0.027(2)	0.022(2)	-0.0052(19)	0.0042(17)	-0.0064(19)
C5	0.031(2)	0.036(3)	0.024(2)	-0.004(2)	-0.0011(18)	-0.010(2)
C6	0.021(2)	0.039(3)	0.043(3)	-0.002(2)	-0.003(2)	-0.004(2)
C7	0.019(2)	0.032(3)	0.046(3)	-0.004(2)	0.0045(19)	0.000(2)
C8	0.023(2)	0.028(2)	0.038(2)	-0.006(2)	0.0142(19)	0.0002(19)
C9	0.034(2)	0.026(2)	0.038(3)	0.000(2)	0.019(2)	0.006(2)
C10	0.039(3)	0.022(2)	0.020(2)	-0.0020(19)	0.0059(18)	0.006(2)
C11	0.026(2)	0.020(2)	0.022(2)	0.0004(18)	0.0017(17)	-0.0022(18)

Table S10. Fractional atomic coordinates and isotropic atomic displacement parameters U_{iso} of all independent atoms of **7**.

Atom label	*x*	*y*	*z*	U_{iso} [Å^2]	Atom label	*x*	*y*	*z*	U_{iso} [Å^2]
N1	0.5730(8)	0.36381(17)	0.43389(10)	0.0453(8)	C17	0.3511(10)	0.3666(2)	0.19349(14)	0.0470(10)
N2	0.6627(8)	0.25208(16)	0.40545(10)	0.0387(8)	C18	0.5077(9)	0.4075(2)	0.22527(13)	0.0419(10)
N3	0.4429(8)	0.15612(17)	0.44721(11)	0.0416(8)	C19	1.1801(10)	0.5801(2)	0.31419(17)	0.0529(11)
N4	0.3533(8)	0.27108(18)	0.47539(10)	0.0424(8)	C20	1.3483(11)	0.6374(2)	0.3302(2)	0.0655(14)
N5	0.5320(7)	0.36699(15)	0.35106(10)	0.0383(7)	C21	1.3464(11)	0.6364(2)	0.3803(2)	0.0666(14)
N6	0.7990(7)	0.45937(15)	0.31007(10)	0.0354(7)	C22	1.1826(11)	0.5789(2)	0.39476(17)	0.0557(12)
N7	0.8308(8)	0.45350(16)	0.39481(11)	0.0393(8)	C23	0.5475(13)	0.4068(2)	0.47642(14)	0.0637(13)
N8	0.7553(7)	0.14065(16)	0.37928(11)	0.0402(8)	H1	0.949345	0.208847	0.329169	0.056
N9	0.1485(8)	0.17600(19)	0.51560(11)	0.0474(9)	H2	1.154916	0.104524	0.28893	0.068
N10	0.4943(7)	0.37455(15)	0.26956(10)	0.0350(7)	H3	0.984094	-0.001388	0.337396	0.068
N11	1.0790(8)	0.54363(16)	0.35379(12)	0.0426(8)	H4	0.666863	0.040993	0.406056	0.063
C1	0.5211(9)	0.2926(2)	0.43766(13)	0.0389(9)	H5	0.147926	0.068373	0.505289	0.07
C2	0.6094(9)	0.1853(2)	0.41178(13)	0.0379(9)	H6	-0.167709	0.062532	0.579412	0.081
C3	0.3251(9)	0.2021(2)	0.47721(13)	0.0411(10)	H7	-0.243666	0.184094	0.610493	0.082
C4	0.6536(9)	0.3953(2)	0.39123(13)	0.0378(9)	H8	0.028836	0.265732	0.554486	0.067
C5	0.6183(8)	0.40124(19)	0.31247(12)	0.0338(8)	H9	0.287042	0.278606	0.288171	0.053
C6	0.8926(9)	0.48271(19)	0.35288(14)	0.0388(9)	H10	0.127559	0.268091	0.20277	0.057
C7	0.9206(10)	0.1622(2)	0.33929(14)	0.0469(10)	H11	0.318451	0.376366	0.160625	0.056
C8	1.0326(11)	0.1053(2)	0.31745(16)	0.0564(12)	H12	0.609458	0.451036	0.218985	0.05
C9	0.9358(11)	0.0457(2)	0.34458(17)	0.0567(12)	H13	1.13963	0.56747	0.281935	0.063
C10	0.7639(10)	0.0688(2)	0.38214(16)	0.0522(11)	H14	1.448752	0.671916	0.310997	0.079
C11	0.0836(10)	0.1067(3)	0.52430(16)	0.0583(12)	H15	1.444466	0.670521	0.400611	0.08
C12	-0.0884(11)	0.1037(3)	0.56492(17)	0.0671(14)	H16	1.145511	0.565288	0.426642	0.067
C13	-0.1313(11)	0.1717(3)	0.58237(17)	0.0687(15)	H17	0.314372	0.407844	0.486469	0.096
C14	0.0173(10)	0.2166(3)	0.55173(15)	0.0561(12)	H18	0.621497	0.454158	0.469345	0.096
C15	0.3301(9)	0.3116(2)	0.26397(14)	0.0439(10)	H19	0.689603	0.387316	0.501962	0.096
C16	0.2425(10)	0.3061(2)	0.21717(14)	0.0476(11)					

Table S11. Anisotropic atomic displacement parameters U_{ij} of all independent atoms of **7**.

Atom label	U_{11} [Å^2]	U_{22} [Å^2]	U_{33} [Å^2]	U_{23} [Å^2]	U_{13} [Å^2]	U_{12} [Å^2]
N1	0.060(2)	0.047(2)	0.0285(17)	-0.0040(14)	0.0018(15)	-0.0015(17)
N2	0.0385(18)	0.041(2)	0.0368(18)	0.0053(15)	-0.0026(15)	-0.0023(15)
N3	0.0346(18)	0.051(2)	0.0388(19)	0.0078(16)	-0.0076(15)	-0.0064(15)
N4	0.0358(18)	0.064(2)	0.0274(17)	0.0089(15)	-0.0019(14)	-0.0030(16)
N5	0.0397(18)	0.0415(18)	0.0337(18)	0.0031(14)	0.0010(14)	0.0005(15)
N6	0.0335(17)	0.0364(18)	0.0363(18)	0.0028(14)	-0.0003(14)	0.0022(14)
N7	0.0417(19)	0.0384(18)	0.0378(18)	0.0006(14)	-0.0022(15)	0.0010(15)
N8	0.0333(18)	0.046(2)	0.0410(19)	0.0018(15)	-0.0086(15)	-0.0046(15)
N9	0.0344(18)	0.070(3)	0.0372(19)	0.0148(18)	-0.0063(15)	-0.0049(17)
N10	0.0318(17)	0.0396(18)	0.0336(17)	-0.0007(14)	0.0026(13)	0.0045(14)
N11	0.0376(18)	0.0331(18)	0.057(2)	0.0011(16)	-0.0056(16)	0.0014(15)
C1	0.036(2)	0.048(2)	0.033(2)	0.0031(18)	-0.0098(18)	-0.0002(19)
C2	0.029(2)	0.048(2)	0.036(2)	0.0045(19)	-0.0113(17)	-0.0039(18)
C3	0.027(2)	0.062(3)	0.033(2)	0.012(2)	-0.0106(17)	-0.0050(19)
C4	0.037(2)	0.042(2)	0.034(2)	-0.0002(17)	0.0009(17)	0.0030(18)
C5	0.0236(18)	0.042(2)	0.035(2)	-0.0006(17)	-0.0004(15)	0.0062(17)
C6	0.029(2)	0.035(2)	0.052(3)	0.0007(18)	-0.0013(18)	0.0057(16)
C7	0.043(2)	0.059(3)	0.038(2)	0.002(2)	-0.0040(19)	-0.004(2)
C8	0.050(3)	0.067(3)	0.052(3)	-0.015(2)	-0.002(2)	0.004(2)
C9	0.051(3)	0.045(3)	0.074(3)	-0.007(2)	-0.012(2)	-0.003(2)
C10	0.045(3)	0.045(3)	0.066(3)	-0.001(2)	-0.009(2)	-0.001(2)
C11	0.044(3)	0.072(3)	0.058(3)	0.028(2)	-0.006(2)	-0.011(2)
C12	0.045(3)	0.091(4)	0.065(3)	0.040(3)	-0.003(2)	-0.007(3)
C13	0.038(3)	0.118(5)	0.050(3)	0.037(3)	0.004(2)	0.005(3)
C14	0.035(2)	0.088(3)	0.045(3)	0.012(2)	-0.005(2)	0.006(2)
C15	0.039(2)	0.044(2)	0.049(2)	-0.0010(19)	0.0011(19)	0.0026(18)
C16	0.038(2)	0.052(3)	0.052(3)	-0.017(2)	-0.0102(19)	0.0094(19)
C17	0.043(2)	0.062(3)	0.035(2)	-0.002(2)	-0.0018(18)	0.013(2)
C18	0.039(2)	0.054(2)	0.033(2)	0.0080(18)	0.0014(18)	0.0106(19)
C19	0.037(2)	0.050(3)	0.072(3)	0.012(2)	0.002(2)	0.002(2)
C20	0.038(3)	0.047(3)	0.112(4)	0.014(3)	0.005(3)	-0.003(2)
C21	0.047(3)	0.052(3)	0.100(4)	-0.011(3)	-0.008(3)	-0.003(2)
C22	0.045(2)	0.051(3)	0.070(3)	-0.011(2)	-0.012(2)	-0.005(2)
C23	0.090(4)	0.067(3)	0.035(2)	-0.008(2)	0.011(2)	-0.011(3)

Table S12. Fractional atomic coordinates and isotropic atomic displacement parameters U_{iso} of all independent atoms of **8**.

Atom label	*x*	*y*	*z*	U_{iso} [Å^2]	Atom label	*x*	*y*	*z*	U_{iso} [Å^2]
N1	0.4695(2)	0.78333(8)	0.27011(9)	0.0415(4)	C18	0.6206(3)	0.52546(11)	0.10017(13)	0.0514(5)
N2	0.3972(2)	0.69970(8)	0.36602(9)	0.0416(4)	C19	0.4782(3)	0.71682(12)	-0.08460(12)	0.0510(5)
N3	0.4251(2)	0.72729(9)	0.50962(10)	0.0468(4)	C20	0.4341(3)	0.75842(13)	-0.14829(13)	0.0606(6)
N4	0.4898(2)	0.81009(8)	0.40874(9)	0.0429(4)	C21	0.3776(3)	0.82052(14)	-0.11562(14)	0.0602(6)
N5	0.5414(2)	0.67647(8)	0.21819(9)	0.0401(4)	C22	0.3884(3)	0.81578(12)	-0.03243(13)	0.0512(5)
N6	0.5292(2)	0.66103(8)	0.07199(9)	0.0423(4)	C23	0.4663(4)	0.85688(11)	0.25271(13)	0.0600(6)
N7	0.4528(2)	0.76757(8)	0.12915(9)	0.0404(4)	C24	0.2705(5)	0.88435(15)	0.24329(19)	0.0974(11)
N8	0.3328(2)	0.62101(9)	0.46548(10)	0.0481(4)	H1	0.290244	0.573244	0.351182	0.069
N9	0.5170(2)	0.83520(9)	0.54830(10)	0.0484(4)	H2	0.198318	0.473146	0.430297	0.085
N10	0.5973(2)	0.57245(8)	0.16153(9)	0.0426(4)	H3	0.219942	0.503793	0.581432	0.091
N11	0.4503(2)	0.75162(8)	-0.01236(9)	0.0433(4)	H4	0.327093	0.621490	0.594628	0.075
C1	0.4530(3)	0.76262(10)	0.35153(11)	0.0390(4)	H5	0.500577	0.778220	0.656484	0.072
C2	0.3874(3)	0.68586(10)	0.44590(12)	0.0420(5)	H6	0.591683	0.882519	0.730809	0.083
C3	0.4746(3)	0.78846(11)	0.48567(12)	0.0430(5)	H7	0.633264	0.974447	0.625650	0.080
C4	0.4868(3)	0.73980(10)	0.20362(11)	0.0376(4)	H8	0.567213	0.925397	0.486865	0.067
C5	0.5541(3)	0.63998(10)	0.15007(11)	0.0390(4)	H9	0.604686	0.560627	0.289649	0.061
C6	0.4793(3)	0.72558(10)	0.06716(11)	0.0389(4)	H10	0.665649	0.440128	0.263387	0.074
C7	0.2867(3)	0.57024(12)	0.40960(15)	0.0575(6)	H11	0.671496	0.423613	0.108645	0.074
C8	0.2359(4)	0.51562(13)	0.45282(16)	0.0705(7)	H12	0.615906	0.534227	0.042447	0.062
C9	0.2483(4)	0.53279(14)	0.53710(17)	0.0758(8)	H13	0.520871	0.671576	-0.088555	0.061
C10	0.3070(3)	0.59701(13)	0.54452(14)	0.0629(6)	H14	0.440067	0.747769	-0.205258	0.073
C11	0.5254(3)	0.82068(14)	0.63211(13)	0.0599(6)	H15	0.338506	0.859134	-0.146771	0.072
C12	0.5751(3)	0.87769(15)	0.67254(15)	0.0689(7)	H16	0.358828	0.850326	0.005393	0.061
C13	0.5984(3)	0.92903(14)	0.61393(15)	0.0664(7)	H17	0.531399	0.865580	0.201320	0.072
C14	0.5623(3)	0.90224(12)	0.53804(14)	0.0560(6)	H18	0.535766	0.880879	0.298393	0.072
C15	0.6145(3)	0.54017(11)	0.23703(13)	0.0509(5)	H19	0.275363	0.932877	0.231910	0.146
C16	0.6477(3)	0.47431(12)	0.22261(15)	0.0620(6)	H20	0.201890	0.861418	0.197362	0.146
C17	0.6512(3)	0.46506(12)	0.13626(15)	0.0615(6)	H21	0.206257	0.876716	0.294432	0.146

Table S13. Anisotropic atomic displacement parameters U_{ij} of all independent atoms of **8**.

Atom label	U_{11} [Å^2]	U_{22} [Å^2]	U_{33} [Å^2]	U_{23} [Å^2]	U_{13} [Å^2]	U_{12} [Å^2]
N1	0.0433(19)	0.0242(16)	0.0195(15)	-0.0008(12)	0.0008(13)	-0.0012(14)
N2	0.0297(17)	0.0230(16)	0.0220(16)	0.0040(13)	-0.0019(13)	-0.0009(13)
N3	0.0261(16)	0.0304(16)	0.0218(16)	0.0067(13)	-0.0046(13)	-0.0050(13)
N4	0.0279(16)	0.0325(18)	0.0197(15)	0.0063(13)	-0.0027(13)	-0.0011(13)
N5	0.0282(16)	0.0217(15)	0.0220(16)	0.0015(12)	0.0015(12)	0.0021(13)
N6	0.0237(15)	0.0190(15)	0.0256(16)	0.0029(12)	0.0019(12)	0.0016(12)
N7	0.0291(16)	0.0219(16)	0.0239(16)	-0.0003(13)	-0.0005(12)	0.0012(13)
N8	0.0265(16)	0.0241(16)	0.0260(16)	0.0023(13)	-0.0041(13)	-0.0025(13)
N9	0.0252(16)	0.0377(18)	0.0224(16)	0.0092(14)	-0.0025(13)	-0.0049(14)
N10	0.0230(15)	0.0225(15)	0.0219(15)	0.0001(12)	0.0027(12)	0.0023(12)
N11	0.0251(16)	0.0193(15)	0.0359(18)	0.0006(13)	-0.0024(13)	0.0007(13)
C1	0.0239(18)	0.030(2)	0.0191(18)	0.0038(16)	-0.0056(14)	-0.0015(16)
C2	0.0205(18)	0.0284(19)	0.0204(18)	0.0045(15)	-0.0091(14)	-0.0023(15)
C3	0.0203(18)	0.037(2)	0.0204(18)	0.0064(16)	-0.0089(14)	-0.0040(16)
C4	0.0269(19)	0.0207(18)	0.0224(18)	0.0005(15)	0.0014(14)	0.0048(15)
C5	0.0203(17)	0.0233(18)	0.0214(18)	-0.0005(15)	0.0008(14)	0.0039(15)
C6	0.0217(18)	0.0178(17)	0.029(2)	-0.0008(15)	-0.0008(14)	0.0070(14)
C7	0.033(2)	0.033(2)	0.0241(19)	0.0012(16)	-0.0027(16)	-0.0024(17)
C8	0.035(2)	0.043(2)	0.029(2)	-0.0063(18)	-0.0011(17)	-0.0004(19)
C9	0.037(2)	0.030(2)	0.038(2)	-0.0065(18)	-0.0039(18)	-0.0011(18)
C10	0.035(2)	0.0226(19)	0.038(2)	0.0024(17)	-0.0053(18)	-0.0040(16)
C11	0.031(2)	0.038(2)	0.038(2)	0.0162(18)	-0.0045(17)	-0.0065(18)
C12	0.031(2)	0.054(3)	0.039(2)	0.025(2)	-0.0024(18)	-0.006(2)
C13	0.028(2)	0.063(3)	0.029(2)	0.019(2)	0.0021(17)	0.002(2)
C14	0.025(2)	0.050(2)	0.027(2)	0.0065(18)	-0.0023(16)	0.0005(18)
C15	0.030(2)	0.0214(18)	0.032(2)	-0.0024(16)	0.0041(16)	0.0004(16)
C16	0.028(2)	0.031(2)	0.034(2)	-0.0107(17)	-0.0051(16)	0.0050(16)
C17	0.032(2)	0.039(2)	0.0191(18)	-0.0026(16)	-0.0021(15)	0.0088(17)
C18	0.030(2)	0.031(2)	0.0235(19)	0.0052(16)	0.0028(15)	0.0067(16)
C19	0.025(2)	0.031(2)	0.045(2)	0.0106(18)	0.0028(17)	0.0014(17)
C20	0.027(2)	0.025(2)	0.072(3)	0.010(2)	0.003(2)	-0.0052(17)
C21	0.032(2)	0.029(2)	0.065(3)	-0.010(2)	-0.006(2)	-0.0007(18)
C22	0.032(2)	0.028(2)	0.041(2)	-0.0083(17)	-0.0040(17)	0.0000(17)
C23	0.062(3)	0.037(2)	0.025(2)	-0.0076(18)	0.0089(19)	-0.006(2)
C24	0.136(3)	0.0697(19)	0.090(2)	0.0249(16)	0.0377(19)	0.0429(19)

Table S14. Fractional atomic coordinates and isotropic atomic displacement parameters U_{iso} of all independent atoms of the first molecule of **9**.

Atom label	*x*	*y*	*z*	U_{iso} [Å^2]	Atom label	*x*	*y*	*z*	U_{iso} [Å^2]
N1A	0.71065(15)	0.25327(11)	-0.0262(14)	0.0253(13)	C15A	0.81435(17)	0.18901(19)	0.6868(16)	0.0539(19)
N2A	0.75484(12)	0.30357(13)	0.2407(10)	0.0247(10)	C16A	0.84145(17)	0.1692(2)	0.8627(17)	0.0490(17)
N3A	0.74530(12)	0.38885(13)	0.2040(12)	0.0252(10)	C17A	0.83797(15)	0.11875(19)	0.8349(14)	0.0295(13)
N4A	0.70410(11)	0.33493(13)	-0.0729(11)	0.0228(10)	C18A	0.80820(16)	0.10903(18)	0.6459(14)	0.0290(13)
N5A	0.75174(12)	0.20461(14)	0.2782(11)	0.0278(11)	C19A	0.71408(15)	0.04146(17)	-0.0989(15)	0.0298(12)
N6A	0.74713(12)	0.11913(14)	0.2217(11)	0.0251(10)	C20A	0.69096(17)	0.01486(18)	-0.2904(16)	0.0350(14)
N7A	0.70562(11)	0.17129(13)	-0.0652(11)	0.0221(10)	C21A	0.66433(15)	0.04591(18)	-0.4365(13)	0.0297(12)
N8A	0.79519(11)	0.35687(14)	0.5026(10)	0.0247(10)	C22A	0.67109(14)	0.09073(17)	-0.3338(14)	0.0233(11)
N9A	0.69842(11)	0.41750(13)	-0.1378(10)	0.0228(9)	C23A	0.68073(19)	0.25259(13)	-0.2369(18)	0.0280(15)
N10A	0.79251(12)	0.15281(14)	0.5514(10)	0.0251(10)	H1A	0.824014	0.290497	0.507601	0.039
N11A	0.70256(11)	0.08872(13)	-0.1321(10)	0.0231(10)	H2A	0.870830	0.328355	0.858422	0.049
N12A	0.65732(16)	0.25226(11)	-0.4282(15)	0.0349(14)	H3A	0.850455	0.414773	0.940652	0.040
C1A	0.72459(14)	0.29958(16)	0.0651(13)	0.0201(11)	H4A	0.791975	0.430006	0.620515	0.033
C2A	0.76366(14)	0.34922(17)	0.3115(13)	0.0249(12)	H5A	0.729042	0.477696	0.026542	0.036
C3A	0.71630(13)	0.37782(17)	0.0049(12)	0.0209(11)	H6A	0.684888	0.524309	-0.324421	0.037
C4A	0.72413(14)	0.20736(16)	0.0784(13)	0.0193(11)	H7A	0.639187	0.466794	-0.581977	0.040
C5A	0.76216(14)	0.15912(16)	0.3441(12)	0.0224(11)	H8A	0.654090	0.385018	-0.396482	0.027
C6A	0.71843(13)	0.12894(15)	0.0139(11)	0.0178(11)	H9A	0.810482	0.222548	0.658019	0.065
C7A	0.82318(13)	0.32282(16)	0.5783(14)	0.0324(12)	H10A	0.860008	0.185869	0.986118	0.059
C8A	0.84871(15)	0.3436(2)	0.7679(15)	0.0408(15)	H11A	0.854133	0.095633	0.934229	0.035
C9A	0.83752(15)	0.3920(2)	0.8127(14)	0.0336(14)	H12A	0.799332	0.077979	0.586688	0.035
C10A	0.80533(16)	0.40020(17)	0.6420(14)	0.0278(13)	H13A	0.734230	0.029710	0.032209	0.036
C11A	0.70904(14)	0.46512(17)	-0.1028(14)	0.0297(13)	H14A	0.692600	-0.018944	-0.319995	0.042
C12A	0.68450(15)	0.49046(18)	-0.2947(15)	0.0306(13)	H15A	0.644929	0.036818	-0.582464	0.036
C13A	0.65905(16)	0.45847(19)	-0.4381(14)	0.0335(13)	H16A	0.656856	0.118788	-0.389409	0.028
C14A	0.66727(14)	0.41375(17)	-0.3389(13)	0.0229(11)					

Table S15. Fractional atomic coordinates and isotropic atomic displacement parameters U_{iso} of all independent atoms of the second molecule of **9**.

Atom label	*x*	*y*	*z*	U_{iso} [Å^2]	Atom label	*x*	*y*	*z*	U_{iso} [Å^2]
N1B	0.46117(15)	0.24816(10)	1.0091(14)	0.0234(13)	C15B	0.57366(15)	0.18236(19)	0.3913(16)	0.0429(15)
N2B	0.50066(12)	0.29806(14)	0.6926(11)	0.0260(10)	C16B	0.60070(14)	0.16140(18)	0.1968(16)	0.0366(14)
N3B	0.49569(12)	0.38310(14)	0.7724(10)	0.0221(10)	C17B	0.58956(15)	0.1123(2)	0.1496(16)	0.0353(14)
N4B	0.45469(12)	0.32952(13)	1.0560(11)	0.0246(10)	C18B	0.55613(15)	0.10465(18)	0.3208(15)	0.0291(13)
N5B	0.50526(12)	0.19935(14)	0.7248(12)	0.0281(11)	C19B	0.46097(16)	0.03652(17)	1.0637(15)	0.0332(14)
N6B	0.49644(12)	0.11424(14)	0.7592(10)	0.0233(10)	C20B	0.43781(17)	0.00941(18)	1.2364(15)	0.0340(14)
N7B	0.45453(12)	0.16657(13)	1.0423(11)	0.0263(11)	C21B	0.40971(15)	0.04089(19)	1.3834(14)	0.0308(12)
N8B	0.54035(12)	0.35159(15)	0.4314(12)	0.0282(10)	C22B	0.41768(15)	0.08657(19)	1.2953(14)	0.0291(13)
N9B	0.44988(12)	0.41140(13)	1.1196(10)	0.0229(10)	C23B	0.43152(18)	0.24754(13)	1.224(2)	0.0302(16)
N10B	0.54602(12)	0.14703(15)	0.4661(11)	0.0291(11)	H1B	0.555633	0.281426	0.290789	0.042
N11B	0.44915(12)	0.08445(13)	1.0878(10)	0.0240(10)	H2B	0.609340	0.320796	-0.014738	0.047
N12B	0.40593(18)	0.24728(12)	1.387(2)	0.0493(19)	H3B	0.602606	0.411550	0.060583	0.041
C1B	0.47281(14)	0.29442(17)	0.9145(15)	0.0247(12)	H4B	0.546847	0.425818	0.414571	0.035
C2B	0.51072(14)	0.34426(17)	0.6464(14)	0.0244(12)	H5B	0.483177	0.471436	0.986550	0.033
C3B	0.46763(16)	0.37411(16)	0.9714(13)	0.0258(13)	H6B	0.440983	0.519665	1.314264	0.041
C4B	0.47474(13)	0.20229(17)	0.9233(14)	0.0235(12)	H7B	0.389781	0.463184	1.561265	0.039
C5B	0.51460(14)	0.15364(17)	0.6640(13)	0.0235(11)	H8B	0.405208	0.380170	1.394059	0.036
C6B	0.46776(15)	0.12253(16)	0.9519(13)	0.0252(12)	H9B	0.573774	0.214732	0.461416	0.051
C7B	0.56089(13)	0.31493(18)	0.2791(13)	0.0348(12)	H10B	0.622892	0.176874	0.109161	0.044
C8B	0.59028(15)	0.3367(2)	0.1083(14)	0.0391(15)	H11B	0.602706	0.089392	0.024284	0.042
C9B	0.58657(15)	0.3875(2)	0.1518(15)	0.0345(14)	H12B	0.542309	0.075072	0.336401	0.035
C10B	0.55593(15)	0.39526(18)	0.3474(14)	0.0289(13)	H13B	0.482251	0.025162	0.942569	0.040
C11B	0.46190(16)	0.45959(16)	1.1058(13)	0.0276(12)	H14B	0.439375	-0.024522	1.259444	0.041
C12B	0.43908(17)	0.48590(18)	1.2839(16)	0.0345(14)	H15B	0.389231	0.031210	1.518231	0.037
C13B	0.41036(15)	0.4539(2)	1.4262(14)	0.0327(13)	H16B	0.404431	0.114790	1.361675	0.035
C14B	0.41836(15)	0.40863(19)	1.3308(15)	0.0304(13)					

Table S16. Anisotropic atomic displacement parameters U_{ij} of all independent atoms of the first molecule of **9**.

Atom label	U_{11} [Å^2]	U_{22} [Å^2]	U_{33} [Å^2]	U_{23} [Å^2]	U_{13} [Å^2]	U_{12} [Å^2]

N1A	0.028(3)	0.025(2)	0.024(3)	-0.0012(15)	-0.003(3)	0.0003(14)
N2A	0.026(2)	0.0228(19)	0.025(2)	0.0025(18)	0.000(2)	-0.0032(16)
N3A	0.022(2)	0.0206(19)	0.033(3)	0.000(2)	0.000(2)	0.0020(16)
N4A	0.021(2)	0.020(2)	0.027(2)	-0.0048(19)	0.002(2)	0.0005(16)
N5A	0.031(2)	0.025(2)	0.028(3)	0.0030(19)	-0.001(2)	-0.0019(17)
N6A	0.022(2)	0.026(2)	0.027(3)	-0.0032(19)	0.000(2)	-0.0008(17)
N7A	0.019(2)	0.024(2)	0.023(2)	0.0037(19)	0.001(2)	-0.0010(16)
N8A	0.023(2)	0.025(2)	0.026(2)	-0.0022(18)	-0.007(2)	-0.0013(16)
N9A	0.021(2)	0.0226(19)	0.025(2)	0.0036(19)	0.004(2)	0.0023(16)
N10A	0.029(2)	0.024(2)	0.022(2)	0.0019(18)	-0.0076(19)	-0.0049(16)
N11A	0.020(2)	0.027(2)	0.022(2)	-0.002(2)	0.002(2)	-0.0017(17)
N12A	0.040(3)	0.035(3)	0.030(3)	0.0031(17)	-0.013(3)	0.0007(16)
C1A	0.026(3)	0.017(2)	0.017(2)	-0.001(2)	0.007(2)	-0.0003(19)
C2A	0.025(2)	0.026(2)	0.024(3)	0.002(2)	0.000(2)	0.003(2)
C3A	0.013(2)	0.030(3)	0.019(3)	0.004(2)	0.003(2)	0.0051(19)
C4A	0.028(3)	0.018(2)	0.011(2)	-0.0039(19)	0.006(2)	-0.0006(19)
C5A	0.029(3)	0.024(2)	0.014(2)	-0.002(2)	0.004(2)	-0.0026(19)
C6A	0.013(2)	0.024(2)	0.016(3)	-0.0037(19)	0.004(2)	-0.0050(18)
C7A	0.030(2)	0.025(2)	0.043(3)	0.002(2)	-0.008(2)	-0.0012(19)
C8A	0.037(3)	0.046(3)	0.039(3)	0.006(3)	-0.013(3)	0.002(2)
C9A	0.027(3)	0.045(3)	0.029(3)	-0.009(3)	-0.001(3)	-0.004(2)
C10A	0.032(3)	0.025(2)	0.027(3)	-0.004(2)	0.006(3)	-0.005(2)
C11A	0.027(3)	0.024(2)	0.038(3)	-0.003(3)	0.003(3)	0.002(2)
C12A	0.035(3)	0.023(2)	0.034(3)	0.006(2)	-0.005(3)	0.005(2)
C13A	0.039(3)	0.029(3)	0.032(3)	0.011(2)	-0.004(3)	-0.002(2)
C14A	0.022(2)	0.023(2)	0.023(3)	0.002(2)	0.001(2)	0.0013(19)
C15A	0.067(4)	0.033(3)	0.061(4)	0.021(3)	-0.041(4)	-0.022(3)
C16A	0.041(3)	0.050(3)	0.056(4)	0.008(3)	-0.021(3)	-0.007(3)
C17A	0.027(3)	0.037(3)	0.024(3)	0.004(2)	-0.002(3)	0.005(2)
C18A	0.032(3)	0.028(3)	0.027(3)	0.001(2)	0.004(3)	0.003(2)
C19A	0.028(3)	0.027(2)	0.034(3)	0.001(2)	-0.003(3)	0.000(2)
C20A	0.048(3)	0.022(2)	0.035(3)	-0.004(2)	-0.007(3)	-0.003(2)
C21A	0.028(3)	0.028(3)	0.033(3)	-0.008(2)	0.000(3)	-0.005(2)
C22A	0.019(2)	0.027(2)	0.024(3)	0.002(2)	0.002(2)	-0.0011(19)
C23A	0.036(4)	0.024(3)	0.025(3)	-0.0022(18)	-0.006(3)	0.0000(17)

Table S17. Anisotropic atomic displacement parameters U_{ij} of all independent atoms of the second molecule of **9**.

Atom label	U_{11} [Å^2]	U_{22} [Å^2]	U_{33} [Å^2]	U_{23} [Å^2]	U_{13} [Å^2]	U_{12} [Å^2]
N1B	0.025(3)	0.019(2)	0.026(3)	-0.0004(14)	0.006(3)	0.0004(12)
N2B	0.028(2)	0.0218(19)	0.028(2)	0.0015(17)	0.007(2)	-0.0006(16)

N3B	0.028(2)	0.0231(19)	0.016(2)	0.0046(18)	0.000(2)	-0.0022(17)
N4B	0.030(2)	0.0196(19)	0.024(2)	-0.0010(19)	0.001(2)	-0.0025(17)
N5B	0.028(2)	0.0209(19)	0.036(3)	-0.0009(19)	0.009(2)	0.0014(16)
N6B	0.027(2)	0.0245(19)	0.018(2)	-0.0012(19)	0.002(2)	0.0021(17)
N7B	0.032(2)	0.020(2)	0.027(3)	0.0053(19)	0.003(2)	0.0004(17)
N8B	0.026(2)	0.027(2)	0.031(2)	0.0050(19)	-0.001(2)	-0.0005(17)
N9B	0.025(2)	0.0180(19)	0.025(2)	0.0013(18)	0.003(2)	-0.0029(16)
N10B	0.029(2)	0.023(2)	0.035(3)	0.0031(19)	0.003(2)	0.0017(17)
N11B	0.028(2)	0.0180(19)	0.026(2)	0.0001(18)	0.002(2)	-0.0001(16)
N12B	0.045(4)	0.031(3)	0.071(5)	-0.002(2)	0.024(4)	-0.0045(17)
C1B	0.019(2)	0.024(2)	0.031(3)	0.005(2)	0.002(2)	-0.0030(19)
C2B	0.020(2)	0.026(2)	0.027(3)	0.007(2)	-0.001(2)	-0.0036(19)
C3B	0.035(3)	0.018(2)	0.024(3)	0.006(2)	-0.007(3)	-0.003(2)
C4B	0.017(2)	0.023(2)	0.031(3)	0.002(2)	0.001(2)	-0.0005(19)
C5B	0.017(2)	0.027(2)	0.026(3)	0.000(2)	-0.005(2)	0.0053(19)
C6B	0.033(3)	0.018(2)	0.025(3)	-0.004(2)	-0.007(3)	0.007(2)
C7B	0.032(2)	0.038(3)	0.034(3)	0.003(2)	-0.002(2)	0.004(2)
C8B	0.032(3)	0.050(3)	0.035(3)	0.013(3)	0.010(3)	0.012(2)
C9B	0.025(3)	0.041(3)	0.038(3)	0.005(3)	-0.001(3)	-0.008(2)
C10B	0.026(3)	0.033(3)	0.027(3)	0.001(2)	0.004(3)	-0.005(2)
C11B	0.041(3)	0.016(2)	0.025(3)	0.004(2)	0.001(3)	-0.004(2)
C12B	0.039(3)	0.024(2)	0.041(3)	-0.003(2)	-0.009(3)	0.002(2)
C13B	0.030(3)	0.034(3)	0.034(3)	0.002(3)	0.003(3)	0.005(2)
C14B	0.028(3)	0.030(3)	0.034(3)	0.002(2)	0.003(3)	-0.004(2)
C15B	0.039(3)	0.034(3)	0.056(4)	0.007(3)	0.018(3)	-0.003(2)
C16B	0.021(2)	0.041(3)	0.047(3)	0.003(3)	0.006(2)	0.001(2)
C17B	0.028(3)	0.038(3)	0.039(4)	-0.004(3)	0.000(3)	0.008(2)
C18B	0.025(3)	0.032(3)	0.030(3)	-0.001(2)	0.004(3)	0.003(2)
C19B	0.045(3)	0.018(2)	0.037(3)	0.003(2)	0.006(3)	0.006(2)
C20B	0.039(3)	0.022(2)	0.041(4)	0.001(2)	-0.001(3)	0.002(2)
C21B	0.025(2)	0.036(3)	0.031(3)	-0.001(3)	-0.004(3)	-0.008(2)
C22B	0.023(3)	0.035(3)	0.030(3)	0.001(3)	0.001(3)	-0.001(2)
C23B	0.029(3)	0.020(3)	0.042(4)	0.000(2)	-0.001(4)	0.0002(16)

Table S18. Fractional atomic coordinates and isotropic atomic displacement parameters U_{iso} of all independent atoms of **11**.

Atom label	*x*	*y*	*z*	U_{iso} [Å^2]	Atom label	*x*	*y*	*z*	U_{iso} [Å^2]
O1	0.45403(8)	0.24754(8)	0.2947(7)	0.0390(7)	C15	0.49535(12)	0.38416(14)	0.8236(9)	0.0336(10)
N1	0.52611(10)	0.25813(9)	0.1909(7)	0.0287(7)	C16	0.50283(13)	0.42274(14)	0.9565(10)	0.0401(11)
N2	0.57616(9)	0.20820(9)	0.4147(7)	0.0281(7)	C17	0.47257(14)	0.45303(14)	0.8343(11)	0.0443(12)
N3	0.50074(9)	0.20361(9)	0.5299(7)	0.0284(7)	C18	0.44728(13)	0.43181(12)	0.6349(10)	0.0368(10)
N4	0.46109(9)	0.31781(9)	0.4591(7)	0.0269(7)	C19	0.33859(12)	0.38679(13)	-0.1054(10)	0.0364(10)
N5	0.40951(9)	0.36888(9)	0.2721(7)	0.0247(7)	C20	0.30626(13)	0.38292(15)	-0.3046(10)	0.0454(12)
N6	0.40767(9)	0.29574(9)	0.1001(7)	0.0278(7)	C21	0.30508(13)	0.33940(15)	-0.4091(11)	0.0448(11)
N7	0.59968(10)	0.26076(9)	0.0803(8)	0.0312(8)	C22	0.33754(12)	0.31717(14)	-0.2687(9)	0.0360(10)
N8	0.55008(10)	0.15593(9)	0.7437(7)	0.0300(8)	H1	0.571027	0.311658	-0.158231	0.050
N9	0.46083(9)	0.38915(9)	0.6251(7)	0.0265(7)	H2	0.643657	0.326953	-0.358557	0.060
N10	0.35871(9)	0.34628(10)	-0.0789(7)	0.0284(7)	H3	0.695774	0.271857	-0.133330	0.064
C1	0.49630(12)	0.23700(11)	0.3457(9)	0.0280(9)	H4	0.653879	0.224285	0.206129	0.050
C2	0.56606(11)	0.24162(11)	0.2402(9)	0.0264(9)	H5	0.616558	0.145273	0.702745	0.043
C3	0.54201(12)	0.19087(11)	0.5543(9)	0.0270(9)	H6	0.606123	0.084241	1.054844	0.050
C4	0.44098(11)	0.28968(11)	0.2806(9)	0.0258(8)	H7	0.525951	0.081254	1.191378	0.055
C5	0.44302(11)	0.35737(11)	0.4421(8)	0.0240(8)	H8	0.488959	0.140509	0.919170	0.045
C6	0.39347(11)	0.33667(12)	0.1067(8)	0.0253(8)	H9	0.510968	0.358102	0.859526	0.040
C7	0.59674(14)	0.29604(12)	-0.1117(10)	0.0413(11)	H10	0.524375	0.428716	1.104799	0.048
C8	0.63633(15)	0.30441(14)	-0.2208(12)	0.0499(12)	H11	0.470612	0.482911	0.884891	0.053
C9	0.66552(15)	0.27367(14)	-0.0945(12)	0.0534(13)	H12	0.424168	0.444020	0.520779	0.044
C10	0.64261(12)	0.24764(13)	0.0897(11)	0.0413(11)	H13	0.346552	0.412479	-0.000194	0.044
C11	0.58983(13)	0.13655(12)	0.7909(10)	0.0359(10)	H14	0.287226	0.405487	-0.366156	0.054
C12	0.58418(14)	0.10326(12)	0.9828(10)	0.0420(11)	H15	0.285132	0.327807	-0.552004	0.054
C13	0.53926(15)	0.10162(13)	1.0591(11)	0.0457(12)	H16	0.344423	0.287381	-0.295527	0.043
C14	0.51904(13)	0.13395(12)	0.9108(10)	0.0374(10)					

Table S19. Anisotropic atomic displacement parameters U_{ij} of all independent atoms of **11**.

Atom label	U_{11} [Å^2]	U_{22} [Å^2]	U_{33} [Å^2]	U_{23} [Å^2]	U_{13} [Å^2]	U_{12} [Å^2]
O1	0.0230(13)	0.0224(13)	0.071(2)	0.0009(14)	-0.0112(14)	0.0014(11)
N1	0.0305(17)	0.0221(16)	0.0334(19)	-0.0011(15)	-0.0058(15)	0.0031(13)
N2	0.0272(17)	0.0259(16)	0.0313(18)	0.0039(15)	-0.0044(15)	-0.0003(13)
N3	0.0281(17)	0.0239(16)	0.0331(19)	-0.0012(15)	-0.0028(15)	0.0027(13)
N4	0.0249(16)	0.0273(17)	0.0286(18)	0.0018(15)	0.0008(14)	0.0023(13)
N5	0.0250(16)	0.0244(16)	0.0246(17)	-0.0001(14)	-0.0029(14)	0.0003(13)
N6	0.0245(16)	0.0279(17)	0.0310(18)	-0.0024(15)	0.0007(15)	0.0016(13)
N7	0.0308(17)	0.0240(16)	0.039(2)	0.0043(15)	0.0001(16)	0.0040(13)
N8	0.0349(18)	0.0241(16)	0.0311(19)	0.0035(15)	-0.0068(16)	-0.0021(14)
N9	0.0229(16)	0.0317(17)	0.0248(17)	-0.0058(14)	0.0017(14)	0.0009(13)
N10	0.0268(16)	0.0352(18)	0.0232(17)	0.0002(15)	-0.0010(14)	0.0011(14)
C1	0.028(2)	0.0240(19)	0.032(2)	-0.0046(18)	-0.0061(18)	0.0019(16)
C2	0.0261(19)	0.0249(19)	0.028(2)	-0.0042(17)	-0.0028(17)	0.0036(15)
C3	0.031(2)	0.0249(19)	0.025(2)	-0.0048(17)	-0.0053(17)	-0.0008(16)
C4	0.0215(18)	0.0229(19)	0.033(2)	0.0034(18)	0.0036(18)	0.0004(15)
C5	0.0250(19)	0.0264(19)	0.0204(19)	0.0012(16)	0.0051(17)	0.0030(15)
C6	0.0242(19)	0.033(2)	0.0185(19)	0.0027(17)	0.0027(16)	0.0013(16)
C7	0.045(3)	0.029(2)	0.050(3)	0.011(2)	0.003(2)	0.0057(19)
C8	0.053(3)	0.034(2)	0.063(3)	0.018(2)	0.013(3)	0.001(2)
C9	0.038(2)	0.048(3)	0.075(4)	0.012(3)	0.014(3)	-0.001(2)
C10	0.030(2)	0.036(2)	0.058(3)	0.009(2)	0.001(2)	0.0060(18)
C11	0.032(2)	0.033(2)	0.043(3)	0.004(2)	-0.010(2)	0.0040(17)
C12	0.050(3)	0.027(2)	0.049(3)	0.007(2)	-0.017(2)	0.0043(19)
C13	0.054(3)	0.036(2)	0.048(3)	0.014(2)	-0.005(2)	-0.011(2)
C14	0.037(2)	0.033(2)	0.042(3)	0.005(2)	-0.002(2)	-0.0054(18)
C15	0.023(2)	0.049(3)	0.028(2)	-0.004(2)	0.0015(18)	-0.0027(17)
C16	0.031(2)	0.052(3)	0.037(3)	-0.007(2)	-0.004(2)	-0.010(2)
C17	0.044(2)	0.037(2)	0.053(3)	-0.017(2)	0.001(2)	-0.0034(19)
C18	0.037(2)	0.030(2)	0.043(3)	-0.006(2)	-0.004(2)	0.0052(18)
C19	0.035(2)	0.034(2)	0.040(3)	0.005(2)	-0.003(2)	0.0095(18)
C20	0.035(2)	0.060(3)	0.041(3)	0.012(2)	-0.011(2)	0.013(2)
C21	0.032(2)	0.064(3)	0.038(3)	0.001(2)	-0.009(2)	-0.002(2)
C22	0.035(2)	0.044(2)	0.029(2)	-0.002(2)	-0.0043(19)	-0.0040(19)

Table S20. Fractional atomic coordinates and isotropic atomic displacement parameters U_{iso} of all independent atoms of **12**.

Atom label	*x*	*y*	*z*	U_{iso} [Å^2]	Atom label	*x*	*y*	*z*	U_{iso} [Å^2]
N1	0.3927(2)	0.43551(19)	0.5164(3)	0.0305(7)	C12	0.1452(2)	0.6799(2)	0.3519(4)	0.0292(8)
N2	0.33304(18)	0.4978(2)	0.2911(3)	0.0227(6)	C13	0.000000	0.500000	-0.0663(5)	0.0269(10)
N3	0.2667(2)	0.55718(19)	0.5141(3)	0.0298(7)	C14	0.0587(2)	0.4438(2)	0.1399(3)	0.0201(7)
N4	0.3269(4)	0.4953(4)	0.8604(4)	0.0766(15)	C15	0.000000	0.500000	-0.2279(6)	0.0388(15)
N5	0.45345(19)	0.38016(18)	0.2971(3)	0.0249(6)	C16	0.1888(2)	0.3183(2)	0.1533(4)	0.0267(8)
N6	0.21243(19)	0.61546(19)	0.2926(4)	0.0246(6)	C17	0.2302(3)	0.2680(3)	0.2629(4)	0.0309(10)
N7	0.0635(2)	0.43979(18)	-0.0063(3)	0.0251(6)	C18	0.1886(2)	0.3016(2)	0.3963(4)	0.0305(8)
N8	0.000000	0.500000	0.2183(4)	0.0210(8)	C19	0.1221(2)	0.3716(2)	0.3651(4)	0.0239(7)
N9	0.000000	0.500000	-0.3491(5)	0.0676(18)	H1	0.520215	0.296131	0.459232	0.035
N10	0.1210(2)	0.38271(19)	0.2143(3)	0.0229(6)	H2	0.608799	0.215231	0.258838	0.041
C1	0.3288(3)	0.4966(3)	0.5782(3)	0.0314(8)	H3	0.555434	0.292273	0.023486	0.041
C2	0.3900(2)	0.4404(2)	0.3712(3)	0.0234(7)	H4	0.431469	0.418214	0.079916	0.034
C3	0.2732(2)	0.5541(2)	0.3690(3)	0.0227(7)	H5	0.252405	0.590622	0.076006	0.033
C4	0.3271(4)	0.4949(5)	0.7361(4)	0.0483(12)	H6	0.131569	0.719349	0.014783	0.038
C5	0.5141(2)	0.3102(2)	0.3588(4)	0.0290(8)	H7	0.056796	0.779969	0.250123	0.039
C6	0.5624(3)	0.2663(3)	0.2492(4)	0.0343(10)	H8	0.130201	0.687279	0.451994	0.035
C7	0.5323(2)	0.3091(3)	0.1174(4)	0.0338(9)	H9	0.203150	0.311160	0.052950	0.032
C8	0.4648(2)	0.3784(2)	0.1480(4)	0.0287(8)	H10	0.278570	0.218709	0.253113	0.037
C9	0.2131(2)	0.6261(2)	0.1428(4)	0.0272(8)	H11	0.204828	0.278803	0.490515	0.037
C10	0.1469(2)	0.6967(3)	0.1093(4)	0.0320(9)	H12	0.083383	0.406687	0.433021	0.029
C11	0.1049(3)	0.7304(3)	0.2412(4)	0.0327(10)					

Table S21. Anisotropic atomic displacement parameters U_{ij} of all independent atoms of **12**.

Atom label	U_{11} [Å^2]	U_{22} [Å^2]	U_{33} [Å^2]	U_{23} [Å^2]	U_{13} [Å^2]	U_{12} [Å^2]
N1	0.0331(15)	0.0327(14)	0.0257(14)	-0.0015(11)	-0.0029(12)	0.0042(13)
N2	0.0197(13)	0.0238(14)	0.0245(12)	-0.0004(15)	-0.0001(12)	-0.0035(13)
N3	0.0338(16)	0.0314(14)	0.0240(14)	-0.0041(12)	-0.0009(12)	0.0032(13)
N4	0.111(4)	0.093(3)	0.0264(18)	0.002(2)	-0.002(2)	0.046(4)
N5	0.0197(13)	0.0220(14)	0.0330(15)	-0.0023(14)	-0.0024(13)	0.0005(11)
N6	0.0199(13)	0.0225(14)	0.0314(14)	-0.0017(16)	-0.0016(13)	0.0005(12)
N7	0.0279(15)	0.0275(14)	0.0199(13)	-0.0020(11)	-0.0004(12)	0.0016(13)
N8	0.021(2)	0.023(2)	0.0191(15)	0.000	0.000	0.0021(19)
N9	0.097(5)	0.084(4)	0.021(2)	0.000	0.000	0.039(4)
N10	0.0214(13)	0.0238(14)	0.0234(13)	-0.0021(14)	-0.0006(14)	-0.0015(13)
C1	0.037(2)	0.0349(19)	0.0227(15)	0.0017(17)	-0.0003(14)	0.0057(19)
C2	0.0216(15)	0.0215(15)	0.0269(15)	-0.0001(12)	-0.0003(13)	-0.0047(13)
C3	0.0197(14)	0.0203(14)	0.0280(15)	-0.0021(13)	-0.0016(13)	-0.0047(13)
C4	0.058(3)	0.051(3)	0.036(2)	-0.0028(19)	-0.0027(18)	0.016(3)
C5	0.0249(16)	0.0258(16)	0.0363(18)	0.0017(15)	-0.0058(16)	0.0010(15)
C6	0.027(2)	0.028(2)	0.048(3)	-0.0048(14)	-0.0040(15)	0.0040(18)
C7	0.0274(18)	0.0384(19)	0.0356(19)	-0.0132(16)	0.0003(15)	0.0054(16)
C8	0.0247(16)	0.0315(18)	0.0301(17)	-0.0057(15)	-0.0014(14)	-0.0018(15)
C9	0.0224(15)	0.0315(18)	0.0275(17)	0.0030(16)	-0.0007(13)	-0.0027(15)
C10	0.0224(17)	0.035(2)	0.039(2)	0.0104(17)	-0.0036(15)	0.0021(15)
C11	0.024(2)	0.026(2)	0.048(3)	-0.0021(14)	-0.0034(15)	0.0055(17)
C12	0.0243(17)	0.0293(17)	0.034(2)	-0.0060(16)	-0.0023(15)	0.0007(15)
C13	0.032(3)	0.031(2)	0.018(2)	0.000	0.000	-0.001(2)
C14	0.0194(15)	0.0229(15)	0.0180(14)	0.0002(12)	0.0001(13)	-0.0025(14)
C15	0.051(4)	0.043(3)	0.022(2)	0.000	0.000	0.020(3)
C16	0.0241(17)	0.0258(17)	0.0301(19)	-0.0046(16)	0.0006(16)	0.0038(15)
C17	0.026(2)	0.026(2)	0.041(2)	0.0000(14)	-0.0027(15)	0.0029(16)
C18	0.0262(18)	0.0314(19)	0.034(2)	0.0078(16)	-0.0012(15)	-0.0056(16)
C19	0.0204(14)	0.0287(17)	0.0227(15)	0.0011(14)	-0.0004(14)	0.0002(15)

Table S22. Fractional atomic coordinates and isotropic atomic displacement parameters U_{iso} of all independent atoms of **13i**.

Atom label	*x*	*y*	*z*	U_{iso} [Å^2]
N1	0.41570(9)	0.5948(3)	0.39605(10)	0.0273(3)
N2	0.25252(10)	0.5444(3)	0.43952(10)	0.0278(3)
N3	0.34089(10)	0.8687(2)	0.50945(9)	0.0272(3)
N4	0.49762(10)	0.9019(3)	0.46850(10)	0.0315(4)
N5	0.32486(9)	0.2922(2)	0.32658(10)	0.0274(3)
N6	0.18178(10)	0.8132(3)	0.54650(10)	0.0296(4)
C1	0.41465(11)	0.7850(3)	0.45753(11)	0.0256(4)
C2	0.33217(11)	0.4874(3)	0.39065(11)	0.0254(4)
C3	0.26205(11)	0.7387(3)	0.49627(11)	0.0260(4)
C4	0.39424(12)	0.2131(3)	0.26131(12)	0.0308(4)
C5	0.35946(13)	0.0182(3)	0.21324(12)	0.0345(4)
C6	0.26620(13)	-0.0267(3)	0.24916(12)	0.0340(4)
C7	0.24644(12)	0.1424(3)	0.31788(12)	0.0305(4)
C8	0.09720(12)	0.6866(4)	0.55260(13)	0.0393(5)
C9	0.03802(14)	0.8110(4)	0.61094(15)	0.0490(6)
C10	0.08550(14)	1.0236(4)	0.64213(14)	0.0473(5)
C11	0.17326(13)	1.0213(3)	0.60283(13)	0.0371(5)
H1	0.548374	0.854240	0.435952	0.038
H2	0.455143	0.282874	0.251993	0.037
H3	0.391769	-0.072625	0.164079	0.041
H4	0.225254	-0.152797	0.228529	0.041
H5	0.188835	0.156464	0.354053	0.037
H6	0.083641	0.537939	0.521028	0.047
H7	-0.024762	0.765298	0.628448	0.059
H8	0.059775	1.145605	0.683391	0.057
H9	0.220823	1.140264	0.611924	0.045

Table S23. Anisotropic atomic displacement parameters U_{ij} of all independent atoms of **13i**.

Atom label	U_{11} [Å^2]	U_{22} [Å^2]	U_{33} [Å^2]	U_{23} [Å^2]	U_{13} [Å^2]	U_{12} [Å^2]
N1	0.0279(8)	0.0271(8)	0.0268(7)	-0.0005(6)	-0.0002(6)	-0.0033(6)
N2	0.0292(8)	0.0287(8)	0.0256(7)	0.0001(6)	0.0012(6)	-0.0031(6)
N3	0.0302(8)	0.0269(8)	0.0244(7)	0.0015(6)	-0.0017(6)	-0.0026(6)
N4	0.0302(8)	0.0306(8)	0.0338(8)	-0.0081(6)	0.0012(6)	-0.0057(6)
N5	0.0293(7)	0.0264(8)	0.0264(7)	-0.0018(6)	-0.0011(6)	-0.0034(6)
N6	0.0305(8)	0.0328(8)	0.0256(7)	-0.0006(6)	0.0016(6)	-0.0005(7)
C1	0.0288(9)	0.0259(9)	0.0221(8)	0.0041(7)	-0.0024(6)	-0.0027(7)
C2	0.0302(9)	0.0237(9)	0.0222(8)	0.0039(7)	-0.0014(6)	-0.0015(7)
C3	0.0298(9)	0.0266(9)	0.0216(8)	0.0029(7)	-0.0014(6)	-0.0009(7)
C4	0.0310(9)	0.0332(10)	0.0281(9)	-0.0019(8)	0.0013(7)	0.0003(8)
C5	0.0404(10)	0.0340(10)	0.0292(9)	-0.0051(8)	-0.0043(7)	0.0044(8)
C6	0.0417(10)	0.0286(9)	0.0317(9)	-0.0010(8)	-0.0116(8)	-0.0047(8)
C7	0.0295(9)	0.0317(9)	0.0303(9)	0.0027(8)	-0.0047(7)	-0.0064(7)
C8	0.0295(10)	0.0502(12)	0.0382(10)	-0.0066(9)	0.0004(8)	-0.0052(9)
C9	0.0294(10)	0.0695(15)	0.0481(12)	-0.0093(11)	0.0058(9)	0.0004(10)
C10	0.0450(12)	0.0553(14)	0.0415(11)	-0.0101(10)	0.0054(9)	0.0113(10)
C11	0.0430(11)	0.0352(10)	0.0331(9)	-0.0052(8)	0.0009(8)	0.0031(9)

Table S24. Fractional atomic coordinates and isotropic atomic displacement parameters U_{iso} of all independent atoms of **13**.

Atom label	*x*	*y*	*z*	U_{iso} [Å^2]
N1	0.59752(8)	0.3875(3)	0.40740(9)	0.0307(3)
N2	0.76296(8)	0.5521(3)	0.44875(7)	0.0233(3)
N3	0.66903(9)	0.7403(3)	0.52245(8)	0.0310(3)
N4	0.50514(10)	0.5897(4)	0.47714(10)	0.0451(4)
N5	0.69185(8)	0.2014(3)	0.33975(8)	0.0254(3)
N6	0.82803(9)	0.9032(3)	0.55884(7)	0.0255(3)
C1	0.59762(10)	0.5662(4)	0.46943(10)	0.0307(3)
C2	0.68410(10)	0.3899(3)	0.40115(9)	0.0248(3)
C3	0.75025(10)	0.7224(3)	0.50764(9)	0.0240(3)
C4	0.61652(10)	0.0225(4)	0.28208(10)	0.0315(3)
C5	0.65256(11)	-0.1273(4)	0.23234(10)	0.0340(4)
C6	0.75289(11)	-0.0408(4)	0.25929(10)	0.0318(3)
C7	0.77530(10)	0.1599(3)	0.32444(9)	0.0278(3)
C8	0.91785(10)	0.9266(4)	0.55338(9)	0.0287(3)
C9	0.97330(11)	1.1284(4)	0.61390(10)	0.0335(4)
C10	0.91705(12)	1.2324(4)	0.65863(10)	0.0348(4)
C11	0.82848(11)	1.0927(3)	0.62414(9)	0.0307(3)
H1	0.551573	0.008605	0.278473	0.038
H2	0.617484	-0.265398	0.187457	0.041
H3	0.796311	-0.112143	0.235513	0.038
H4	0.836998	0.255397	0.354379	0.033
H5	0.936979	0.820517	0.514351	0.034
H6	1.038206	1.189695	0.624606	0.040
H7	0.937981	1.374515	0.704524	0.042
H8	0.776217	1.119387	0.641400	0.037

Table S25. Anisotropic atomic displacement parameters U_{ij} of all independent atoms of **13**.

Atom label	U_{11} [Å^2]	U_{22} [Å^2]	U_{33} [Å^2]	U_{23} [Å^2]	U_{13} [Å^2]	U_{12} [Å^2]
N1	0.0222(6)	0.0298(6)	0.0437(7)	0.0058(6)	0.0170(5)	0.0025(5)
N2	0.0207(5)	0.0240(6)	0.0277(6)	0.0056(5)	0.0125(4)	0.0034(4)
N3	0.0289(6)	0.0362(7)	0.0346(7)	0.0064(6)	0.0198(5)	0.0075(5)
N4	0.0326(7)	0.0522(9)	0.0632(10)	0.0212(8)	0.0323(7)	0.0132(7)
N5	0.0220(5)	0.0232(6)	0.0309(6)	0.0032(5)	0.0105(5)	0.0007(5)
N6	0.0283(6)	0.0247(6)	0.0270(6)	0.0036(5)	0.0149(5)	0.0039(5)
C1	0.0242(7)	0.0328(8)	0.0409(8)	0.0122(7)	0.0192(6)	0.0075(6)
C2	0.0224(6)	0.0231(7)	0.0307(7)	0.0085(5)	0.0128(5)	0.0045(5)
C3	0.0242(6)	0.0240(7)	0.0267(7)	0.0079(5)	0.0135(5)	0.0055(5)
C4	0.0233(7)	0.0286(7)	0.0363(8)	0.0038(6)	0.0057(6)	-0.0013(6)
C5	0.0359(8)	0.0295(8)	0.0299(7)	0.0009(6)	0.0066(6)	-0.0015(6)
C6	0.0366(8)	0.0308(8)	0.0317(7)	0.0016(6)	0.0177(6)	0.0001(6)
C7	0.0252(7)	0.0279(7)	0.0332(7)	0.0017(6)	0.0148(6)	-0.0002(6)
C8	0.0265(7)	0.0309(7)	0.0318(7)	0.0013(6)	0.0149(6)	0.0024(6)
C9	0.0299(7)	0.0336(8)	0.0347(8)	0.0012(6)	0.0110(6)	0.0019(6)
C10	0.0408(8)	0.0331(8)	0.0271(7)	-0.0006(6)	0.0102(6)	0.0051(7)
C11	0.0383(8)	0.0300(7)	0.0275(7)	0.0036(6)	0.0172(6)	0.0091(6)

Table S26. Fractional atomic coordinates and isotropic atomic displacement parameters U_{iso} of all independent atoms of **14**.

Atom label	*x*	*y*	*z*	U_{iso} [Å^2]	Atom label	*x*	*y*	*z*	U_{iso} [Å^2]
N1	1.0961(7)	0.25983(14)	0.23230(10)	0.0281(6)	C13	1.2973(9)	0.34890(19)	0.02790(14)	0.0370(8)
N2	0.8249(6)	0.15045(13)	0.27857(11)	0.0261(5)	C14	1.3968(10)	0.3936(2)	-0.02093(14)	0.0450(10)
N3	0.7740(6)	0.17133(14)	0.17295(11)	0.0282(6)	C15	1.3694(10)	0.4744(2)	-0.01933(14)	0.0450(10)
N4	1.0521(7)	0.27303(14)	0.12610(11)	0.0314(6)	C16	1.2437(11)	0.5119(2)	0.03076(15)	0.0471(10)
N5	1.1523(6)	0.23524(14)	0.33426(10)	0.0271(6)	C17	1.1450(9)	0.46757(18)	0.08005(14)	0.0384(8)
N6	0.5085(6)	0.06707(14)	0.21970(10)	0.0272(6)	H1	1.388.730	0.342380	0.316857	0.039
N7	1.0661(7)	0.34637(14)	0.13154(10)	0.0313(6)	H2	1.549.865	0.340730	0.424202	0.045
C1	0.9645(8)	0.23431(16)	0.18121(12)	0.0262(6)	H3	1.321.209	0.214712	0.470755	0.045
C2	1.0151(7)	0.21412(16)	0.27920(13)	0.0257(6)	H4	1.016.876	0.143069	0.391377	0.041
C3	0.7108(7)	0.13244(15)	0.22408(13)	0.0260(6)	H5	0.477083	0.024046	0.307868	0.038
C4	1.3351(8)	0.30255(18)	0.34533(14)	0.0321(7)	H6	0.129449	-0.082521	0.265873	0.040
C5	1.4240(9)	0.3015(2)	0.40401(14)	0.0372(8)	H7	0.083558	-0.059821	0.153196	0.042
C6	1.2952(8)	0.2311(2)	0.43015(14)	0.0371(8)	H8	0.398819	0.060044	0.127695	0.036
C7	1.1294(9)	0.19188(19)	0.38668(13)	0.0338(8)	H9	1.316.112	0.293001	0.026878	0.044
C8	0.4171(8)	0.01782(17)	0.26682(14)	0.0313(7)	H10	1.484.297	0.368414	-0.055628	0.054
C9	0.2276(8)	-0.04055(17)	0.24388(14)	0.0337(8)	H11	1.437.716	0.504729	-0.053056	0.054
C10	0.2020(8)	-0.02766(18)	0.18076(14)	0.0346(7)	H12	1.224.759	0.567794	0.031489	0.057
C11	0.3740(8)	0.03788(17)	0.16671(13)	0.0300(7)	H13	1.060.373	0.493010	0.114883	0.046
C12	1.1702(9)	0.38619(17)	0.07827(13)	0.0300(7)					

Table S27. Anisotropic atomic displacement parameters U_{ij} of all independent atoms of **14**.

Atom label	U_{11} [Å^2]	U_{22} [Å^2]	U_{33} [Å^2]	U_{23} [Å^2]	U_{13} [Å^2]	U_{12} [Å^2]
N1	0.0326(15)	0.0255(12)	0.0262(13)	0.0000(10)	0.0034(11)	0.0007(11)
N2	0.0262(13)	0.0258(12)	0.0263(12)	0.0013(10)	0.0040(11)	0.0016(11)
N3	0.0323(15)	0.0251(12)	0.0272(13)	0.0006(10)	0.0043(11)	0.0015(11)
N4	0.0416(17)	0.0266(13)	0.0261(13)	0.0004(10)	0.0041(12)	-0.0031(13)
N5	0.0277(14)	0.0273(12)	0.0262(13)	-0.0009(10)	0.0023(11)	0.0014(12)
N6	0.0273(13)	0.0257(12)	0.0285(13)	0.0030(10)	0.0036(12)	0.0010(11)
N7	0.0417(16)	0.0270(13)	0.0252(13)	0.0007(10)	0.0037(12)	-0.0014(12)
C1	0.0315(17)	0.0217(13)	0.0255(15)	-0.0006(12)	0.0056(13)	0.0039(14)
C2	0.0277(16)	0.0240(15)	0.0254(15)	-0.0025(12)	0.0051(14)	0.0054(12)
C3	0.0252(16)	0.0203(13)	0.0324(15)	-0.0006(12)	0.0059(14)	0.0054(12)
C4	0.0330(17)	0.0274(15)	0.0360(17)	0.0006(13)	0.0000(14)	-0.0007(15)
C5	0.0363(19)	0.0386(18)	0.0367(18)	-0.0063(14)	-0.0054(15)	-0.0002(16)
C6	0.0363(19)	0.0473(19)	0.0277(16)	0.0019(14)	-0.0004(14)	0.0033(17)
C7	0.0368(19)	0.0345(17)	0.0301(16)	0.0061(13)	0.0049(14)	-0.0003(15)
C8	0.0310(18)	0.0311(16)	0.0317(16)	0.0072(13)	0.0056(13)	0.0025(14)
C9	0.0335(19)	0.0247(15)	0.0430(18)	0.0076(13)	0.0075(15)	-0.0012(15)
C10	0.0332(18)	0.0291(16)	0.0415(18)	0.0011(14)	-0.0031(15)	0.0013(15)
C11	0.0325(18)	0.0262(15)	0.0314(16)	0.0012(13)	0.0003(13)	0.0032(14)
C12	0.0394(19)	0.0286(15)	0.0220(14)	0.0015(12)	0.0017(14)	-0.0042(15)
C13	0.048(2)	0.0319(17)	0.0311(16)	-0.0044(13)	0.0073(16)	-0.0042(17)
C14	0.061(3)	0.047(2)	0.0272(17)	-0.0048(15)	0.0108(17)	-0.0096(19)
C15	0.061(3)	0.046(2)	0.0279(17)	0.0081(15)	0.0082(17)	-0.006(2)
C16	0.073(3)	0.0306(17)	0.0380(18)	0.0065(14)	0.0107(19)	-0.0021(19)
C17	0.056(2)	0.0295(16)	0.0300(16)	-0.0017(13)	0.0094(16)	0.0018(17)

Table S28. Fractional atomic coordinates and isotropic atomic displacement parameters U_{iso} of all independent atoms of **15·H_2O**.

Atom label	*x*	*y*	*z*	U_{iso} [$Å^2$]
O1	0.70833(13)	1.01350(18)	0.68943(9)	0.0315(3)
N1	0.84668(11)	0.59273(17)	0.71508(8)	0.0146(3)
N2	0.67401(11)	0.42258(17)	0.73828(8)	0.0145(3)
N3	0.67328(12)	0.34869(19)	0.89930(9)	0.0191(3)
N4	0.85730(12)	0.50340(17)	0.87051(9)	0.0154(3)
N5	0.66166(11)	0.67392(17)	0.59122(8)	0.0146(3)
N6	0.66035(12)	0.62501(18)	0.42679(8)	0.0172(3)
N7	0.85271(12)	0.55746(18)	0.55602(9)	0.0160(3)
N8	0.50991(13)	0.2511(2)	0.76731(10)	0.0212(3)
N9	0.84926(15)	0.4304(3)	1.02369(10)	0.0309(4)
N10	0.47582(12)	0.7216(2)	0.46603(10)	0.0198(3)
N11	0.85123(13)	0.5314(2)	0.39680(9)	0.0204(3)
C1	0.78928(13)	0.50410(19)	0.77693(10)	0.0123(3)
C2	0.62178(14)	0.3451(2)	0.80343(10)	0.0151(3)
C3	0.79233(14)	0.4265(2)	0.92853(10)	0.0180(3)
C4	0.78278(13)	0.60759(19)	0.61516(10)	0.0132(3)
C5	0.60177(14)	0.6718(2)	0.49452(10)	0.0149(3)
C6	0.78656(14)	0.5739(2)	0.46071(10)	0.0159(3)
C7	0.98288(14)	0.6548(2)	0.75013(11)	0.0167(3)
H1	0.461(2)	0.286(3)	0.7139(16)	0.023(5)
H2	0.472(2)	0.209(3)	0.8065(16)	0.026(5)
H3	0.803(3)	0.383(4)	1.0606(19)	0.047(7)
H4	0.929(2)	0.465(3)	1.0479(17)	0.030(5)
H5	0.435(2)	0.755(3)	0.5085(16)	0.026(5)
H6	0.432(2)	0.705(3)	0.4082(17)	0.031(6)
H7	0.808(2)	0.530(3)	0.3348(17)	0.028(5)
H8	0.933(2)	0.503(3)	0.4153(15)	0.025(5)
H9	0.9907(19)	0.733(3)	0.8042(15)	0.022(5)
H10	1.0042(19)	0.719(3)	0.6970(15)	0.024(5)
H11	1.0409(19)	0.551(3)	0.7682(14)	0.021(5)
H12	0.665(3)	0.905(4)	0.644(2)	0.095(12)
H13	0.618(3)	1.051(5)	0.703(2)	0.074(10)

Table S29. Anisotropic atomic displacement parameters U_{ij} of all independent atoms of **15·H_2O**.

Atom label	U_{11} [Å^2]	U_{22} [Å^2]	U_{33} [Å^2]	U_{23} [Å^2]	U_{13} [Å^2]	U_{12} [Å^2]
O1	0.0311(7)	0.0332(7)	0.0285(7)	-0.0034(5)	0.0057(5)	-0.0001(5)
N1	0.0104(5)	0.0240(6)	0.0094(6)	0.0014(5)	0.0025(4)	-0.0018(5)
N2	0.0117(5)	0.0213(6)	0.0099(5)	0.0012(4)	0.0024(4)	-0.0016(4)
N3	0.0153(6)	0.0291(7)	0.0123(6)	0.0029(5)	0.0031(5)	-0.0056(5)
N4	0.0134(6)	0.0223(6)	0.0099(6)	0.0006(5)	0.0022(4)	-0.0027(5)
N5	0.0131(6)	0.0204(6)	0.0103(6)	0.0007(4)	0.0032(4)	0.0016(4)
N6	0.0130(6)	0.0283(7)	0.0099(5)	0.0009(5)	0.0026(4)	0.0022(5)
N7	0.0126(5)	0.0244(6)	0.0108(6)	0.0006(5)	0.0032(4)	0.0019(5)
N8	0.0162(6)	0.0332(8)	0.0125(6)	0.0047(5)	0.0012(5)	-0.0088(5)
N9	0.0219(7)	0.0585(11)	0.0106(6)	0.0052(6)	0.0017(5)	-0.0181(7)
N10	0.0139(6)	0.0341(7)	0.0112(6)	0.0009(5)	0.0028(5)	0.0057(5)
N11	0.0139(6)	0.0369(8)	0.0107(6)	-0.0005(5)	0.0041(5)	0.0042(5)
C1	0.0114(6)	0.0157(6)	0.0101(6)	0.0001(5)	0.0035(5)	0.0025(5)
C2	0.0136(6)	0.0181(7)	0.0133(6)	0.0011(5)	0.0034(5)	0.0002(5)
C3	0.0151(7)	0.0259(8)	0.0125(7)	0.0016(5)	0.0030(5)	-0.0028(6)
C4	0.0127(6)	0.0164(6)	0.0105(6)	0.0009(5)	0.0032(5)	-0.0015(5)
C5	0.0143(6)	0.0185(7)	0.0118(6)	0.0015(5)	0.0034(5)	0.0010(5)
C6	0.0140(6)	0.0214(7)	0.0122(7)	0.0006(5)	0.0034(5)	0.0002(5)
C7	0.0120(6)	0.0256(8)	0.0122(6)	-0.0003(6)	0.0030(5)	-0.0053(5)

Table S30. Fractional atomic coordinates and isotropic atomic displacement parameters U_{iso} of all independent atoms of **16·1.5H$_2$O**.

Atom label	*x*	*y*	*z*	U_{iso} [Å^2]	Atom label	*x*	*y*	*z*	U_{iso} [Å^2]
O1	1.4903(2)	0.56724(19)	0.62678(19)	0.0289(5)	C5B	1.1628(3)	0.3934(2)	0.5776(2)	0.0196(5)
O2	1.6221(4)	0.4777(4)	0.8355(3)	0.0889(12)	C6B	1.1479(3)	0.3450(2)	0.3852(2)	0.0174(5)
O3	1.6168(2)	0.4254(2)	1.04458(19)	0.0344(5)	C7B	0.7128(3)	0.2508(3)	0.2466(2)	0.0214(6)
N1A	0.2298(2)	0.2352(2)	0.95590(19)	0.0247(5)	C8B	0.6847(4)	0.1272(3)	0.1711(3)	0.0378(8)
N2A	0.1677(2)	0.1559(2)	0.74948(19)	0.0189(5)	H1A	0.030(3)	0.115(3)	0.537(3)	0.032(8)
N3A	0.3495(2)	0.13302(19)	0.67982(19)	0.0186(5)	H2A	0.141(3)	0.085(3)	0.488(3)	0.026(8)
N4A	0.4094(2)	0.2068(2)	0.89324(19)	0.0214(5)	H3A	0.601(4)	0.151(3)	0.760(3)	0.046(10)
N5A	0.0711(2)	0.3389(2)	0.84123(19)	0.0203(5)	H4A	0.641(3)	0.197(3)	0.897(3)	0.031(9)
N6A	-0.1227(2)	0.3761(2)	0.88950(19)	0.0207(5)	H5A	-0.065(3)	0.407(3)	0.668(3)	0.039(9)
N7A	0.0548(2)	0.2845(2)	1.01612(19)	0.0208(5)	H6A	-0.183(4)	0.440(3)	0.703(3)	0.035(9)
N8A	0.1172(2)	0.1101(2)	0.5456(2)	0.0201(5)	H7A	-0.196(4)	0.373(3)	1.054(3)	0.036(9)
N9A	0.5781(2)	0.1646(2)	0.8231(2)	0.0261(5)	H8A	-0.086(3)	0.314(3)	1.133(3)	0.031(9)
N10A	-0.1050(3)	0.4166(2)	0.7170(2)	0.0258(5)	H9A	0.311(3)	0.282(3)	1.135(3)	0.025(8)
N11A	-0.1269(3)	0.3323(2)	1.0645(2)	0.0249(5)	H10A	0.407(4)	0.214(3)	1.085(3)	0.045(10)
N1B	0.8083(2)	0.2647(2)	0.37656(18)	0.0188(5)	H11A	0.315(5)	0.081(4)	1.172(5)	0.090(15)
N2B	0.8349(2)	0.16014(19)	0.52870(18)	0.0166(4)	H12A	0.145(6)	0.087(5)	1.087(5)	0.13(2)
N3B	0.6507(2)	0.1298(2)	0.59683(19)	0.0196(5)	H13A	0.252(5)	0.029(5)	1.034(5)	0.101(18)
N4B	0.6226(2)	0.2283(2)	0.43360(19)	0.0207(5)	H1B	0.952(3)	0.089(3)	0.707(3)	0.031(8)
N5B	1.0216(2)	0.35815(19)	0.53839(18)	0.0182(5)	H2B	0.832(3)	0.047(3)	0.738(3)	0.025(8)
N6B	1.2314(2)	0.39220(19)	0.50390(19)	0.0198(5)	H3B	0.415(3)	0.167(3)	0.549(3)	0.026(8)
N7B	1.0075(2)	0.29980(19)	0.33636(18)	0.0181(5)	H4B	0.398(4)	0.227(3)	0.444(3)	0.050(11)
N8B	0.8610(2)	0.0666(2)	0.6847(2)	0.0226(5)	H5B	1.197(3)	0.420(3)	0.744(3)	0.035(9)
N9B	0.4438(3)	0.1880(3)	0.4948(3)	0.0319(6)	H6B	1.330(4)	0.454(3)	0.729(3)	0.038(9)
N10B	1.2378(3)	0.4330(2)	0.6979(2)	0.0306(6)	H7B	1.303(4)	0.373(3)	0.338(3)	0.036(9)
N11B	1.2125(2)	0.3384(2)	0.3083(2)	0.0230(5)	H8B	1.158(3)	0.311(3)	0.228(3)	0.030(8)
C1A	0.2707(3)	0.1984(2)	0.8608(2)	0.0191(5)	H9B	0.756(3)	0.308(2)	0.217(2)	0.018(7)
C2A	0.2145(3)	0.1335(2)	0.6605(2)	0.0164(5)	H10B	0.626(3)	0.272(2)	0.246(2)	0.019(7)
C3A	0.4421(3)	0.1680(2)	0.7978(2)	0.0191(5)	H11B	0.621(4)	0.120(3)	0.086(3)	0.041(9)
C4A	0.1126(3)	0.2878(2)	0.9360(2)	0.0196(5)	H12B	0.775(4)	0.109(3)	0.175(3)	0.054(11)
C5A	-0.0513(3)	0.3760(2)	0.8190(2)	0.0182(5)	H13B	0.639(4)	0.072(3)	0.199(3)	0.054(11)
C6A	-0.0617(3)	0.3333(2)	0.9888(2)	0.0187(5)	H14	1.417(3)	0.501(3)	0.571(3)	0.074(13)
C7A	0.3103(3)	0.2126(4)	1.0773(3)	0.0386(9)	H15	1.454(4)	0.638(2)	0.611(3)	0.060(12)
C8A	0.2530(7)	0.0941(5)	1.0930(4)	0.0677(14)	H16	1.585(6)	0.507(5)	0.760(4)	0.14(2)
C1B	0.7537(3)	0.2160(2)	0.4511(2)	0.0176(5)	H17	1.703(5)	0.439(6)	0.844(6)	0.17(3)
C2B	0.7790(3)	0.1216(2)	0.6019(2)	0.0172(5)	H18	1.598(5)	0.467(4)	0.979(3)	0.084(15)
C3B	0.5749(3)	0.1807(2)	0.5085(2)	0.0202(5)	H19	1.541(3)	0.355(3)	1.003(3)	0.073(13)
C4B	0.9526(3)	0.3089(2)	0.4192(2)	0.0167(5)					

Table S31. Anisotropic atomic displacement parameters U_{ij} of all independent atoms of **16·1.5H$_2$O**.

Atom label	U_{11} [Å^2]	U_{22} [Å^2]	U_{33} [Å^2]	U_{23} [Å^2]	U_{13} [Å^2]	U_{12} [Å^2]
O1	0.0185(10)	0.0307(12)	0.0329(11)	0.0072(9)	0.0066(9)	0.0038(9)
O2	0.108(3)	0.144(3)	0.089(2)	0.085(2)	0.076(2)	0.090(3)
O3	0.0316(12)	0.0406(13)	0.0267(11)	0.0053(10)	0.0086(10)	0.0081(10)
N1A	0.0188(11)	0.0467(15)	0.0146(11)	0.0080(10)	0.0106(9)	0.0142(10)
N2A	0.0141(10)	0.0288(12)	0.0167(11)	0.0051(9)	0.0090(9)	0.0056(9)
N3A	0.0162(10)	0.0267(12)	0.0171(11)	0.0055(9)	0.0107(9)	0.0056(9)
N4A	0.0150(10)	0.0318(13)	0.0188(11)	0.0047(9)	0.0088(9)	0.0052(9)
N5A	0.0192(11)	0.0281(12)	0.0167(11)	0.0047(9)	0.0110(9)	0.0044(9)
N6A	0.0201(11)	0.0273(12)	0.0179(11)	0.0056(9)	0.0103(9)	0.0078(9)
N7A	0.0156(10)	0.0334(13)	0.0156(11)	0.0053(9)	0.0080(9)	0.0079(9)
N8A	0.0171(11)	0.0291(13)	0.0155(11)	0.0008(9)	0.0097(10)	0.0057(9)
N9A	0.0151(11)	0.0446(16)	0.0200(13)	0.0052(11)	0.0091(11)	0.0079(10)
N10A	0.0316(14)	0.0334(14)	0.0229(12)	0.0113(10)	0.0179(11)	0.0150(11)
N11A	0.0206(12)	0.0454(16)	0.0178(12)	0.0142(11)	0.0125(10)	0.0148(11)
N1B	0.0142(10)	0.0315(13)	0.0140(10)	0.0083(9)	0.0083(9)	0.0036(9)
N2B	0.0130(10)	0.0225(11)	0.0164(10)	0.0062(9)	0.0078(9)	0.0037(8)
N3B	0.0187(11)	0.0276(12)	0.0200(11)	0.0101(9)	0.0133(9)	0.0073(9)
N4B	0.0172(11)	0.0295(13)	0.0216(11)	0.0108(10)	0.0117(9)	0.0073(9)
N5B	0.0193(11)	0.0222(12)	0.0159(10)	0.0057(9)	0.0103(9)	0.0023(9)
N6B	0.0168(11)	0.0249(12)	0.0170(11)	0.0051(9)	0.0077(9)	-0.0013(9)
N7B	0.0140(10)	0.0272(12)	0.0150(10)	0.0058(9)	0.0077(9)	0.0044(9)
N8B	0.0163(12)	0.0369(14)	0.0215(12)	0.0162(10)	0.0109(10)	0.0075(10)
N9B	0.0226(13)	0.0551(18)	0.0397(15)	0.0323(14)	0.0236(12)	0.0195(12)
N10B	0.0247(14)	0.0432(16)	0.0168(12)	0.0003(11)	0.0086(11)	-0.0092(11)
N11B	0.0139(11)	0.0382(15)	0.0168(12)	0.0053(10)	0.0074(10)	0.0036(10)
C1A	0.0182(13)	0.0262(14)	0.0170(13)	0.0074(11)	0.0099(11)	0.0073(10)
C2A	0.0163(12)	0.0140(12)	0.0193(13)	0.0039(10)	0.0080(10)	0.0021(9)
C3A	0.0171(12)	0.0216(14)	0.0206(13)	0.0057(10)	0.0097(11)	0.0040(10)
C4A	0.0135(12)	0.0291(15)	0.0143(12)	0.0007(10)	0.0067(10)	0.0011(10)
C5A	0.0218(13)	0.0164(13)	0.0151(12)	0.0004(10)	0.0086(11)	0.0010(10)
C6A	0.0154(12)	0.0238(14)	0.0152(12)	0.0005(10)	0.0068(10)	0.0015(10)
C7A	0.0260(16)	0.082(3)	0.0161(14)	0.0127(16)	0.0114(13)	0.0298(17)
C8A	0.113(4)	0.074(3)	0.046(2)	0.037(2)	0.043(3)	0.060(3)
C1B	0.0152(12)	0.0229(14)	0.0154(12)	0.0027(10)	0.0086(10)	0.0012(10)
C2B	0.0168(12)	0.0186(13)	0.0168(12)	0.0036(10)	0.0082(10)	0.0020(10)
C3B	0.0183(13)	0.0229(14)	0.0234(13)	0.0079(11)	0.0117(11)	0.0052(10)
C4B	0.0180(12)	0.0192(13)	0.0164(12)	0.0078(10)	0.0089(10)	0.0060(10)
C5B	0.0219(13)	0.0175(13)	0.0177(13)	0.0031(10)	0.0085(11)	-0.0019(10)
C6B	0.0180(12)	0.0217(13)	0.0173(12)	0.0089(10)	0.0098(11)	0.0069(10)
C7B	0.0140(13)	0.0387(17)	0.0159(12)	0.0125(12)	0.0079(11)	0.0063(11)
C8B	0.041(2)	0.041(2)	0.0224(16)	0.0048(14)	0.0065(15)	0.0021(16)

Table S32. Fractional atomic coordinates and isotropic atomic displacement parameters U_{iso} of all independent atoms of **17**.

Atom label	*x*	*y*	*z*	U_{iso} [Å^2]
N1	0.34481(14)	0.5654(2)	0.71419(10)	0.0165(3)
N2	0.16296(15)	0.4232(2)	0.74155(10)	0.0170(3)
N3	0.16658(15)	0.3658(2)	0.90315(11)	0.0180(3)
N4	0.35510(15)	0.4940(2)	0.86993(10)	0.0167(3)
N5	0.16428(15)	0.6764(2)	0.59131(10)	0.0164(3)
N6	0.16456(15)	0.6391(2)	0.42932(10)	0.0179(3)
N7	0.35263(15)	0.5505(2)	0.55866(10)	0.0173(3)
N8	-0.01773(16)	0.3143(3)	0.77663(12)	0.0227(4)
N9	0.35453(17)	0.4283(3)	1.02278(11)	0.0246(4)
N10	-0.01597(16)	0.7511(3)	0.46431(12)	0.0201(4)
N11	0.35063(17)	0.5312(3)	0.40246(12)	0.0216(4)
N12	0.58738(16)	0.5910(3)	0.77035(11)	0.0273(4)
C1	0.28200(17)	0.4885(3)	0.77905(12)	0.0146(4)
C2	0.10605(18)	0.3683(3)	0.80852(12)	0.0162(4)
C3	0.28760(18)	0.5767(3)	0.46436(12)	0.0164(4)
C4	0.10613(18)	0.6877(3)	0.49467(12)	0.0158(4)
C5	0.29065(18)	0.4270(3)	0.92986(13)	0.0171(4)
C6	0.28165(17)	0.5998(3)	0.61428(12)	0.0146(4)
C7	0.47594(18)	0.5771(3)	0.74509(12)	0.0193(4)
H1	-0.064(2)	0.330(4)	0.7131(18)	0.025(6)
H2	-0.056(2)	0.269(4)	0.8180(17)	0.029(6)
H3	0.313(2)	0.383(4)	1.0633(18)	0.034(7)
H4	0.443(3)	0.450(4)	1.0470(18)	0.034(7)
H5	-0.057(2)	0.780(4)	0.5062(18)	0.027(6)
H6	-0.060(2)	0.735(4)	0.4026(18)	0.028(6)
H7	0.306(2)	0.537(4)	0.3388(18)	0.029(6)
H8	0.438(3)	0.503(4)	0.4214(18)	0.038(7)

Table S33. Anisotropic atomic displacement parameters U_{ij} of all independent atoms of **17**.

Atom label	U_{11} [Å^2]	U_{22} [Å^2]	U_{33} [Å^2]	U_{23} [Å^2]	U_{13} [Å^2]	U_{12} [Å^2]
N1	0.0130(7)	0.0243(8)	0.0123(7)	0.0020(6)	0.0043(6)	-0.0017(6)
N2	0.0162(8)	0.0211(8)	0.0139(7)	0.0014(6)	0.0048(6)	-0.0013(6)
N3	0.0167(8)	0.0228(8)	0.0146(7)	0.0011(6)	0.0052(6)	-0.0022(6)
N4	0.0170(8)	0.0192(8)	0.0142(7)	0.0005(6)	0.0053(6)	-0.0017(6)
N5	0.0165(8)	0.0195(8)	0.0141(7)	0.0006(6)	0.0059(6)	0.0004(6)
N6	0.0175(8)	0.0238(8)	0.0130(7)	0.0005(6)	0.0055(6)	0.0009(6)
N7	0.0166(8)	0.0204(8)	0.0152(7)	0.0009(6)	0.0054(6)	0.0009(6)
N8	0.0171(8)	0.0365(10)	0.0139(8)	0.0033(7)	0.0036(6)	-0.0078(7)
N9	0.0211(9)	0.0397(11)	0.0119(8)	0.0029(7)	0.0035(6)	-0.0087(8)
N10	0.0164(8)	0.0296(9)	0.0141(8)	0.0015(7)	0.0047(6)	0.0042(7)
N11	0.0199(9)	0.0323(10)	0.0141(8)	0.0010(7)	0.0073(6)	0.0059(7)
N12	0.0190(9)	0.0497(12)	0.0138(8)	-0.0010(7)	0.0060(6)	-0.0046(8)
C1	0.0169(9)	0.0148(8)	0.0134(8)	0.0011(7)	0.0067(7)	0.0018(7)
C2	0.0178(9)	0.0163(9)	0.0145(8)	0.0012(7)	0.0048(7)	0.0003(7)
C3	0.0183(9)	0.0171(9)	0.0146(8)	0.0000(7)	0.0064(7)	-0.0002(7)
C4	0.0177(9)	0.0159(9)	0.0142(8)	0.0004(7)	0.0057(7)	-0.0019(7)
C5	0.0175(9)	0.0184(9)	0.0152(8)	0.0000(7)	0.0048(7)	-0.0006(7)
C6	0.0159(8)	0.0153(8)	0.0124(8)	0.0010(7)	0.0043(6)	-0.0018(7)
C8	0.0192(10)	0.0274(10)	0.0120(8)	0.0009(7)	0.0059(7)	0.0000(8)

Table S34. Fractional atomic coordinates and isotropic atomic displacement parameters U_{iso} of all independent atoms of **18·2H$_2$O**.

Atom label	*x*	*y*	*z*	U_{iso} [Å^2]
O1[a]	0.4794(3)	0.8029(3)	0.7931(5)	0.0607(13)
O2[a]	0.5063(3)	0.9321(3)	0.5388(4)	0.0553(10)
N1	0.500000	0.76219(15)	0.250000	0.0158(4)
N2	0.60310(10)	0.91475(11)	0.23097(15)	0.0176(3)
N3	0.75201(10)	0.92139(11)	0.37071(14)	0.0182(3)
N4	0.65119(10)	0.76001(11)	0.36761(15)	0.0193(3)
N5	0.56292(9)	0.59862(11)	0.16135(14)	0.0156(3)
N6	0.500000	0.42886(15)	0.250000	0.0173(4)
N7	0.70055(12)	1.06795(12)	0.23800(19)	0.0257(4)
N8	0.80016(12)	0.76588(13)	0.48753(18)	0.0273(4)
N9	0.61539(11)	0.43679(12)	0.06695(16)	0.0208(3)
C1	0.58917(11)	0.81617(12)	0.28505(16)	0.0150(3)
C2	0.68516(11)	0.96554(13)	0.28186(17)	0.0172(3)
C3	0.73334(12)	0.81710(13)	0.40557(17)	0.0182(3)
C4	0.500000	0.64534(17)	0.250000	0.0136(4)
C5	0.55788(10)	0.48752(13)	0.16218(17)	0.0158(3)
H1	0.654(2)	1.101(2)	0.188(3)	0.052(8)
H2	0.762(2)	1.101(2)	0.260(3)	0.055(8)
H3	0.8505(19)	0.804(2)	0.528(3)	0.036(6)
H4	0.7908(18)	0.695(2)	0.509(3)	0.043(7)
H5	0.6569(18)	0.478(2)	0.005(2)	0.033(6)
H6	0.6067(18)	0.367(2)	0.050(3)	0.038(6)

[a] These atom positions are only half occupied.

Table S35. Anisotropic atomic displacement parameters U_{ij} of all independent atoms of **18·2H$_2$O**.

Atom label	U_{11} [Å^2]	U_{22} [Å^2]	U_{33} [Å^2]	U_{23} [Å^2]	U_{13} [Å^2]	U_{12} [Å^2]
O1	0.052(3)	0.0318(15)	0.098(4)	-0.0016(18)	-0.019(2)	0.0145(15)
O2	0.061(2)	0.072(2)	0.0329(17)	-0.0208(16)	0.0098(16)	-0.0308(19)
N1	0.0138(8)	0.0114(8)	0.0220(9)	0.000	-0.0028(7)	0.000
N2	0.0146(6)	0.0149(6)	0.0233(7)	0.0036(5)	-0.0004(5)	-0.0004(5)
N3	0.0164(6)	0.0168(7)	0.0215(7)	-0.0003(5)	-0.0009(5)	-0.0027(5)
N4	0.0190(7)	0.0142(6)	0.0248(7)	0.0023(5)	-0.0064(5)	-0.0023(5)
N5	0.0142(6)	0.0135(6)	0.0191(6)	-0.0011(5)	0.0003(5)	-0.0007(5)
N6	0.0153(9)	0.0129(8)	0.0239(10)	0.000	-0.0002(7)	0.000
N7	0.0202(7)	0.0163(7)	0.0405(9)	0.0071(6)	-0.0008(6)	-0.0033(5)
N8	0.0270(8)	0.0188(7)	0.0360(9)	0.0033(6)	-0.0174(7)	-0.0030(6)
N9	0.0198(7)	0.0175(7)	0.0251(7)	-0.0049(6)	0.0021(6)	0.0002(5)
C1	0.0143(7)	0.0139(7)	0.0169(7)	-0.0013(5)	0.0003(5)	0.0005(5)
C2	0.0148(7)	0.0156(7)	0.0213(8)	-0.0002(6)	0.0043(6)	0.0007(6)
C3	0.0178(7)	0.0177(8)	0.0191(7)	-0.0012(6)	-0.0030(6)	-0.0004(6)
C4	0.0112(9)	0.0134(10)	0.0161(10)	0.000	-0.0040(7)	0.000
C5	0.0123(7)	0.0156(7)	0.0195(7)	-0.0017(6)	-0.0029(6)	-0.0004(5)

Table S36. Fractional atomic coordinates and isotropic atomic displacement parameters U_{iso} of all independent atoms of **19**.

Atom label	*x*	*y*	*z*	U_{iso} [Å^2]
O1	0.500000	0.1516(3)	0.250000	0.0347(5)
N1	0.61690(14)	0.4490(3)	0.29889(11)	0.0309(4)
N2	0.65985(13)	0.4991(3)	0.47515(11)	0.0278(4)
N3	0.54293(13)	0.1915(3)	0.41469(11)	0.0273(4)
N4	0.72992(16)	0.7396(3)	0.36061(14)	0.0375(5)
N5	0.58665(15)	0.2429(3)	0.58297(13)	0.0305(4)
C1	0.55566(17)	0.2746(3)	0.32564(14)	0.0279(4)
C2	0.66775(15)	0.5590(3)	0.37868(13)	0.0277(4)
C3	0.59820(15)	0.3141(3)	0.48944(13)	0.0255(4)
H1	0.737(2)	0.770(4)	0.296(2)	0.058(8)
H2	0.7656(19)	0.814(4)	0.4121(18)	0.034(6)
H3	0.6109(18)	0.316(4)	0.6285(18)	0.027(6)
H4	0.5479(19)	0.120(4)	0.5887(16)	0.031(6)

Table S37. Anisotropic atomic displacement parameters U_{ij} of all independent atoms of **19**.

Atom label	U_{11} [Å^2]	U_{22} [Å^2]	U_{33} [Å^2]	U_{23} [Å^2]	U_{13} [Å^2]	U_{12} [Å^2]
O1	0.0583(14)	0.0252(10)	0.0201(9)	0.000	-0.0005(9)	0.000
N1	0.0414(10)	0.0300(9)	0.0220(8)	0.0009(7)	0.0077(7)	0.0002(7)
N2	0.0267(8)	0.0329(9)	0.0241(8)	0.0035(7)	0.0035(6)	0.0008(7)
N3	0.0346(9)	0.0265(8)	0.0211(8)	0.0018(6)	0.0039(6)	0.0029(7)
N4	0.0456(11)	0.0417(11)	0.0257(9)	0.0030(8)	0.0062(8)	-0.0108(9)
N5	0.0362(9)	0.0340(10)	0.0210(8)	0.0047(7)	0.0010(7)	-0.0048(8)
C1	0.0360(10)	0.0247(10)	0.0236(9)	-0.0011(7)	0.0054(8)	0.0067(8)
C2	0.0279(9)	0.0306(10)	0.0255(9)	0.0016(8)	0.0076(7)	0.0041(8)
C3	0.0235(9)	0.0295(10)	0.0237(9)	0.0029(8)	0.0029(7)	0.0071(8)

Table S38. Fractional atomic coordinates and isotropic atomic displacement parameters U_{iso} of all independent atoms of **20·3H$_2$O**.

Atom label	*x*	*y*	*z*	U_{iso} [Å^2]	Atom label	*x*	*y*	*z*	U_{iso} [Å^2]
Cu1	0.26281(9)	0.51812(3)	0.34341(7)	0.01845(16)	C6A	0.5661(7)	0.4040(2)	0.7408(5)	0.0257(10)
Cl1	0.69624(16)	0.63242(5)	0.34666(13)	0.0270(3)	C7A	0.2783(7)	0.3279(2)	0.3503(5)	0.0282(11)
Cl2	-0.15136(18)	0.40760(5)	0.34869(14)	0.0313(3)	C8A	0.4409(10)	0.2950(2)	0.3467(7)	0.0463(16)
O1	0.1691(9)	0.25912(19)	0.9863(6)	0.0559(13)	C1B	0.2614(6)	0.62378(18)	0.4826(5)	0.0184(8)
O2	0.4693(6)	0.26214(16)	0.8933(5)	0.0401(9)	C2B	0.2434(7)	0.55233(19)	0.6324(5)	0.0209(9)
O3	0.8408(7)	0.26506(18)	1.1199(6)	0.0500(11)	C3B	0.3860(7)	0.63117(18)	0.7385(5)	0.0224(9)
O4	0.5494(5)	0.59348(17)	0.3402(4)	0.0313(8)	C4B	0.1170(6)	0.62466(18)	0.2010(5)	0.0180(8)
O5	0.7273(6)	0.6280(2)	0.2084(5)	0.0421(10)	C5B	-0.0403(6)	0.55172(18)	0.0532(5)	0.0201(9)
O6	0.8613(5)	0.62015(19)	0.4773(5)	0.0407(9)	C6B	-0.0170(6)	0.63194(18)	-0.0570(5)	0.0213(9)
O7	0.6366(7)	0.68709(17)	0.3622(6)	0.0468(11)	C7B	0.2394(7)	0.7103(2)	0.3463(5)	0.0261(11)
O8	-0.0311(7)	0.4521(2)	0.3456(4)	0.0527(13)	C8B	0.0919(8)	0.7413(2)	0.3817(6)	0.0350(12)
O9A[a]	-0.3153(15)	0.4215(6)	0.3462(17)	0.077(4)	H1A	0.366194	0.545405	-0.033765	0.032
O9B[a]	-0.1925(16)	0.4158(5)	0.4836(12)	0.057(3)	H2A	0.408901	0.553558	0.135282	0.032
O10A[a]	-0.1637(13)	0.3783(3)	0.2125(9)	0.044(2)	H3A	0.078272	0.384711	-0.2627	0.05
O10B[a]	-0.3225(13)	0.4126(5)	0.2186(12)	0.060(3)	H4A	0.063307	0.337274	-0.162996	0.05
O11A[a]	-0.0344(15)	0.3787(4)	0.4785(9)	0.051(2)	H5A	0.73153	0.547893	0.71967	0.03
O11B[a]	-0.0879(16)	0.3518(4)	0.3580(14)	0.062(3)	H6A	0.609965	0.555593	0.551281	0.03
N1A	0.3091(6)	0.38836(15)	0.3473(4)	0.0197(8)	H7A	0.672779	0.387047	0.954512	0.045
N2A	0.2997(6)	0.46652(14)	0.1961(4)	0.0196(8)	H8A	0.555235	0.340342	0.856947	0.045
N3A	0.2356(7)	0.45279(17)	-0.0669(5)	0.0275(9)	H9A	0.163997	0.31773	0.26156	0.034
N4A	0.2132(6)	0.37784(15)	0.0880(4)	0.0228(8)	H10A	0.257144	0.318556	0.443776	0.034
N5A	0.3657(6)	0.53291(16)	0.0530(4)	0.0268(9)	H11A	0.413956	0.255627	0.348841	0.069
N6A	0.0989(8)	0.37108(19)	-0.1719(5)	0.0417(13)	H12A	0.460862	0.303485	0.253252	0.069
N7A	0.4447(5)	0.46764(14)	0.4940(4)	0.0193(8)	H13A	0.554022	0.304311	0.435492	0.069
N8A	0.6391(6)	0.45463(16)	0.7560(4)	0.0260(9)	H1B	0.18179	0.491286	0.732257	0.035
N9A	0.4619(6)	0.37991(16)	0.6056(4)	0.0240(8)	H2B	0.119687	0.483455	0.560756	0.035
N10A	0.6479(6)	0.53505(16)	0.6339(4)	0.0249(9)	H3B	0.524018	0.646854	0.95306	0.039
N11A	0.6020(7)	0.37376(19)	0.8646(5)	0.0378(12)	H4B	0.536249	0.693079	0.848821	0.039
N1B	0.2083(6)	0.64943(14)	0.3417(4)	0.0193(8)	H5B	-0.191306	0.489032	-0.040601	0.033
N2B	0.2307(5)	0.56960(15)	0.4922(4)	0.0192(8)	H6B	-0.083548	0.482422	0.130501	0.033
N3B	0.3254(6)	0.58213(16)	0.7601(4)	0.0238(8)	H7B	-0.094337	0.64718	-0.27049	0.034
N4B	0.3443(6)	0.65616(15)	0.6015(4)	0.0214(8)	H8B	0.009486	0.694919	-0.168971	0.034
N5B	0.1739(7)	0.50362(17)	0.6430(5)	0.0288(10)	H9B	0.237598	0.722641	0.24649	0.031
N6B	0.4946(7)	0.66041(18)	0.8609(5)	0.0322(10)	H10B	0.364596	0.718846	0.42471	0.031
N7B	0.0869(5)	0.56955(15)	0.1932(4)	0.0195(8)	H11B	0.117101	0.780925	0.383706	0.053
N8B	-0.0869(6)	0.58107(15)	-0.0746(4)	0.0229(8)	H12B	-0.032081	0.733503	0.303215	0.053
N9B	0.0670(6)	0.65748(15)	0.0806(4)	0.0217(8)	H13B	0.094867	0.72971	0.481366	0.053
N10B	-0.1131(6)	0.50224(16)	0.0470(5)	0.0274(9)	H14	0.073(7)	0.237(2)	0.963(8)	0.045(19)
N11B	-0.0361(6)	0.66128(18)	-0.1791(5)	0.0286(9)	H15	0.16(2)	0.285(4)	1.043(13)	0.14(5)
C1A	0.2740(6)	0.41180(17)	0.2062(5)	0.0183(8)	H16	0.375(6)	0.260(2)	0.915(7)	0.025(15)
C2A	0.3004(7)	0.48336(18)	0.0596(5)	0.0210(9)	H17	0.429(11)	0.241(3)	0.818(7)	0.06(2)
C3A	0.1842(8)	0.4017(2)	-0.0476(5)	0.0280(10)	H18	0.726(5)	0.262(3)	1.066(8)	0.06(2)
C4A	0.4094(6)	0.41317(17)	0.4868(5)	0.0198(9)	H19	0.839(11)	0.2968(17)	1.157(8)	0.05(2)
C5A	0.5791(7)	0.48482(19)	0.6291(5)	0.0213(9)					

[a] These atom positions are only half occupied.

Table S39. Anisotropic atomic displacement parameters U_{ij} of all independent atoms of **20·3H$_2$O**.

Atom label	U_{11} [Å^2]	U_{22} [Å^2]	U_{33} [Å^2]	U_{23} [Å^2]	U_{13} [Å^2]	U_{12} [Å^2]
Cu1	0.0266(3)	0.0139(3)	0.0102(3)	-0.0003(2)	0.0019(2)	0.0009(2)
Cl1	0.0271(5)	0.0286(6)	0.0240(6)	0.0032(4)	0.0084(4)	-0.0007(4)
Cl2	0.0346(6)	0.0315(6)	0.0262(6)	-0.0044(4)	0.0099(5)	-0.0036(5)
O1	0.090(4)	0.035(2)	0.058(3)	-0.015(2)	0.044(3)	-0.021(2)
O2	0.040(2)	0.028(2)	0.043(2)	0.0025(17)	0.0059(19)	-0.0061(17)
O3	0.043(2)	0.033(2)	0.057(3)	-0.009(2)	0.001(2)	0.0087(19)
O4	0.0258(16)	0.042(2)	0.0245(18)	0.0014(15)	0.0074(14)	-0.0073(15)
O5	0.045(2)	0.061(3)	0.027(2)	0.0065(19)	0.0209(18)	0.000(2)
O6	0.0275(18)	0.056(3)	0.030(2)	0.0046(19)	0.0015(16)	-0.0030(18)
O7	0.059(3)	0.029(2)	0.058(3)	0.0028(19)	0.029(2)	0.0041(19)
O8	0.059(3)	0.070(3)	0.023(2)	0.0010(19)	0.009(2)	-0.029(2)
O9A	0.045(6)	0.095(9)	0.101(11)	-0.022(8)	0.039(7)	-0.003(6)
O9B	0.065(6)	0.074(7)	0.048(6)	-0.008(5)	0.039(5)	-0.011(5)
O10A	0.067(6)	0.033(4)	0.020(4)	-0.012(3)	0.003(4)	-0.001(4)
O10B	0.032(4)	0.083(8)	0.046(6)	-0.003(5)	-0.007(4)	-0.014(5)
O11A	0.080(7)	0.047(5)	0.014(4)	0.008(3)	0.005(4)	-0.007(5)
O11B	0.063(6)	0.037(5)	0.084(8)	0.001(5)	0.025(6)	0.002(4)
N1A	0.0250(18)	0.0170(19)	0.0109(18)	0.0010(13)	0.0000(14)	-0.0020(15)
N2A	0.029(2)	0.0143(17)	0.0139(18)	-0.0012(13)	0.0067(16)	-0.0010(14)
N3A	0.046(2)	0.0204(19)	0.0144(18)	-0.0014(15)	0.0092(18)	-0.0050(17)
N4A	0.034(2)	0.0185(18)	0.0118(18)	-0.0011(14)	0.0041(16)	-0.0008(16)
N5A	0.045(2)	0.023(2)	0.0124(18)	-0.0047(14)	0.0107(18)	-0.0109(17)
N6A	0.080(4)	0.024(2)	0.012(2)	-0.0035(17)	0.007(2)	-0.018(2)
N7A	0.027(2)	0.0141(17)	0.0127(18)	-0.0003(13)	0.0028(15)	-0.0011(14)
N8A	0.033(2)	0.0215(19)	0.0143(18)	0.0005(15)	-0.0007(16)	-0.0064(16)
N9A	0.033(2)	0.0183(18)	0.0118(18)	0.0020(14)	-0.0012(16)	-0.0039(16)
N10A	0.030(2)	0.0211(19)	0.0124(18)	0.0044(13)	-0.0047(16)	-0.0077(15)
N11A	0.055(3)	0.026(2)	0.014(2)	0.0061(17)	-0.0076(19)	-0.014(2)
N1B	0.0256(19)	0.0134(19)	0.0137(19)	-0.0007(13)	0.0020(15)	-0.0032(14)
N2B	0.0265(19)	0.0188(18)	0.0112(18)	-0.0005(14)	0.0059(15)	-0.0026(15)
N3B	0.035(2)	0.0231(19)	0.0103(17)	-0.0015(14)	0.0053(16)	-0.0028(16)
N4B	0.029(2)	0.0163(17)	0.0151(18)	-0.0017(14)	0.0038(15)	-0.0021(15)
N5B	0.043(2)	0.028(2)	0.0134(19)	-0.0039(14)	0.0085(18)	-0.0145(18)
N6B	0.050(3)	0.024(2)	0.015(2)	-0.0049(16)	0.0048(19)	-0.0071(19)
N7B	0.0242(19)	0.0166(18)	0.0123(18)	-0.0004(14)	0.0011(15)	-0.0023(14)
N8B	0.028(2)	0.0194(18)	0.0124(18)	0.0024(14)	-0.0014(15)	-0.0044(15)
N9B	0.029(2)	0.0158(17)	0.0151(18)	0.0003(14)	0.0035(16)	-0.0004(15)
N10B	0.036(2)	0.022(2)	0.0139(19)	0.0002(14)	-0.0027(17)	-0.0067(16)
N11B	0.043(2)	0.0209(19)	0.0155(19)	0.0012(15)	0.0044(17)	-0.0062(17)
C1A	0.023(2)	0.015(2)	0.014(2)	-0.0021(15)	0.0034(17)	0.0002(16)
C2A	0.034(2)	0.018(2)	0.011(2)	-0.0037(16)	0.0083(19)	-0.0009(18)
C3A	0.044(3)	0.022(2)	0.016(2)	-0.0052(18)	0.009(2)	-0.007(2)
C4A	0.023(2)	0.017(2)	0.015(2)	0.0025(16)	0.0026(17)	0.0000(17)
C5A	0.022(2)	0.021(2)	0.013(2)	0.0031(16)	-0.0016(17)	0.0017(17)
C6A	0.033(2)	0.023(2)	0.014(2)	0.0013(17)	0.0025(19)	-0.0051(19)
C7A	0.041(3)	0.019(3)	0.015(2)	0.0007(16)	0.000(2)	-0.008(2)
C8A	0.060(4)	0.021(3)	0.037(3)	-0.002(2)	-0.005(3)	0.009(2)

PXRD Data

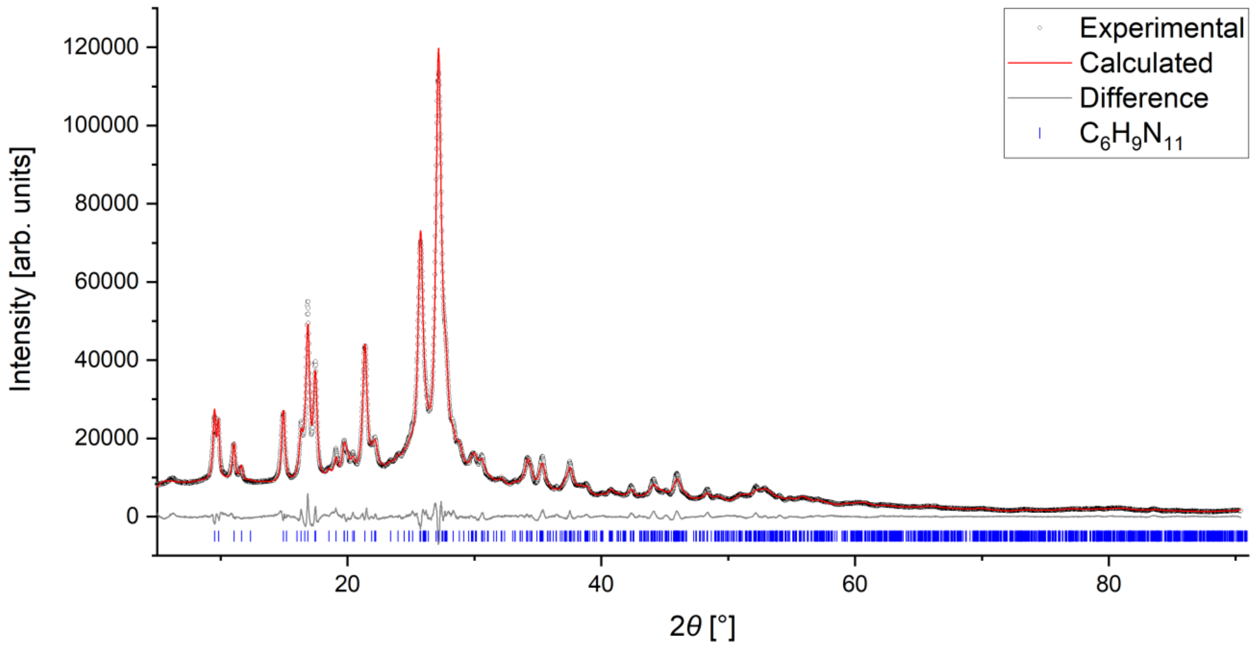

Figure S1. Rietveld refinement plot of the PXRD pattern obtained from synthesized melam (**1**). The resulting difference plot (grey line) is depicted below the experimental (black circles) and calculated (red line) PXRD pattern. Blue lines mark the positions of the Bragg reflections of melam (**1**) [12].

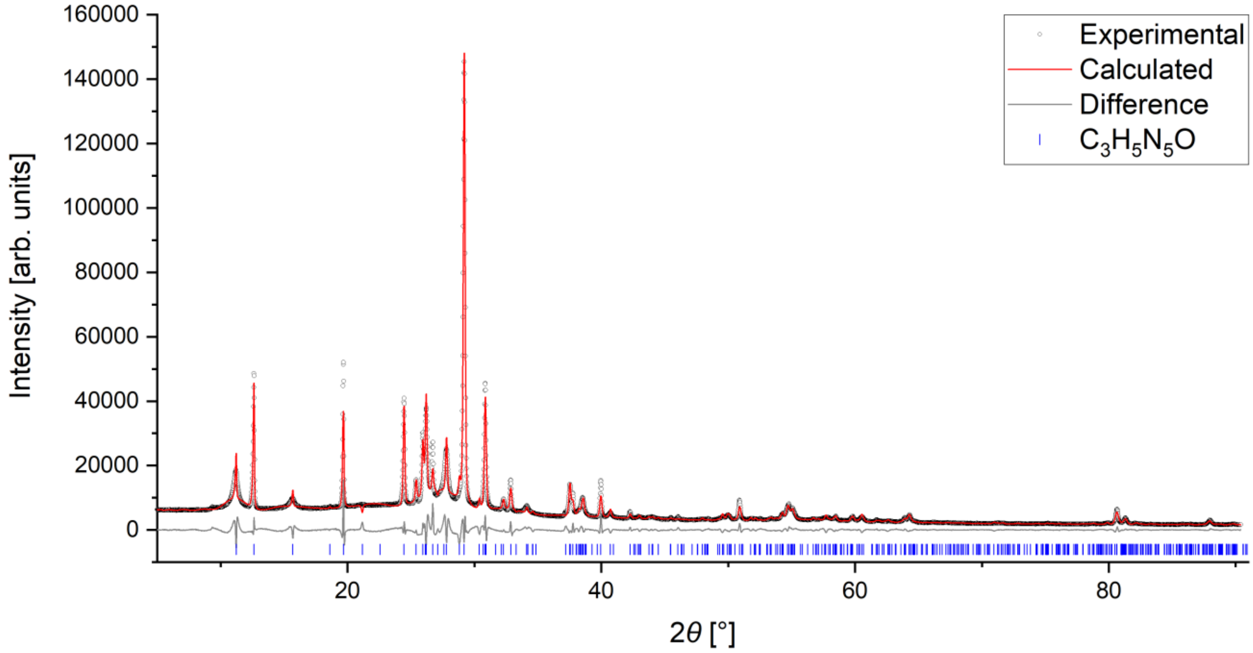

Figure S2. Rietveld refinement plot of the PXRD pattern obtained from synthesized ammeline (**3**). The resulting difference plot (grey line) is depicted below the experimental (black circles) and calculated (red line) PXRD pattern. Blue lines mark the positions of the Bragg reflections of ammeline (**3**) [13].

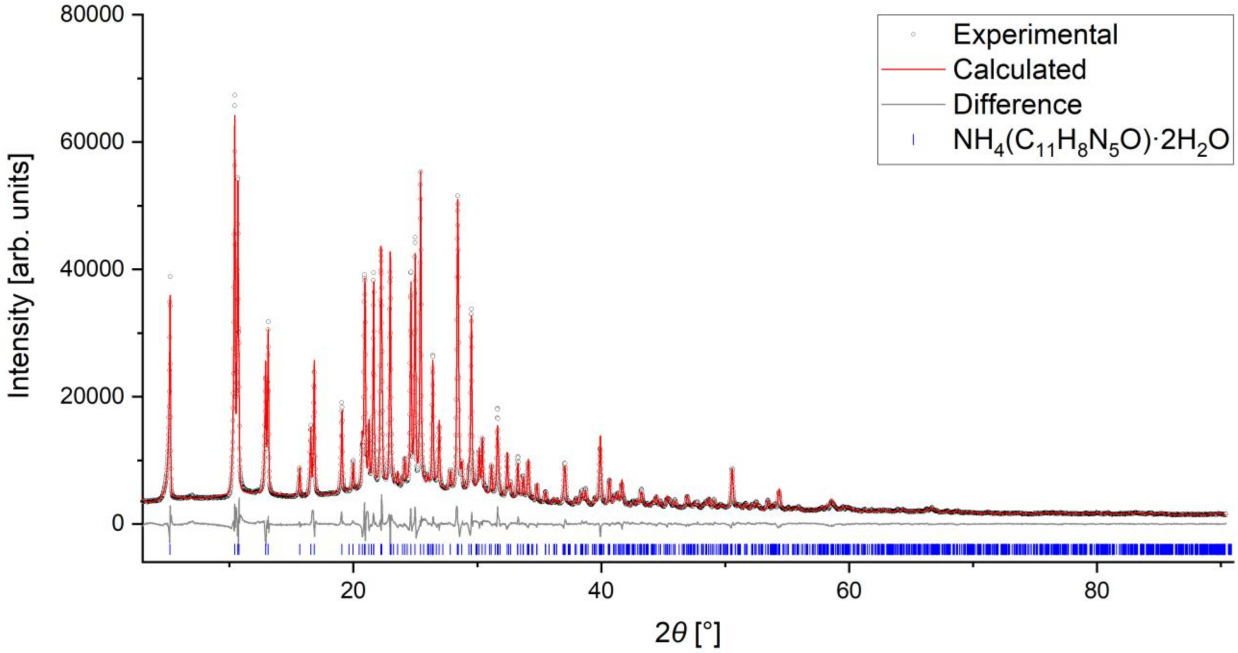

Figure S3. Rietveld refinement plot of the PXRD pattern obtained from synthesized **6·2H$_2$O**. The resulting difference plot (grey line) is depicted below the experimental (black circles) and calculated (red line) PXRD pattern. Blue lines mark the positions of the Bragg reflections of **6·2H$_2$O**.

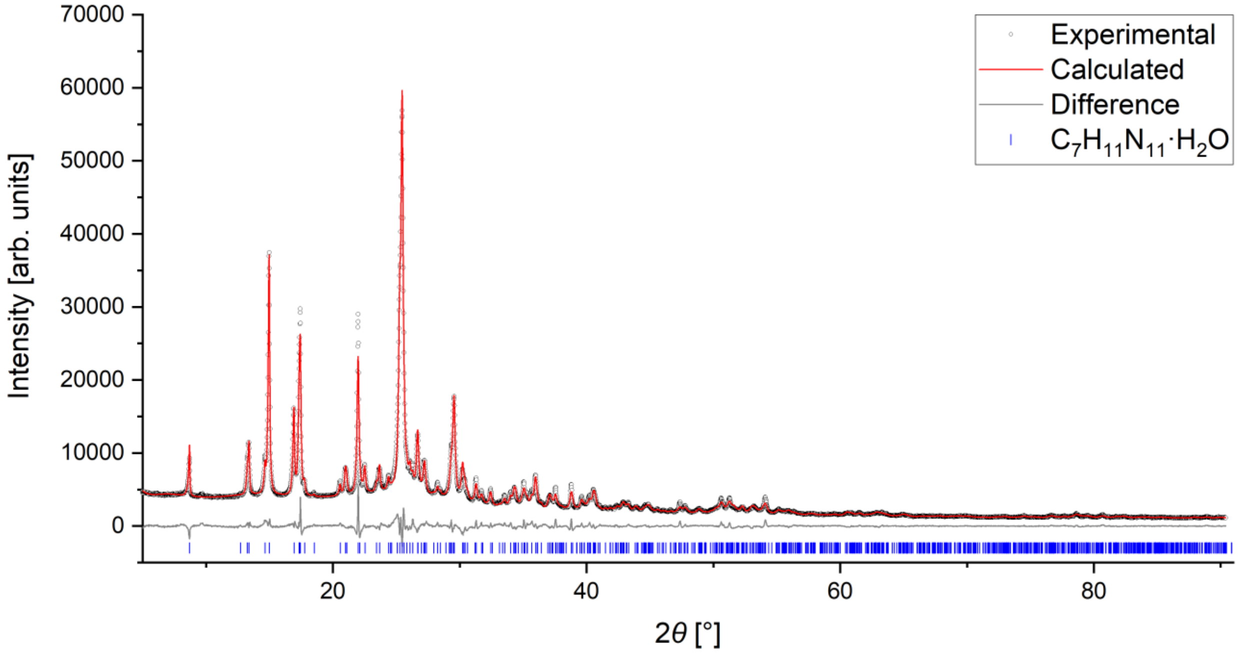

Figure S4. Rietveld refinement plot of the PXRD pattern obtained from synthesized **15·H$_2$O**. The resulting difference plot (grey line) is depicted below the experimental (black circles) and calculated (red line) PXRD pattern. Blue lines mark the positions of the Bragg reflections of **15·H$_2$O**.

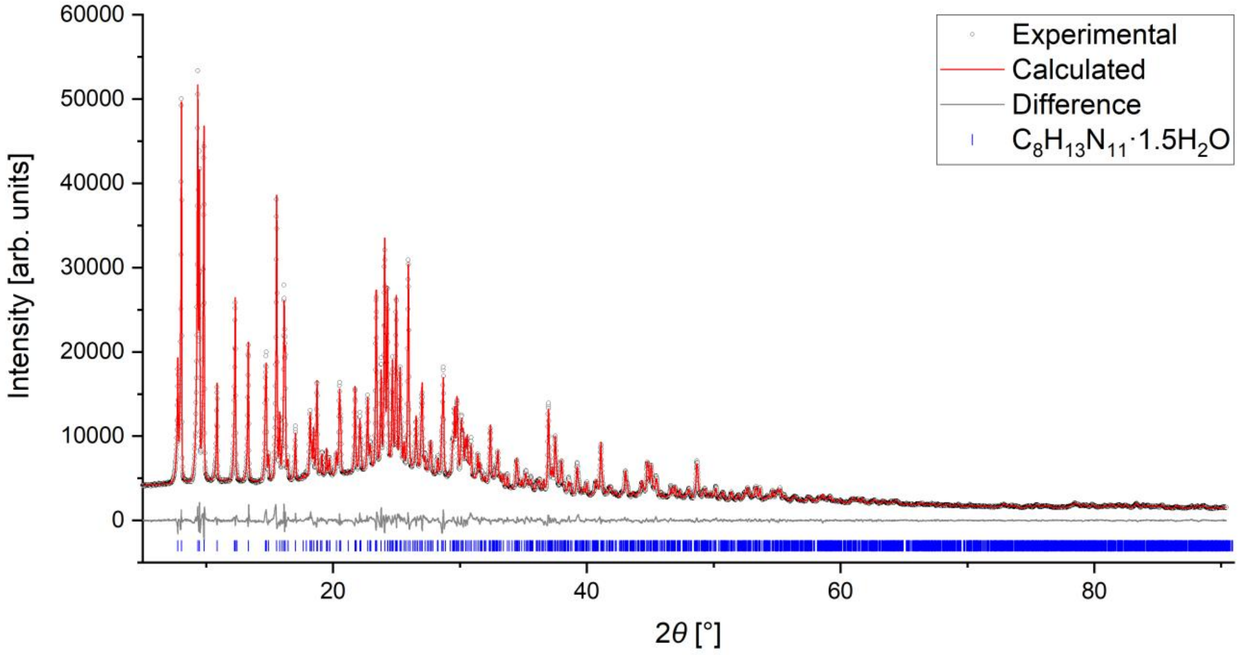

Figure S5. Rietveld refinement plot of the PXRD pattern obtained from synthesized **16·1.5H$_2$O**. The resulting difference plot (grey line) is depicted below the experimental (black circles) and calculated (red line) PXRD pattern. Blue lines mark the positions of the Bragg reflections of **16·1.5H$_2$O**.

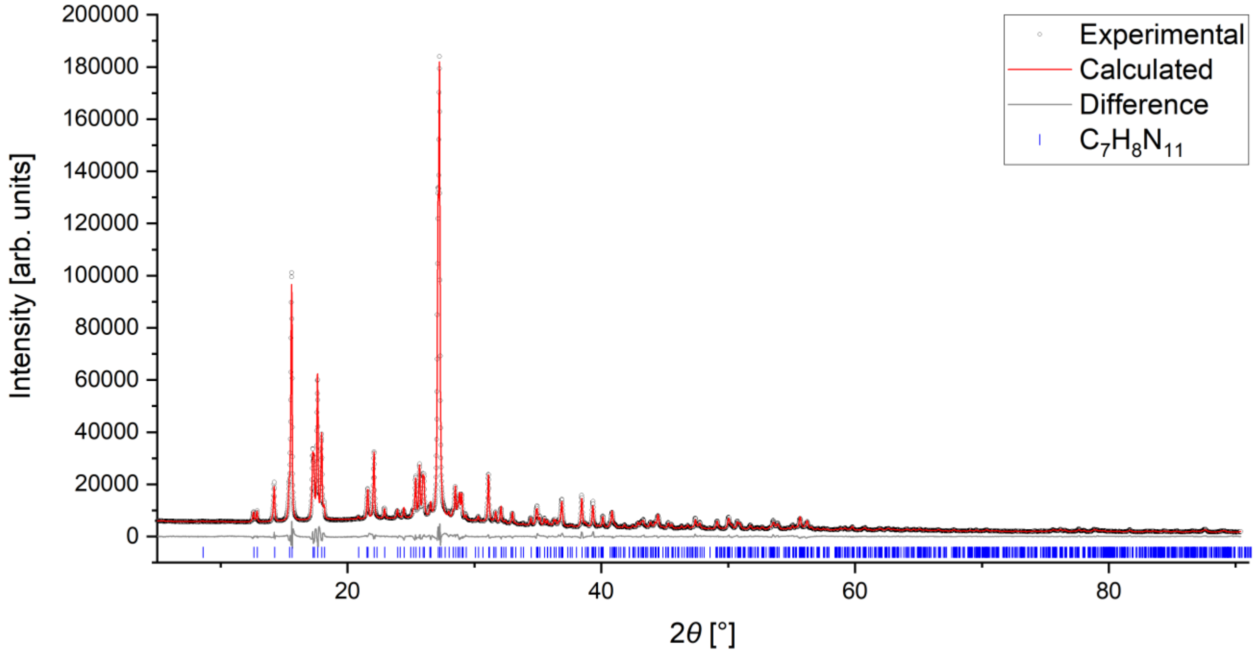

Figure S6. Rietveld refinement plot of the PXRD pattern obtained from synthesized **17**. The resulting difference plot (grey line) is depicted below the experimental (black circles) and calculated (red line) PXRD pattern. Blue lines mark the positions of the Bragg reflections of **17**.

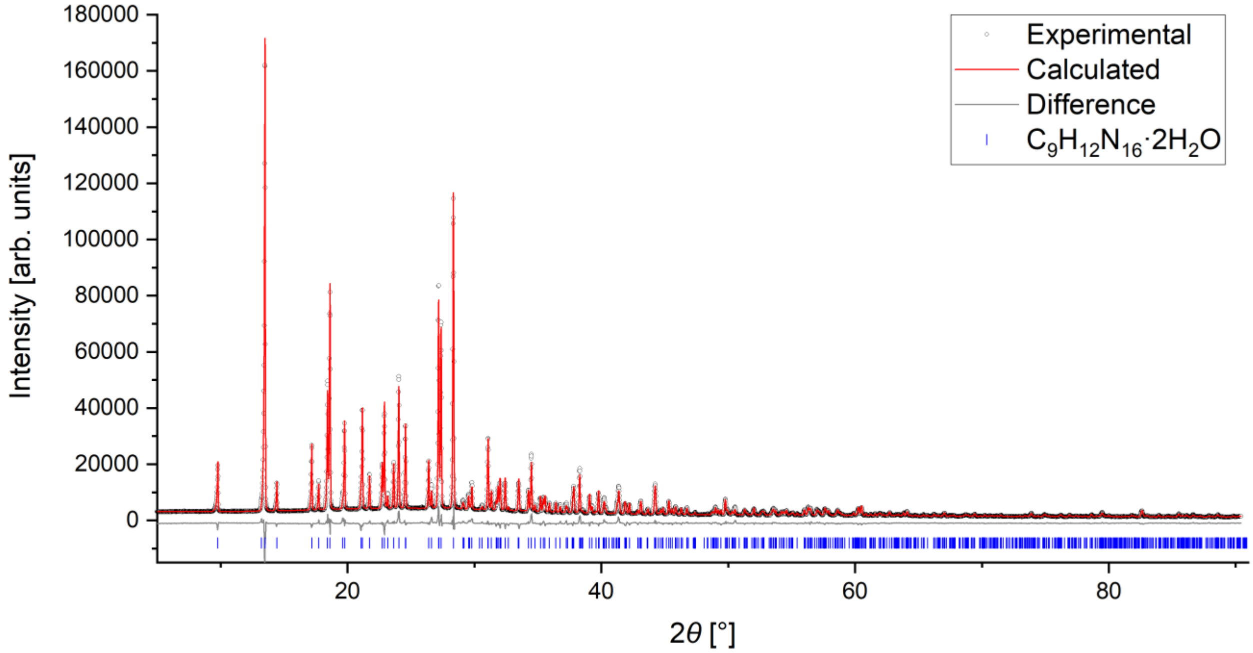

Figure S7. Rietveld refinement plot of the PXRD pattern obtained from synthesized **18·2H$_2$O**. The resulting difference plot (grey line) is depicted below the experimental (black circles) and calculated (red line) PXRD pattern. Blue lines mark the positions of the Bragg reflections of **18·2H$_2$O**.

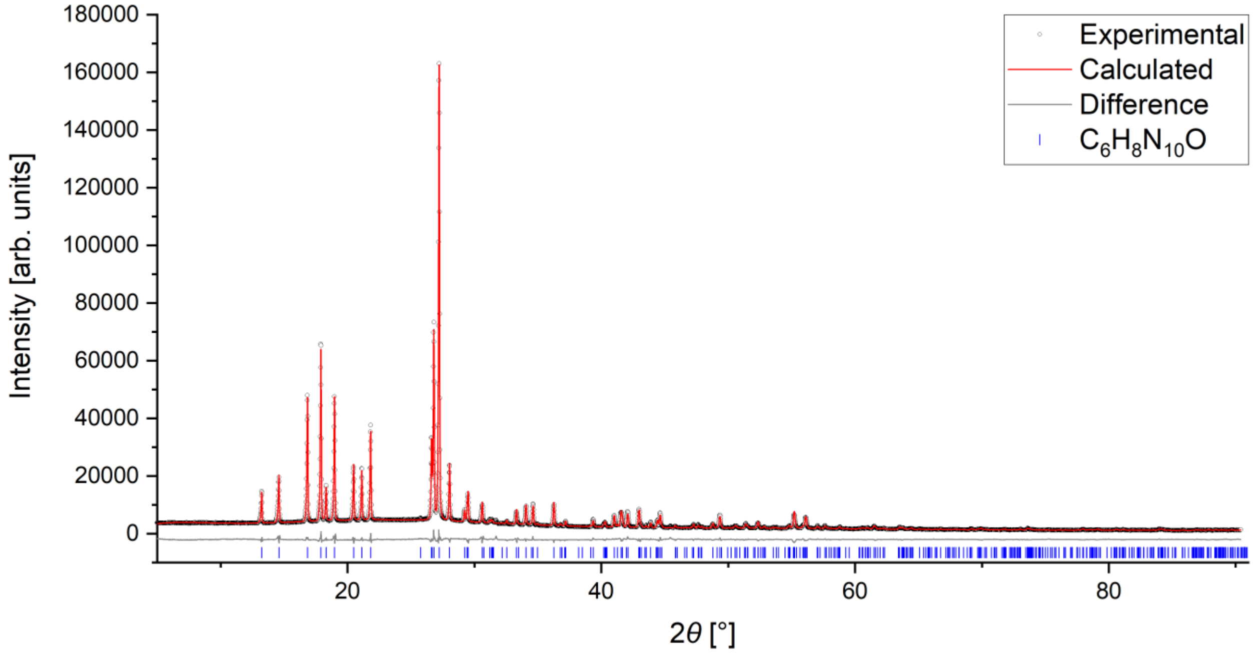

Figure S8. Rietveld refinement plot of the PXRD pattern obtained from synthesized **19**. The resulting difference plot (grey line) is depicted below the experimental (black circles) and calculated (red line) PXRD pattern. Blue lines mark the positions of the Bragg reflections of **19**.

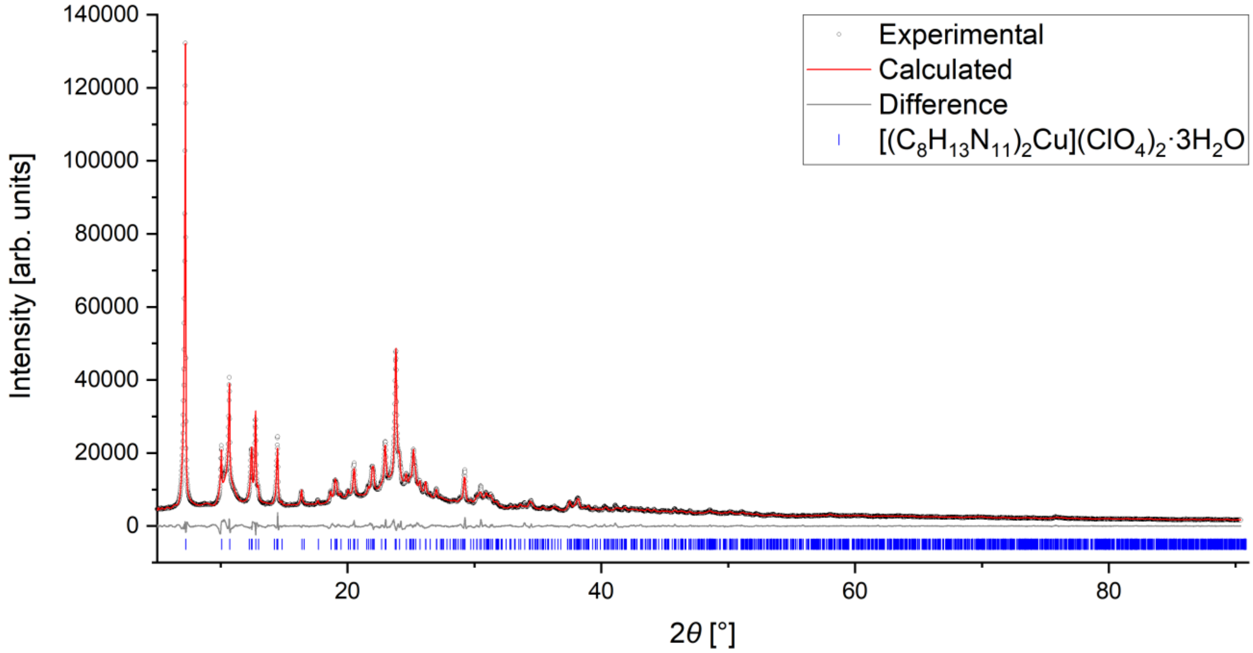

Figure S9. Rietveld refinement plot of the PXRD pattern obtained from synthesized **20·3H$_2$O**. The resulting difference plot (grey line) is depicted below the experimental (black circles) and calculated (red line) PXRD pattern. Blue lines mark the positions of the Bragg reflections of **20·3H$_2$O**.

Table S40. Determined lattice parameters and cell volumes *V* of the different compounds at 20 °C by Rietveld refinement.

Phase	*a* [Å]	*b* [Å]	*c* [Å]	*α* [°]	*β* [°]	*γ* [°]	*V* [Å^3]
**1**	18.1038(15)	10.8434(9)	14.4135(7)	90	96.185(5)	90	2813.0(4)
**3**	3.67640(15)	9.5271(4)	14.1188(9)	90	96.811(3)	90	491.03(4)
**6·2H$_2$O**	34.8361(8)	4.69329(7)	17.0113(3)	90	103.1492(15)	90	2708.36(9)
**15·H$_2$O**	10.5799(5)	7.5107(3)	14.4857(7)	90	106.171(3)	90	1105.53(9)
**16·1.5H$_2$O**	10.48067(10)	11.78375(14)	12.21958(14)	92.2761(7)	96.957(4)	92.2761(7)	492.50(7)
**17**	10.7868(2)	6.93088(10)	14.7511(2)	90	108.0284(13)	90	1048.68(3)
**18·2H$_2$O**	13.38914(11)	12.26967(14)	9.52825(10)	90	90	90	1565.30(3)
**19**	12.17325(11)	5.83772(7)	13.41075(17)	90	94.3499(8)	90	950.276(19)
**20·3H$_2$O**	7.84065(17)	24.4080(12)	9.5263(4)	90	112.902(4)	90	1679.39(13)

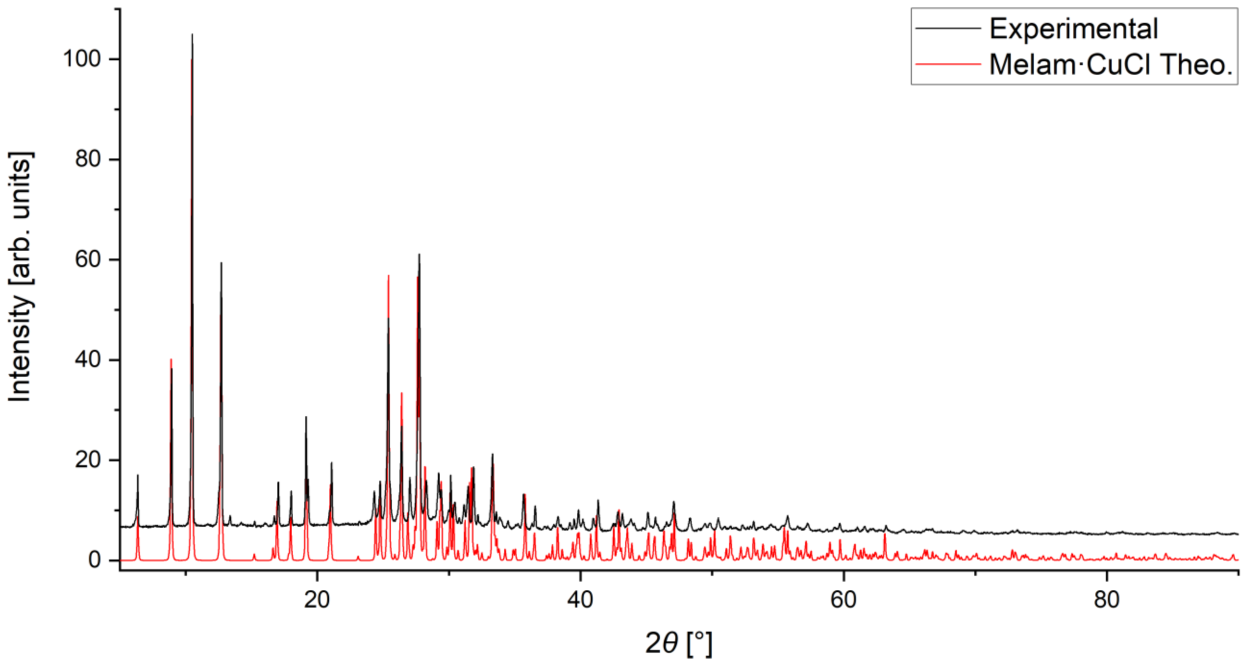

Figure S10. Experimental PXRD pattern (black) of the product obtained from the reaction of melamine with 0.50 eq. of $CuCl_2$ using the same reaction setup and conditions as in the reported synthesis of **1·CuCl**[14] in comparison to the theoretical pattern (red) determined from the reported structural data of **1·CuCl**[14].

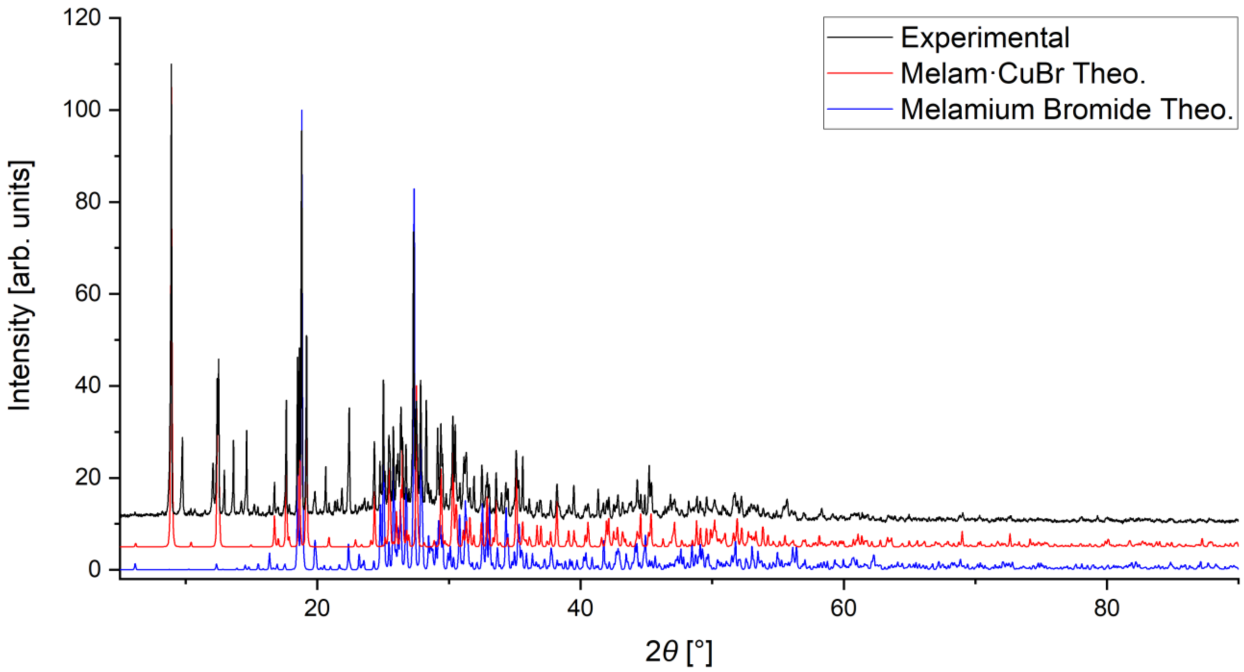

Figure S11. Experimental PXRD pattern (black) of the product obtained from the reaction of melamine with 0.50 eq. of $CuBr_2$ using the same reaction setup and conditions as in the reported synthesis of **1·CuBr**[14] in comparison to the theoretical patterns determined from the reported structural data of **1·CuBr**[14] (red) and **1·HBr**[15] (blue).

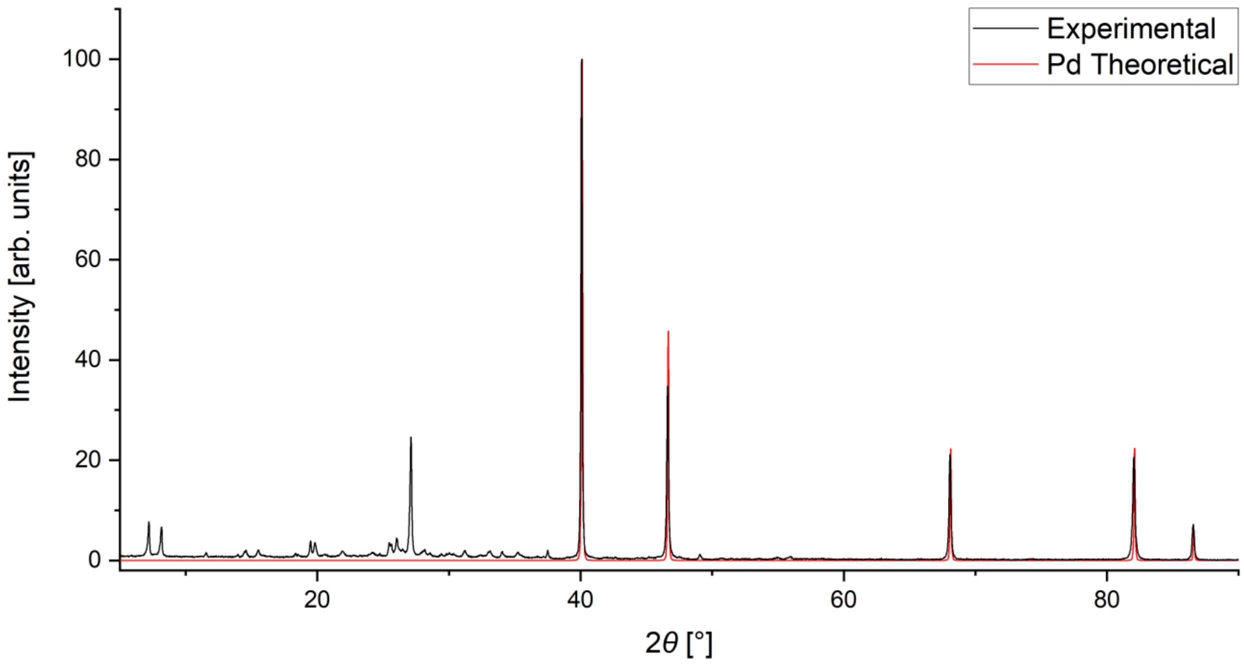

Figure S12. Experimental PXRD pattern (black) of the product obtained from the reaction of melamine with 0.50 eq. of $PdCl_2$ using the same reaction setup as in the reported synthesis of **1·CuCl**[14] but with a slightly altered furnace program (i.e. a heating/cooling rate of 1 °C/min and a reaction time of 12 h at 370 °C) in comparison to the theoretical pattern (red) determined from the reported structural data of elemental Pd[16].

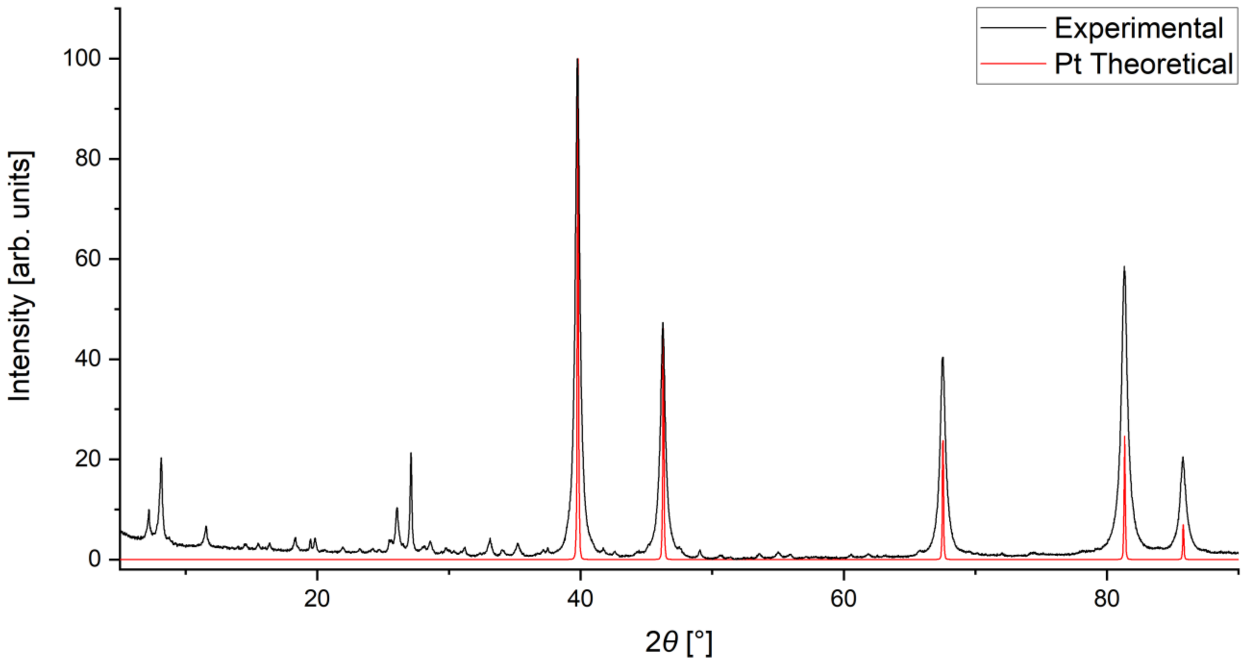

Figure S13. Experimental PXRD pattern (black) of the product obtained from the reaction of melamine with 0.50 eq. of $PtCl_2$ using the same reaction setup as in the reported synthesis of **1·CuCl**[14] but with a slightly altered furnace program (i.e. a heating/cooling rate of 1 °C/min and a reaction time of 12 h at 370 °C) in comparison to the theoretical pattern (red) determined from the reported structural data of elemental Pt[17].

NMR Spectra

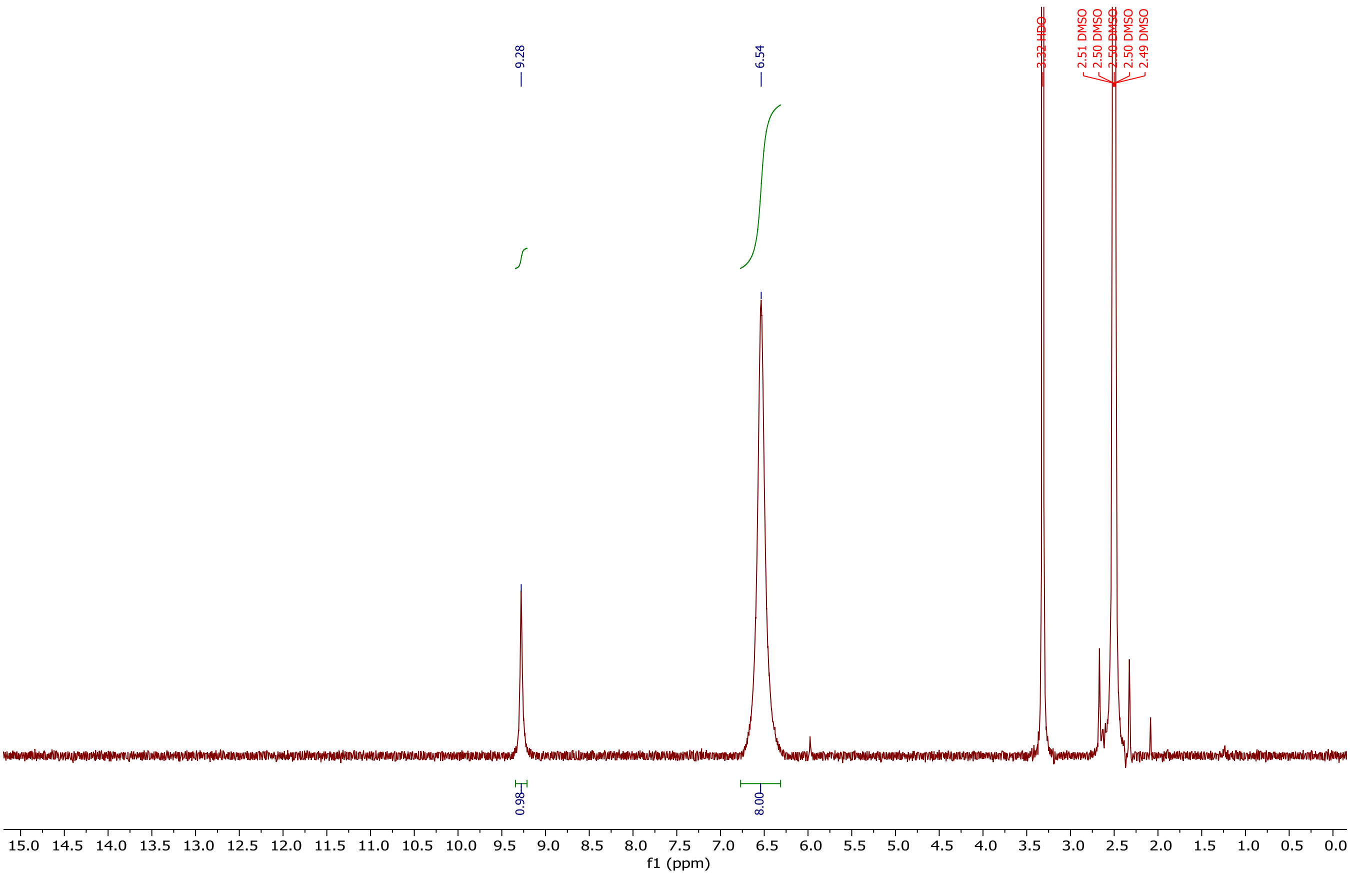

Figure S14. ^{1}H-NMR spectrum of a sample of **1** dissolved in DMSO-d_6.

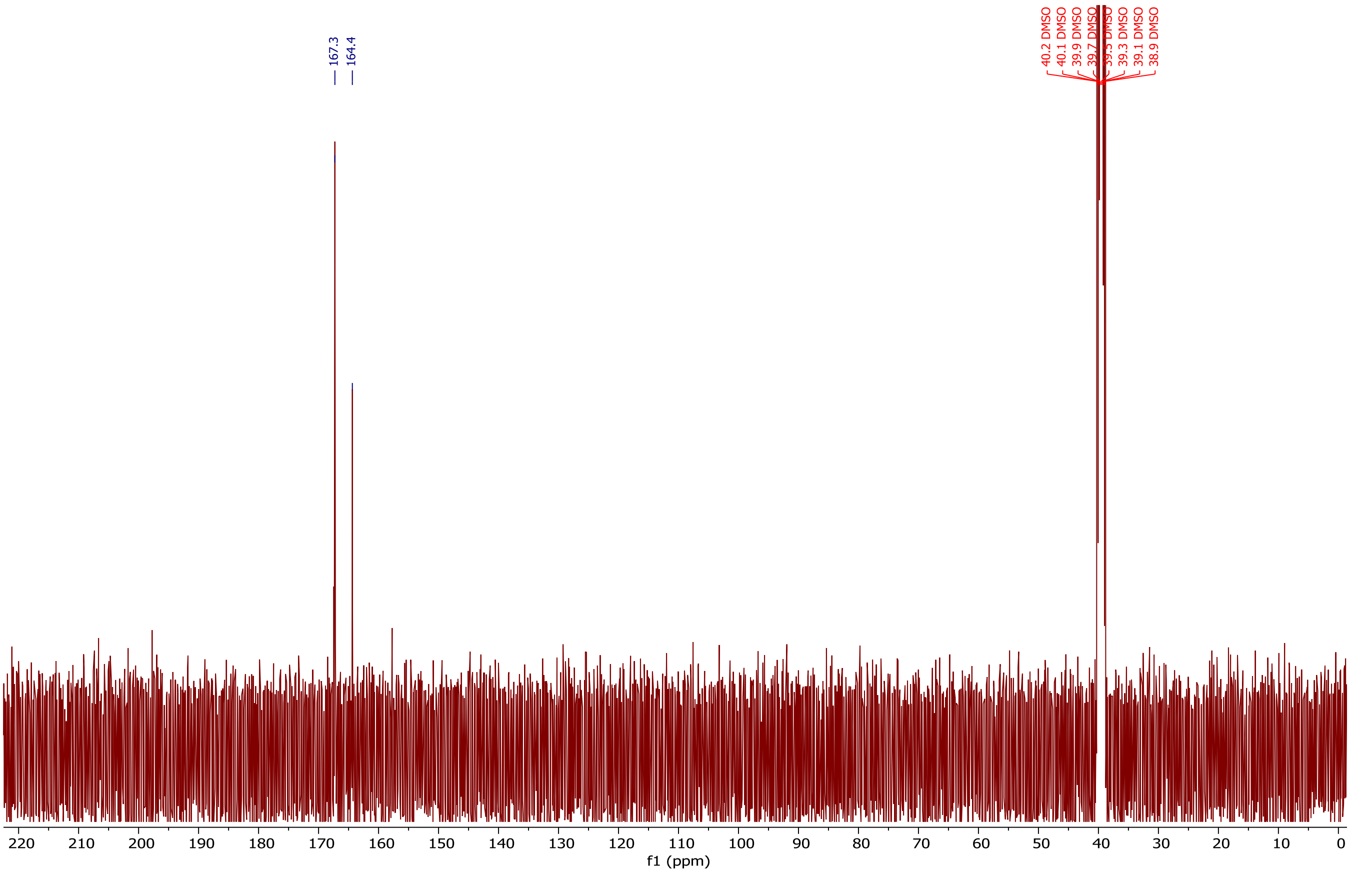

Figure S15. ^{13}C-NMR spectrum of a sample of **1** dissolved in DMSO-d_6.

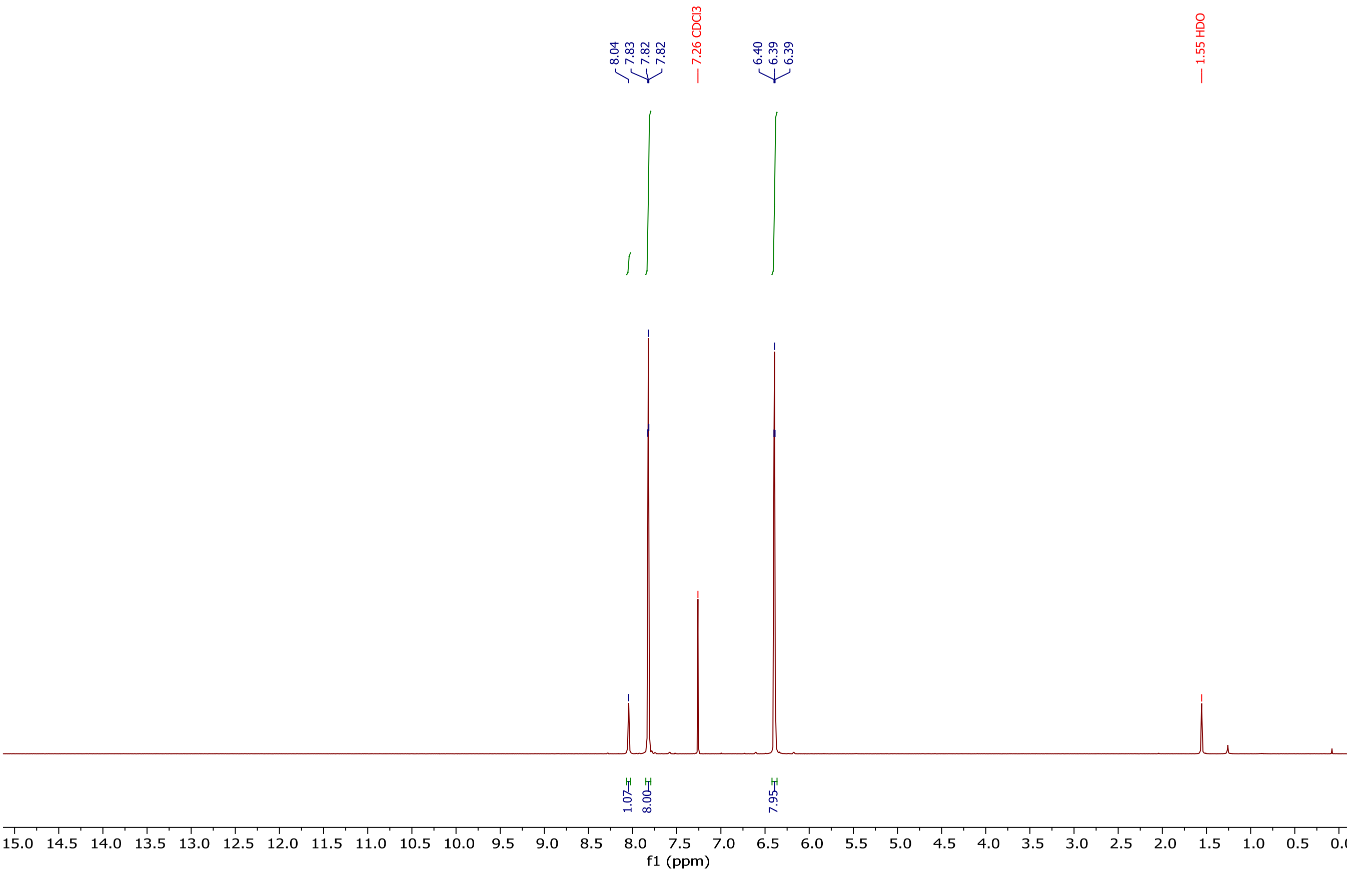

Figure S16. ^{1}H-NMR spectrum of a sample of **2** dissolved in $CDCl_3$.

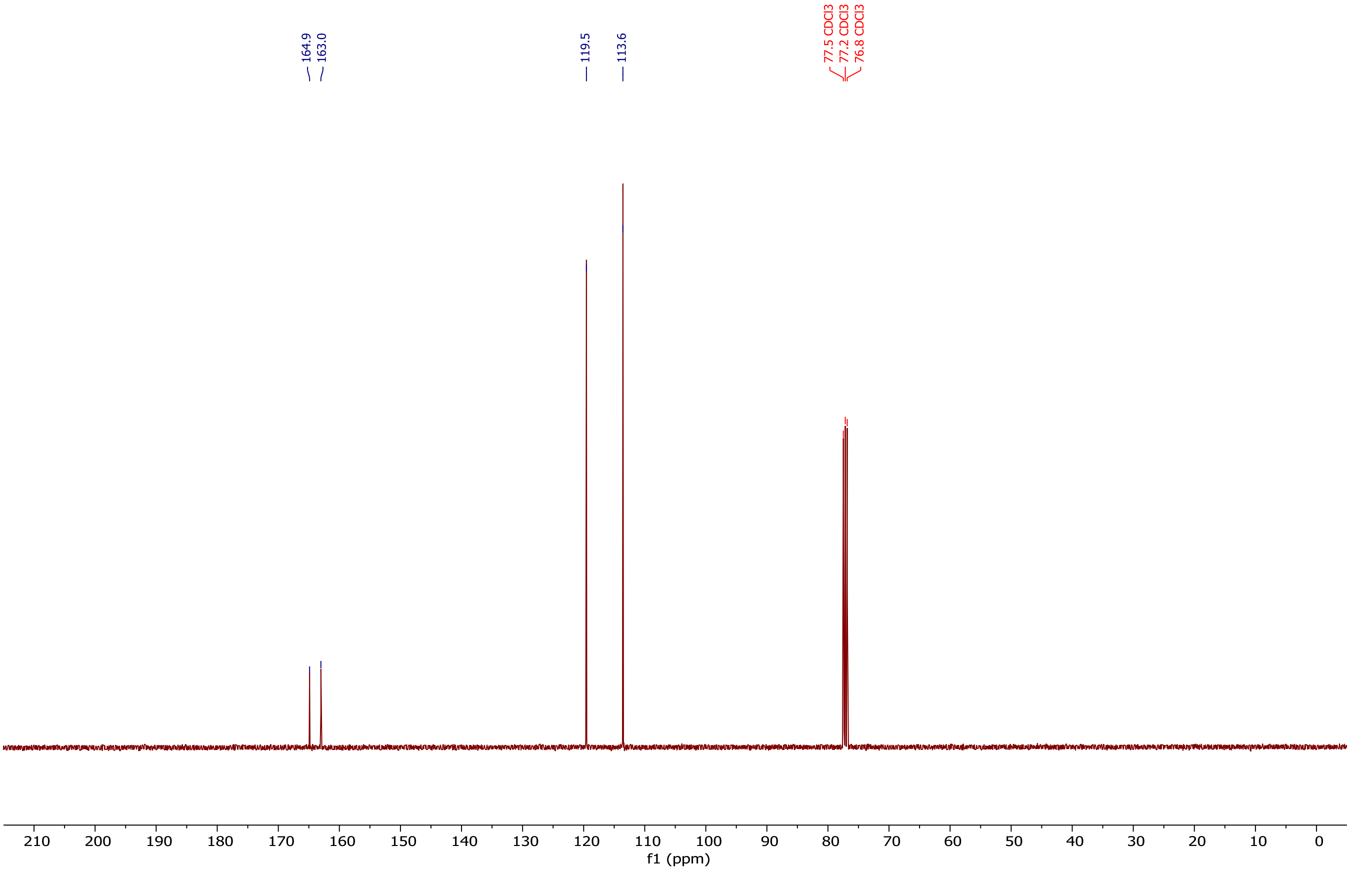

Figure S17. ^{13}C-NMR spectrum of a sample of **2** dissolved in $CDCl_3$.

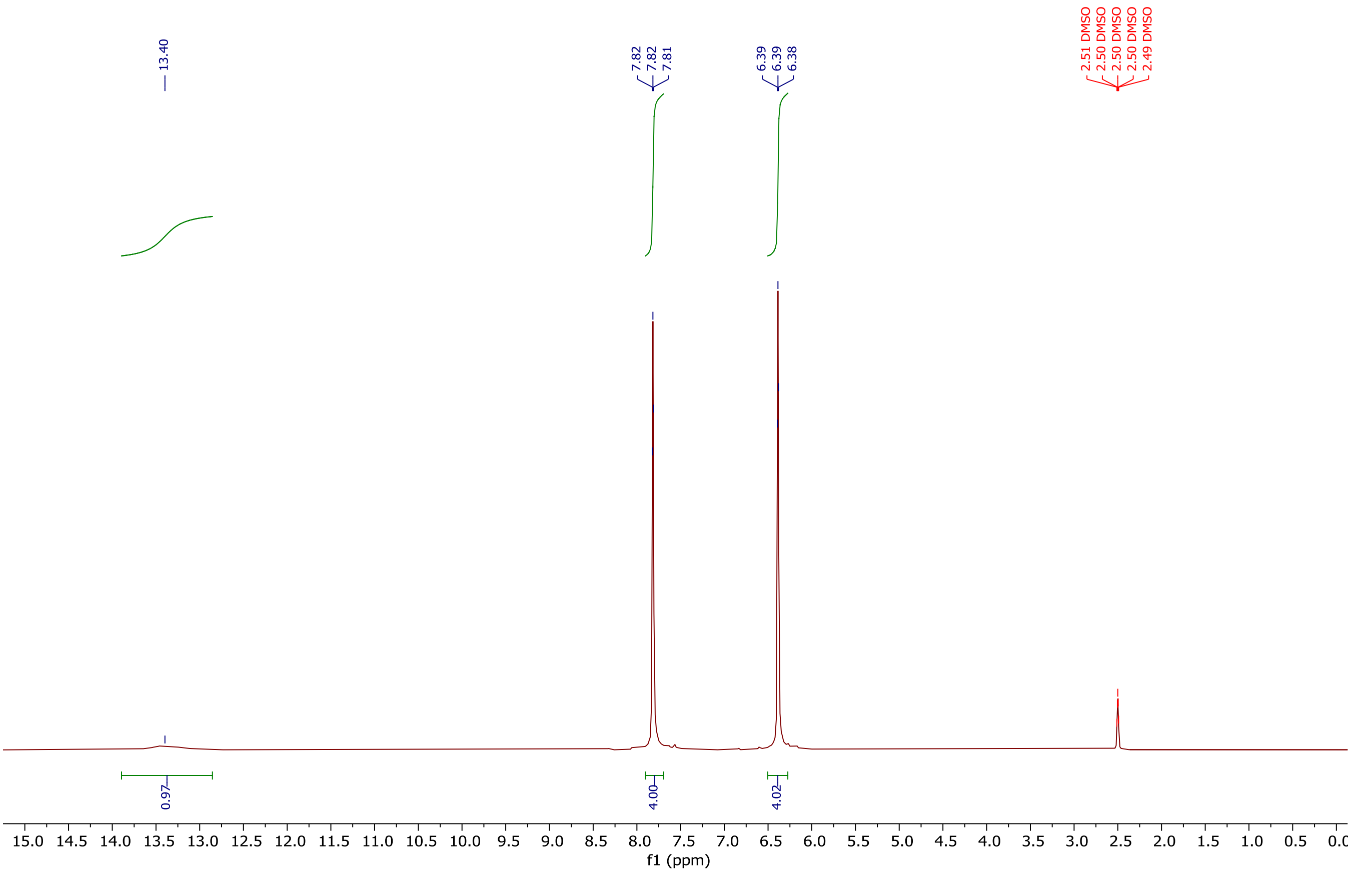

Figure S18. ^{1}H-NMR spectrum of a sample of **4** dissolved in DMSO-d_6.

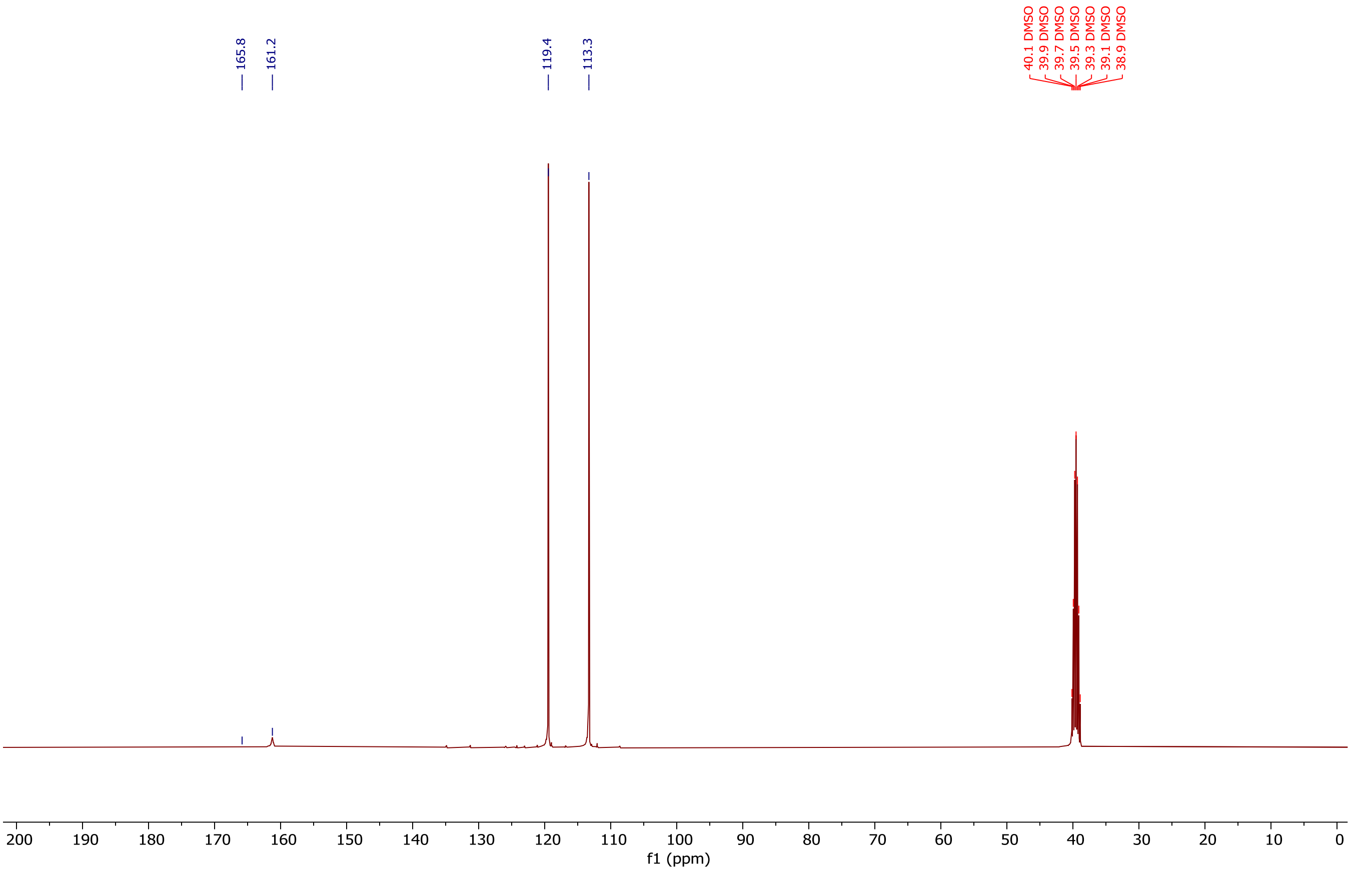

Figure S19. ^{13}C-NMR spectrum of a sample of **4** dissolved in DMSO-d_6.

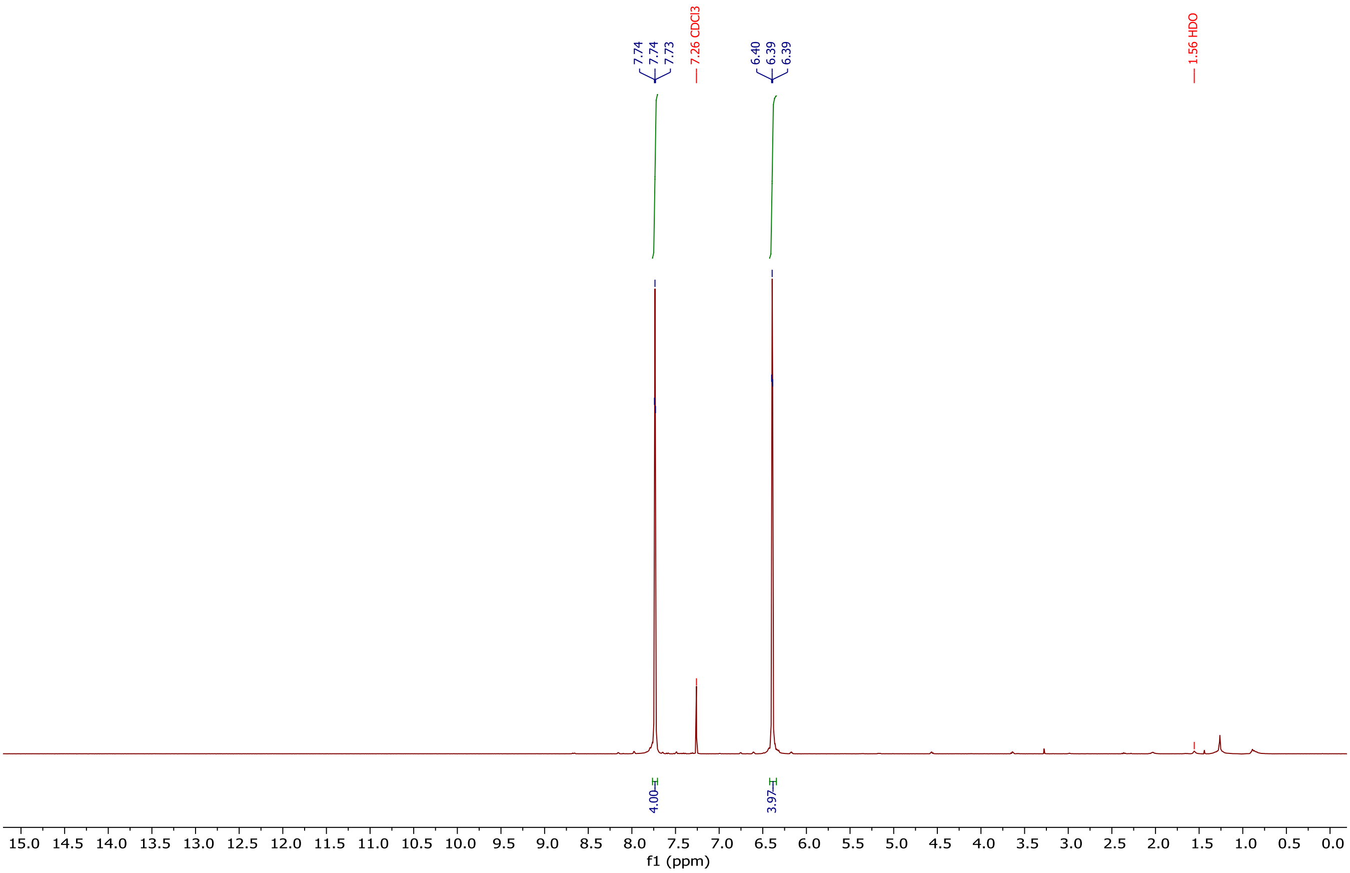

Figure S20. ^{1}H-NMR spectrum of a sample of **5** dissolved in $CDCl_3$.

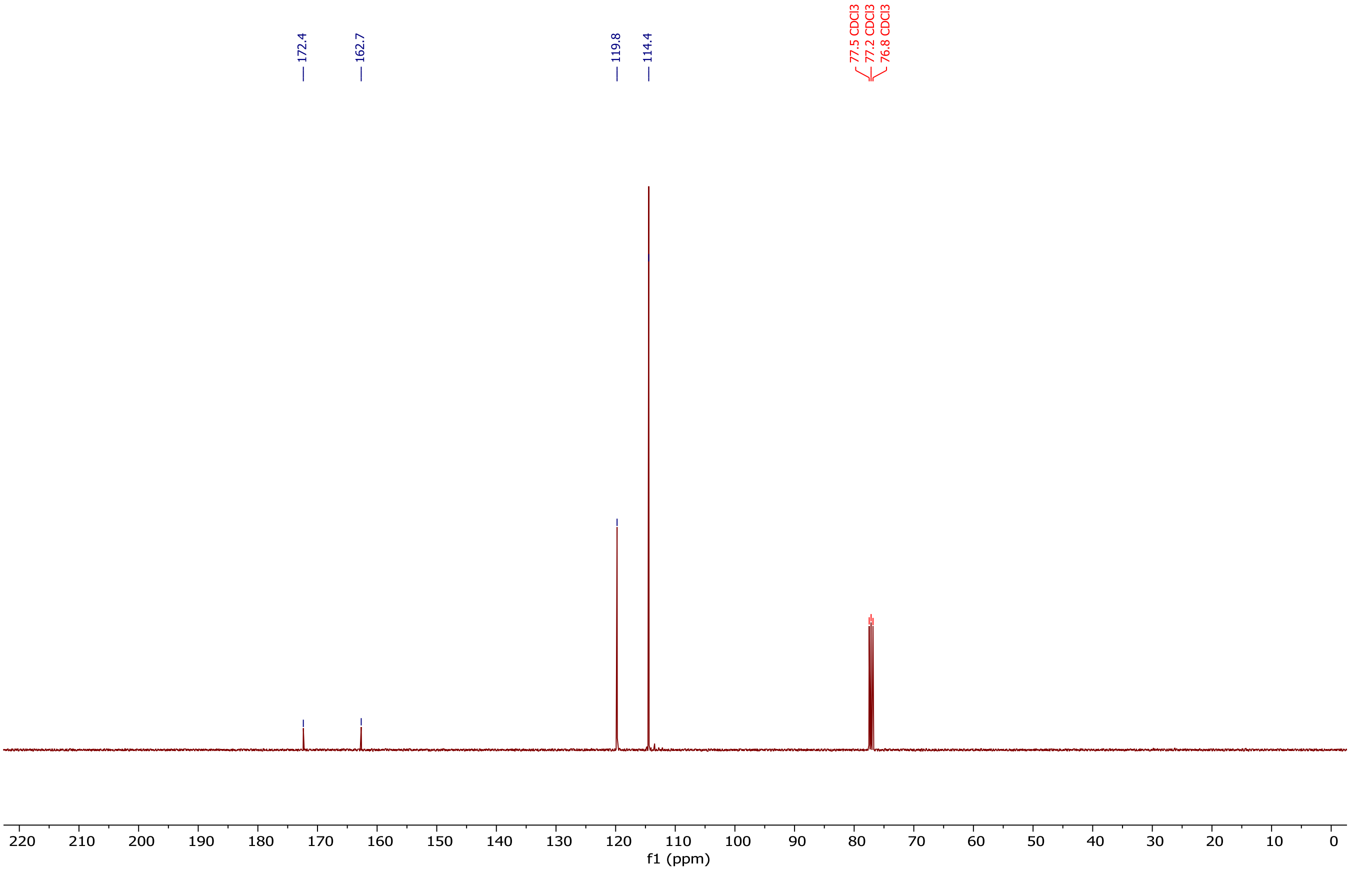

Figure S21. ^{13}C-NMR spectrum of a sample of **5** dissolved in $CDCl_3$.

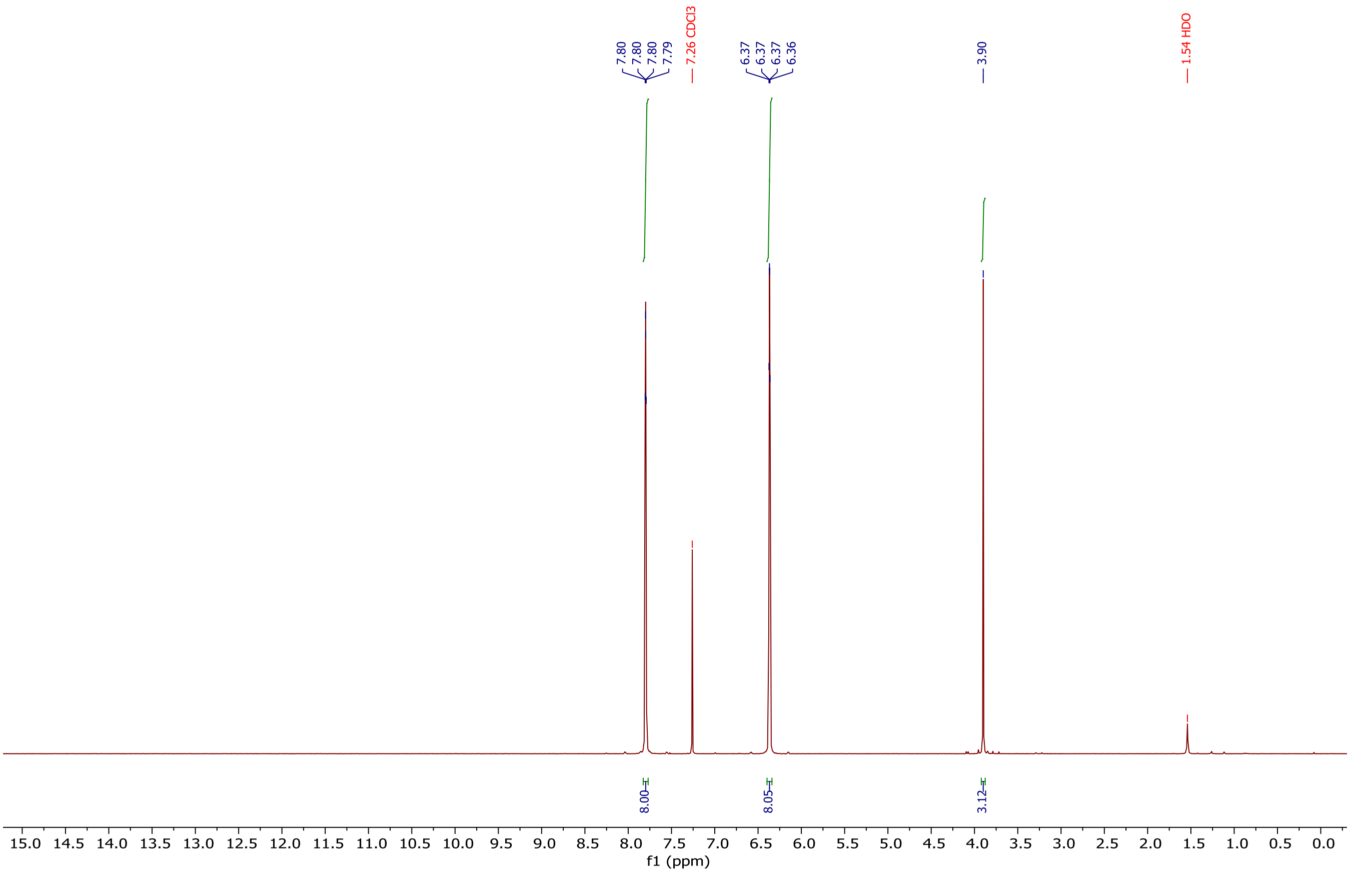

Figure S22. ^{1}H-NMR spectrum of a sample of **7** dissolved in $CDCl_3$.

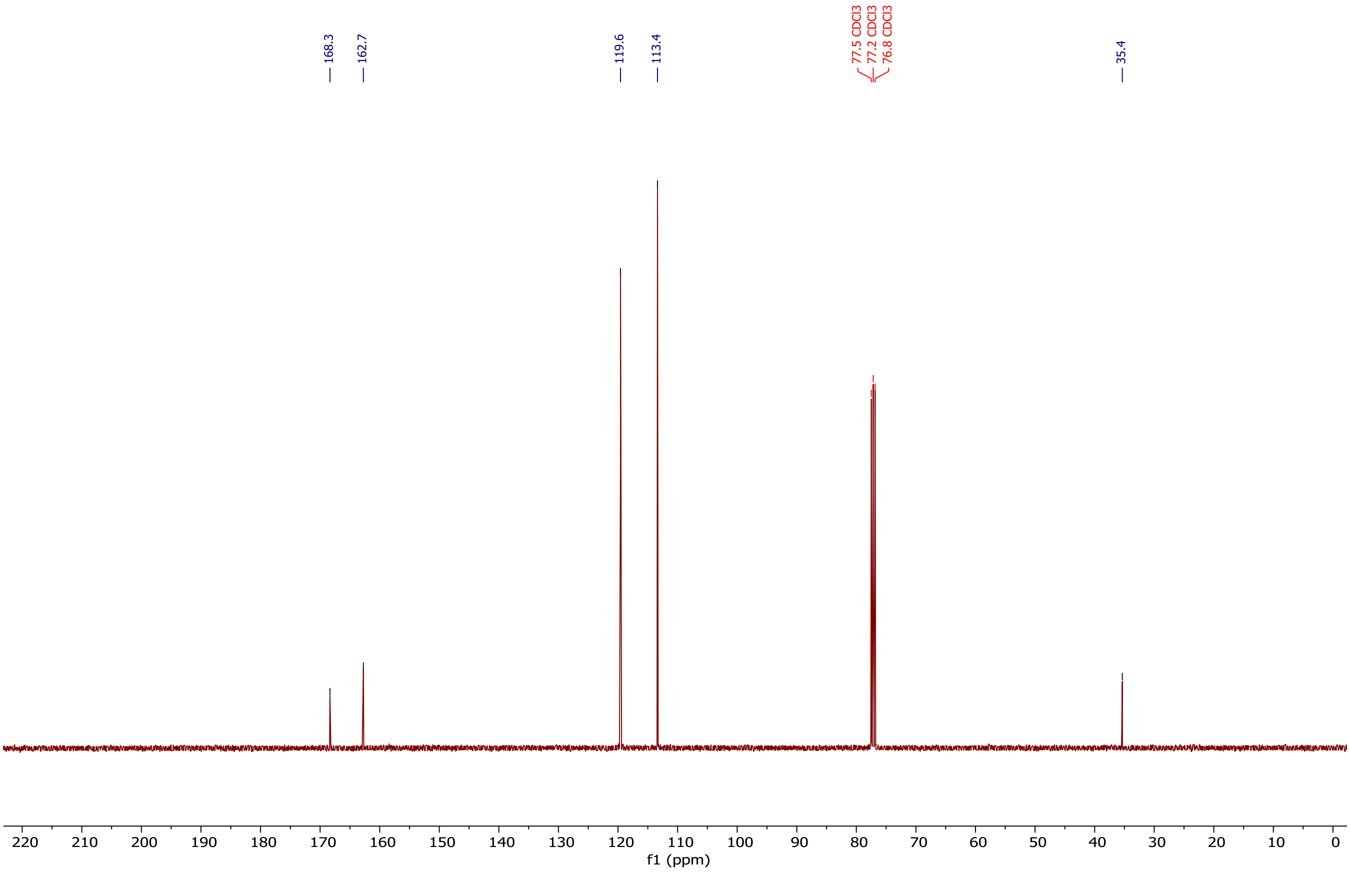

Figure S23. ^{13}C-NMR spectrum of a sample of **7** dissolved in $CDCl_3$.

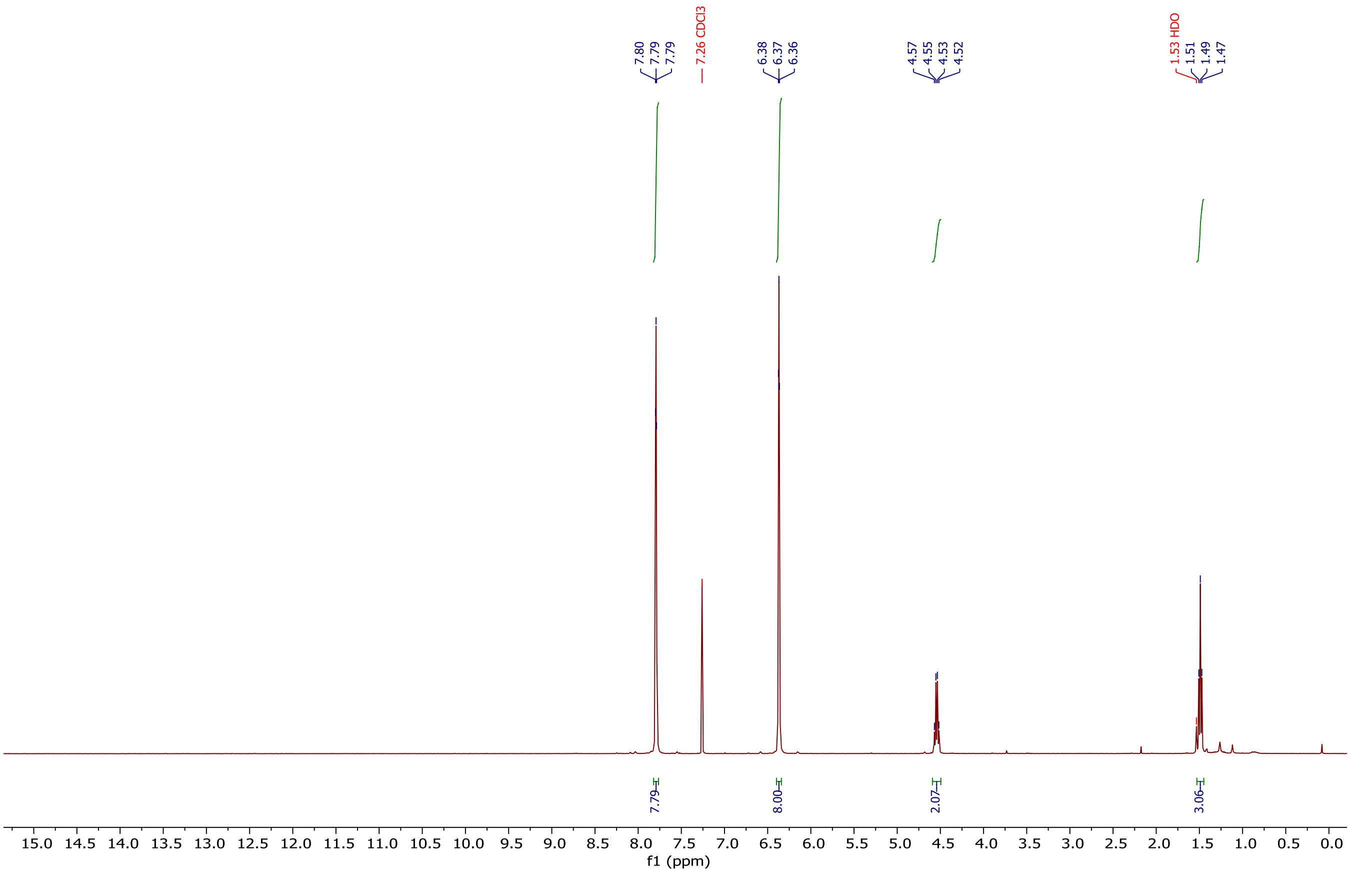

Figure S24. ^{1}H-NMR spectrum of a sample of **8** dissolved in $CDCl_3$.

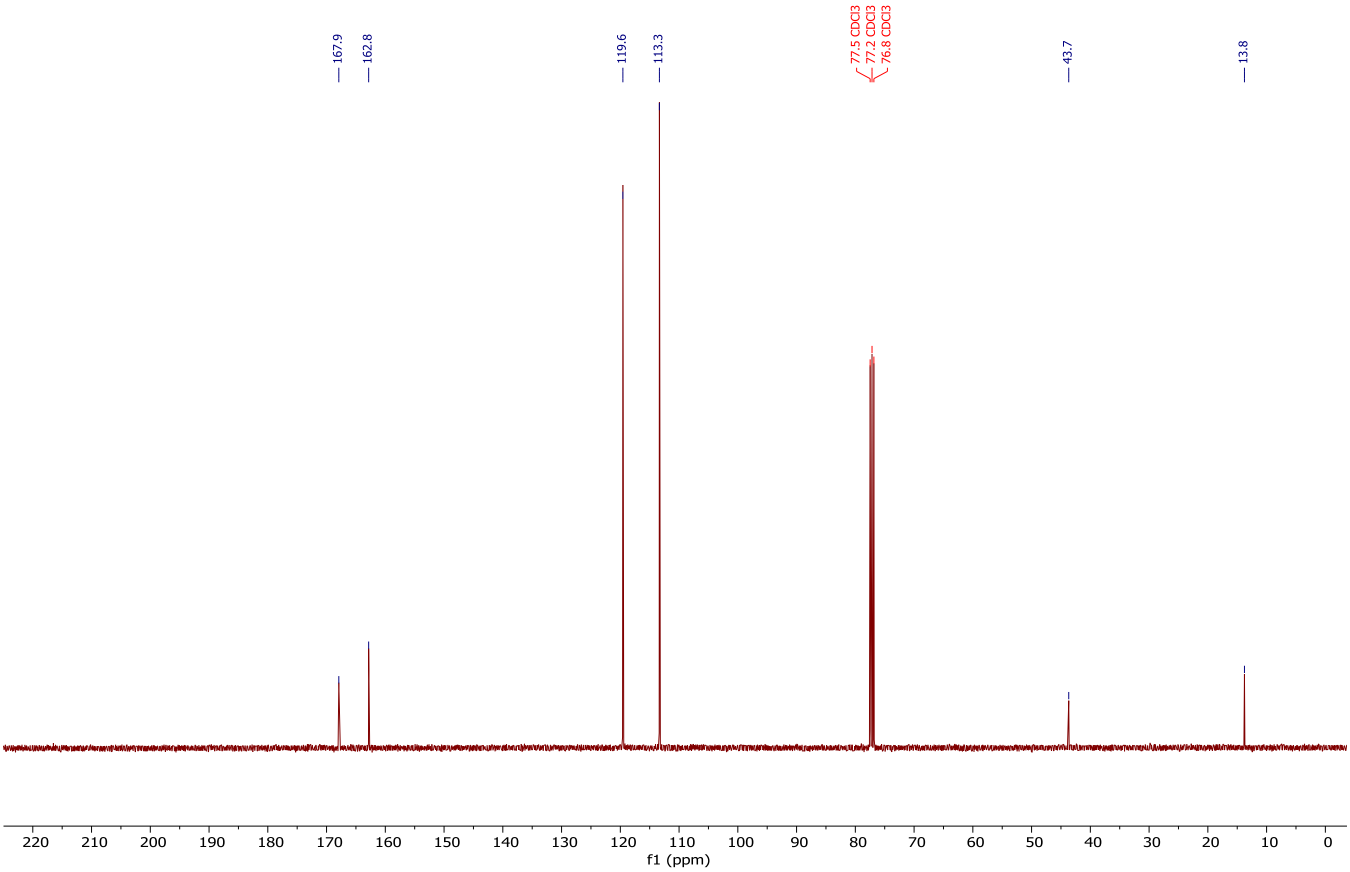

Figure S25. ^{13}C-NMR spectrum of a sample of **8** dissolved in $CDCl_3$.

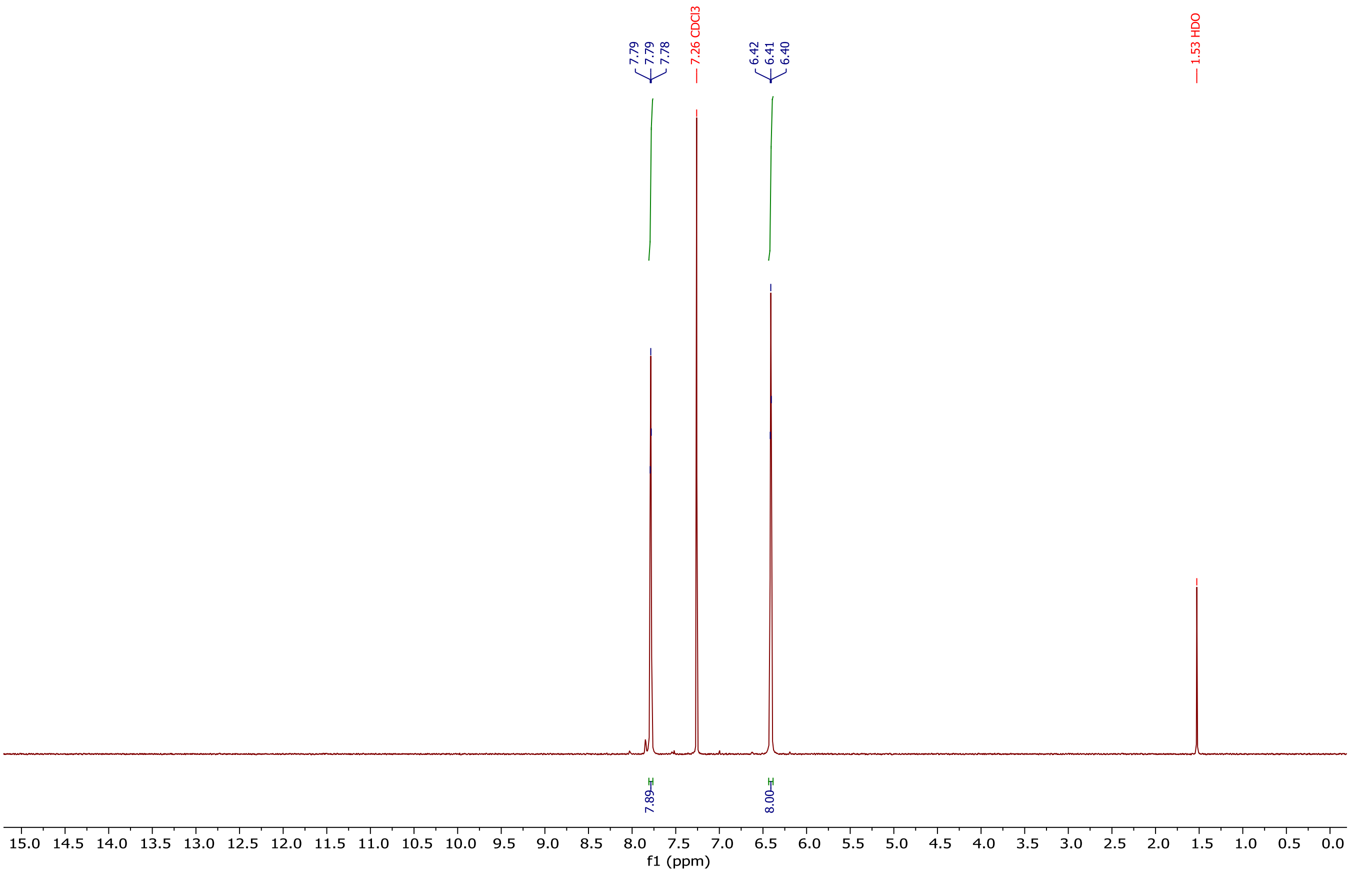

Figure S26. ^{1}H-NMR spectrum of a sample of **9** dissolved in $CDCl_3$.

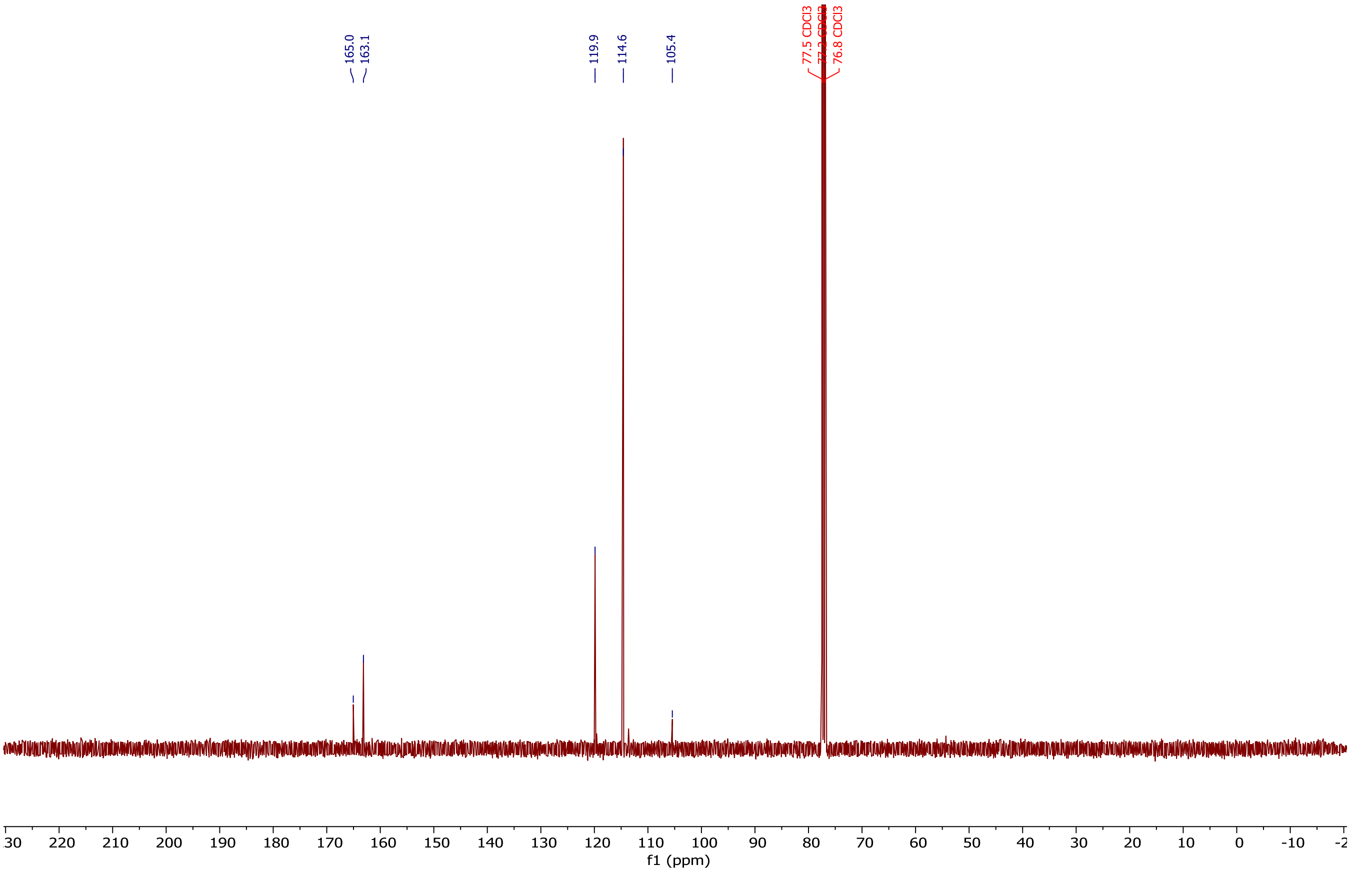

Figure S27. ^{13}C-NMR spectrum of a sample of **9** dissolved in $CDCl_3$.

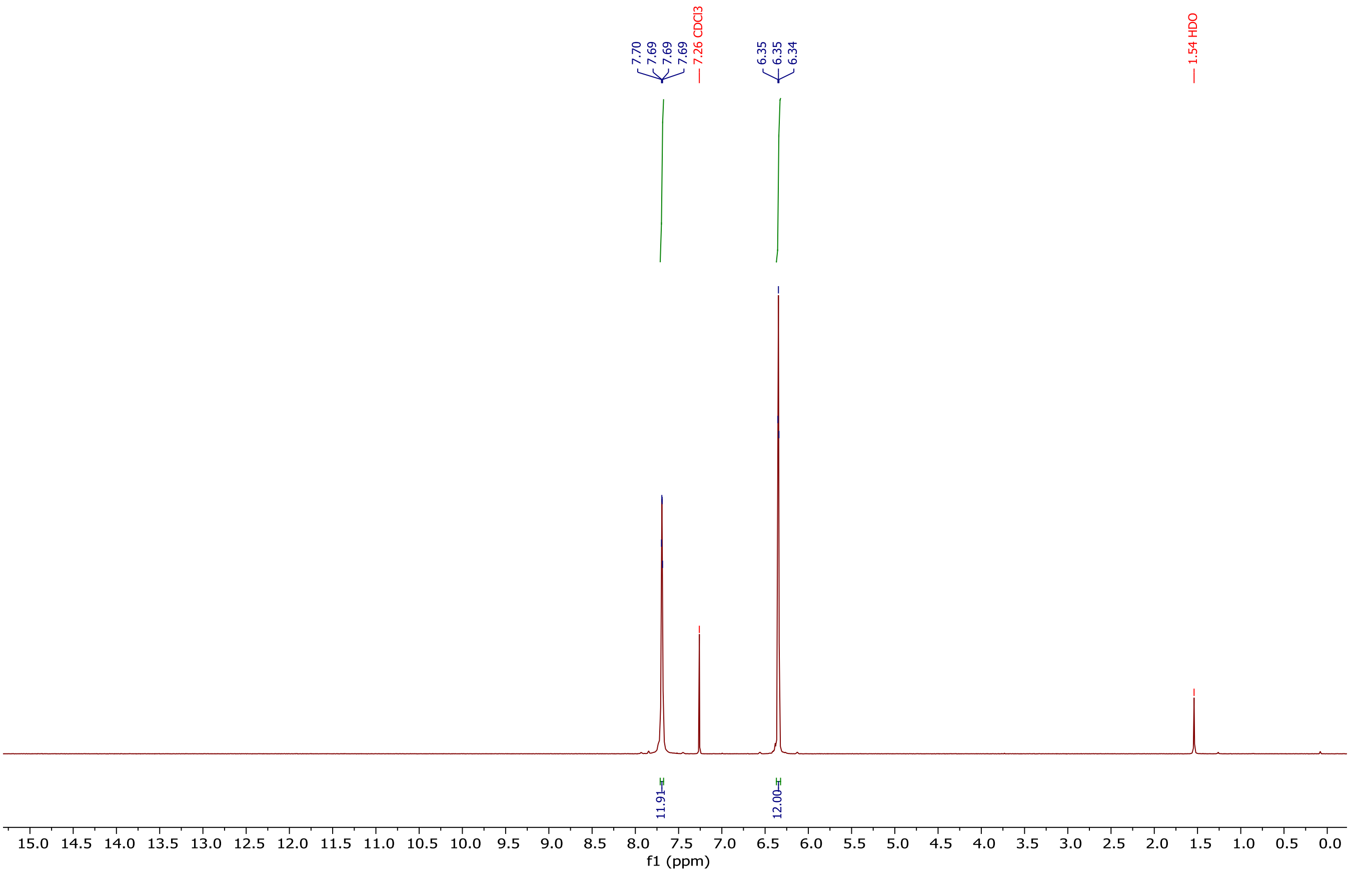

Figure S28. ^{1}H-NMR spectrum of a sample of **10** dissolved in $CDCl_3$.

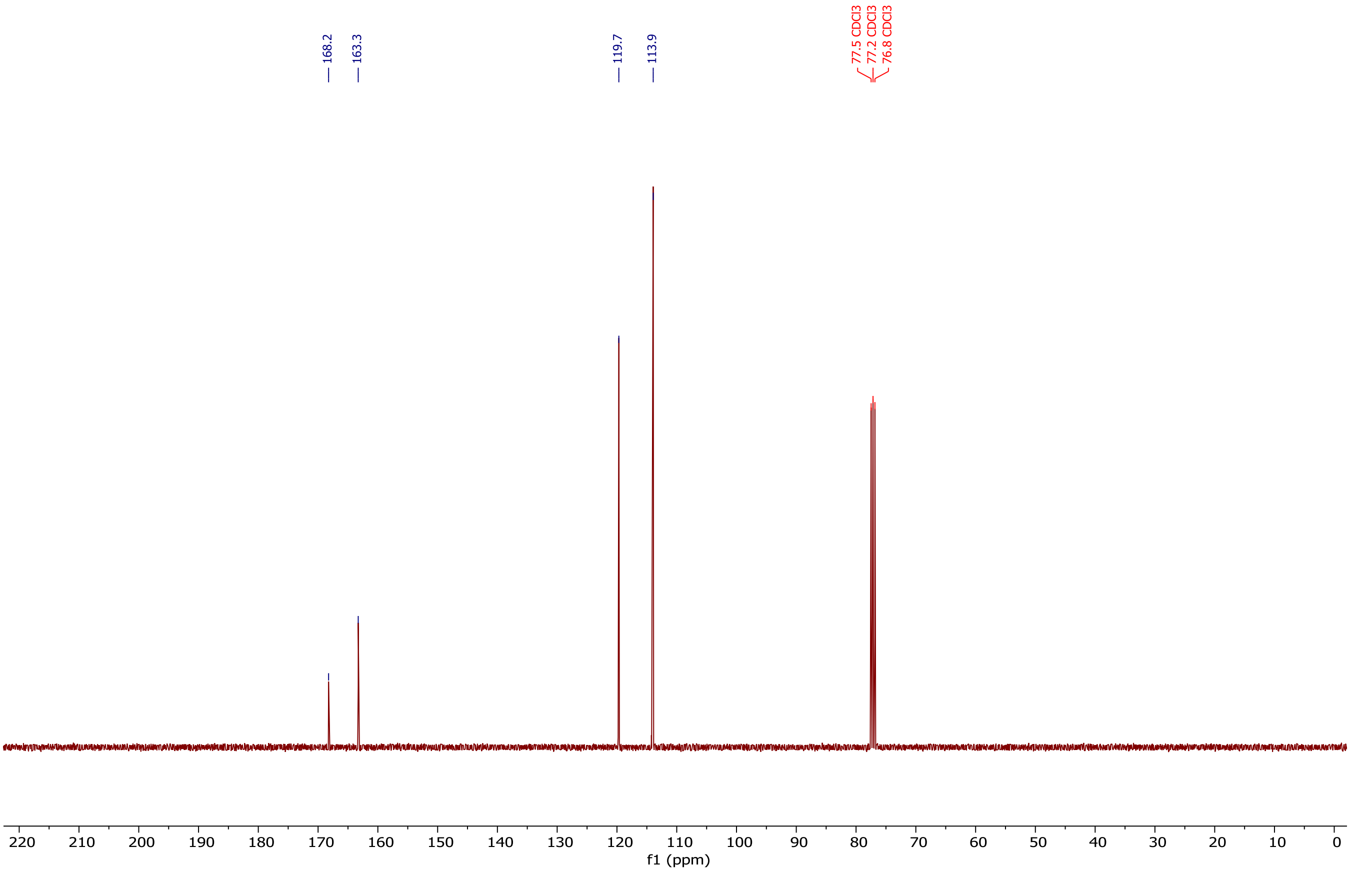

Figure S29. ^{13}C-NMR spectrum of a sample of **10** dissolved in $CDCl_3$.

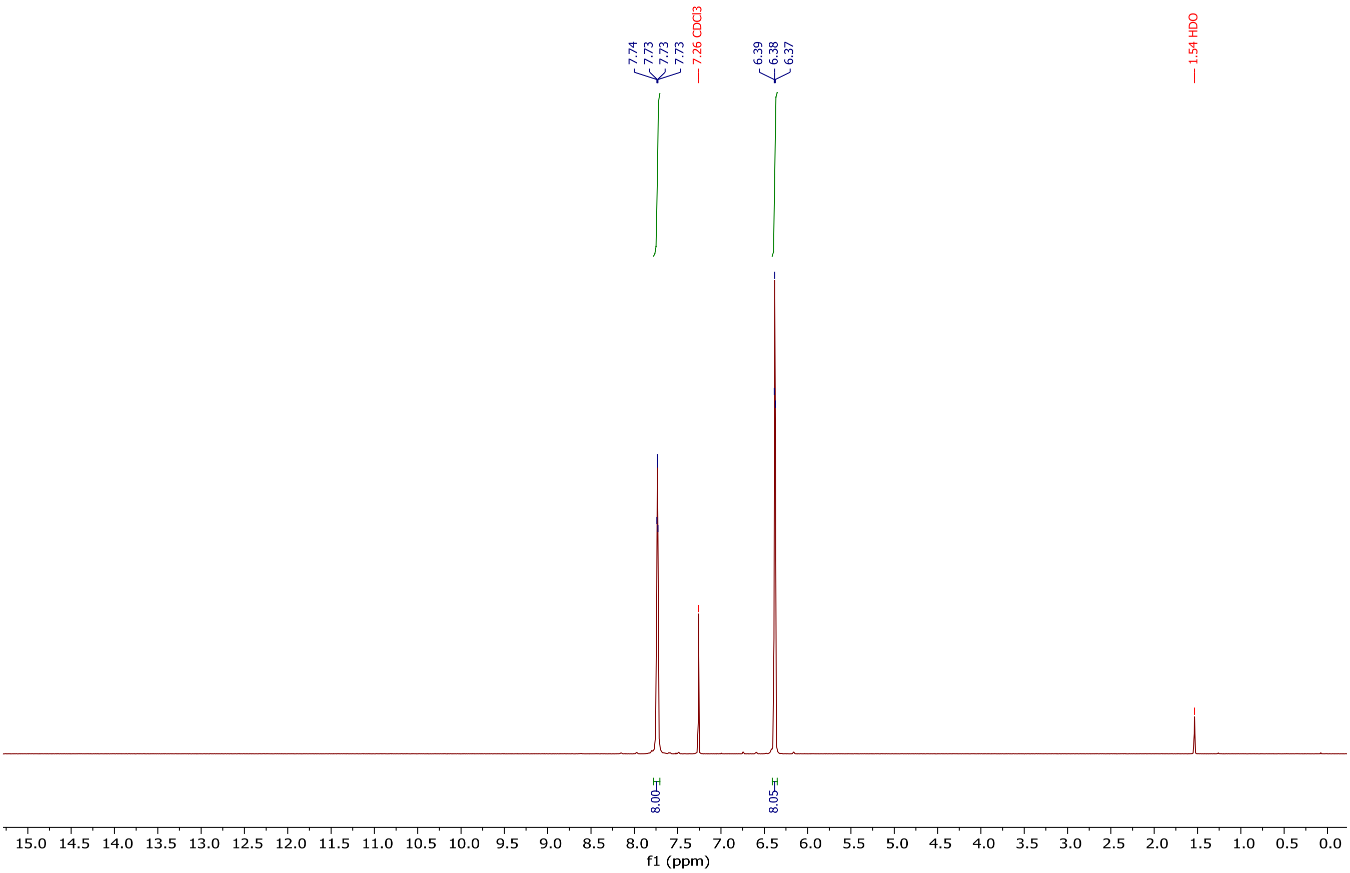

Figure S30. ^{1}H-NMR spectrum of a sample of **11** dissolved in $CDCl_3$.

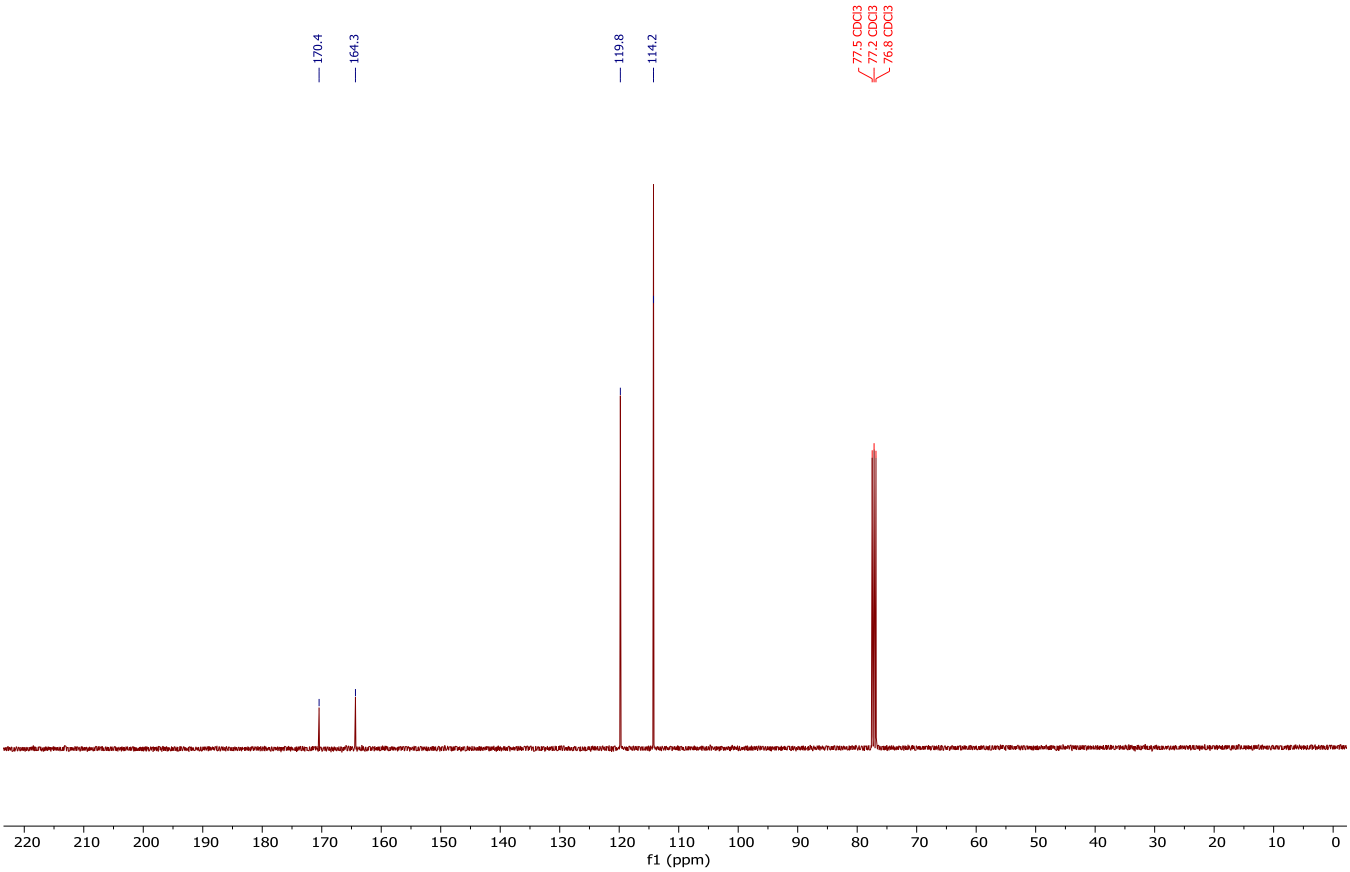

Figure S31. ^{13}C-NMR spectrum of a sample of **11** dissolved in $CDCl_3$.

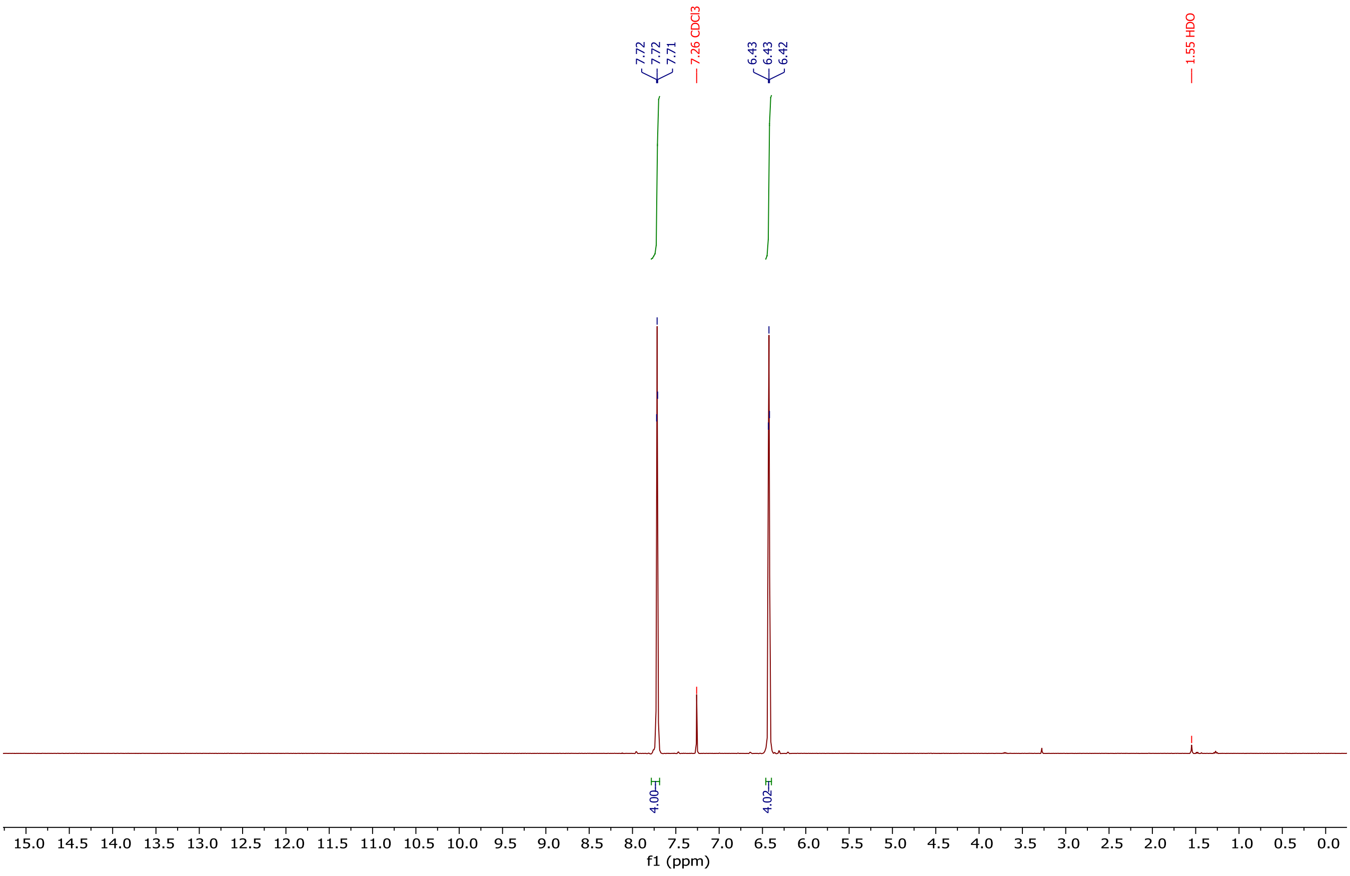

Figure S32. ^{1}H-NMR spectrum of a sample of **12** dissolved in $CDCl_3$.

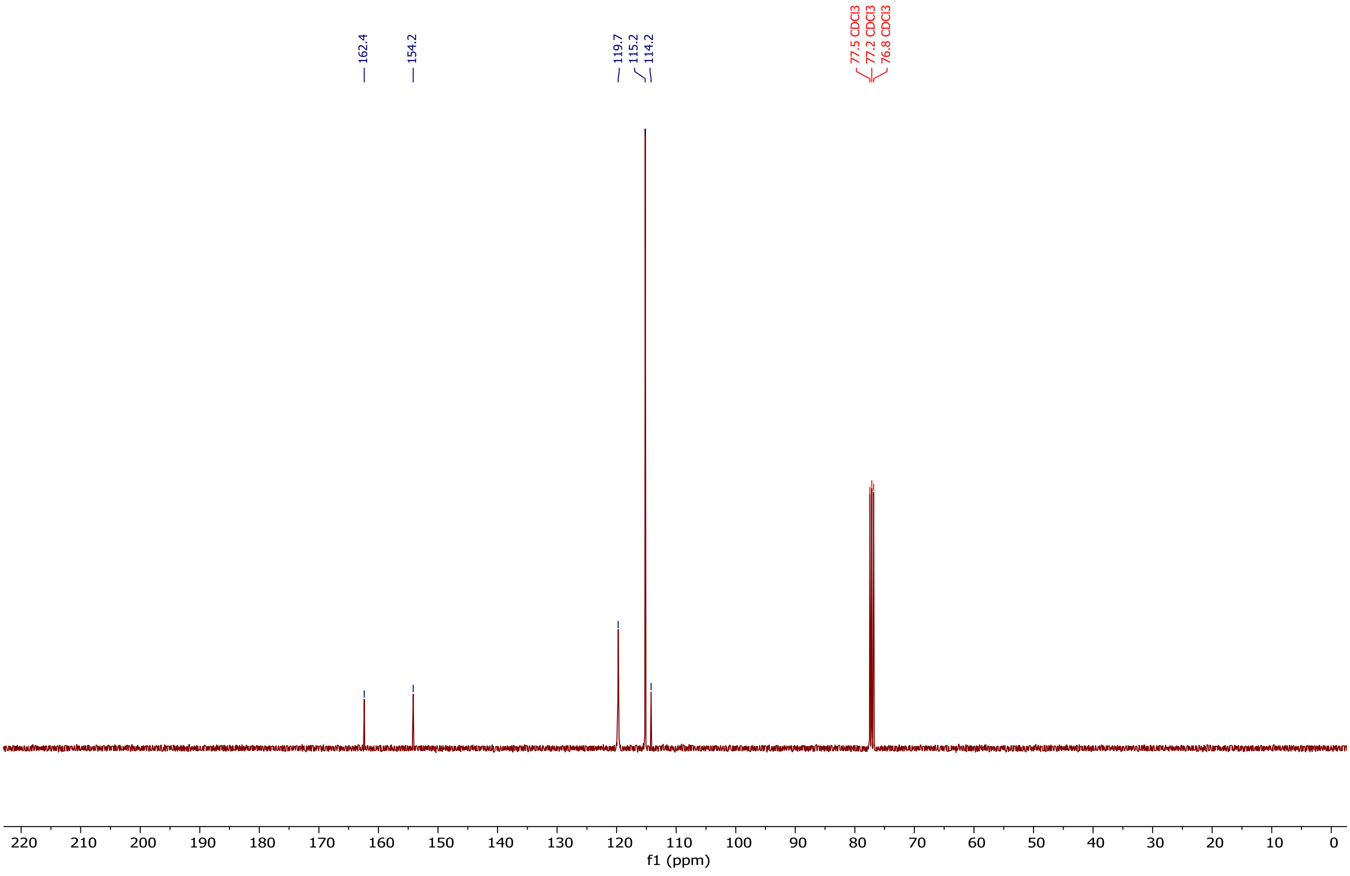

Figure S33. ^{13}C-NMR spectrum of a sample of **12** dissolved in $CDCl_3$.

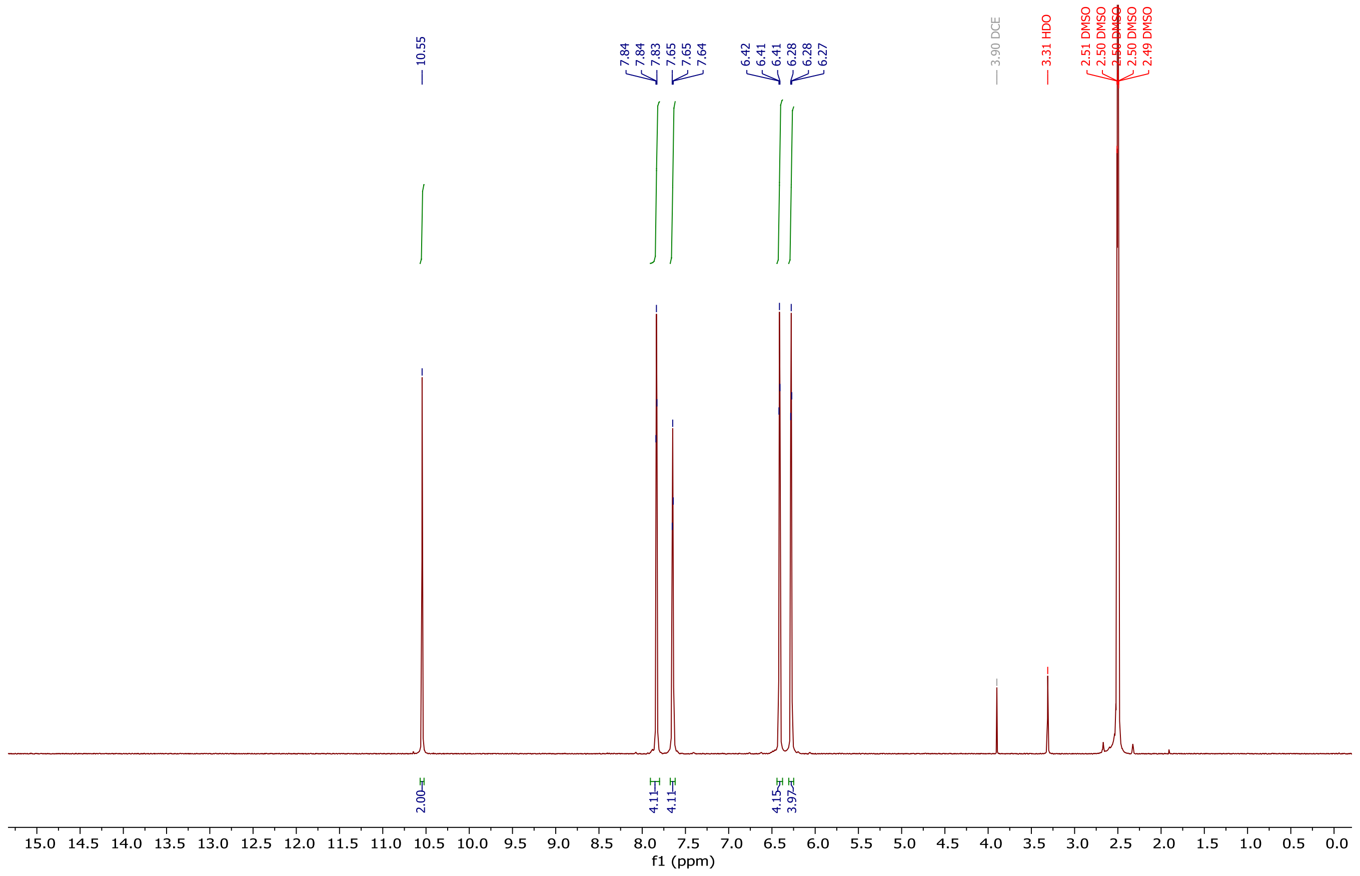

Figure S34. ^{1}H-NMR spectrum of a sample of **13i** dissolved in DMSO-d_6.

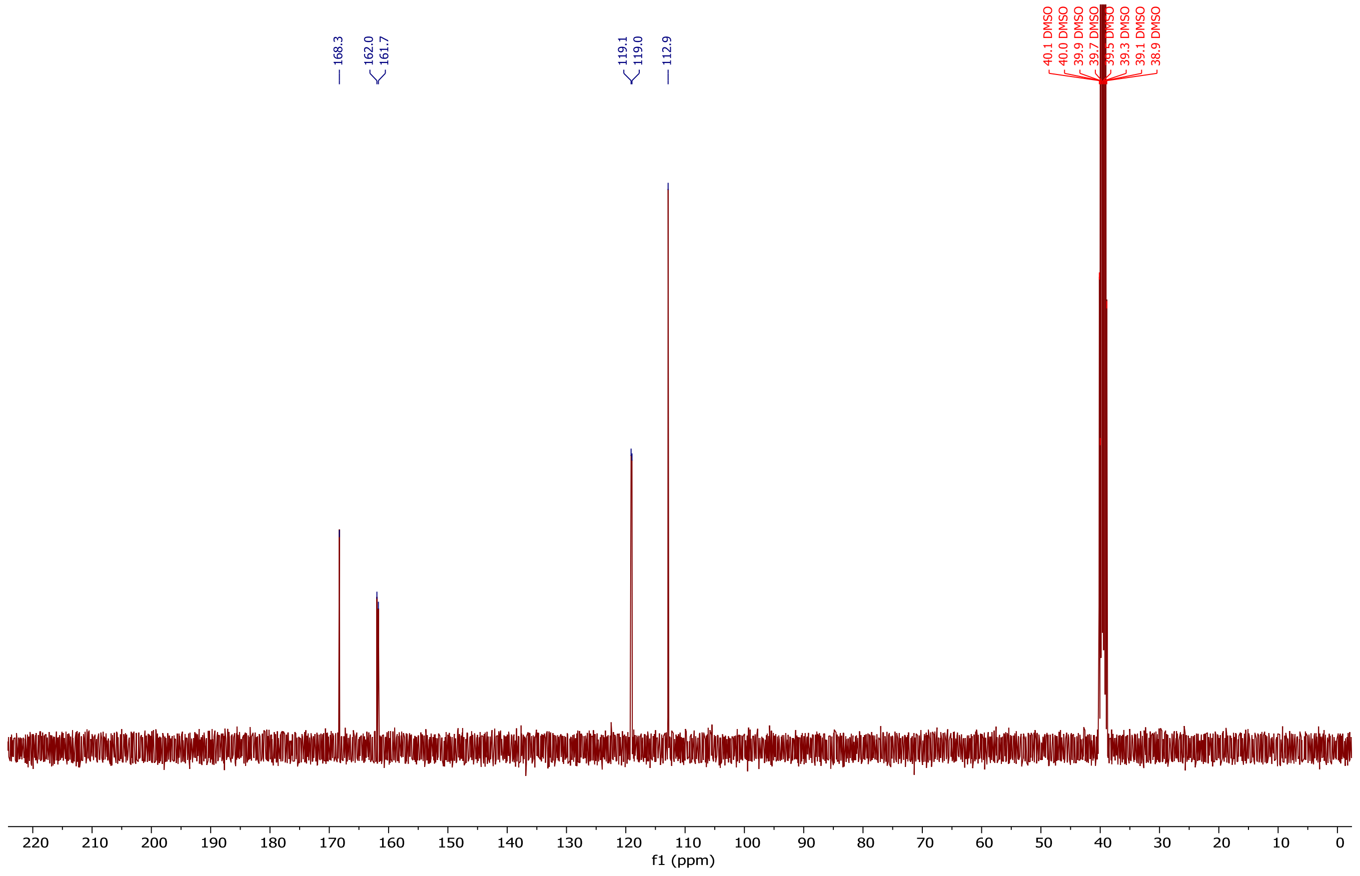

Figure S35. ^{13}C-NMR spectrum of a sample of **13i** dissolved in DMSO-d_6.

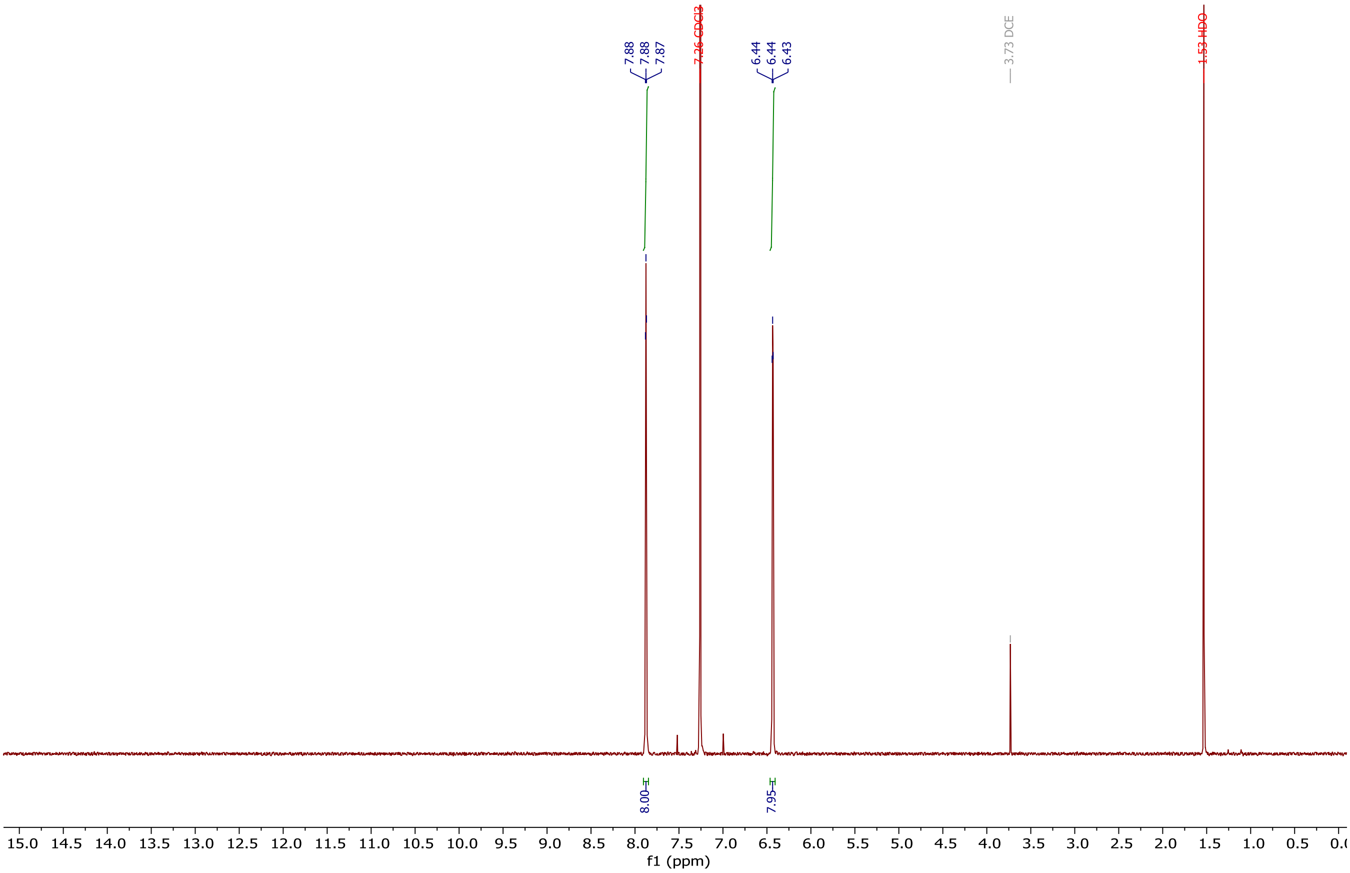

Figure S36. ^{1}H-NMR spectrum of a sample of **13** dissolved in $CDCl_3$.

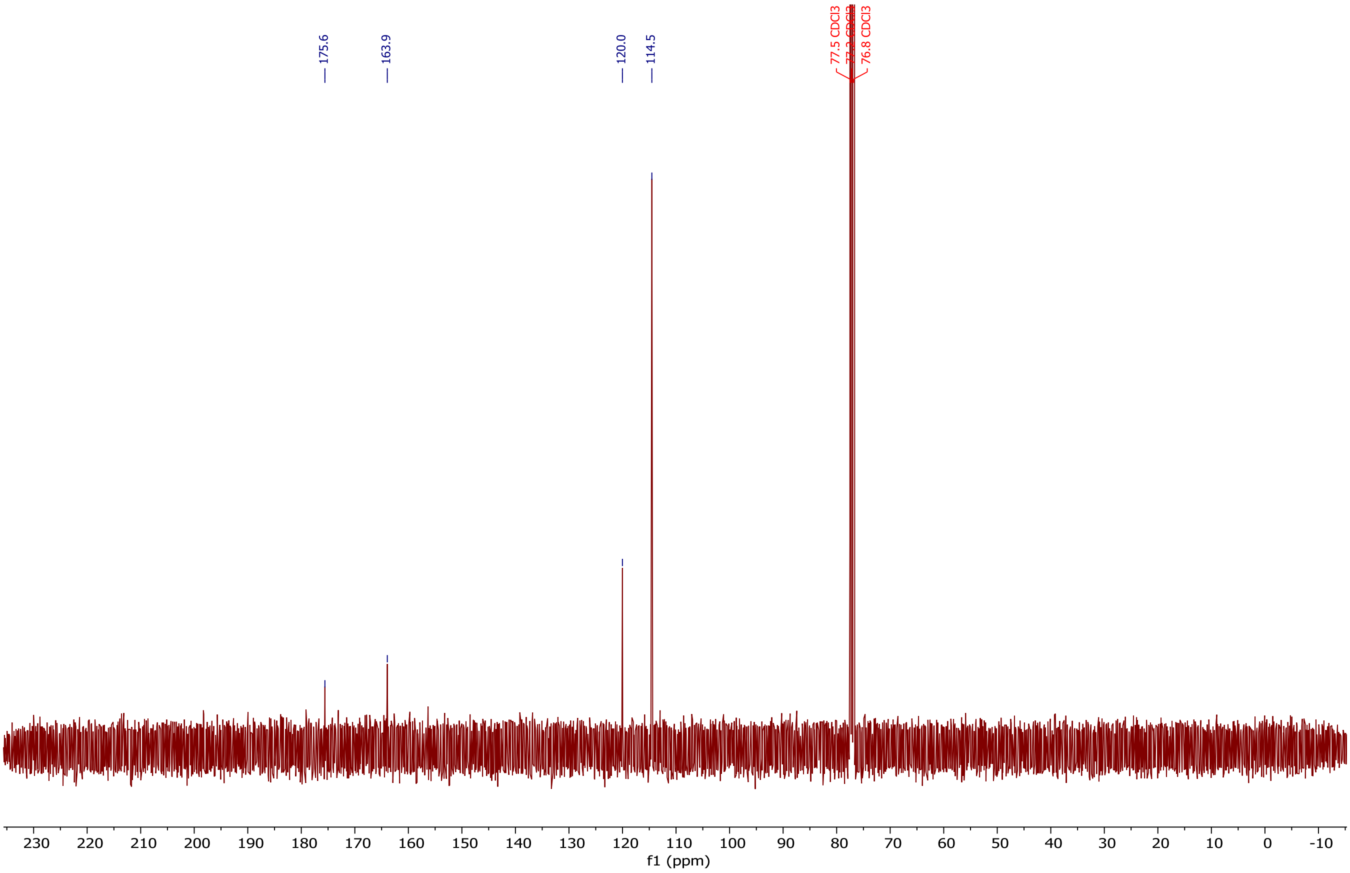

Figure S37. ^{13}C-NMR spectrum of a sample of **13** dissolved in $CDCl_3$.

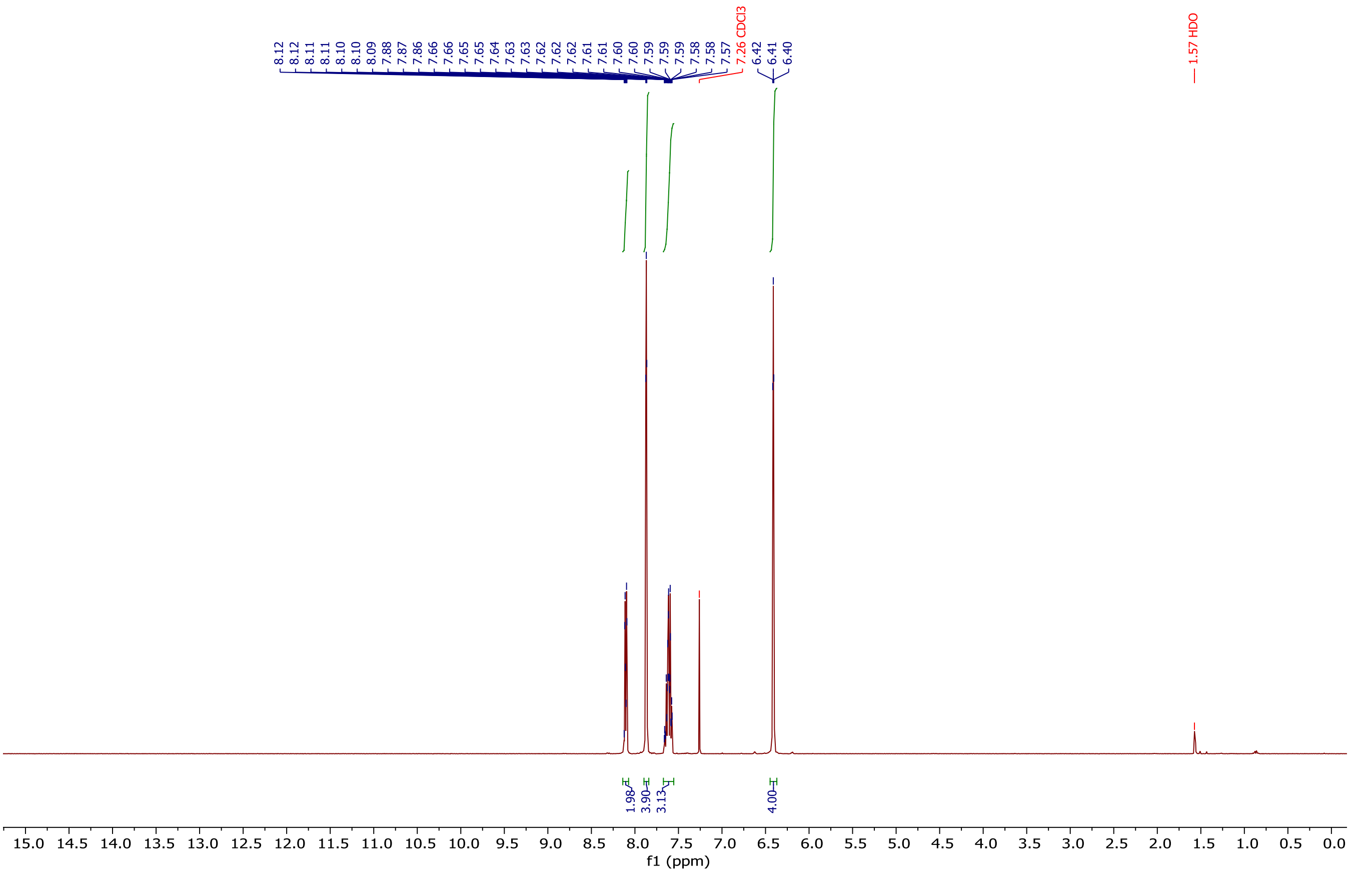

Figure S38. ^{1}H-NMR spectrum of a sample of **14** dissolved in $CDCl_3$.

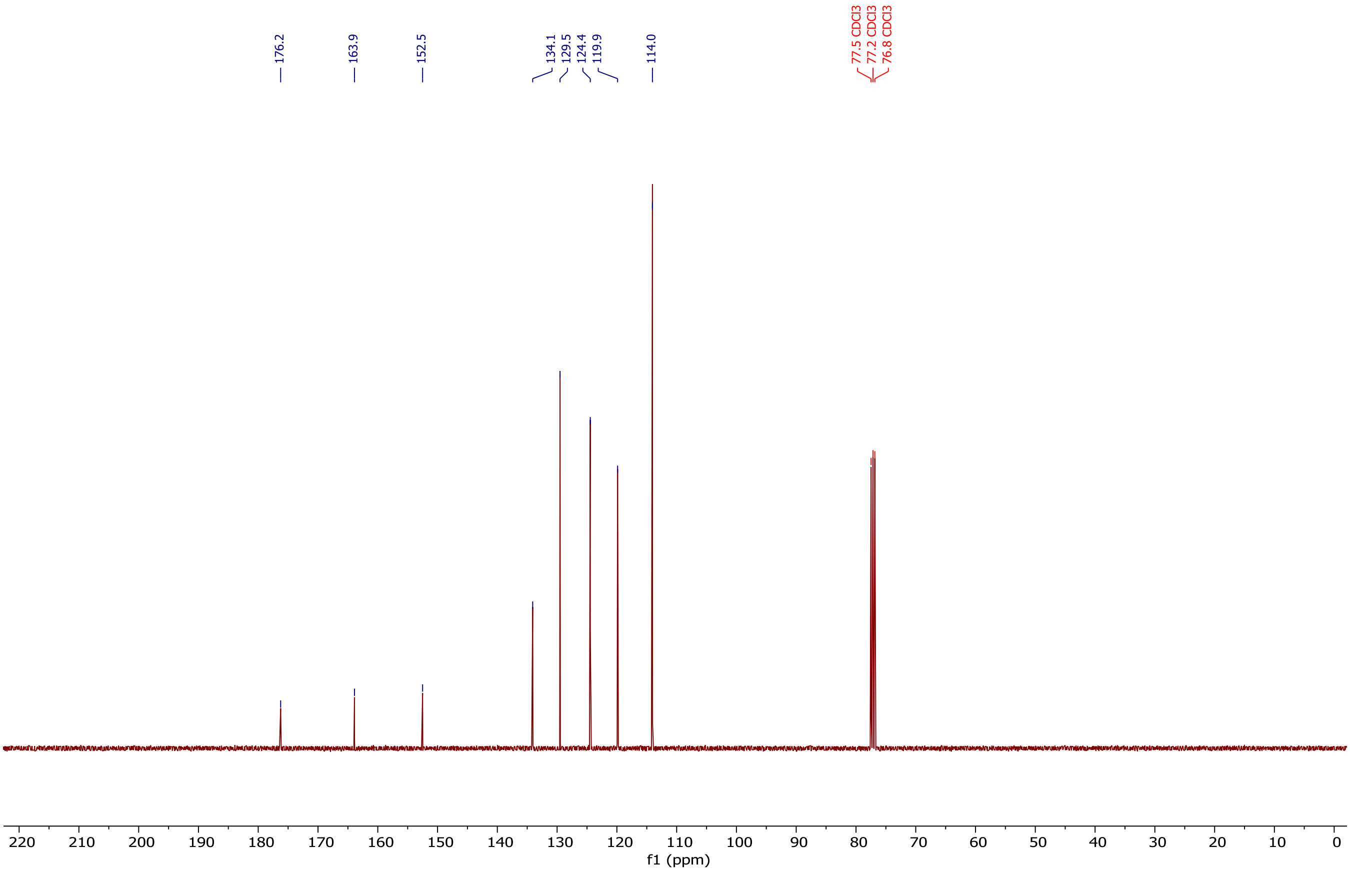

Figure S39. ^{13}C-NMR spectrum of a sample of **14** dissolved in $CDCl_3$.

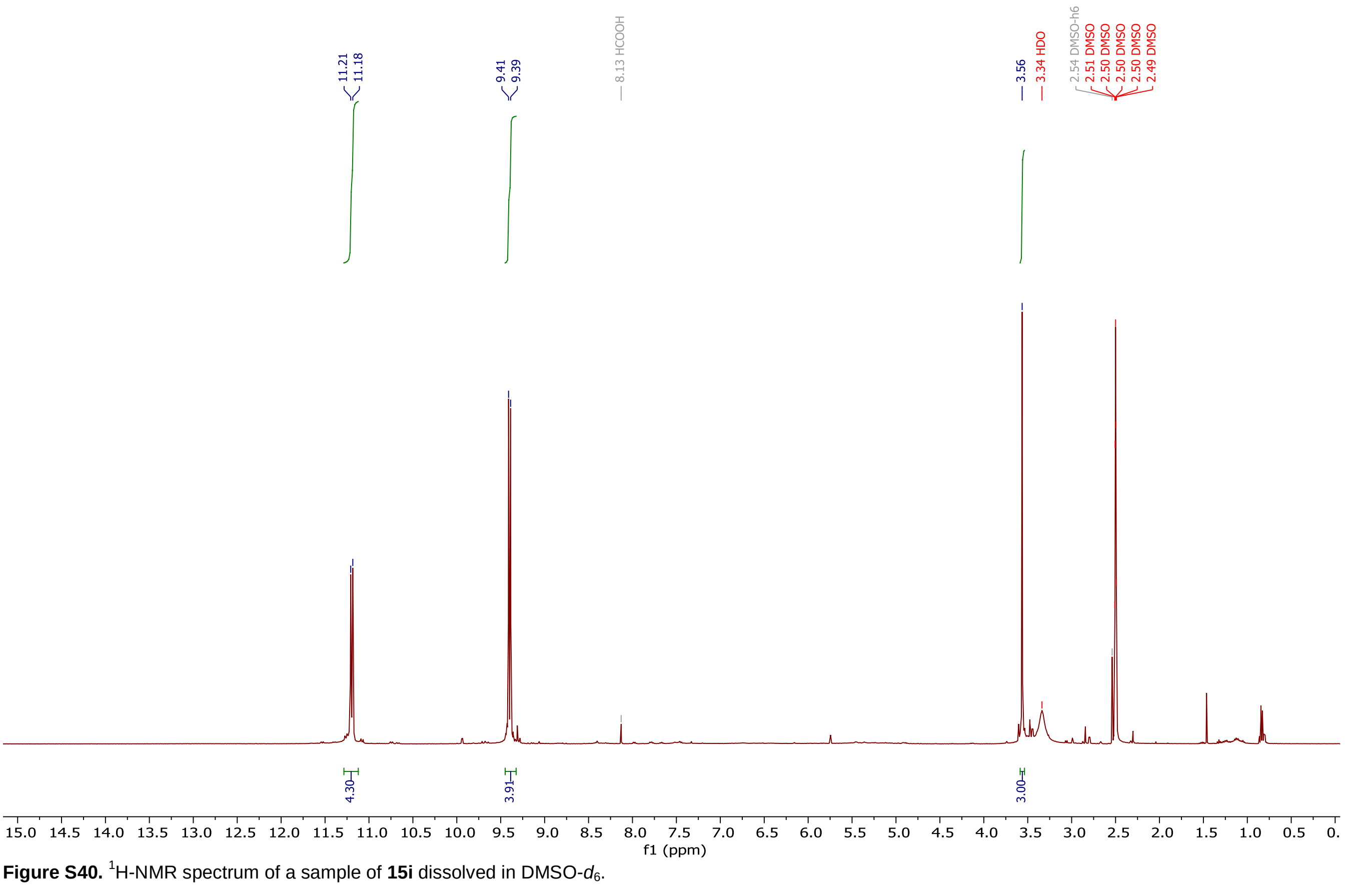

Figure S40. ^{1}H-NMR spectrum of a sample of **15i** dissolved in DMSO-d_6.

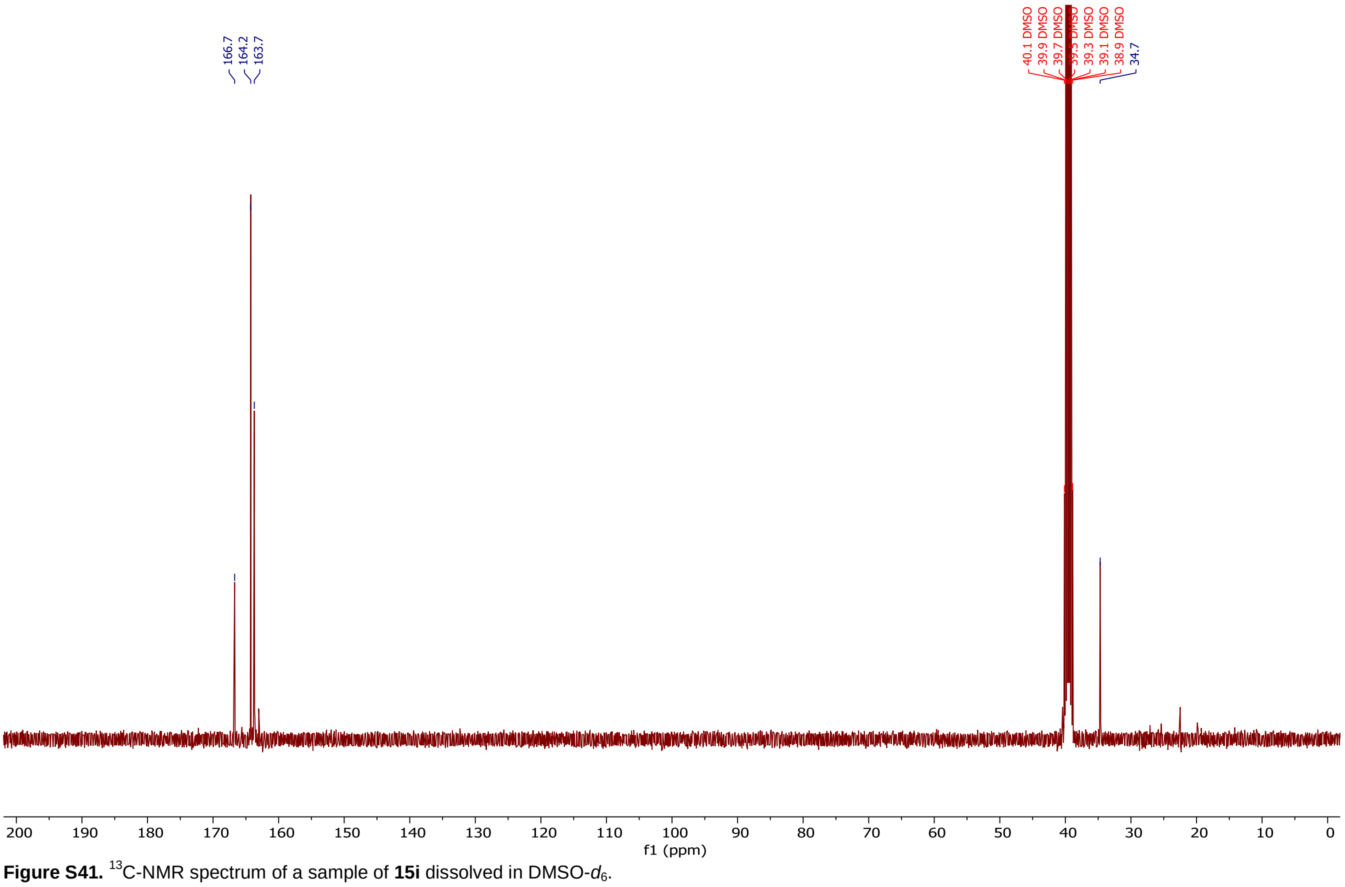

Figure S41. ^{13}C-NMR spectrum of a sample of **15i** dissolved in DMSO-d_6.

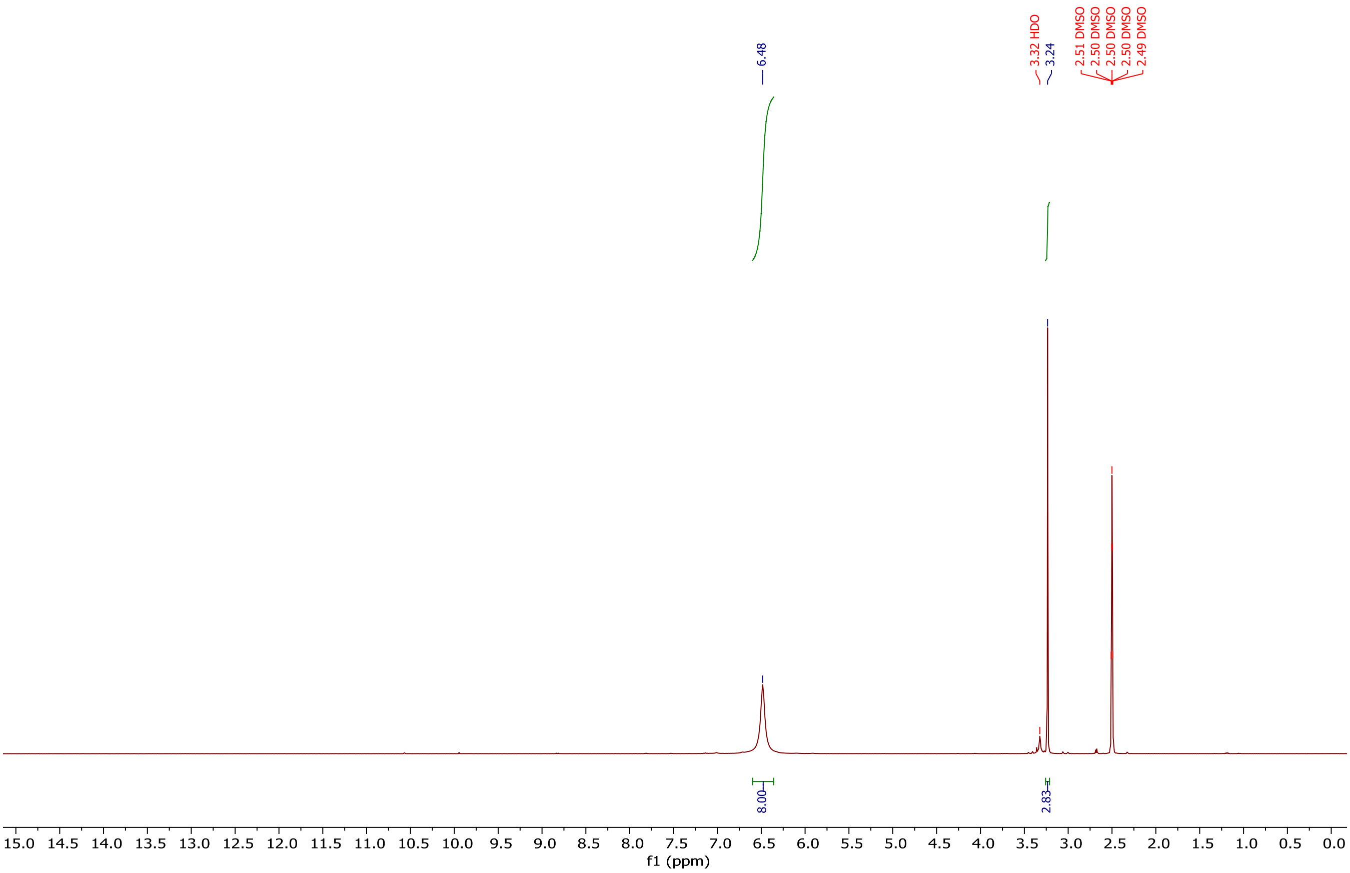

Figure S42. ^{1}H-NMR spectrum of a sample of **15** dissolved in DMSO-d_6.

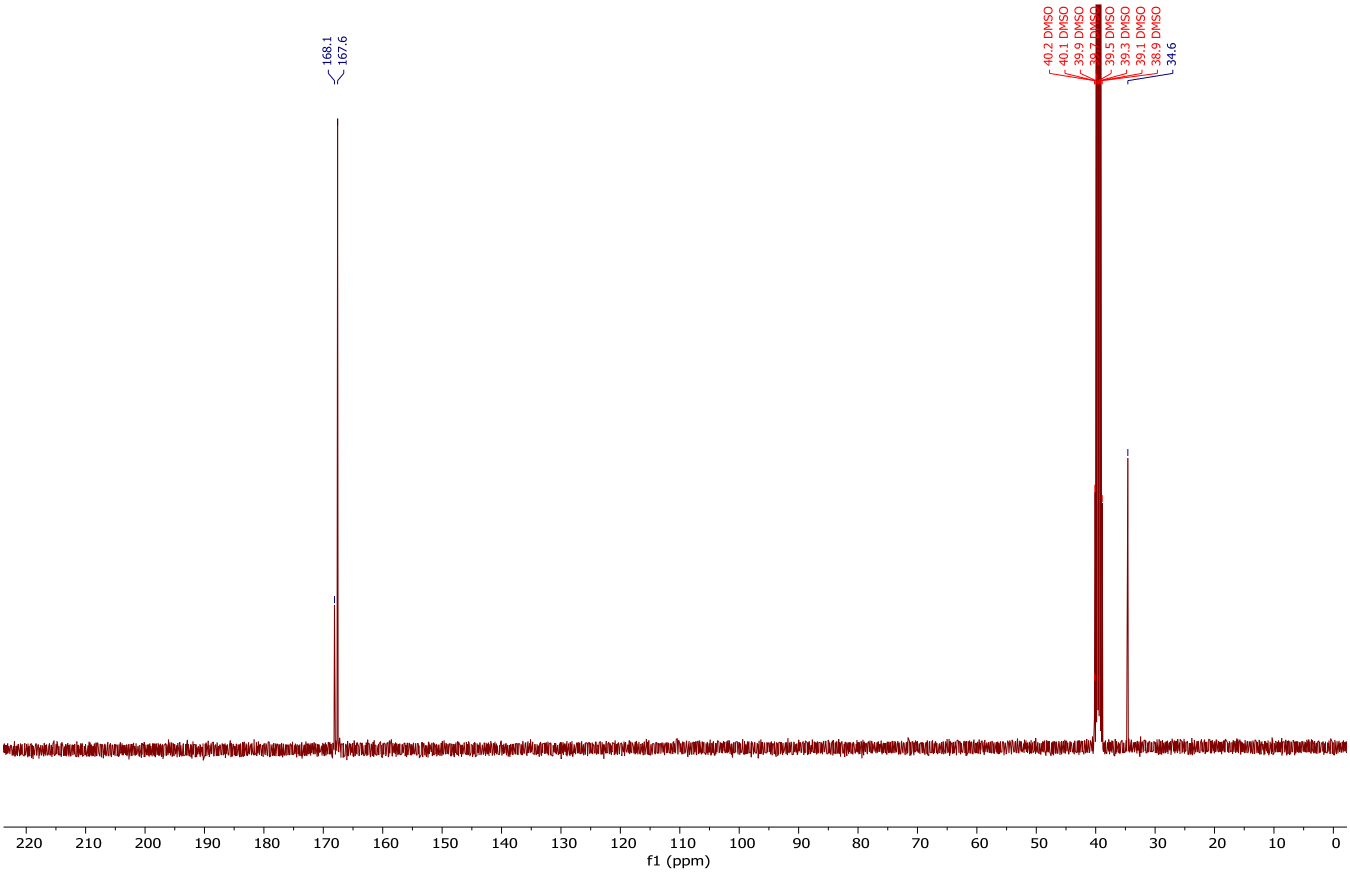

Figure S43. ^{13}C-NMR spectrum of a sample of **15** dissolved in DMSO-d_6.

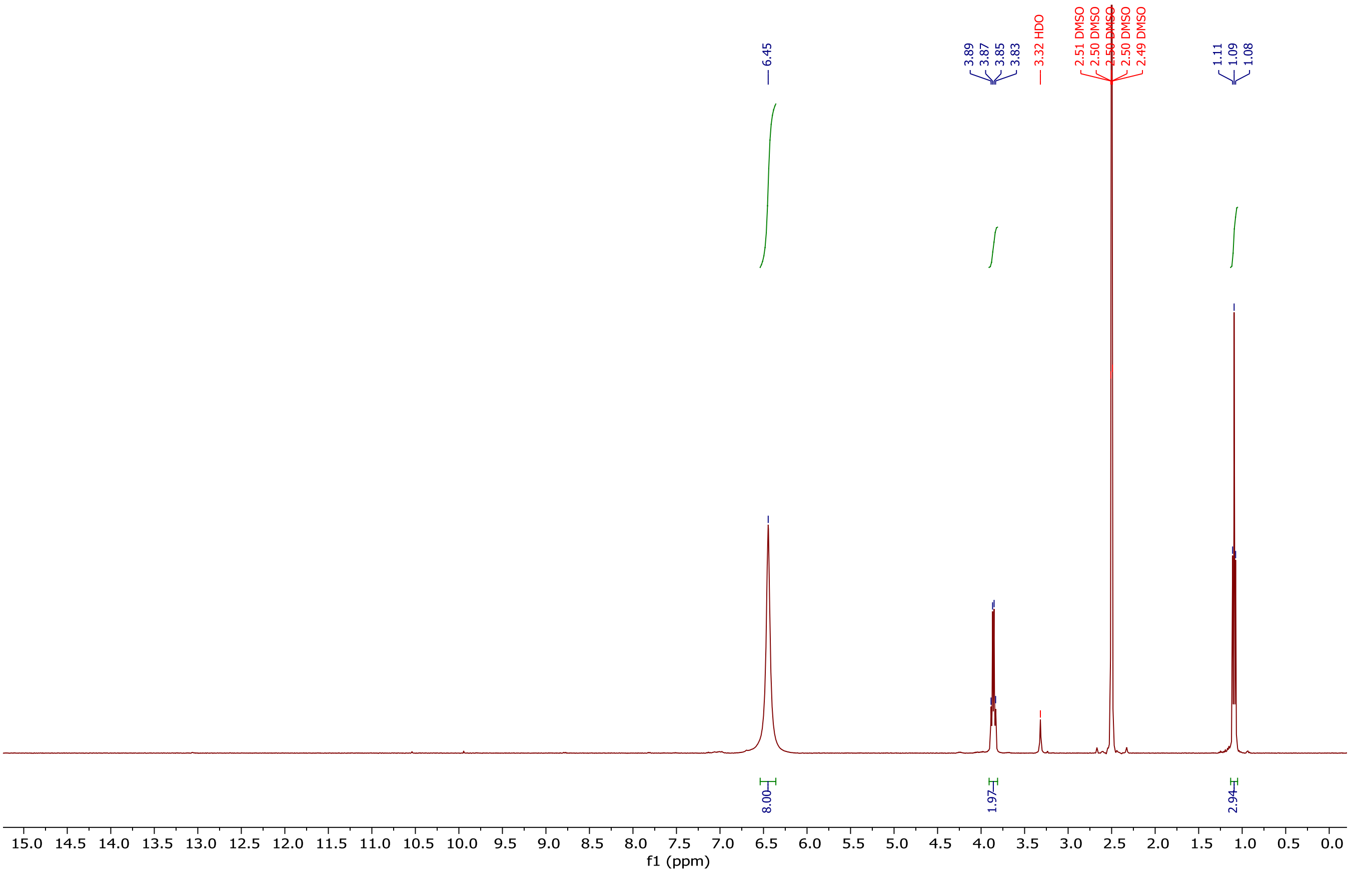

Figure S44. ^{1}H-NMR spectrum of a sample of **16** dissolved in DMSO-d_6.

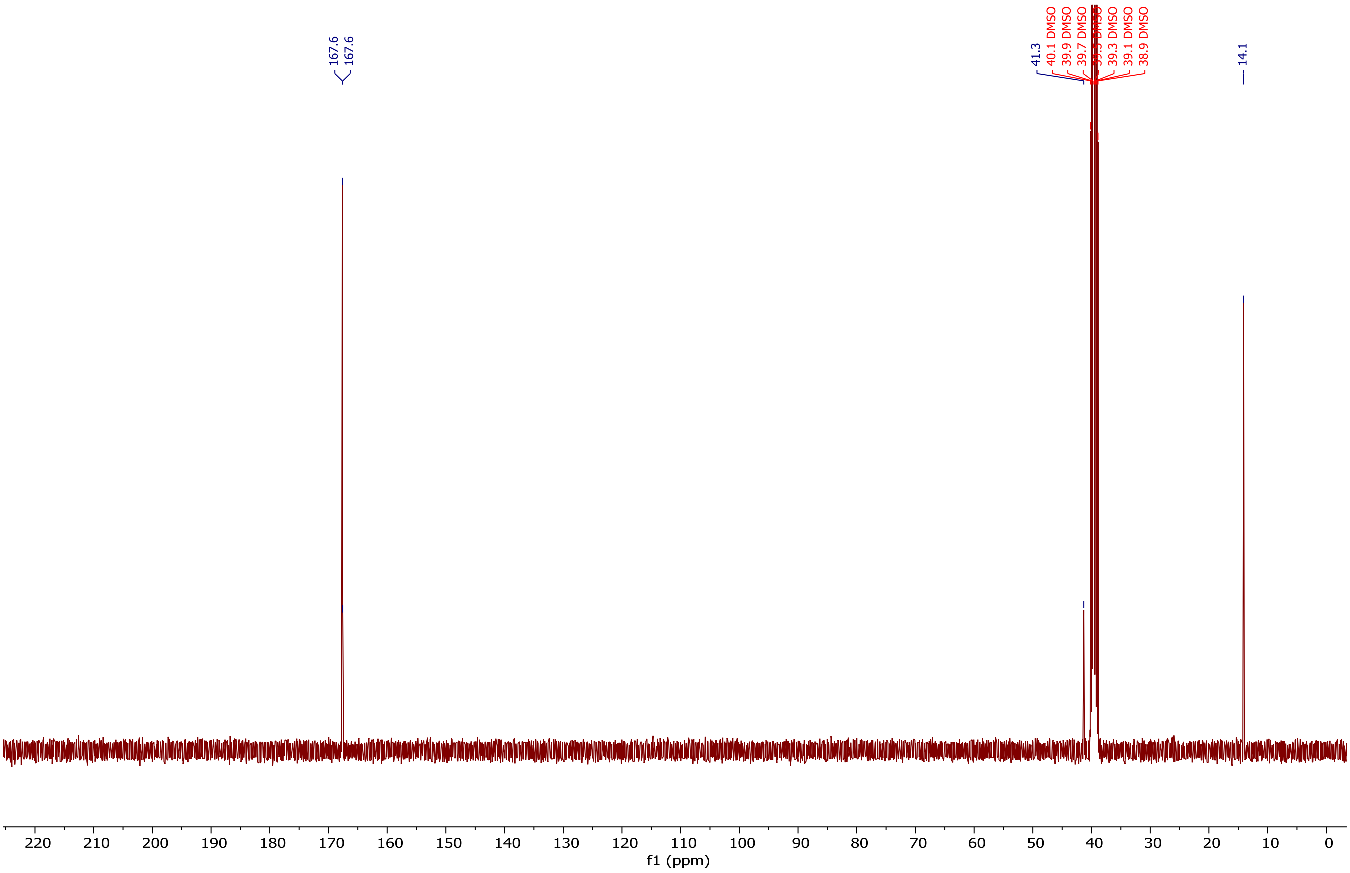

Figure S45. ^{13}C-NMR spectrum of a sample of **16** dissolved in DMSO-d_6.

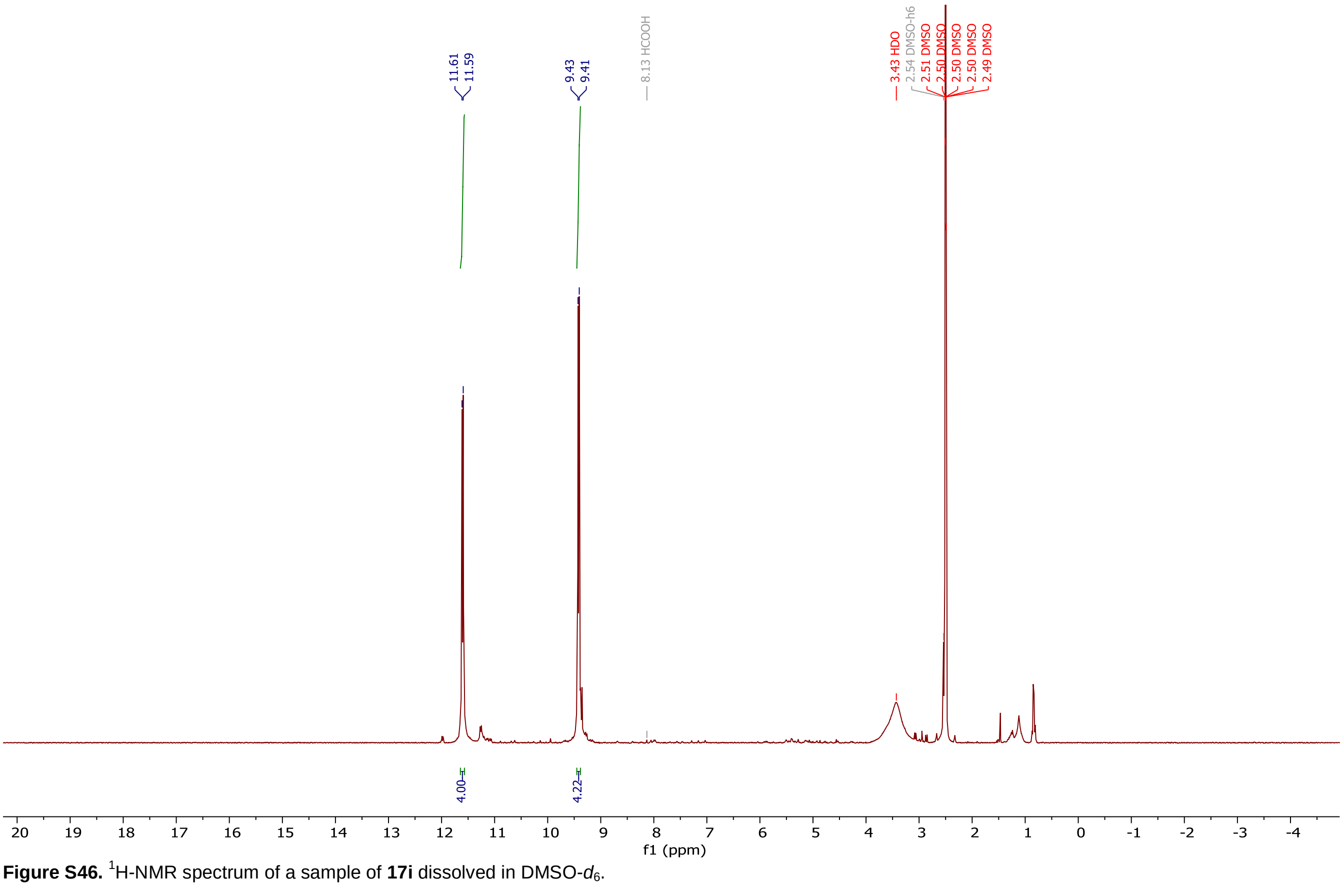

Figure S46. ^{1}H-NMR spectrum of a sample of **17i** dissolved in DMSO-d_6.

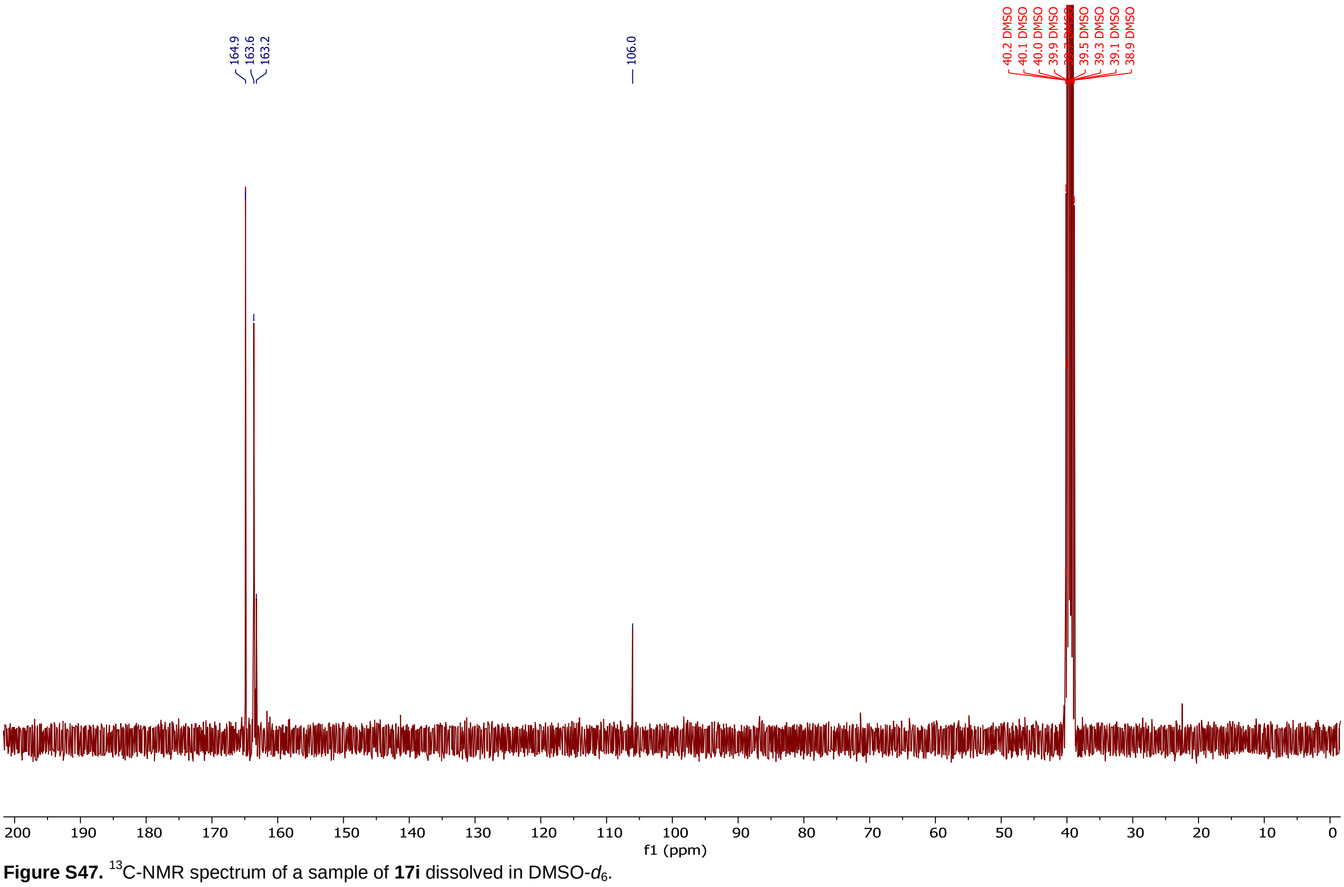

Figure S47. ^{13}C-NMR spectrum of a sample of **17i** dissolved in DMSO-d_6.

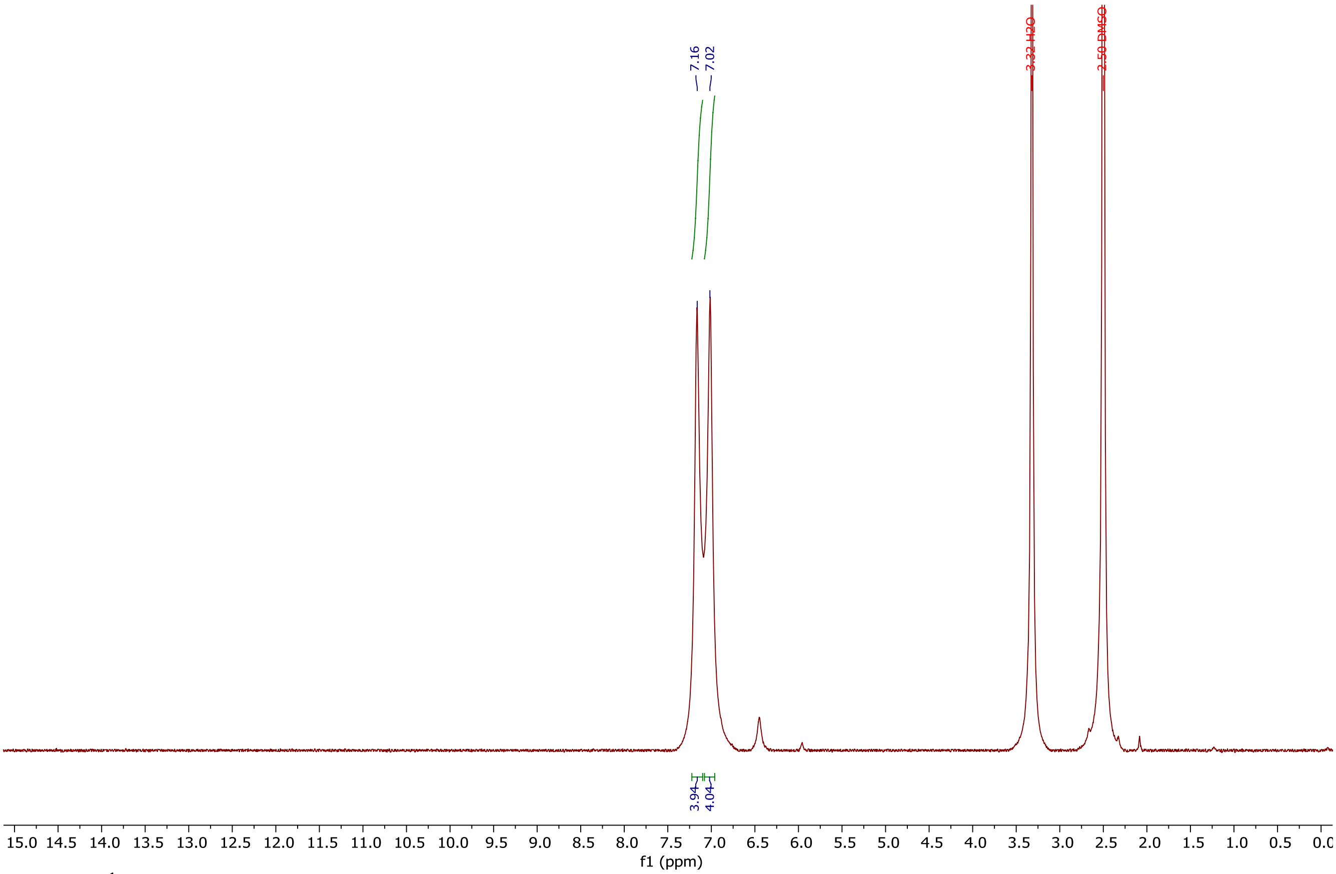

Figure S48. ^{1}H-NMR spectrum of a sample of **17** dissolved in DMSO-d_6.

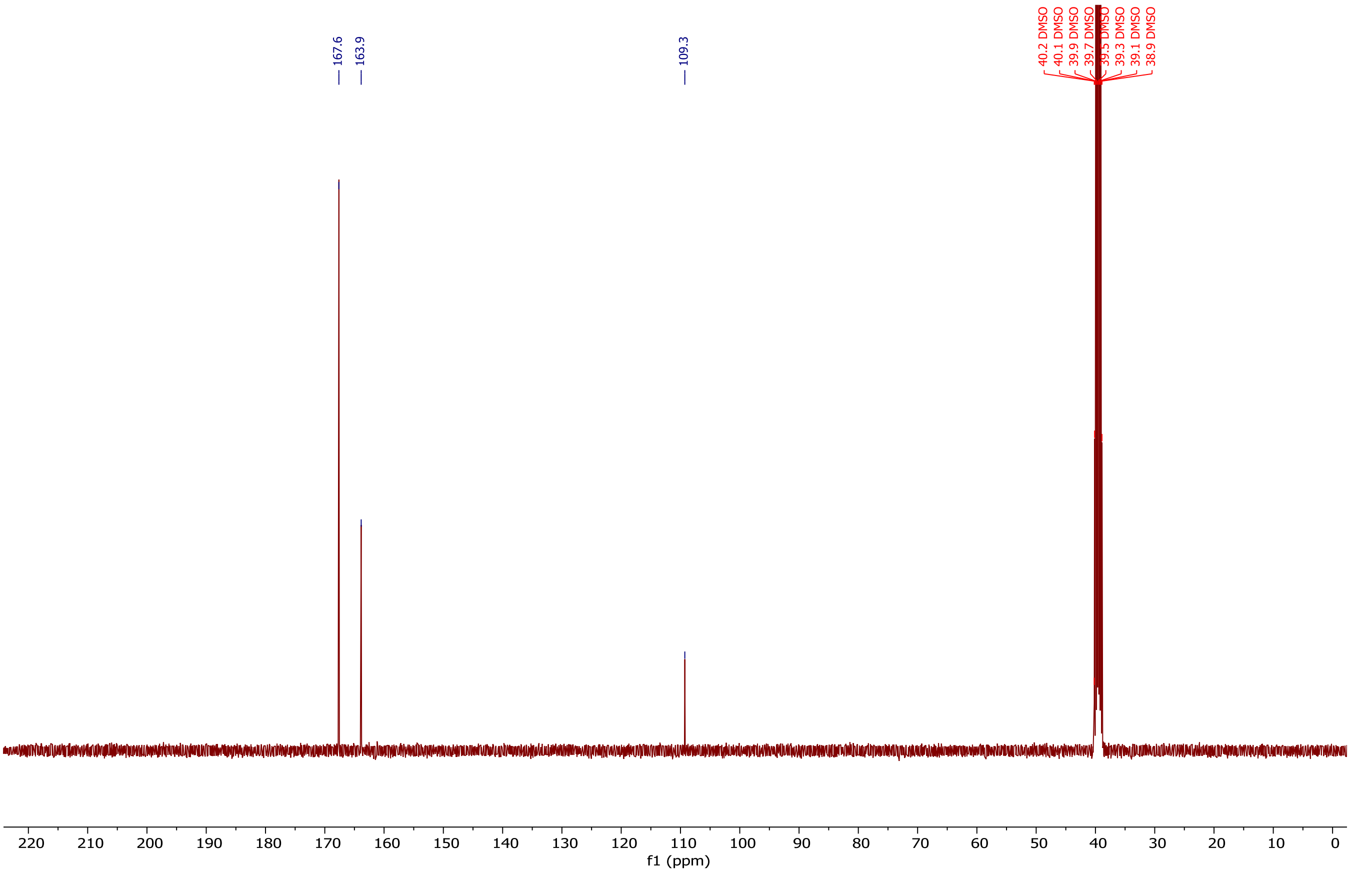

Figure S49. ^{13}C-NMR spectrum of a sample of **17** dissolved in DMSO-d_6.

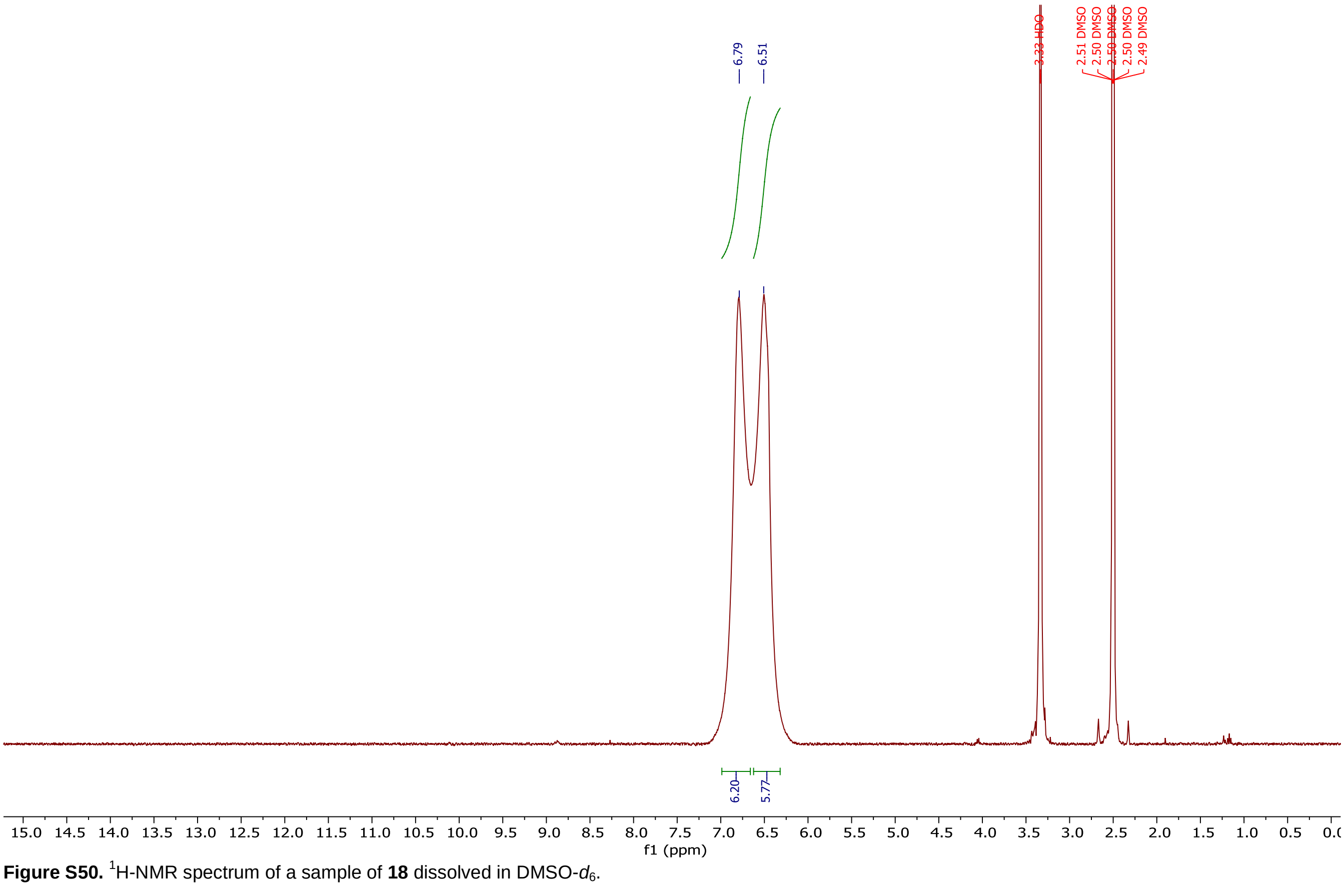

Figure S50. ^{1}H-NMR spectrum of a sample of **18** dissolved in DMSO-d_6.

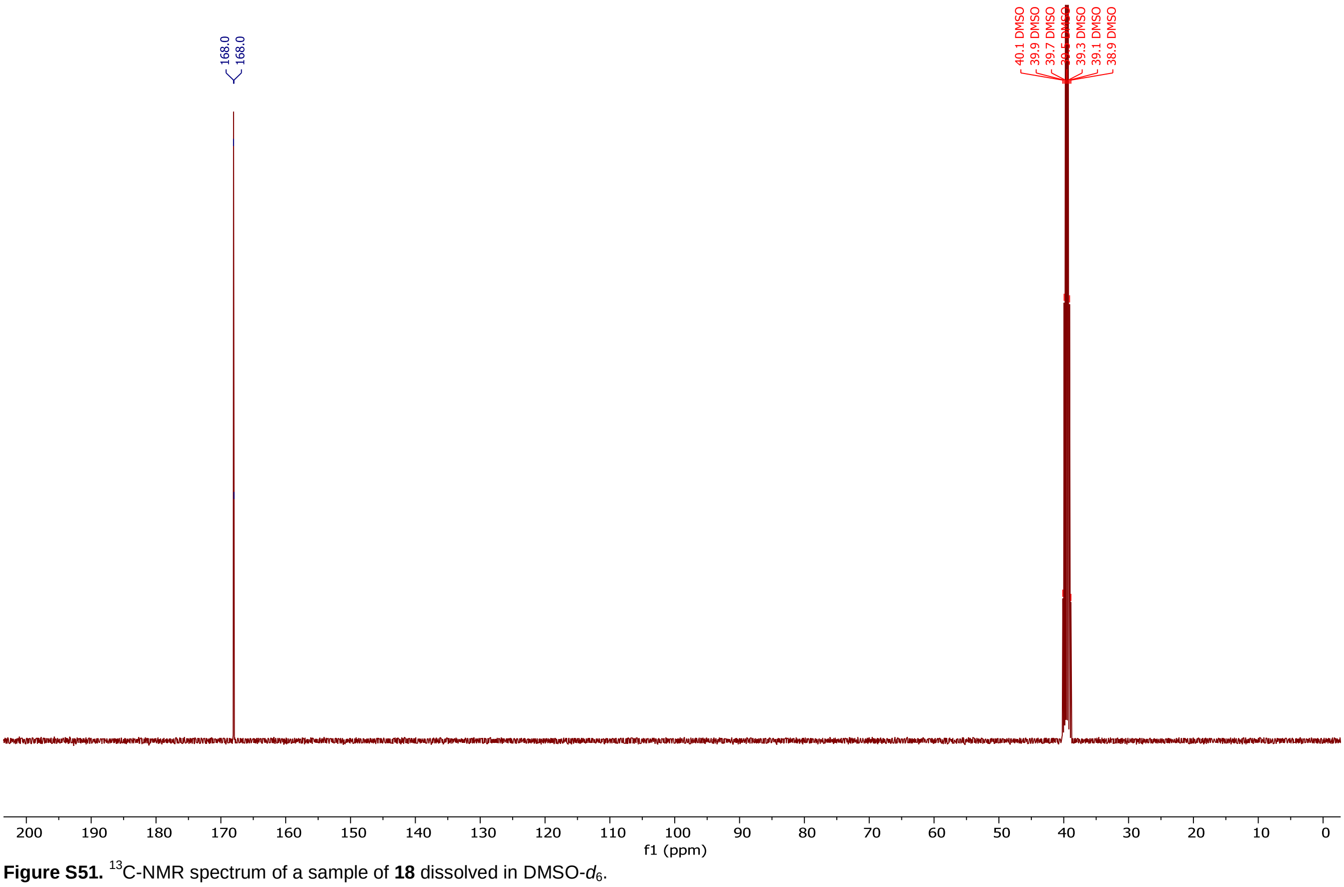

Figure S51. ^{13}C-NMR spectrum of a sample of **18** dissolved in DMSO-d_6.

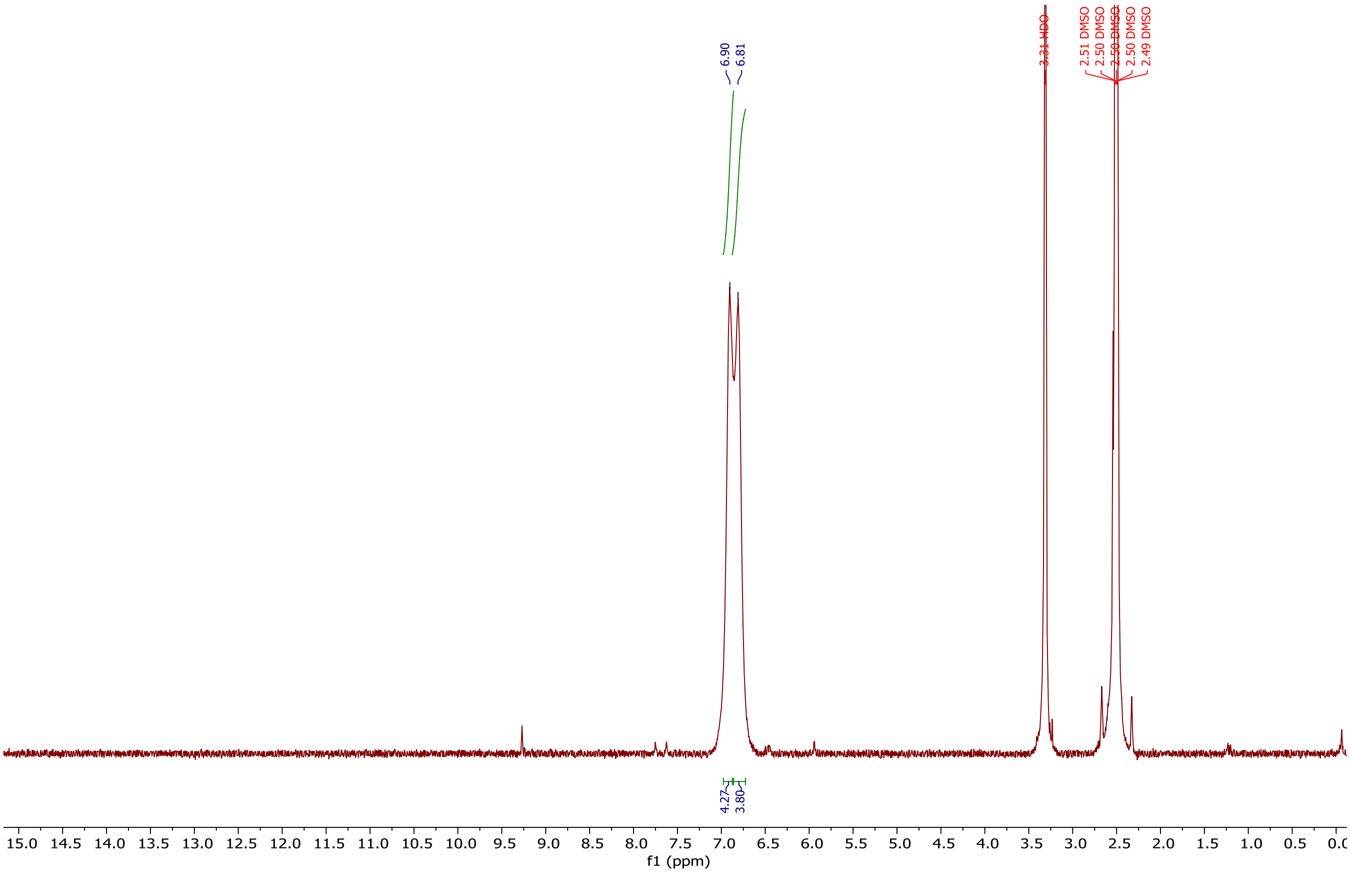

Figure S52. ^{1}H-NMR spectrum of a sample of **19** dissolved in DMSO-d_6.

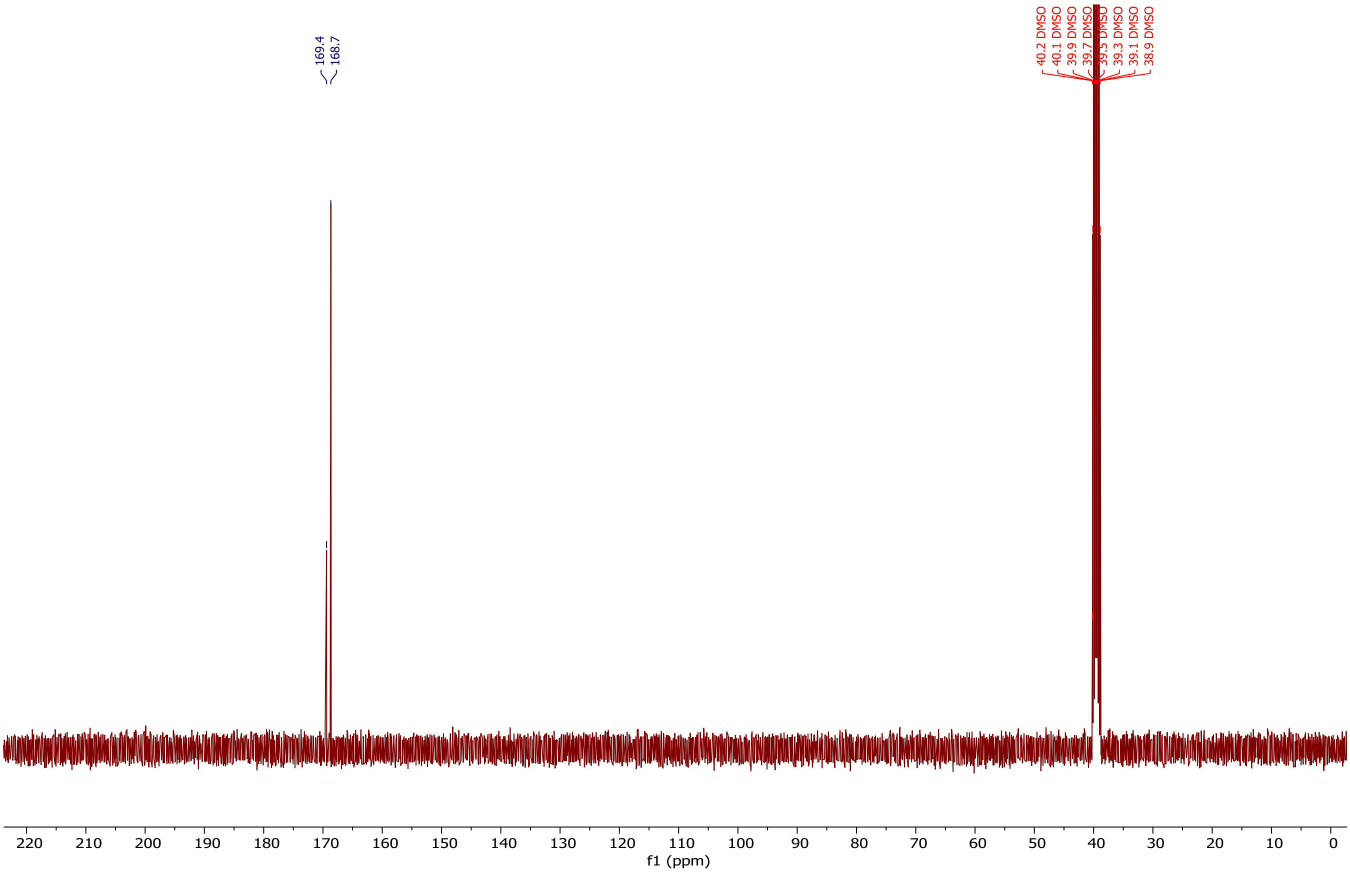

Figure S53. ^{13}C-NMR spectrum of a sample of **19** dissolved in DMSO-d_6.

FTIR Spectra

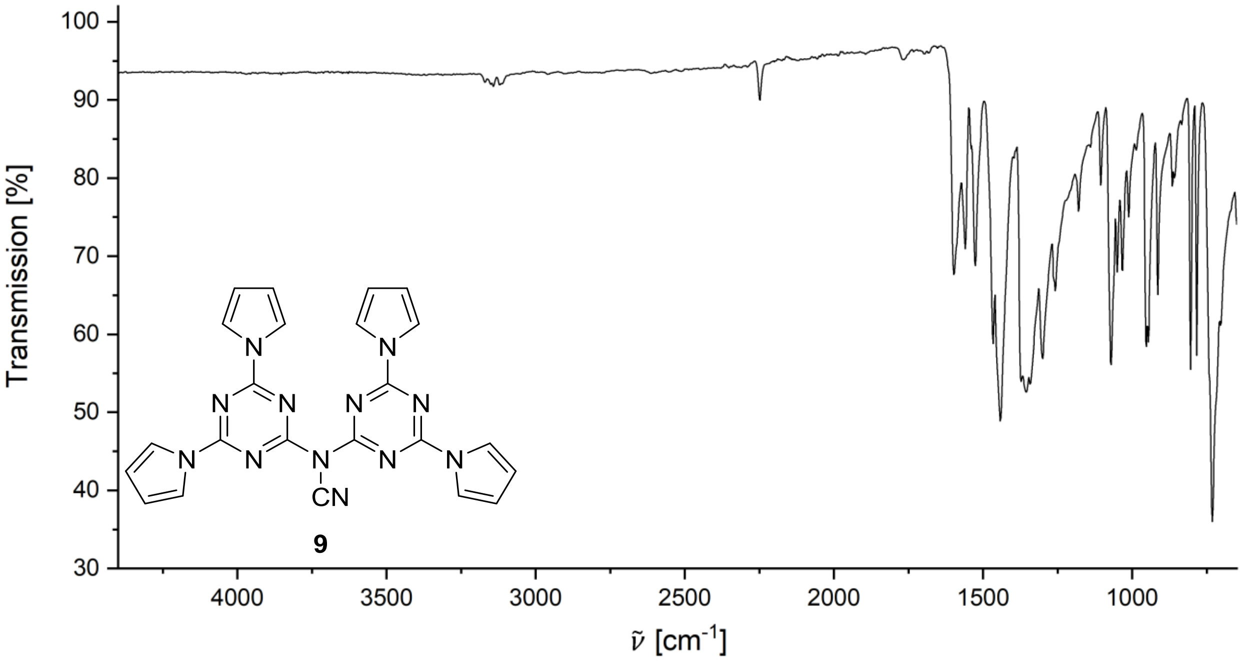

Figure S54. FTIR spectrum of **9**.

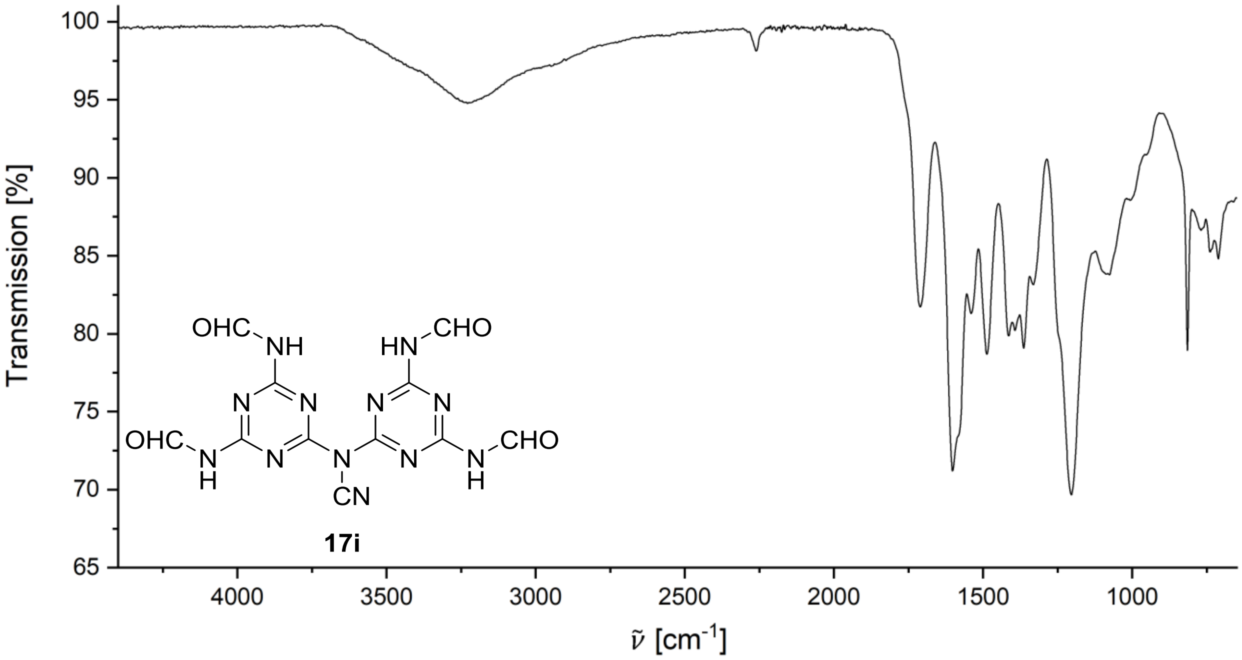

Figure S55. FTIR spectrum of the ozonolysis product of **9**, **17i**.

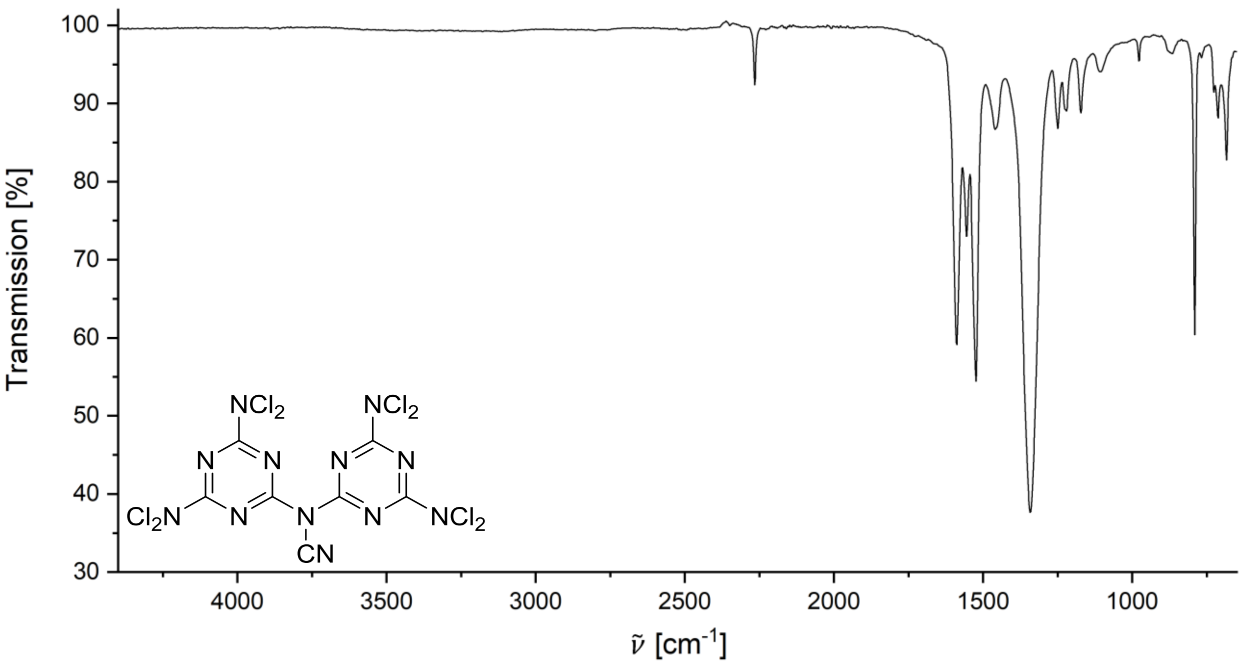

Figure S56. FTIR spectrum of the product obtained from the reaction of **17i** with acidified aqueous NaOCl.

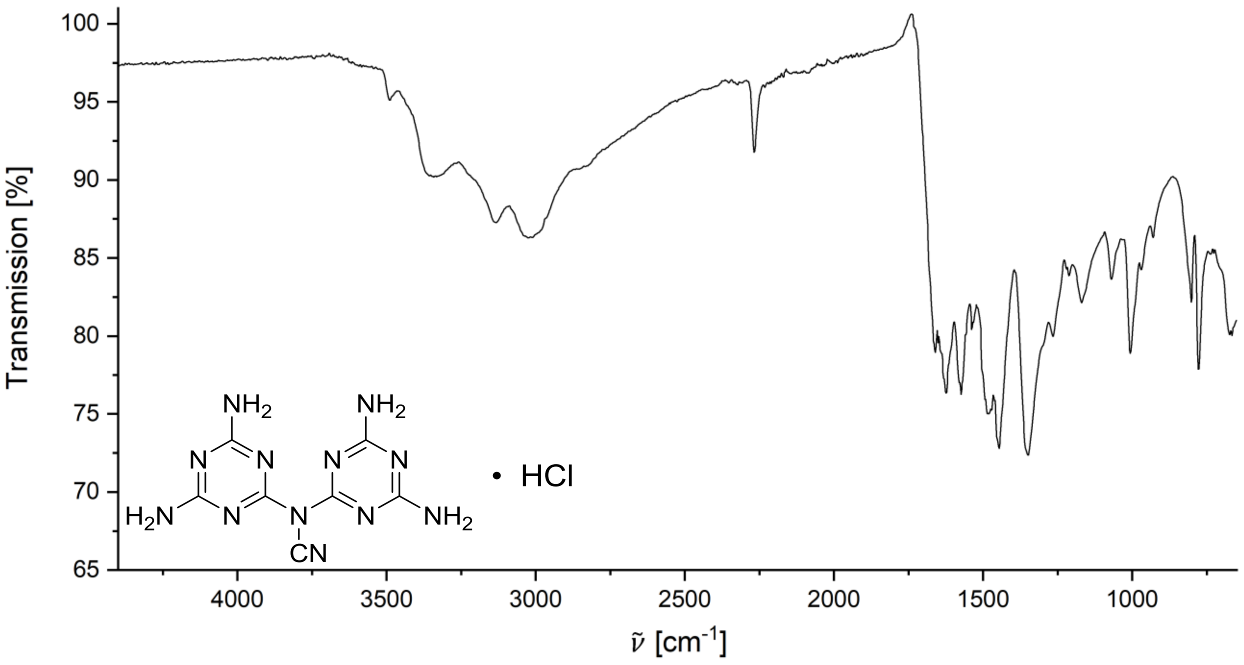

Figure S57. FTIR spectrum of the product obtained from the reaction of **17i** with acidified aqueous NaOCl, followed by treatment with *i*PrOH.

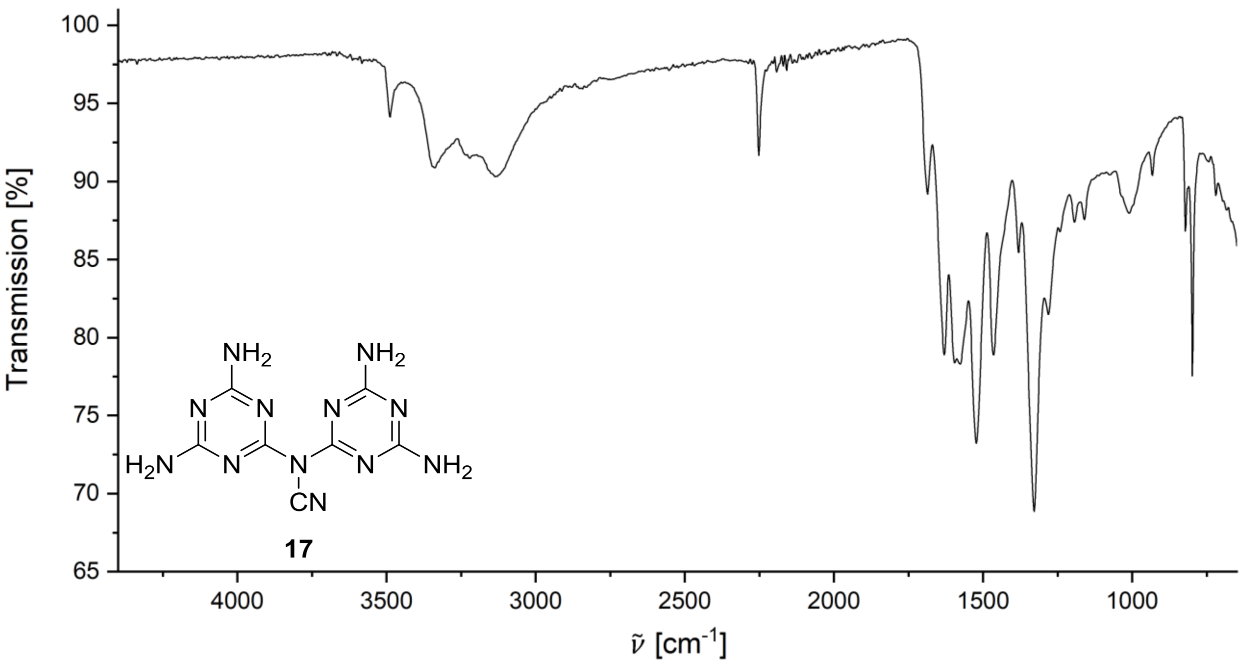

Figure S58. FTIR spectrum of **17**.

UV/Vis Spectra

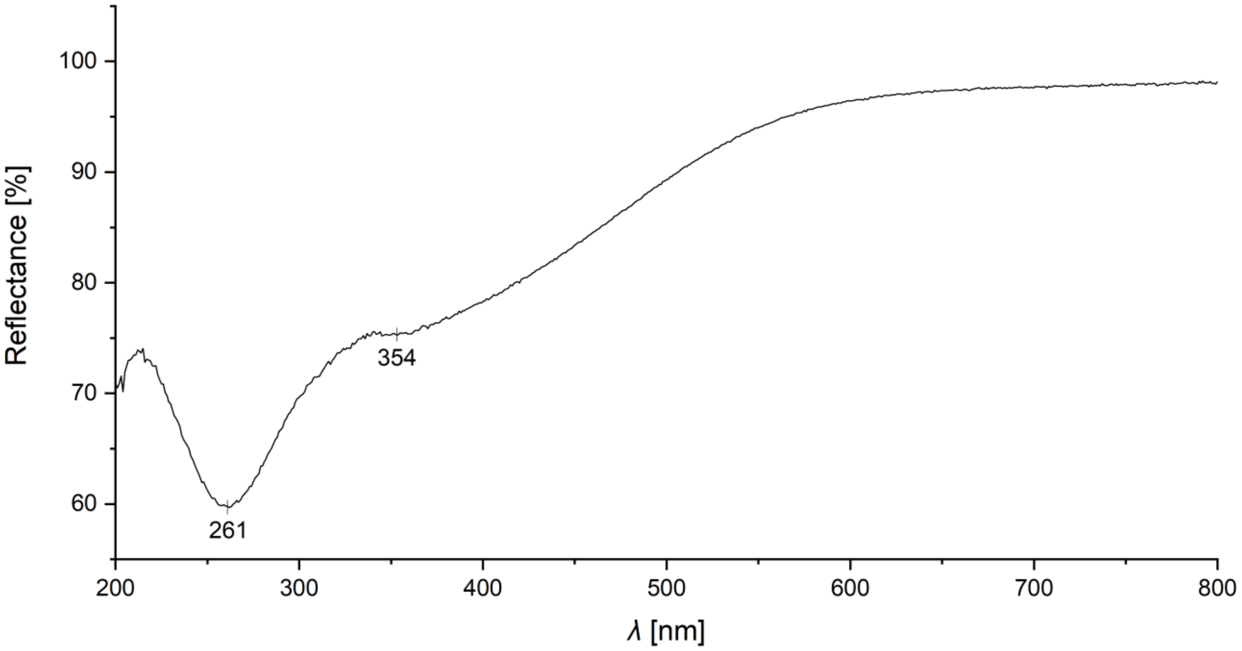

Figure S59. UV/Vis spectrum of **13**.

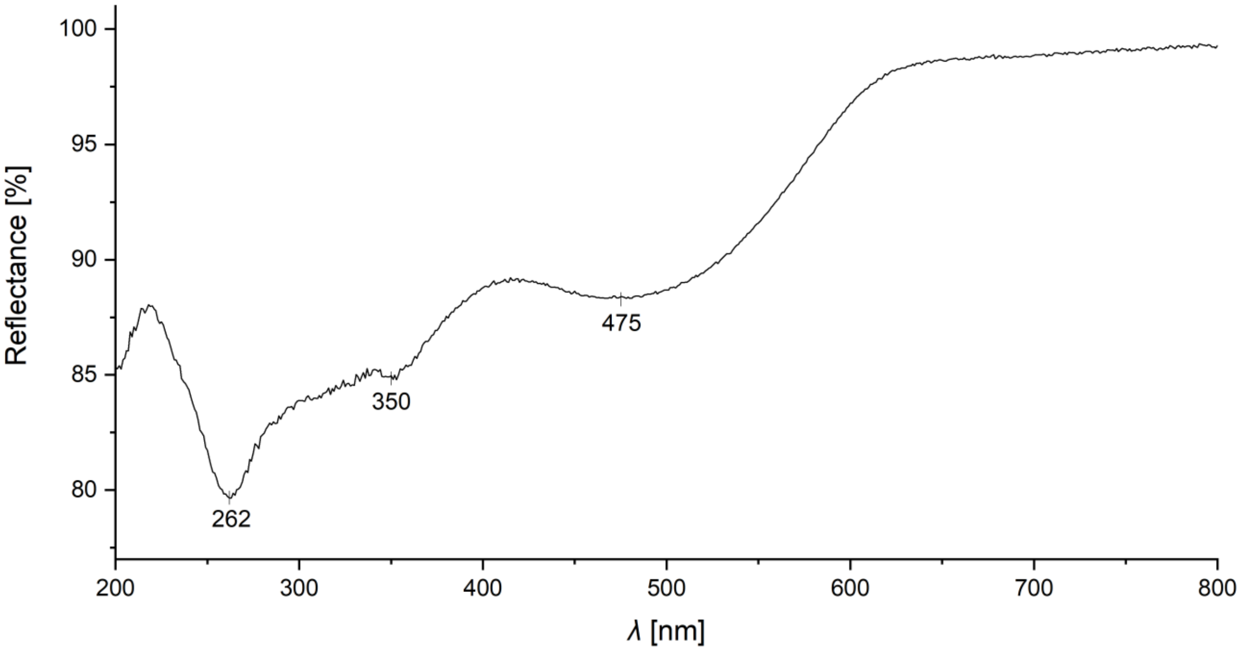

Figure S60. UV/Vis spectrum of **14**.

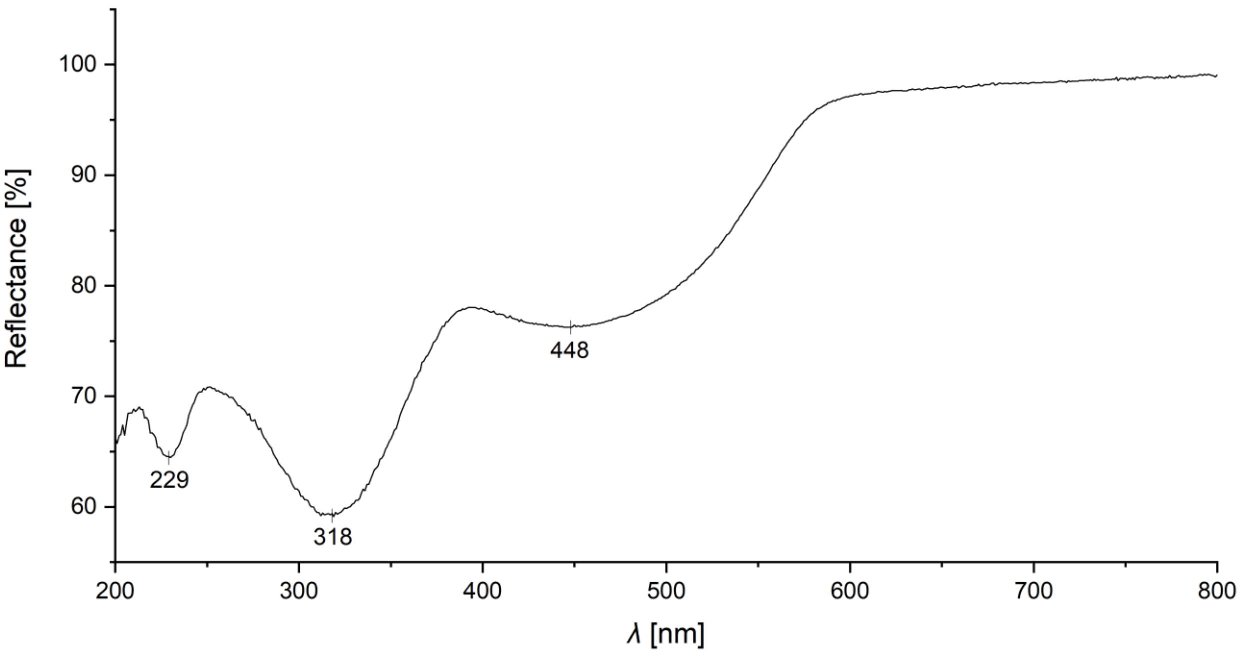

Figure S61. UV/Vis spectrum of commercially purchased azobenzene.

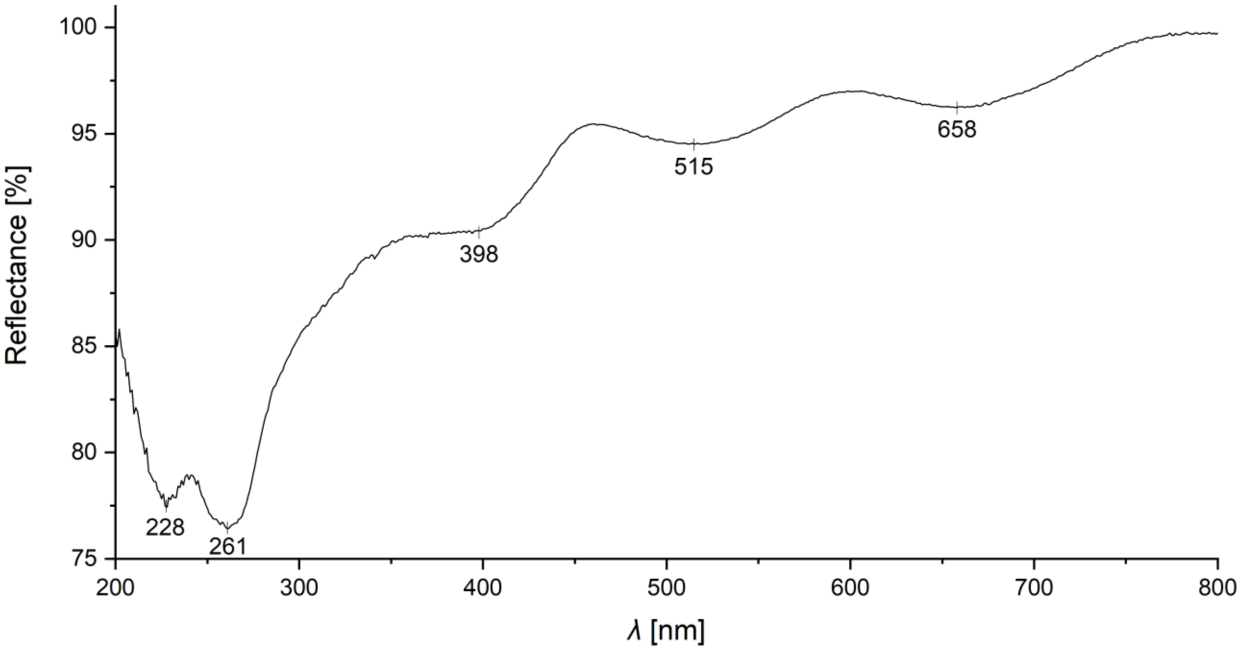

Figure S62. UV/Vis spectrum of **20·3H_2O**.

Pictures of Prepared Samples

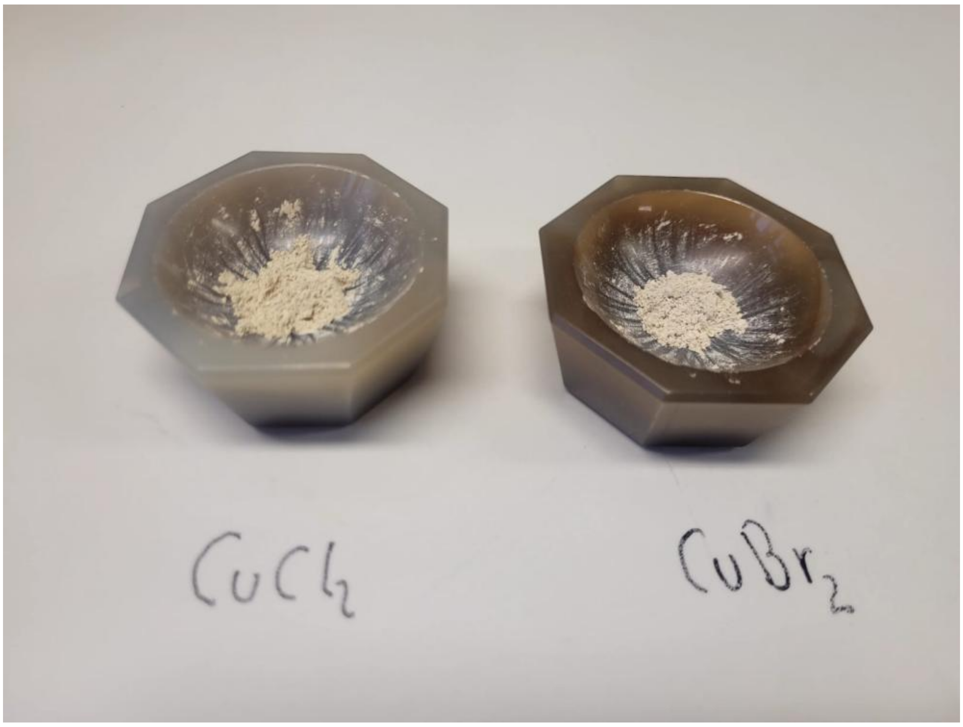

Figure S63. Products obtained from the reaction of melamine with $CuCl_2$ (left) and $CuBr_2$ (right). Their near colorless appearances corroborate the absence of any Cu(II) coordination complexes within the reaction products.

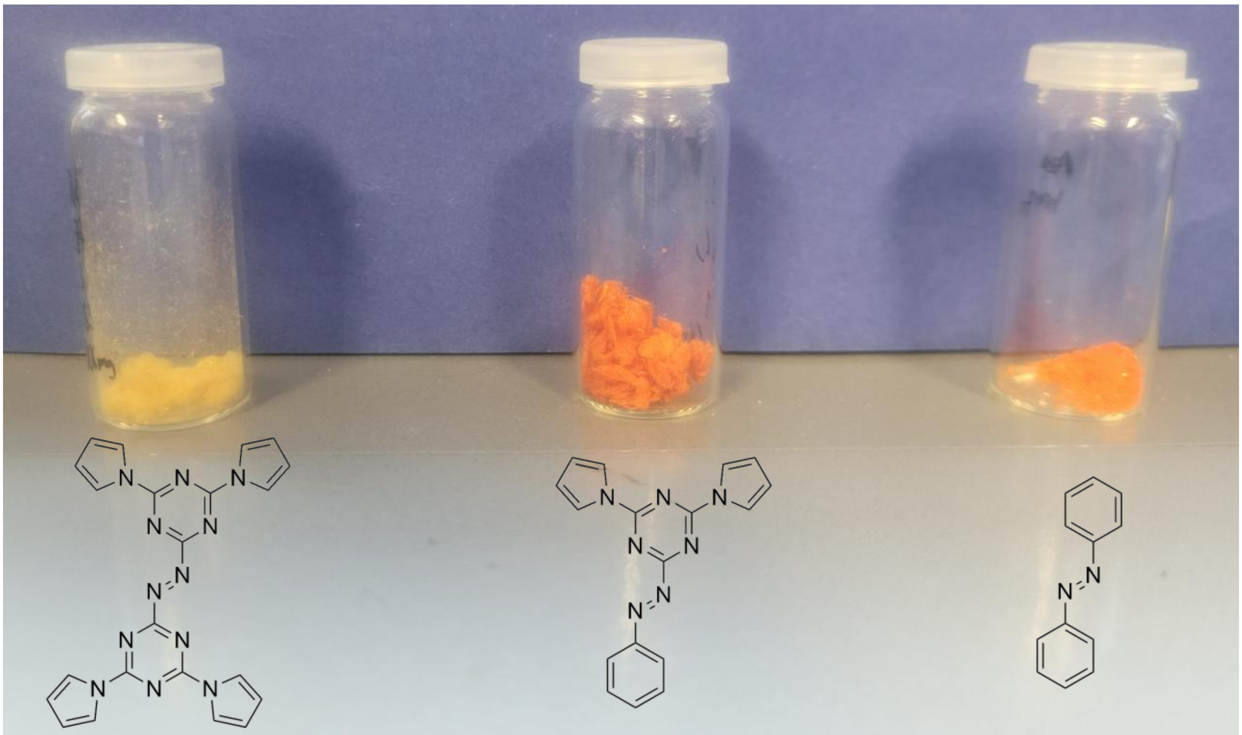

Figure S64. Samples of **13** (left), **14** (middle) and azobenzene (right) in comparison.

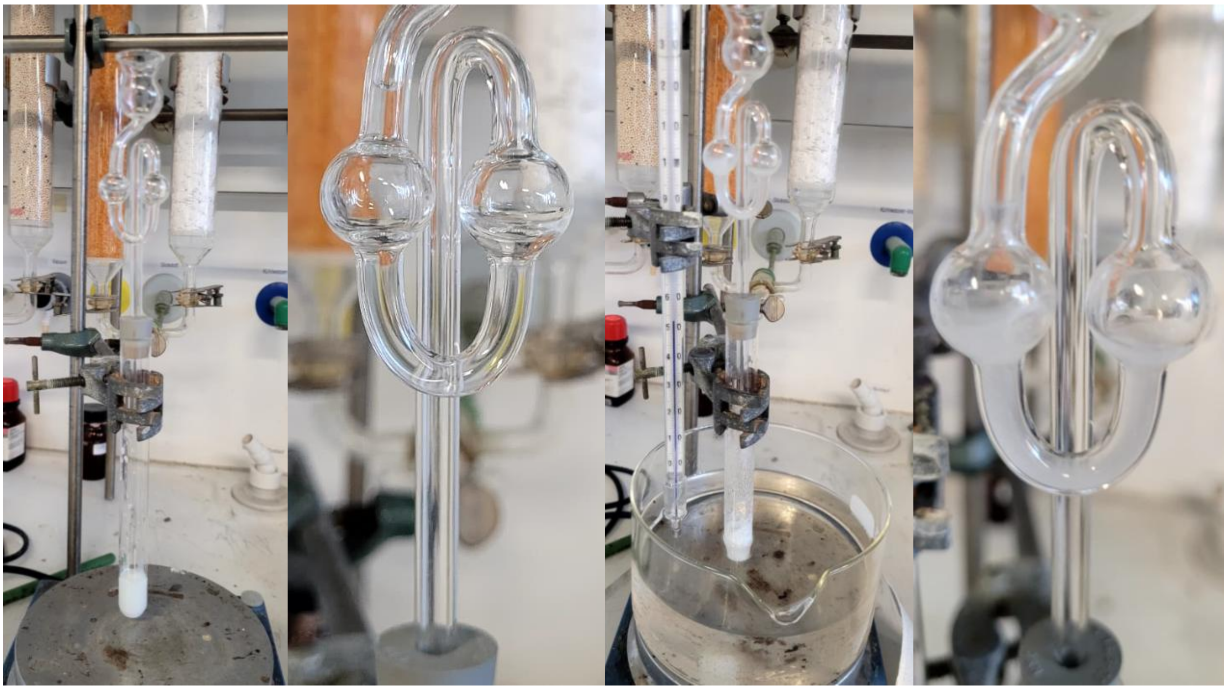

Figure S65. Test for CO_2 through a fermentation lock filled with aqueous $Ba(OH)_2$ during the treatment of **15i** with acidified aqueous NaOCl. The two pictures on the left were recorded at the start of the reaction, while the two pictures on the right were made after completion of the reaction.

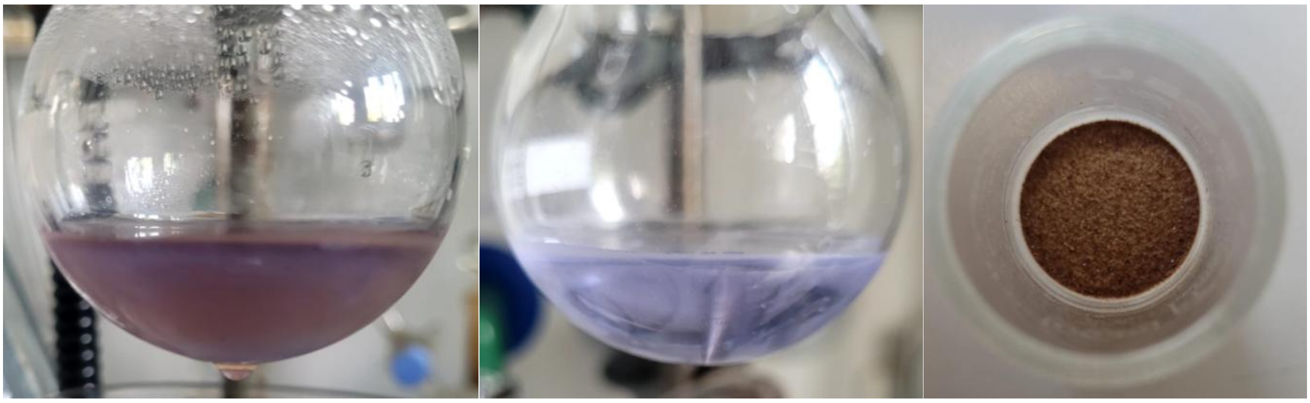

Figure S66. Picture of precipitated **20·3H$_2$O** (left) from aqueous solution and pictures of the resulting filtrate (center) and residue (right) after suction filtration.

References

[1] *APEX3*, Bruker AXS, Karlsruhe (Germany), **2018**.
[2] O. D. Rigaku, *CrysAlis^{Pro} 1.171.42.102a*, Oxford Diffraction/Agilent Technologies UK Ltd, Yarnton (United Kingdom), **2023**.
[3] G. M. Sheldrick, *Acta Crystallogr. Sect. A* **2008**, *64*, 112–122.
[4] K. Momma, F. Izumi, *J. Appl. Crystallogr.* **2008**, *41*, 653–658.
[5] *WinXPow 3.0.2.1*, STOE & Cie. GmbH, Darmstadt (Germany), **2011**.
[6] H. Rietveld, *J. Appl. Crystallogr.* **1969**, *2*, 65–71.
[7] *TOPAS 6*, Bruker AXS, Karlsruhe (Germany), **2016**.
[8] *Origin 2019b*, OriginLab Corporation, Northampton (USA), **2019**.
[9] *MestReNova 12.0.2*, Mestrelab Research, S.L., Santiago de Compostela (Spain), **2018**.
[10] G. R. Fulmer, A. J. M. Miller, N. H. Sherden, H. E. Gottlieb, A. Nudelman, B. M. Stoltz, J. E. Bercaw, K. I. Goldberg, *Organometallics* **2010**, *29*, 2176–2179.
[11] *ChemDraw 20*, PerkinElmer, Walton (USA), **2021**.
[12] B. V. Lotsch, W. Schnick, *Chem. Eur. J.* **2007**, *13*, 4956–4968.
[13] T. J. Koller, S. M. J. Endraß, M. Rösch, K. Witthaut, T. M. Klapötke, W. Schnick, *Angew. Chem. Int. Ed.* **2024**, *63*, e202404927.
[14] T. J. Koller, L. G. Balzat, J. Blahusch, A. Pichler, B. V. Lotsch, W. Schnick, *Eur. J. Inorg. Chem.* **2025**, *28*, e202500240.
[15] F. K. Kessler, T. J. Koller, W. Schnick, *Z. Anorg. Allg. Chem.* **2018**, *644*, 186–192.
[16] O. Loebich, C. J. Raub, *J. Less-Common Met.* **1977**, *55*, 67–76.
[17] W. P. Davey, *Phys. Rev.* **1925**, *25*, 753–761.

Author Contributions

Thaddäus J. Koller: Conceptualization (Lead), Formal Analysis (Lead), Investigation (Lead), Validation (Equal), Visualization (Lead), Writing – original draft (Lead), Writing – review & editing (Lead)
Alexander Pichler: Formal Analysis (Supporting), Investigation (Supporting), Validation (Supporting), Writing – review & editing (Supporting)
Johannes N. Singer: Formal Analysis (Supporting), Investigation (Supporting), Validation (Supporting), Writing – review & editing (Supporting)
Zehua Xu: Formal Analysis (Supporting), Investigation (Supporting), Validation (Supporting), Writing – review & editing (Supporting)
Vasiliki Valsamidou: Formal Analysis (Supporting), Investigation (Supporting), Validation (Supporting), Writing – review & editing (Supporting)
Reinhard M. Pritzl: Formal Analysis (Supporting), Validation (Supporting), Visualization (Supporting), Writing – review & editing (Supporting)
Wolfgang Schnick: Funding acquisition (Lead), Resources (Lead), Supervision (Lead), Validation (Equal), Writing – original draft (Supporting), Writing – review & editing (Supporting)
